# Improving sperm banking efficiency in endangered species through the use of a sperm selection method in brown bear (*Ursus arctos*) thawed sperm

**DOI:** 10.1186/s12917-017-1124-2

**Published:** 2017-06-26

**Authors:** L. Anel-Lopez, C. Ortega-Ferrusola, M. Álvarez, S. Borragán, C. Chamorro, F. J. Peña, J. Morrell, L. Anel, P. de Paz

**Affiliations:** 1 Animal Reproduction and Obstetrics, University of León, León, Spain; 2 ITRA-ULE, INDEGSAL, University of León, 24071 León, Spain; 3 Cabárceno Park, Cantabria, Spain; 4 Veterinary Anatomy, University of Leon, 24071 León, Spain; 5 Laboratory of Equine Reproduction and Equine Spermatology, Veterinary Teaching Hospital, University of Extremadura, Cáceres, Spain; 6 Swedish University of Agricultural Sciences (SLU), Clinical Sciences, Uppsala, Sweden; 7 Molecular Biology (Cell Biology), University of León, León, Spain

**Keywords:** Brown bear, Sperm selection, Androcoll-bear, Cryopreservation, Genetic resources bank

## Abstract

**Background:**

Sperm selection methods such as Single Layer Centrifugation (SLC) have been demonstrated to be a useful tool to improve the quality of sperm samples and therefore to increase the efficiency of other artificial reproductive techniques in several species. This procedure could help to improve the quality of genetic resource banks, which is essential for endangered species. In contrast, these sperm selection methods are optimized and focused on farm animals, where the recovery task is not as important as in endangered species because of their higher sperm availability. The aim of this study was to evaluate two centrifugation methods (300 x g/20 min and 600 x g/10 min) and three concentrations of SLC media (Androcoll-Bear −80, 65 and 50%) to optimise the procedure in order to recover as many sperm with the highest quality as possible. Sperm morphology could be important in the hydrodynamic relationship between the cell and centrifugation medium and thus the effect of sperm head morphometry on sperm yield and its hydrodynamic relationship were studied.

**Results:**

The samples selected with Androcoll-Bear 65% showed a very good yield (53.1 ± 2.9) although the yield from Androcoll-Bear 80% was lower (19.3 ± 3.3). The latter showed higher values of motility than the control immediately after post-thawing selection. However, both concentrations of colloid (65 and 80%) showed higher values of viable sperm and viable sperm with intact acrosome than the control. After an incubation of 2 h at 37 °C, the samples from Androcoll-Bear 80% had higher kinematics and proportion of viable sperm with intact acrosome. In the morphometric analysis, the sperm selected by the Androcoll-Bear 80% showed a head with a bigger area which was more elongated than the sperm from other treatments.

**Conclusions:**

We conclude that sperm selection with Androcoll-Bear at either 65% or 80% is a suitable technique that allows a sperm population with better quality than the initial sample to be obtained. We recommend the use of Androcoll-Bear 65% since the yield is better than Androcoll-Bear 80%. Our findings pave the way for further research on application of sperm selection techniques to sperm banking in the brown bear.

**Electronic supplementary material:**

The online version of this article (doi:10.1186/s12917-017-1124-2) contains supplementary material, which is available to authorized users.

## Background

The development of specific protocols for sperm handling adapted to endangered species, i.e., the Cantabrian brown bear (*Ursus arctos*) in Spain, is essential for the creation of ahigh quality genetic resource bank (GRB). In relation to bear GRB, our research group have evaluated different aspects that influence brown bear sperm quality after freezing and thawing: a species-specific extender [1], refrigeration or post-thawing incubation -thermal stress test- [2, 3] or using additives in the extender formulation [4].

Usually sperm samples have to be frozen in-situ because of the logistical problems related to the distance between the animals (in a zoo or their own habitat) and the laboratory. The use of selection methods may represent an important tool for improving the quality of sperm samples and removing the extender [5, 6], allowing us to carry out other artificial reproductive techniques such as sperm sex sorting or in vitro *fertilization* (IVF) [7, 8]. For success with such techniques, which are now being employed in brown bear, a high quality of sperm samples and the absence of egg yolk and glycerol are mandatory. In wild species, the logistical problems of transporting sperm samples to the laboratory in good condition are a barrier in the application of reproductive biotechnologies. One of the most important objectives of conservationist projects is to store as many high-quality samples as possible. Sperm selection optimizes the use of available space, allowing us to select useful cells (viable and motile sperm) and discard non-viable, apoptotic and non- motile sperm that impair the quality of the sample [9].

Previous studies with colloid centrifugation, using species -specific formulations of Androcoll, have reported beneficial effects in improving the motility, viability or mitochondrial activity of sperm samples, and removing dead cells, in red deer [10], boar [11], bull [12], or stallion [13], or buck [14]. In contrast, one of the main problems of the selection process with colloid centrifugation is the low yield in thawed samples. Anel-Lopez et al. [10] recovered 22 and 26% of the spermatozoa in red deer thawed semen, Garcia et al. [13] obtained yields of 20–40% from stallion thawed semen, whereas, Jimenez Rabadan et al. [14] achieved yields of 50% in fresh samples from buck and 14% in frozen-thawed samples.

Another factor that has been intensively studied in recent years is the sperm head morphology and its relationships with factors such as freezability [15, 16], fertility [17], or with the ability of sperm to migrate through artificial mucus [18]. The yield of sperm in a selection process could be related to the specific shape of individual sperm in the sample and their ability to penetrate the selection medium i.e. colloid, mucus etc. These differences among sperm inside the same sample could result in sperm subpopulations with different sperm motilities or a different degree of damaged nucleus. Sperm head morphology has been analysed as a fertility biomarker in various species, such as humans [19], boar [17] or sheep [20].

The aim of this study was to find an optimal procedure for the selection of frozen-thawed brown bear spermatozoa using Androcoll-Bear to separate viable spermatozoa from the extender and non-viable, non-motile cells after thawing. The effect of the density of the colloid on sperm yield, and the role of sperm head morphology on the selected sperm subpopulation were analysed.

## Methods

### Reagents and media

All the products used in this paper were of at least reagent grade and were acquired from Sigma (Madrid, Spain), unless otherwise stated. Fluorescence probes YO-PRO-1 and Hoechst33342 were purchased from Invitrogen (Barcelona, Spain). Stock solutions of the fluorescence probes were: PI: 7.5 mM; PNA-FITC: 0.2 mg/mL; YO-PRO-1: 50 μM. All fluorescent stocks were prepared in DMSO —except for PI, Hoechst 33,342 and PNA-FITC, which were prepared in water— and kept at −20 °C in the dark until needed, with the exception of the Hoechst, which was stored at 5 °C. Flow cytometry equipment, software and consumables were purchased from Beckman Coulter (Fullerton, CA, USA) or Becton Dickinson (San Jose, CA, USA). The medium for cytometry assessment was bovine gamete medium (BGM-3) composed of 87 mM NaCl, 3.1 mM KCl, 2 mM CaCl2, 0.4 mM MgCl2, 0.3 mM NaH2PO4, 40 mM HEPES, 21.6 mM sodium lactate, 1 mM sodium pyruvate, 50 μg/mL kanamycin, 10 μg/mL phenol red and 6 mg/mL BSA (pH 7.5).

For fixing samples, a 2% Glutaraldehyde solution was used, composed of BL-1 medium (glucose 14.6 mM, sodium citrate anhydrous 3.4 mM, sodium bicarbonate 2.4 mM.

### Animals and sperm collection

Animal handling and electroejaculation were performed in accordance with Spanish Animal Protection Regulation RD53/2013, which conforms to European Union Regulation 2010/63/UE. All experiments were approved by the Ethical Committee for Experimentation with Animals of León University, Spain (03–02/2010).

Ejaculates from 6 sexually mature (≥ 6 years old) brown bear (*Ursus arctos*) males were obtained by electroejaculation, during the breeding season (end of April to early July). The animals were housed in a half-freedom regimen in Cabarceno Park (Cantabria, Spain; 43° 21′ N, 3° 50′ W; altitude: 143 m), and fed with a diet based on chicken meat, bread, fruits and vegetables.

The animals were immobilized by teleanaesthesia and the electroejaculation was carried out as described by [2]. Immediately after collection, the volume and concentration of each ejaculate were recorded. Sperm concentration, sperm motility kinematics, and urospermia content were assessed as previously described by [2]. Low motility (< 50%) or urine-contaminated (>80 mg urea/dL) samples were discarded. The selected ejaculates were centrifuged at 600×g for 6 min at room temperature. The supernatants were discarded and each pellet was resuspended with the same volume of TES-Tris-Fructose (TTF) extender at room temperature (dilution 1:1), and cooled at −0.25 °C/min to a final temperature of 5 °C. The extender was prepared using TTF (300 mOsm/kg, pH 7.1); 20% egg yolk, supplemented with 2% EDTA, 1% Equex (Equex STM Paste; Minitüb, Tiefenbach, Germany), 0.302 mg penicillin G sodium salt/mL and 0.625 mg dihydrostreptomycin sesquisulfate/mL. The glycerol concentration was adjusted to a final concentration of 6%.

### Sperm cryopreservation

Sperm samples were diluted to a final concentration of 100 × 10^6^ sperm/mL. After 2 h of equilibration at 5 °C, 0.25 ml straws were frozen in a programmable biofreezer (Kryo 560–16 Planer™, Planer plc, Sunbury-On-Thames, UK) at −20 °C/min up to −100 °C, transferred to liquid nitrogen containers and stored for a minimum of one year. Thawing was performed by dropping straws in water at 65 °C for 6 s.

### Experimental design

Seven straws from each male were thawed and pooled to avoid differences in the starting concentration. An aliquot of 150 μL was used as an unselected control. Three proportions of Androcoll-Bear were assessed, at 80, 65 and 50%.

A further 6 aliquots of 150 μL were each layered on top of a column (1 mL of Androcol-Bear in a 1.5 mL Eppendorf® tube; 2 per each concentration (80, 65 and 50%)). The centrifugation was carried out with two different protocols; CM1: 300 x g/20 min and CM2: 600 x g/10 min. The resulting sperm pellets were harvested and resuspended in 50 μL of TES-Tris-Fructose. Selected samples and unselected samples were assessed, then incubated for 2 h at 37 °C and re-assessed. The number of spermatozoa was counted before and after single layer centrifugation (SLC) using a Nucleocounter®SP-100 (Chemometec, Allerod, Denmark) to determine the yield of the separated sperm population.

### Computer-assisted sperm analysis

Total motility (%; TM), progressive motility (%; PM), curvilinear path velocity (μm/s; VCL) and linearity (%; LIN) were assessed using a CASA system (Computer Assisted Sperm Analysis), as described by [21].

Image sequences were saved and analysed afterwards using the editing facilities provided by ISAS (v. 12, Integrated Semen Analyser System; Poriser, Valencia, Spain). Sperm were considered motile when VCL > 10 μm/s and progressive if VCL >25 and straightness (STR) >80%. The progressive sperm subpopulations were classified according to velocities as follows: Slow (VCL <25), Medium (VCL >25 and <65), Rapid (VCL >65). Other events different from spermatozoa were removed, and settings were adjusted in each case to assure a correct track analysis.

### Evaluation of sperm viability, apoptotic markers and acrosomal status

Several physiological traits (viability, apoptosis and acrosomal status) were assessed using fluorescent probes and flow cytometry, as described in a previous study Anel-Lopez et al. [21]. Spermatozoa stained in these two solutions were incubated for 10 min in the dark before analysis with a CyAn ADP flow cytometer (Beckman Coulter, Brea, CA, USA), equipped with semiconductor lasers emitting at 405 nm (violet; Hoechst 33,342) and 488 nm (blue; YO-PRO-1, PNA-FITC and PI). Filters used for each fluorochrome were 450/50 (blue) for Hoechst 33,342, 530/40 (green) for YO-PRO-1 and PNA-FITC and 613/20 (red) for PI.

The sperm subpopulations obtained go as follow; PI (+) (dead sperm), PI (−) (Viable sperm), PNA-FITC (+) (Acrosoma damaged), PNA-FITC (−) (Acrosoma non damaged), Yo-Pro1 (+) (early permeability changes in the membrane).

### Morphometric assessment

The sperm samples (before and after Androcoll-Bear) were fixed in 2% glutaraldehyde and smeared on microscope slides, air dried and then stained using Diff-Quick (QCA, Tarragona, Spain). For staining, slides were immersed for 10 min in solution A, for 15 min in solution B and then rinsed using distilled water, air dried and mounted with Entellan (Sigma-Aldrich). Samples were examined using a Nikon Eclipse E600 microscope (Nikon, Tokyo, Japan) equipped with a ×60 bright field objective (×500). An average of 200 cells in each sample were photographed using a Nikon DF1200 digital camera (Nikon, Tokyo, Japan) and the pictures were processed using the NIS Elements v.3 image analysis system. The morphometric analysis was carried out using a semi-automatic macro that allowed the operator to discard the sperm heads in the image that did not meet the technical requirements for the study (e.g. overlapping cells and the presence of staining artefacts). For each cell, the following parameters were calculated: area (A; μm^2^), length (L; μm), perimeter (P; μm), width (W; μm) and elongation (L - W)/(L + W).

### Statistical analysis

Data were analysed using the SAS™ V.9.1. Package (SAS Institute Inc., Cary, NC, USA). Results are shown as means and standard errors of the mean. Data were tested for normality (Shapiro-Wilk test) and arcsin square-root transformation was used to normalise the data before analysis when necessary. Analyses of the data were carried out using linear mixed-effects models (MIXED procedure, ML method), including proportion of Androcoll-B (80, 65 and 50%), centrifugation protocols (V1 and V2) and incubation time after thawing (0 vs. 2 h) as fixed factors, and males as random effect. Significant fixed effects were further analysed using multiple comparisons of means with Tukey contrasts. A significance level of *P* < 0.05 was used.

## Results

There were no significant differences (*P* > 0.05) between centrifugation methods (CM1 and CM2) in any of the sperm parameters assessed. The centrifugation method had no effects on sperm quality, and therefore it was not considered as a weight factor.

### Sperm recovery after SLC

The straws had an initial sperm concentration of 100 × 10^6^ mL^−1^. After SLC, there were differences in yield (*P* < 0.05) among the different treatments (Table 1). Androcoll-Bear at 80% showed the lowest values (19.3 ± 3.3) while Androcoll-Bear at 65% showed a higher recovery, being more than double that of Androcoll-Bear 80 (53.1 ± 2.9). Androcoll-Bear 50 was used as a negative control showing values similar to those obtained with a conventional sperm washing centrifugation.

**Table 1:** Effect of the selection with different concentrations of Androcoll-Bear on yield in brown bear sperm

TREATMENT	%YIELD
Androcoll-Bear 50	79.6 ± 1.8; A
Androcoll-Bear 65	53.1 ± 2.9; B
Androcoll-Bear 80	19.3 ± 3.3; C

Treatments: Androcoll-Bear 50%, Androcoll-Bear 65% and Androcoll-Bear 80%. Data are shown as the model-derived mean ± s.e.m

(^A,B,C^) Capital letters show differences (*P* < 0.05) among treatments

### Sperm motility

**Fig. 1:**
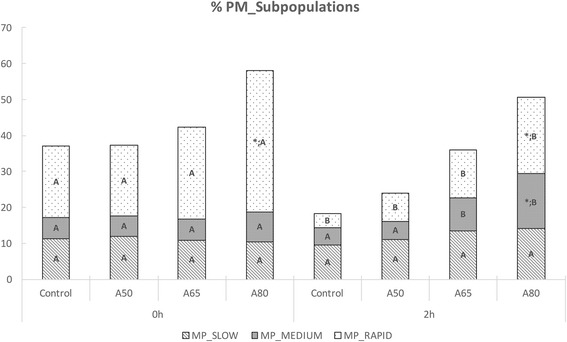
Progressive motility and its different subpopulations (slow, medium and rapid sperm) just after thawing-selection (0 h) and after an incubation at 37 °C for (2 h). Treatments: A control unselected (Control), Androcoll-Bear 50% (A50), Androcoll-Bear 65% (A65) and Androcoll-Bear 80% (A80). (*) Asterisks show differences (*P* < 0.05) between a specific Androcoll concentration and its Control. (^A,B^) Capital letters show differences (*P* < 0.05) for the same treatment between time (0 and 2 h)

After post-thawing selection, sperm motility was improved for TM, showing higher values (*P* < 0.05) for Androcoll-Bear 80 than the Control (Table 2). Neither Androcoll-Bear 50 nor Androcoll-Bear 65 showed differences with respect to the control for TM and PM. In the same way, Androcoll-Bear 80 showed the highest values of PM (*P* < 0.05) and the highest proportion of rapid PM sperm (Fig. 1). Other kinematic parameters such as VAP or LIN were improved significantly (*P* < 0.05) by Androcoll-Bear 80 compared to the control (Table 2).

**Table 2:** Effect of the selection with different concentrations of Androcoll-Bear on sperm motility in brown bear sperm

TIME	TREATMENT	%TM	%PM	VAP	LIN
0 h	Control	68.4 ± 3.8 A	37.1 ± 0.8 A	53.4 ± 2.2 A	39.8 ± 2.2 A
	A50	64.6 ± 4.2 A	37.2 ± 1.9 A	53.1 ± 2.8 A	42.6 ± 1.6 A
	A65	72.5 ± 4.1 A	42.3 ± 2.6 A	59.1 ± 3.5 A	43.3 ± 1.9 A
	A80	84.7 ± 1.6 *;A	58 ± 2.4 *;A	71.6 ± 3.3 *;A	50.9 ± 2.5 *;A
2 h	Control	39.1 ± 4.5 B	18.4 ± 3 B	32.4 ± 3.1 B	33 ± 1.3 B
	A50	41.5 ± 5.3 B	23.9 ± 3.2 B	35.7 ± 3.1 B	37.5 ± 1.5 B
	A65	57.3 ± 5.1 B	36.1 ± 3.6 *;A	42.1 ± 3.4 B	39.7 ± 1.6 A
	A80	74.9 ± 3.6 *;B	50.7 ± 3 *;A	49.1 ± 3 *;B	40.6 ± 2.1 *;B

The table shows: total motility (%TM), progressive motility (%PM), velocity average pathway (VAP) and linearity (LIN). Time: Just after thawing (0 h) and after an incubation of 2 h at 37 °C (2 h). Treatments: Androcoll-Bear 50% (A50), Androcoll-Bear 65% (A65) and Androcoll-Bear 80% (A80). Data shown are the model-derived mean ± s.e.m

(*) Asterisks show differences (*P* < 0.05) between Androcoll proportion and its Control

(^A,B^) Capital letters show differences (*P* < 0.05) for the same treatment between time (0 and 2 h)

After 2 h of incubation at 37 °C, the kinematics had decreased in all treatments (*P* < 0.05). Samples selected using Androcoll-Bear 80 maintained higher values of TM than the other treatments. Either Androcoll-Bear 80 or Androcoll-Bear 65 maintained higher values (*P* < 0.05) of PM (Table 2) than the control. In addition, after incubation at 37 °C for 2 h, Androcoll-Bear 80 was able to maintain a higher proportion (*P* < 0.05) of progressive rapid sperm than the control (Fig. 1). Moreover, VAP and LIN were reduced with time, but the samples selected by Androcoll-Bear 80 kept significant higher values (*P* < 0.05) than the control after the incubation (Table 2).

### Sperm viability, apoptosis and acrosomal status

Post-thawing, selected samples (Androcoll-Bear 65 and Androcoll-Bear 80) showed higher values (*P* < 0.05) of viable sperm than the Control (Table 3). In the same way, Androcoll-Bear 65 and Androcoll-Bear 80 samples showed higher values (*P* < 0.05) of viable sperm with intact acrosome (Table 3) than the control. Meanwhile, Androcoll-Bear 50 did not show any difference to the control, either for viability or for viable sperm with intact acrosome. No differences were observed in the percentage of apoptotic cells just after thawing for any treatment (Table 3).

**Table 3:** Effect of the selection with different concentrations of Androcoll-Bear on sperm membrane and acrosomal status

TIME	TREATMENT	%Viab	%Apoptosis	%Viable_Acro
0 h	Control	46.7 ± 4 A	24.2 ± 4.3 A	75.4 ± 3.6 A
	A50	58.4 ± 2.7 A	16 ± 2.4 A	81.6 ± 2.1 A
	A65	73.9 ± 2.4 *;A	15 ± 2.8 A	88 ± 1.4 *;A
	A80	78.1 ± 2.3 *;A	15.3 ± 2.6 A	93 ± 1.2 *;A
2 h	Control	31.7 ± 3.9 B	24.1 ± 5.5 A	55.9 ± 5.2 B
	A50	45.6 ± 3.6 B	16.9 ± 1.9 A	64.1 ± 4 B
	A65	50.7 ± 5.1 *;B	16.8 ± 1.7 A	68.4 ± 4.3 B
	A80	56.1 ± 4.2 *;B	19.6 ± 2 A	75.9 ± 4.2 *;B

The table shows: viability (%Viab), apoptosis (%Apoptosis) and viable sperm with intact acrosome (%Viable_Acro). Time: immediately after thawing (0 h) and after an incubation of 2 h at 37 °C (2 h). Treatments: Androcoll-Bear 50% (A50), Androcoll-Bear 65% (A65) and Androcoll-Bear 80% (A80). Data shown are the model-derived mean ± s.e.m

(*) Asterisks show differences (*P* < 0.05) between a specific Androcoll-Bear proportion and its Control

(^A,B^) Capital letters show differences (*P* < 0.05) for the same treatment between time (0 and 2 h)

After 2 h of incubation, all treatments showed lower values (*P* < 0.05) of viability and viable sperm with intact acrosome (Table 3). In contrast, no differences were observed in the percentage of apoptotic sperm after incubation. Androcoll-Bear 80 samples maintained higher values (*P* < 0.05) of viable sperm and viable sperm with intact acrosome than the Control (Table 3). There were no differences among treatments in the percentage of apoptotic sperm (Table 3).

### Sperm morphometry

The sperm samples selected with Androcoll-Bear 80 showed significantly (*P* < 0.05) higher values for all the morphometric parameters assessed (Area, length, width, perimeter and elongation) than the control samples (Table 4). In contrast, samples selected with Androcoll-Bear 65 showed significantly lower values (*P* < 0.05) than the Control for the area, length and width. Meanwhile, Androcoll-Bear 50 showed similar results to the control for the perimeter, length and elongation (*P* > 0.05) and lower values for area and width (*P* < 0.05) (Table 4).

**Table 4:** Effect of the selection with different concentrations of Androcoll-Bear on sperm morphology

	Area	Perimeter	Length	Width	Elongation
Control	19.78 ± 0.06 A	16.03 ± 0.02 A	6.01 ± 0.01 A	3.29 ± 0.01 A	1.3934 ± 0.0024 A
A50	19.55 ± 0.04 B	15.96 ± 0.02 AB	5.99 ± 0.01 AB	3.26 ± 0 B	1.3910 ± 0.0016 AB
A65	19.48 ± 0.04 B	15.93 ± 0.02 B	5.98 ± 0.01 B	3.25 ± 0 B	1.3985 ± 0.0016 AC
A80	20.21 ± 0.03 C	16.2 ± 0.01 C	6.08 ± 0.01 C	3.32 ± 0 C	1.4007 ± 0.0015 C

The table shows: Area (μm^2^), Perimeter (μm), Length (μm), Width (μm), Elongation.

Samples were assessed after thawing and after selection. Treatments: Androcoll-Bear 50% (A50), Androcoll-Bear 65% (A65) and Androcoll-Bear 80% (A80). Data are shown the model-derived mean ± s.e.m

(^A,B^) Capital letters show differences (*P* < 0.05)

## Discussion

The effect of the selection with different proportions of Androcoll-Bear on bear sperm quality and yield were assessed to optimize the protocol to be used with Androcoll-Bear in the selection of brown bear (*Ursus arctos*) sperm and then improving the sperm banking efficiency.

The Cantabric population of brown bear in Spain is endangered and sperm samples are very difficult to obtain; for this reason, every spermatozoon is valuable. In order to optimize sperm selection to get the best quality and the highest yield, three concentrations of Androcoll-Bear and two centrifugation methods were tested. No differences were observed between centrifugation methods, and therefore we recommend CM2 since it takes half the time and achieves the same sperm quality and yield.

Some species-specific colloids of Androcoll have been tested under different handling conditions, obtaining varied results. Our results show an improvement in sperm quality with Androcoll-Bear 65 and especially with Androcoll-Bear 80. These results are in concordance with Anel-Lopez et al. [10] who found several improvements in the sperm quality (sperm motility, cells with intact acrosomes, sperm viability and cells with mitochondrial activity) from red deer after thawing and selecting, in sperm samples obtained either by electroejaculation or post-mortem. In addition, these improvements were maintained after an incubation of 2 h at 37 °C compared to the control, in agreement with our results with Androcoll-Bear 80 in the present study. In the same way, positive effects on sperm quality were reported by [13] in stallion semen.

However, some authors have reported an absence of effect in individual parameters when selecting sperm samples with Androcoll. Bucci et al. [22] working with boar sperm did not observe any significant difference between the control and the selected samples in terms of percentage of damaged acrosomes, although other parameters of sperm quality were improved.

The most important problem when using sperm selection methods is the low yield, especially with frozen-thawed samples [10, 14]. Referring to yield, there were differences among colloid proportions. Androcoll-Bear 65 samples showed good recovery and an improvement in sperm viability. In addition, the sperm maintained higher values of viability not only immediately after selection, but also after incubation. This formulation Androcoll-Bear 65 could help us to optimize the space in GRBs, since non-viable sperm are discarded. Moreover, it could improve processes such as sperm sex sorting. When sperm samples are sorted, the presence of a high percentage of non-viable sperm compromises the efficiency of the sorting process, increasing the time required to sort the sample, which impairs its quality, and decreases the efficiency of this technique [23]. The use of selection methods such as single layer centrifugation enables all of the extender to be removed from the sample as well as facilitating the application of other techniques such as sperm sex sorting or in vitro *fertilization* [5, 6]. Removal of extender is mandatory for success with techniques such as sperm sex-sorting, since the egg yolk causes opacity in the media surrounding the sperm cells, compromising the uniformity of the staining, interfering with laser excitation of the Hoechst-33,342 and with light transmission to the detectors [24, 25], and therefore resulting in a poor resolution of X- and Y- chromosome bearing sperm population [26].

Finally, Androcoll-Bear 80 showed the lowest recovery in this experiment, although improving all the sperm parameters assessed immediately after selection. This formulation (Androcoll-Bear 80) could be optimal in processes where a very high sperm quality, especially sperm motility, is required for success such as *IVF* [27]. Mitochondrial activity is known to be related with sperm motility and a relationship with fertility has been demonstrated [28, 29]. Cooling and freezing causes stress to spermatozoa and the mitochondria are the sperm structure most sensitive to this damage [30]; thus, the effect of selection bear spermatozoa using Androcoll-Bear on mitochondrial membrane potential will be considered in future studies.

The sperm population selected with Androcoll-Bear 80 had the highest values for area, length, width and elongation. This observation indicates that the sperm selected with the highest proportion of Androcoll were the biggest, the most hydrodynamic and the fastest of the whole sample. Therefore, according to [18], we can hypothesize that these selected samples could penetrate better and deeper into the female reproductive tract. Previous studies carried out in red deer showed that the lower the sperm head area in fresh samples, the greater the sperm cryoresistance [31]. These authors not only found a correlation between area and freezability but also between shape and freezability. This factor could be important since the distance from the side of the cell to the centre will determine the freezing velocity, being higher with elongation. In this context, the selection with Androcoll-Bear 80 in the present study resulted in the highest yield the highest elongation, potentially indicating better freezability.

Artificial insemination in this species is not yet developed because of many logistical and physiological issues that cannot be solved at present. In the absence of fertility trials, our further investigations will focus on the detection of predictive parameters of sperm quality such as: oxidative stress markers; 8-iso-PGF2alfa, 8-OHguanosine, 4-hydroxynonenaloxidation or the presence of active caspase 3 and 7 [32, 33]. Martin-Muñoz et al. [33] demonstrated assessing these markers to determine the differential effect of ROS during cryopreservation on stallion sperm and the importance of ROS homeostasis in the selection of ejaculates for freezing. The elimination of non-viable sperm from a sample could be important in ROS homeostasis since the dead sperm are known to have a negative effect on the viable subpopulation [9]. In addition, Roca et al. [34] showed that high proportions of dead sperm in raw semen samples before and during freezing induce significantly increased ROS generation and nuclear DNA fragmentation in frozen-thawed sperm. This is important since it is known that lipid peroxidation is related to “apoptosis-like” changes [35]. In this context, removing the dead sperm by selection with Androcoll-Bear may play an important role in improving cryosurvival rates not only after thawing, but also after incubation.

## Conclusions

In conclusion, sperm selection by Androcoll-Bear at proportions of 80% and 65% in brown bear sperm samples after thawing could be a useful tool. Androcoll-Bear 65 can improve the viability while giving good recoveries after thawing; meanwhile, Androcoll-Bear 80 resulted in a very high sperm quality but lower yield. In our opinion, Androcoll-Bear 65 would be optimal due to the higher difference in the recovery than Androcoll-Bear 80. Our findings pave the way for further research on application of sperm selection techniques to sperm banking in wild species and especially in brown bear.

## Additional files

Additional file 1: Data from the sperm quality parameters assessed. (PDF 39 kb)

Additional file 2: Data from sperm morphometry assessment. (PDF 1413 kb)

## Abbreviations

%; LIN: Linearity
%; PM: Progressive Motility
%; TM: Total Motility
(E) (L - W)/ (L + W): Elongation
A; μm^2^: Area
BGM-3: Bovine Gamete Medium
BSA: Bovine Serum Albumin
CASA: Computer Assisted Sperm Analysis
CM: Centrifugation Method
GRB: Genetic Resource Banks
IVF: in vitro *fertilization*
L; μm: Length
P; μm: Perimeter
SLC: Single Layer Centrifugation
STR: Straightness
TTF: TES-Tris-Fructose
VAP: Average Path Velocity
W; μm: Width
μm/s; VCL: Curvilinear Path Velocity
