# Supplementary material for: Improving sperm banking efficiency in endangered species through the use of a sperm selection method in brown bear (Ursus arctos) thawed sperm
Source: BMC Vet Res. 2017 Jun 26;13:200. doi: 10.1186/s12917-017-1124-2 (PMC5485503; doi:10.1186/s12917-017-1124-2)
Supplement: Supplementary file 1 — Data from the sperm quality parameters assessed. (PDF 39 kb) [file 12917_2017_1124_MOESM1_ESM.pdf]

Male	Time	Velocity	Gradiente	Yield
M_1	0	Control	Control	
M_1	0	v1	80	10,8
M_1	0	v1	65	45,9
M_1	0	v1	50	84,3
M_1	0	v2	80	15,1
M_1	0	v2	65	58,2
M_1	0	v2	50	73,4
M_2	0	Control	Control	
M_2	0	v1	80	42,4
M_2	0	v1	65	58,0
M_2	0	v1	50	74,3
M_2	0	v2	80	21,8
M_2	0	v2	65	61,4
M_2	0	v2	50	69,9
M_3	0	Control	Control	
M_3	0	v1	80	33,1
M_3	0	v1	65	51,4
M_3	0	v1	50	84,5
M_3	0	v2	80	19,8
M_3	0	v2	65	57,3
M_3	0	v2	50	85,2
M_4	0	Control	Control	
M_4	0	v1	80	11,8
M_4	0	v1	65	63,7
M_4	0	v1	50	85,8
M_4	0	v2	80	20,0
M_4	0	v2	65	70,5
M_4	0	v2	50	82,9
M_5	0	Control	Control	
M_5	0	v1	80	30,6
M_5	0	v1	65	41,5
M_5	0	v1	50	78,4
M_5	0	v2	80	15,3
M_5	0	v2	65	48,1
M_5	0	v2	50	69,3
M_6	0	Control	Control	
M_6	0	v1	80	6,4
M_6	0	v1	65	45,8
M_6	0	v1	50	84,2
M_6	0	v2	80	4,9
M_6	0	v2	65	35,4
M_6	0	v2	50	82,7
M_1	2	Control	Control	
M_1	2	v1	80	

M_1	2	v1	65
M_1	2	v1	50
M_1	2	v2	80
M_1	2	v2	65
M_1	2	v2	50
M_2	2	Control	Control
M_2	2	v1	80
M_2	2	v1	65
M_2	2	v1	50
M_2	2	v2	80
M_2	2	v2	65
M_2	2	v2	50
M_3	2	Control	Control
M_3	2	v1	80
M_3	2	v1	65
M_3	2	v1	50
M_3	2	v2	80
M_3	2	v2	65
M_3	2	v2	50
M_4	2	Control	Control
M_4	2	v1	80
M_4	2	v1	65
M_4	2	v1	50
M_4	2	v2	80
M_4	2	v2	65
M_4	2	v2	50
M_5	2	Control	Control
M_5	2	v1	80
M_5	2	v1	65
M_5	2	v1	50
M_5	2	v2	80
M_5	2	v2	65
M_5	2	v2	50
M_6	2	Control	Control
M_6	2	v1	80
M_6	2	v1	65
M_6	2	v1	50
M_6	2	v2	80
M_6	2	v2	65
M_6	2	v2	50

%Live intact Acro	%live not apop	%Live apop	TM	PM
84,35	59,3	14,74	75,21	39,41
94,59	76,01	16,02	88,56	49,13
92,6	78,77	13,26	78,55	44,10
86,14	63,85	10,91	71,15	33,22
96,04	79,61	15,16	87,98	43,25
93,83	82,6	9,87	83,67	37,91
86,62	64,65	11,63	77,94	38,27
86,92	42,5	29,23	82,18	39,50
95,87	77,99	17,49	89,55	63,96
90,79	72,52	16,86	85,40	51,59
88,45	60,15	19,08	80,49	48,15
95,71	78	16,01	92,25	52,46
90,16	73,19	15,84	85,25	44,47
86,45	59,17	17,66	85,93	46,30
66,2	31,92	38,57	55,62	35,57
95,14	59,54	35,91	90,03	60,42
88,92	54,88	36,36	83,30	51,80
79,05	39,77	33,45	80,79	45,32
95,45	66,51	29,61	89,80	72,63
86,41	59,61	31,5	87,12	59,79
74,47	43,08	31,42	63,77	38,69
74,11	47,3	22,79	67,41	34,35
86,74	82,16	8,58	81,03	63,84
85,17	80,15	7,64	61,64	30,09
83,34	66,69	10,22	54,35	28,33
86,78	77,2	8,26	81,17	62,60
83,97	77,16	8,34	54,31	30,46
81,94	67,19	9,14	57,01	35,03
74,24	44,18	29,94	63,69	36,78
95,83	85,6	10,47	80,37	51,91
92,17	81,08	11,04	73,94	40,60
88,62	66,1	14,55	57,88	37,22
96,64	88,9	7,67	80,37	51,91
91,7	76,67	14,35	73,94	40,60
86,82	63,39	15,95	57,88	37,22
66,53	55,17	9,79	66,02	36,78
90,46	83,59	9,41	76,19	65,65
80,11	74,67	7,65	56,58	43,28
69,59	53,28	9,06	42,04	30,01
86,66	82	9,13	78,87	58,58
80,53	75,09	6,79	46,63	32,44
67,39	53,79	8,41	45,79	28,59
37,39	19,69	10,16	36,99	17,15
61,53	42,8	15,2	52,51	34,47

47,62	31,34	13,22	44,67	23,23
41,67	21,13	11,15	26,97	14,83
48,77	43,78	13,57	54,43	33,98
52,92	31,1	15,21	28,81	16,67
51,8	25,54	13,9	34,19	18,06
64,67	31,02	38,01	47,59	16,17
82,34	58,44	26,96	85,24	49,60
80,79	59,94	20,83	72,43	45,54
78,48	53,54	18,98	52,80	35,05
83,93	58,49	27,46	86,14	44,50
81,44	64,15	16,75	80,73	44,79
80,3	52,05	17,26	73,17	40,90
66,35	27,68	41,99	56,92	33,11
89,39	62,23	27,13	84,22	59,06
83,53	59,73	27,28	82,35	59,59
73,44	35,8	32,48	59,77	31,87
90,94	72,52	20,46	87,59	51,12
82,41	65,22	20,81	77,53	49,02
76,83	44,04	25,65	73,88	43,53
61,91	35,03	25,37	32,14	14,49
82,49	64,12	17,3	82,10	62,66
67,37	57,6	15,17	50,58	29,81
74	45,1	15,44	32,38	17,41
86,96	47,52	30,11	79,51	57,75
77,53	51,99	17,39	57,23	31,44
72,18	42,53	14,96	31,05	15,42
41,78	28,59	16,42	32,73	16,36
59,45	37,35	15,96	65,39	42,55
51,06	25,35	20,52	55,40	40,06
48,07	23,75	16,26	30,60	16,68
59,14	34,92	19,26	65,82	49,44
45,15	23,74	20,41	49,86	31,98
45,69	17,7	17,34	32,69	22,97
63,33	47,96	12,81	28,43	12,96
86,66	74,32	12,99	77,04	65,19
75,72	70,5	6,83	51,46	36,48
64,77	44,83	9,87	27,82	17,39
78,65	76,56	8,48	78,98	58,14
75,34	68,11	6,66	36,15	24,26
62,29	45,38	8,89	23,17	12,83

VAP	LIN	PM SLOW	PM MEDIUM	PM RAPID
54,36	35,66	10,28	6,57	22,56
59,72	38,63	10,33	7,81	30,99
56,34	38,47	10,08	9,04	24,98
49,14	34,72	11,90	5,58	15,74
62,23	35,03	6,87	4,84	31,54
58,07	33,76	8,27	6,08	23,56
53,66	35,59	12,12	6,02	20,14
56,96	34,20	9,31	5,45	24,74
77,85	54,58	8,01	10,35	45,61
68,29	40,29	7,38	6,63	37,58
61,58	40,17	8,89	8,02	31,23
76,97	41,29	6,27	5,84	40,34
62,88	37,62	7,72	6,54	30,20
64,76	38,24	8,04	6,39	31,87
57,68	49,03	8,92	6,85	19,80
85,50	54,14	4,48	5,88	50,06
70,42	50,86	8,49	7,06	36,25
58,31	45,45	12,70	7,39	25,23
90,11	64,14	5,01	7,69	59,93
79,17	53,45	5,92	6,61	47,26
62,87	46,97	7,68	4,81	26,20
45,06	37,73	14,42	6,45	13,48
63,36	51,55	18,64	11,16	34,04
43,16	36,81	15,18	4,18	10,73
40,57	37,17	13,85	3,78	10,70
49,12	55,29	32,63	10,88	19,10
35,11	39,35	20,46	3,49	6,51
36,43	41,78	22,19	5,63	7,22
57,83	40,82	9,17	3,88	23,74
73,10	51,50	9,06	6,12	36,72
65,05	44,33	9,06	3,70	27,85
59,42	47,67	8,87	5,30	23,05
73,10	51,50	9,06	6,12	36,72
65,05	44,33	9,06	3,70	27,85
59,42	47,67	8,87	5,30	23,05
48,26	41,15	16,25	5,43	15,10
76,22	61,21	6,46	13,27	45,92
56,95	53,35	15,28	8,35	19,66
48,52	51,39	13,71	5,95	10,35
72,19	51,88	9,41	8,79	40,38
48,51	46,51	14,20	5,49	12,75
42,48	44,29	14,37	4,79	9,43
31,28	35,57	11,50	3,90	1,75
35,10	41,41	20,41	10,06	3,99

36,03	37,68	13,37	6,05	3,80
30,49	41,67	10,79	2,70	1,35
36,20	40,17	18,23	10,94	4,82
28,87	42,49	13,13	2,54	0,99
35,60	40,93	11,28	3,76	3,02
33,56	30,25	8,89	2,90	4,38
54,84	35,35	9,14	8,35	32,11
49,10	42,08	14,69	12,54	18,31
50,63	42,60	10,75	7,48	16,82
51,73	32,84	8,72	10,49	25,29
51,37	35,27	10,23	8,85	25,71
48,69	37,45	12,05	8,40	20,45
43,54	37,41	9,75	13,83	9,52
50,61	51,00	16,88	29,22	12,97
58,63	48,53	9,21	20,97	29,41
44,69	33,99	8,78	5,64	17,45
61,39	39,17	5,47	9,75	35,90
52,58	43,81	10,64	14,81	23,57
50,52	39,58	10,59	11,76	21,18
19,96	29,17	12,67	1,47	0,35
36,38	38,11	29,67	20,72	12,28
24,03	31,88	24,26	4,65	0,90
19,60	29,27	15,48	1,68	0,25
37,54	33,25	17,93	25,35	14,47
24,70	29,07	22,80	7,47	1,17
20,58	28,50	14,12	1,09	0,22
34,33	33,91	7,41	3,37	5,58
51,83	37,22	5,97	9,15	27,44
49,72	44,15	9,15	11,11	19,80
31,20	36,62	9,01	3,73	3,94
61,54	38,36	4,94	4,38	40,11
50,33	38,17	6,82	5,55	19,61
32,67	46,42	14,46	5,78	2,73
31,88	31,53	6,85	3,16	2,94
60,93	57,95	15,37	23,89	25,93
43,50	44,35	15,88	8,84	11,76
32,76	38,43	9,32	4,66	3,41
51,00	42,69	15,76	21,19	21,19
36,37	38,61	12,01	6,62	5,64
30,52	34,40	7,31	2,65	2,87
