# Supplementary material for: Improving sperm banking efficiency in endangered species through the use of a sperm selection method in brown bear (Ursus arctos) thawed sperm
Source: BMC Vet Res. 2017 Jun 26;13:200. doi: 10.1186/s12917-017-1124-2 (PMC5485503; doi:10.1186/s12917-017-1124-2)
Supplement: Supplementary file 2 — Data from sperm morphometry assessment. (PDF 1413 kb) [file 12917_2017_1124_MOESM2_ESM.pdf]

Velocidad	Gradiente	Area	EqDiameter	Perimeter	Length	Width
Control	Control	19,977	5,043	16,602	6,602	3,026
Control	Control	18,888	4,904	15,620	5,881	3,211
Control	Control	26,523	5,811	18,703	6,941	3,821
Control	Control	22,288	5,327	17,177	6,218	3,585
Control	Control	18,791	4,891	15,692	5,870	3,201
Control	Control	25,713	5,722	18,469	6,923	3,714
Control	Control	21,163	5,191	16,922	6,510	3,251
Control	Control	20,098	5,059	16,246	6,208	3,237
Control	Control	19,844	5,027	16,012	5,786	3,430
Control	Control	20,691	5,133	16,627	6,453	3,206
Control	Control	19,941	5,039	16,124	5,995	3,327
Control	Control	19,735	5,013	16,098	6,137	3,216
Control	Control	18,198	4,814	15,437	5,867	3,102
Control	Control	19,505	4,983	15,915	6,017	3,242
Control	Control	18,791	4,891	15,829	6,164	3,049
Control	Control	21,272	5,204	16,749	6,032	3,526
Control	Control	21,574	5,241	17,177	6,699	3,220
Control	Control	20,183	5,069	16,505	6,428	3,140
Control	Control	17,460	4,715	15,102	5,645	3,093
Control	Control	19,554	4,990	15,915	6,018	3,249
Control	Control	19,614	4,997	16,037	6,128	3,201
Control	Control	19,747	5,014	16,048	6,034	3,273
Control	Control	18,319	4,830	15,447	5,871	3,120
Control	Control	18,561	4,861	15,666	5,870	3,162
Control	Control	19,808	5,022	16,073	6,199	3,195
Control	Control	19,300	4,957	15,865	6,004	3,215
Control	Control	21,018	5,173	16,612	6,403	3,282
Control	Control	20,328	5,087	16,246	6,018	3,378
Control	Control	20,437	5,101	16,663	6,618	3,088
Control	Control	21,417	5,222	16,872	6,490	3,300
Control	Control	15,210	4,401	14,110	5,526	2,752
Control	Control	19,493	4,982	15,829	5,971	3,265
Control	Control	19,433	4,974	15,961	5,993	3,243
Control	Control	20,691	5,133	16,653	6,413	3,226
Control	Control	21,780	5,266	16,836	6,378	3,415
Control	Control	20,449	5,103	16,404	6,111	3,346
Control	Control	20,074	5,056	16,332	6,210	3,232
Control	Control	19,336	4,962	15,656	5,748	3,364
Control	Control	21,090	5,182	16,688	6,356	3,318
Control	Control	21,526	5,235	16,688	6,378	3,375
Control	Control	20,534	5,113	16,455	6,262	3,279
Control	Control	18,150	4,807	15,325	5,818	3,119
Control	Control	20,933	5,163	16,429	6,034	3,469
Control	Control	21,901	5,281	17,339	6,760	3,240
Control	Control	20,534	5,113	16,332	6,120	3,355
Control	Control	19,481	4,980	15,865	5,845	3,333
Control	Control	19,251	4,951	15,854	5,911	3,257
Control	Control	20,376	5,094	16,271	5,982	3,406
Control	Control	20,147	5,065	16,170	6,124	3,290
Control	Control	21,248	5,201	16,638	6,332	3,356
Control	Control	18,332	4,831	15,351	5,636	3,252

Control	Control	26,717	5,832	18,591	7,014	3,809
Control	Control	21,405	5,220	16,516	6,275	3,411
Control	Control	18,755	4,887	15,681	5,967	3,143
Control	Control	19,614	4,997	15,890	6,134	3,198
Control	Control	20,159	5,066	15,987	5,993	3,364
Control	Control	20,098	5,059	16,073	6,030	3,333
Control	Control	20,933	5,163	16,541	6,183	3,385
Control	Control	28,205	5,993	19,181	7,075	3,987
Control	Control	21,018	5,173	16,490	6,063	3,467
Control	Control	22,361	5,336	17,156	6,404	3,492
Control	Control	21,102	5,183	16,577	6,140	3,437
Control	Control	21,623	5,247	16,699	6,162	3,509
Control	Control	21,853	5,275	16,872	6,264	3,489
Control	Control	21,248	5,201	16,688	6,304	3,371
Control	Control	18,949	4,912	16,235	5,807	3,263
Control	Control	21,199	5,195	16,577	6,227	3,404
Control	Control	21,030	5,175	16,566	6,120	3,436
Control	Control	21,974	5,289	17,044	6,483	3,390
Control	Control	20,582	5,119	16,480	6,217	3,310
Control	Control	22,361	5,336	17,202	6,728	3,323
Control	Control	22,300	5,329	16,983	6,428	3,469
Control	Control	21,199	5,195	16,577	6,227	3,404
Control	Control	21,030	5,175	16,566	6,120	3,436
Control	Control	21,974	5,289	17,044	6,483	3,390
Control	Control	20,582	5,119	16,480	6,217	3,310
Control	Control	22,361	5,336	17,202	6,728	3,323
Control	Control	22,300	5,329	16,983	6,428	3,469
Control	Control	20,582	5,119	16,307	6,164	3,339
Control	Control	19,687	5,007	15,976	5,994	3,284
Control	Control	19,215	4,946	15,717	5,927	3,242
Control	Control	20,183	5,069	16,318	6,061	3,330
Control	Control	20,921	5,161	16,490	6,096	3,432
Control	Control	21,490	5,231	16,688	6,410	3,353
Control	Control	21,344	5,213	16,663	6,353	3,360
Control	Control	20,921	5,161	16,160	6,064	3,450
Control	Control	20,679	5,131	16,393	6,247	3,310
Control	Control	19,626	4,999	16,012	6,034	3,253
Control	Control	20,485	5,107	16,663	6,428	3,187
Control	Control	21,828	5,272	16,699	6,278	3,477
Control	Control	21,030	5,175	16,480	6,032	3,487
Control	Control	20,933	5,163	16,429	6,134	3,413
Control	Control	22,446	5,346	17,131	6,510	3,448
Control	Control	22,421	5,343	17,080	6,304	3,557
Control	Control	21,102	5,183	16,612	6,170	3,420
Control	Control	20,594	5,121	16,256	5,993	3,436
Control	Control	21,187	5,194	16,638	6,169	3,435
Control	Control	19,457	4,977	15,778	5,918	3,288
Control	Control	22,530	5,356	17,314	6,634	3,396
Control	Control	23,740	5,498	17,940	6,757	3,513
Control	Control	22,083	5,302	16,933	6,387	3,457
Control	Control	19,372	4,966	15,778	5,871	3,299
Control	Control	21,441	5,225	16,811	6,367	3,367

Control	Control	21,344	5,213	16,785	6,259	3,410
Control	Control	19,275	4,954	16,109	6,189	3,114
Control	Control	19,844	5,027	15,865	5,889	3,370
Control	Control	23,317	5,449	17,645	6,769	3,445
Control	Control	22,034	5,297	17,091	6,438	3,422
Control	Control	20,159	5,066	16,271	6,238	3,232
Control	Control	21,066	5,179	16,612	6,341	3,322
Control	Control	21,695	5,256	16,836	6,483	3,347
Control	Control	21,623	5,247	16,688	6,457	3,349
Control	Control	22,095	5,304	16,897	6,363	3,472
Control	Control	18,961	4,913	15,692	5,927	3,199
Control	Control	21,078	5,181	16,602	6,222	3,388
Control	Control	19,191	4,943	15,742	5,877	3,265
Control	Control	22,240	5,321	16,846	6,197	3,589
Control	Control	19,529	4,987	16,124	6,148	3,177
Control	Control	19,493	4,982	15,926	5,927	3,289
Control	Control	18,888	4,904	15,803	5,967	3,165
Control	Control	16,867	4,634	14,822	5,636	2,993
Control	Control	20,461	5,104	16,332	6,157	3,323
Control	Control	21,006	5,172	16,688	6,237	3,368
Control	Control	20,812	5,148	16,541	6,301	3,303
Control	Control	20,038	5,051	16,195	6,238	3,212
Control	Control	29,016	6,078	19,857	6,792	4,272
Control	Control	20,013	5,048	16,246	6,125	3,268
Control	Control	21,006	5,172	16,688	6,237	3,368
Control	Control	20,812	5,148	16,541	6,301	3,303
Control	Control	20,038	5,051	16,195	6,238	3,212
Control	Control	20,013	5,048	16,246	6,125	3,268
Control	Control	21,187	5,194	16,627	6,303	3,361
Control	Control	19,880	5,031	15,987	5,772	3,444
Control	Control	21,018	5,173	16,587	6,162	3,411
Control	Control	20,691	5,133	16,612	6,618	3,126
Control	Control	21,260	5,203	16,699	6,240	3,407
Control	Control	18,791	4,891	15,890	6,277	2,993
Control	Control	20,522	5,112	16,368	6,199	3,310
Control	Control	19,300	4,957	15,656	5,786	3,336
Control	Control	20,800	5,146	16,393	6,057	3,434
Control	Control	16,214	4,544	14,527	5,418	2,993
Control	Control	20,618	5,124	16,551	6,278	3,284
Control	Control	18,465	4,849	15,559	5,889	3,135
Control	Control	20,280	5,081	16,404	6,111	3,318
Control	Control	18,900	4,906	15,605	5,748	3,288
Control	Control	20,122	5,062	16,195	6,140	3,277
Control	Control	20,897	5,158	16,638	6,218	3,361
Control	Control	19,880	5,031	16,160	6,171	3,221
Control	Control	21,018	5,173	16,638	6,197	3,392
Control	Control	21,429	5,223	16,627	6,257	3,425
V1	50	18,574	4,863	15,926	6,157	3,017
V1	50	20,340	5,089	16,663	6,427	3,165
V1	50	17,642	4,739	15,214	5,744	3,071
V1	50	18,707	4,880	15,839	6,222	3,006
V1	50	19,675	5,005	16,109	6,034	3,261

V1	50	20,522	5,112	16,587	6,170	3,326
V1	50	19,771	5,017	15,951	6,062	3,262
V1	50	18,779	4,890	15,778	5,845	3,213
V1	50	19,844	5,027	16,160	6,277	3,161
V1	50	20,328	5,087	16,541	6,482	3,136
V1	50	18,791	4,891	15,717	5,994	3,135
V1	50	19,711	5,010	15,926	5,826	3,383
V1	50	19,663	5,004	16,210	6,182	3,180
V1	50	16,262	4,550	14,440	5,473	2,971
V1	50	18,186	4,812	15,509	5,965	3,049
V1	50	19,142	4,937	16,063	6,148	3,114
V1	50	20,897	5,158	16,602	6,218	3,361
V1	50	20,074	5,056	16,195	6,096	3,293
V1	50	20,582	5,119	16,516	6,063	3,395
V1	50	18,259	4,822	15,361	5,936	3,076
V1	50	21,163	5,191	16,592	6,021	3,515
V1	50	18,828	4,896	15,570	5,718	3,293
V1	50	18,634	4,871	15,605	5,686	3,277
V1	50	17,581	4,731	15,091	5,786	3,039
V1	50	19,445	4,976	16,012	5,977	3,253
V1	50	16,819	4,628	14,674	5,636	2,984
V1	50	19,312	4,959	15,656	5,815	3,321
V1	50	20,086	5,057	16,134	6,164	3,259
V1	50	19,784	5,019	16,393	6,317	3,132
V1	50	20,497	5,109	16,124	6,071	3,376
V1	50	18,997	4,918	15,742	6,057	3,136
V1	50	20,280	5,081	16,307	6,118	3,315
V1	50	19,638	5,000	16,419	6,406	3,066
V1	50	18,949	4,912	15,768	6,032	3,141
V1	50	20,945	5,164	16,638	6,556	3,195
V1	50	19,856	5,028	16,185	6,157	3,225
V1	50	21,998	5,292	17,070	6,469	3,401
V1	50	23,002	5,412	17,177	5,922	3,884
V1	50	21,357	5,215	16,541	6,266	3,409
V1	50	26,281	5,785	19,145	7,148	3,677
V1	50	20,461	5,104	16,846	6,227	3,286
V1	50	21,901	5,281	17,009	6,454	3,394
V1	50	20,739	5,139	16,749	6,409	3,236
V1	50	20,255	5,078	16,307	6,257	3,237
V1	50	21,357	5,215	16,541	6,266	3,409
V1	50	20,461	5,104	16,846	6,227	3,286
V1	50	21,901	5,281	17,009	6,454	3,394
V1	50	20,739	5,139	16,749	6,409	3,236
V1	50	20,255	5,078	16,307	6,257	3,237
V1	50	19,094	4,931	15,951	5,934	3,218
V1	50	22,639	5,369	17,314	6,032	3,753
V1	50	19,070	4,927	15,768	6,096	3,128
V1	50	18,634	4,871	15,692	5,899	3,159
V1	50	19,324	4,960	16,037	6,167	3,133
V1	50	19,384	4,968	16,073	6,111	3,172
V1	50	20,848	5,152	16,541	6,211	3,357
V1	50	18,900	4,906	15,778	5,984	3,158

V1	50	18,223	4,817	15,422	5,737	3,177
V1	50	19,130	4,935	15,890	5,967	3,206
V1	50	20,497	5,109	16,551	6,297	3,255
V1	50	19,554	4,990	15,865	5,971	3,275
V1	50	17,218	4,682	14,919	5,607	3,071
V1	50	19,445	4,976	16,246	6,358	3,058
V1	50	19,445	4,976	16,246	6,358	3,058
V1	50	21,042	5,176	16,846	6,713	3,134
V1	50	22,518	5,355	17,228	6,602	3,411
V1	50	19,735	5,013	16,048	6,096	3,237
V1	50	21,066	5,179	16,602	6,300	3,344
V1	50	19,094	4,931	16,134	5,852	3,263
V1	50	21,720	5,259	16,983	6,483	3,350
V1	50	21,357	5,215	16,577	6,406	3,334
V1	50	15,645	4,463	14,206	5,480	2,855
V1	50	21,030	5,175	16,627	6,221	3,380
V1	50	19,421	4,973	16,037	6,147	3,159
V1	50	20,062	5,054	16,221	6,083	3,298
V1	50	20,691	5,133	16,735	6,218	3,328
V1	50	19,554	4,990	15,865	5,953	3,285
V1	50	21,889	5,279	16,861	6,211	3,524
V1	50	22,990	5,410	17,192	6,310	3,643
V1	50	19,723	5,011	16,048	5,871	3,359
V1	50	21,042	5,176	16,836	6,170	3,410
V1	50	17,714	4,749	15,264	5,882	3,012
V1	50	20,715	5,136	16,602	6,333	3,271
V1	50	24,720	5,610	18,281	6,643	3,721
V1	50	20,715	5,136	16,602	6,333	3,271
V1	50	20,691	5,133	16,332	6,064	3,412
V1	50	22,252	5,323	17,411	6,317	3,523
V1	50	21,272	5,204	16,663	6,218	3,421
V1	50	20,413	5,098	16,480	6,222	3,281
V1	50	20,425	5,100	16,307	6,111	3,342
V1	50	19,917	5,036	16,185	6,094	3,268
V1	50	21,381	5,218	16,577	6,237	3,428
V1	50	20,413	5,098	16,160	6,134	3,328
V1	50	18,477	4,850	15,631	5,826	3,172
V1	50	21,841	5,273	16,749	6,167	3,541
V1	50	19,336	4,962	15,865	5,899	3,278
V1	50	25,035	5,646	18,418	6,454	3,879
V1	50	20,207	5,072	16,307	6,210	3,254
V1	50	20,280	5,081	16,149	6,031	3,363
V1	50	21,841	5,273	16,749	6,167	3,541
V1	50	19,336	4,962	15,865	5,899	3,278
V1	50	20,207	5,072	16,307	6,210	3,254
V1	50	20,280	5,081	16,149	6,031	3,363
V1	50	22,385	5,339	17,167	6,483	3,453
V1	50	19,820	5,023	16,098	6,034	3,285
V1	50	20,461	5,104	16,404	6,218	3,291
V1	50	19,239	4,949	15,829	6,162	3,122
V1	50	21,115	5,185	16,455	6,117	3,452
V1	50	19,699	5,008	16,393	6,400	3,078

V1	50	26,426	5,801	18,408	6,864	3,850
V1	50	21,296	5,207	16,541	6,264	3,400
V1	50	21,998	5,292	16,933	6,260	3,514
V1	50	21,490	5,231	17,238	6,700	3,207
V1	50	20,510	5,110	16,307	6,183	3,317
V1	50	20,268	5,080	16,073	6,021	3,366
V1	50	21,695	5,256	17,116	6,368	3,407
V1	50	19,917	5,036	16,012	5,922	3,363
V1	50	21,490	5,231	17,030	6,754	3,182
V1	50	21,635	5,248	16,958	6,618	3,269
V1	50	21,647	5,250	16,724	6,169	3,509
V1	50	20,618	5,124	16,297	6,264	3,292
V1	50	21,465	5,228	16,969	6,324	3,394
V1	50	19,868	5,030	16,063	5,937	3,347
V1	50	21,695	5,256	17,034	6,563	3,306
V1	50	21,889	5,279	17,019	6,575	3,329
V1	50	19,300	4,957	15,961	6,021	3,206
V1	50	19,324	4,960	15,656	5,927	3,260
V1	50	20,582	5,119	16,221	5,956	3,456
V1	50	21,998	5,292	17,167	6,553	3,357
V1	50	21,720	5,259	17,228	6,381	3,404
V1	50	22,675	5,373	17,314	6,363	3,564
V1	50	20,280	5,081	16,343	6,266	3,237
V1	50	20,159	5,066	16,109	5,927	3,401
V1	50	20,219	5,074	16,246	6,156	3,284
V1	50	21,248	5,201	16,882	6,381	3,330
V1	50	19,650	5,002	16,073	5,927	3,315
V1	50	20,558	5,116	16,368	6,237	3,296
V1	50	21,320	5,210	16,811	6,457	3,302
V1	50	19,759	5,016	15,803	5,772	3,423
V1	50	21,574	5,241	16,958	6,459	3,340
V1	50	21,357	5,215	16,638	6,275	3,403
V1	50	18,404	4,841	15,483	5,683	3,239
V1	50	21,381	5,218	16,922	6,483	3,298
V1	50	21,199	5,195	17,044	6,623	3,201
V1	50	21,090	5,182	16,749	6,428	3,281
V1	50	19,033	4,923	15,951	6,222	3,059
V1	50	20,643	5,127	16,195	6,072	3,400
V1	50	20,836	5,151	16,577	6,124	3,402
V1	50	21,623	5,247	16,749	6,364	3,398
V1	50	22,869	5,396	17,472	6,656	3,436
V1	50	19,663	5,004	15,987	5,977	3,290
V1	50	19,687	5,007	16,012	6,317	3,116
V1	50	20,183	5,069	16,185	6,003	3,362
V1	50	20,110	5,060	16,195	5,977	3,365
V1	50	19,009	4,920	15,768	5,841	3,254
V1	50	22,409	5,342	17,289	6,509	3,443
V1	50	20,522	5,112	16,307	6,120	3,353
V1	50	22,639	5,369	17,681	6,895	3,283
V1	50	19,215	4,946	15,768	5,967	3,220
V1	50	20,994	5,170	16,541	6,373	3,294
V1	50	22,518	5,355	17,080	6,400	3,519

V1	50	22,712	5,377	17,202	6,428	3,533
V1	50	20,933	5,163	16,577	6,509	3,216
V1	50	21,562	5,240	16,724	6,246	3,452
V1	50	20,909	5,160	16,749	6,531	3,201
V1	50	17,727	4,751	15,325	6,011	2,949
V1	50	20,969	5,167	16,749	6,463	3,245
V1	50	23,631	5,485	17,695	6,769	3,491
V1	50	21,284	5,206	16,638	6,111	3,483
V1	50	19,953	5,040	16,134	6,200	3,218
V1	50	22,542	5,357	17,335	6,613	3,409
V1	50	20,219	5,074	16,343	6,237	3,242
V1	50	22,337	5,333	17,411	6,787	3,291
V1	50	20,812	5,148	16,343	6,127	3,397
V1	50	21,163	5,191	16,674	6,218	3,403
V1	50	20,921	5,161	16,907	6,386	3,276
V1	50	19,433	4,974	15,961	6,062	3,206
V1	50	21,744	5,262	16,969	6,324	3,438
V1	50	22,325	5,331	17,019	6,417	3,479
V1	50	19,953	5,040	16,109	5,977	3,338
V1	50	22,349	5,334	17,533	6,728	3,322
V1	50	19,578	4,993	16,109	6,111	3,204
V1	50	21,756	5,263	16,836	6,373	3,414
V1	50	15,657	4,465	14,293	5,472	2,862
V1	50	21,962	5,288	17,181	6,509	3,374
V1	50	22,663	5,372	17,375	6,514	3,479
V1	65	20,268	5,080	16,246	6,140	3,301
V1	65	19,215	4,946	15,951	6,148	3,126
V1	65	18,876	4,902	15,681	5,882	3,209
V1	65	20,739	5,139	16,368	6,264	3,311
V1	65	18,465	4,849	15,753	6,059	3,048
V1	65	27,419	5,909	18,972	7,101	3,861
V1	65	22,167	5,313	17,055	6,210	3,569
V1	65	20,038	5,051	16,160	6,127	3,270
V1	65	19,747	5,014	16,185	6,157	3,207
V1	65	21,550	5,238	17,167	6,580	3,275
V1	65	23,280	5,444	17,584	6,495	3,584
V1	65	20,316	5,086	16,282	6,111	3,324
V1	65	23,813	5,506	17,645	6,584	3,617
V1	65	19,517	4,985	15,890	5,922	3,296
V1	65	19,070	4,927	15,778	6,011	3,172
V1	65	21,756	5,263	16,907	6,324	3,440
V1	65	21,804	5,269	16,872	6,278	3,473
V1	65	20,606	5,122	16,429	6,171	3,339
V1	65	18,041	4,793	15,605	5,899	3,058
V1	65	20,062	5,054	16,073	5,928	3,384
V1	65	20,231	5,075	16,282	6,164	3,282
V1	65	21,236	5,200	17,106	6,787	3,129
V1	65	18,912	4,907	15,631	5,918	3,196
V1	65	20,292	5,083	16,160	6,125	3,313
V1	65	19,929	5,037	16,282	6,062	3,287
V1	65	20,268	5,080	16,185	6,072	3,338
V1	65	18,900	4,906	15,717	5,845	3,234

V1	65	19,275	4,954	15,829	5,904	3,265
V1	65	20,473	5,106	16,541	6,336	3,231
V1	65	17,702	4,748	15,178	5,786	3,059
V1	65	20,147	5,065	16,185	6,087	3,310
V1	65	19,808	5,022	16,307	6,156	3,217
V1	65	18,513	4,855	15,544	5,852	3,164
V1	65	20,086	5,057	16,195	6,111	3,287
V1	65	21,078	5,181	16,699	6,279	3,357
V1	65	20,643	5,127	16,307	6,127	3,369
V1	65	19,832	5,025	16,073	6,062	3,271
V1	65	20,485	5,107	16,419	6,222	3,293
V1	65	19,941	5,039	16,073	6,059	3,291
V1	65	19,856	5,028	16,282	6,034	3,291
V1	65	18,719	4,882	15,473	5,772	3,243
V1	65	21,695	5,256	16,846	6,297	3,445
V1	65	19,554	4,990	16,185	6,222	3,142
V1	65	20,885	5,157	16,429	6,096	3,426
V1	65	19,747	5,014	16,037	6,127	3,223
V1	65	20,667	5,130	16,419	6,217	3,324
V1	65	21,236	5,200	16,760	6,472	3,281
V1	65	19,142	4,937	15,620	5,956	3,214
V1	65	25,398	5,687	18,677	6,835	3,716
V1	65	19,856	5,028	16,109	5,899	3,366
V1	65	21,599	5,244	16,983	6,649	3,248
V1	65	17,424	4,710	15,005	5,645	3,086
V1	65	20,534	5,113	16,836	6,364	3,227
V1	65	21,163	5,191	16,551	6,171	3,429
V1	65	21,006	5,172	16,480	6,030	3,483
V1	65	21,163	5,191	16,724	6,189	3,419
V1	65	19,203	4,945	15,839	5,743	3,344
V1	65	20,268	5,080	16,318	6,140	3,301
V1	65	19,941	5,039	16,160	6,087	3,276
V1	65	18,368	4,836	15,498	5,732	3,204
V1	65	19,505	4,983	15,865	5,971	3,267
V1	65	19,602	4,996	16,037	6,208	3,158
V1	65	20,606	5,122	16,429	6,227	3,309
V1	65	18,440	4,846	15,681	6,087	3,030
V1	65	27,830	5,953	19,674	6,699	4,154
V1	65	21,018	5,173	16,714	6,456	3,255
V1	65	20,969	5,167	16,490	6,217	3,373
V1	65	18,440	4,846	15,681	6,087	3,030
V1	65	21,018	5,173	16,714	6,456	3,255
V1	65	20,969	5,167	16,490	6,217	3,373
V1	65	19,687	5,007	16,124	6,167	3,192
V1	65	20,389	5,095	16,440	6,310	3,231
V1	65	21,562	5,240	16,749	6,337	3,403
V1	65	20,437	5,101	16,541	6,456	3,165
V1	65	20,909	5,160	16,455	6,157	3,396
V1	65	19,300	4,957	15,803	5,927	3,256
V1	65	20,534	5,113	16,282	5,977	3,435
V1	65	21,683	5,254	17,044	6,580	3,295
V1	65	20,534	5,113	16,551	6,189	3,318

V1	65	20,364	5,092	16,551	6,510	3,128
V1	65	20,691	5,133	16,480	6,157	3,361
V1	65	21,768	5,265	16,836	6,363	3,421
V1	65	21,151	5,189	16,785	6,363	3,324
V1	65	20,401	5,097	16,358	6,057	3,368
V1	65	20,994	5,170	16,602	6,211	3,380
V1	65	19,590	4,994	15,839	5,815	3,369
V1	65	21,090	5,182	16,541	6,184	3,411
V1	65	19,844	5,027	15,987	5,899	3,364
V1	65	20,328	5,087	16,282	6,023	3,375
V1	65	20,062	5,054	16,343	6,257	3,206
V1	65	20,401	5,097	16,627	6,364	3,206
V1	65	19,421	4,973	16,012	6,003	3,235
V1	65	21,586	5,243	17,055	6,622	3,260
V1	65	20,268	5,080	16,160	5,977	3,391
V1	65	18,719	4,882	15,620	5,977	3,132
V1	65	19,336	4,962	15,803	5,849	3,306
V1	65	21,526	5,235	16,958	6,463	3,331
V1	65	18,755	4,887	15,717	5,880	3,189
V1	65	21,018	5,173	16,551	6,125	3,432
V1	65	21,066	5,179	16,480	5,995	3,514
V1	65	21,853	5,275	16,811	6,218	3,514
V1	65	19,505	4,983	15,926	6,164	3,164
V1	65	19,058	4,926	15,926	5,977	3,189
V1	65	19,808	5,022	16,149	6,032	3,284
V1	65	19,941	5,039	16,332	6,373	3,129
V1	65	21,913	5,282	16,872	6,510	3,366
V1	65	20,207	5,072	16,526	6,363	3,175
V1	65	22,131	5,308	16,969	6,604	3,351
V1	65	18,646	4,872	15,534	5,787	3,222
V1	65	21,465	5,228	16,907	6,603	3,251
V1	65	22,337	5,333	17,044	6,417	3,481
V1	65	19,965	5,042	16,379	6,340	3,149
V1	65	19,348	4,963	15,717	5,880	3,291
V1	65	20,352	5,091	16,526	6,472	3,144
V1	65	20,594	5,121	16,541	6,318	3,259
V1	65	19,675	5,005	16,271	6,318	3,114
V1	65	20,776	5,143	16,455	6,168	3,368
V1	65	20,159	5,066	16,221	6,164	3,270
V1	65	20,606	5,122	16,688	6,490	3,175
V1	65	19,166	4,940	15,803	6,094	3,145
V1	65	18,114	4,802	15,534	5,967	3,035
V1	65	20,255	5,078	16,368	6,218	3,258
V1	65	18,307	4,828	15,483	5,927	3,089
V1	65	19,336	4,962	15,951	5,940	3,255
V1	65	20,389	5,095	16,490	6,266	3,254
V1	65	21,102	5,183	16,455	6,264	3,369
V1	65	19,965	5,042	16,160	6,096	3,275
V1	65	17,848	4,767	15,264	5,724	3,118
V1	65	15,597	4,456	14,171	5,426	2,874
V1	65	19,336	4,962	15,865	5,962	3,243
V1	65	21,127	5,186	16,846	6,430	3,286

V1	65	23,450	5,464	17,314	5,955	3,938
V1	65	19,554	4,990	16,160	6,218	3,145
V1	65	18,888	4,904	15,595	5,504	3,432
V1	65	21,066	5,179	16,638	6,228	3,382
V1	65	21,066	5,179	16,638	6,228	3,382
V1	65	19,880	5,031	16,455	6,454	3,080
V1	65	19,771	5,017	15,951	6,030	3,279
V1	65	19,348	4,963	15,803	5,967	3,242
V1	65	18,779	4,890	15,447	5,731	3,277
V1	65	20,764	5,142	16,516	6,278	3,307
V1	65	19,324	4,960	15,829	5,871	3,291
V1	65	20,437	5,101	16,516	6,427	3,180
V1	65	21,078	5,181	16,627	6,481	3,252
V1	65	15,730	4,475	14,685	5,081	3,096
V1	65	20,437	5,101	16,516	6,427	3,180
V1	65	21,078	5,181	16,627	6,481	3,252
V1	65	18,622	4,869	15,620	5,841	3,188
V1	65	18,477	4,850	15,473	5,827	3,171
V1	65	22,216	5,318	17,152	6,275	3,541
V1	65	21,066	5,179	16,516	6,125	3,440
V1	80	15,996	4,513	14,023	5,105	3,329
V1	80	15,839	4,491	14,329	5,327	3,089
V2	80	16,867	4,634	14,674	5,305	3,241
V2	80	15,246	4,406	14,120	5,361	2,898
V1	80	16,916	4,641	14,588	5,390	3,198
V1	80	15,936	4,504	14,522	5,296	3,009
V2	80	16,734	4,616	14,857	5,327	3,141
V1	80	16,045	4,520	14,379	5,336	3,007
V1	80	17,146	4,672	14,710	5,339	3,211
V1	80	16,396	4,569	14,466	5,380	3,048
V1	80	17,400	4,707	14,980	5,396	3,225
V1	80	16,033	4,518	14,390	5,401	2,969
V1	80	16,783	4,623	14,685	5,401	3,107
V2	80	16,662	4,606	15,188	5,419	3,074
V1	80	15,391	4,427	14,145	5,429	2,835
V1	80	18,937	4,910	15,412	5,438	3,482
V1	80	16,662	4,606	14,527	5,439	3,064
V1	80	17,581	4,731	14,944	5,445	3,229
V1	80	16,383	4,567	14,552	5,466	2,997
V1	80	16,867	4,634	14,700	5,471	3,083
V1	80	15,960	4,508	14,379	5,671	2,917
V1	80	15,960	4,508	14,379	5,771	2,917
V1	80	17,593	4,733	15,020	5,473	3,215
V1	80	16,408	4,571	14,440	5,473	2,998
V1	80	16,238	4,547	14,893	5,480	2,963
V2	80	15,161	4,394	14,131	5,486	2,764
V1	80	16,504	4,584	14,588	5,501	3,000
V1	80	16,311	4,557	14,466	5,504	2,963
V1	80	17,400	4,707	14,908	5,526	3,149
V2	80	17,521	4,723	14,980	5,532	3,167
V1	80	17,436	4,712	14,908	5,541	3,147
V1	80	17,533	4,725	14,944	5,544	3,163

V2	80	18,223	4,817	15,275	5,552	3,282
V1	80	17,896	4,773	15,249	5,552	3,224
V2	80	18,356	4,834	15,372	5,552	3,306
V2	80	17,025	4,656	14,710	5,566	3,058
V1	80	17,569	4,730	15,117	5,572	3,153
V2	80	16,940	4,644	14,822	5,573	3,040
V2	80	18,670	4,876	15,458	5,579	3,346
V1	80	17,327	4,697	15,005	5,579	3,106
V2	80	17,823	4,764	15,066	5,580	3,194
V1	80	17,448	4,713	15,005	5,580	3,127
V1	80	18,150	4,807	15,372	5,580	3,253
V2	80	17,460	4,715	14,980	5,580	3,129
V2	80	18,029	4,791	15,249	5,581	3,230
V1	80	17,436	4,712	14,969	5,581	3,124
V2	80	17,908	4,775	15,310	5,582	3,208
V1	80	18,247	4,820	15,239	5,598	3,260
V2	80	16,577	4,594	14,761	5,607	2,957
V1	80	16,783	4,623	14,685	5,607	2,993
V1	80	18,574	4,863	15,397	5,609	3,312
V2	80	18,428	4,844	15,458	5,609	3,286
V1	80	18,416	4,842	15,275	5,609	3,284
V1	80	16,347	4,562	14,735	5,609	2,915
V2	80	19,191	4,943	15,753	5,609	3,422
V1	80	17,969	4,783	15,188	5,609	3,204
V1	80	17,702	4,748	15,005	5,609	3,156
V1	80	17,279	4,690	14,822	5,619	3,075
V2	80	18,477	4,850	15,351	5,622	3,287
V1	80	17,412	4,708	14,944	5,622	3,097
V1	80	17,739	4,752	14,944	5,626	3,153
V2	80	17,835	4,765	15,005	5,626	3,170
V1	80	17,097	4,666	15,005	5,629	3,037
V2	80	18,852	4,899	15,509	5,629	3,349
V1	80	18,537	4,858	15,325	5,634	3,290
V2	80	17,799	4,761	15,203	5,634	3,159
V2	80	18,029	4,791	15,239	5,636	3,199
V1	80	17,472	4,717	14,944	5,636	3,100
V1	80	18,816	4,895	15,447	5,637	3,338
V2	80	18,344	4,833	15,325	5,637	3,254
V1	80	18,828	4,896	15,631	5,637	3,340
V2	80	18,198	4,814	15,372	5,637	3,228
V2	80	17,521	4,723	15,102	5,637	3,108
V2	80	17,872	4,770	15,127	5,637	3,170
V2	80	18,840	4,898	15,473	5,645	3,337
V1	80	17,097	4,666	14,919	5,658	3,022
V2	80	18,477	4,850	15,397	5,658	3,265
V1	80	18,005	4,788	15,264	5,661	3,181
V1	80	18,307	4,828	15,300	5,661	3,234
V1	80	17,896	4,773	15,142	5,661	3,161
V1	80	17,085	4,664	14,883	5,661	3,018
V1	80	18,150	4,807	15,239	5,672	3,200
V1	80	17,775	4,757	15,066	5,672	3,134
V1	80	17,775	4,757	15,117	5,673	3,133

V1	80	18,114	4,802	15,336	5,673	3,193
V1	80	18,029	4,791	15,310	5,673	3,178
V1	80	18,682	4,877	15,473	5,676	3,291
V1	80	18,707	4,880	15,361	5,676	3,295
V1	80	17,944	4,780	15,091	5,676	3,161
V1	80	17,714	4,749	15,056	5,677	3,120
V1	80	17,763	4,756	15,005	5,683	3,126
V1	80	18,610	4,868	15,422	5,683	3,275
V2	80	18,283	4,825	15,300	5,683	3,217
V2	80	19,650	5,002	15,900	5,686	3,456
V1	80	18,380	4,838	15,361	5,686	3,232
V1	80	17,872	4,770	15,285	5,686	3,143
V2	80	19,106	4,932	15,559	5,691	3,357
V2	80	17,811	4,762	15,178	5,691	3,130
V1	80	18,307	4,828	15,300	5,697	3,213
V1	80	17,122	4,669	15,030	5,698	3,005
V1	80	18,271	4,823	15,300	5,711	3,199
V2	80	18,126	4,804	15,249	5,711	3,174
V1	80	18,646	4,872	15,351	5,711	3,265
V2	80	18,356	4,834	15,458	5,711	3,214
V1	80	19,130	4,935	15,666	5,715	3,347
V2	80	18,973	4,915	15,544	5,715	3,320
V2	80	18,259	4,822	15,300	5,715	3,195
V1	80	18,211	4,815	15,397	5,715	3,187
V1	80	18,077	4,798	15,239	5,715	3,163
V2	80	18,888	4,904	15,559	5,715	3,305
V2	80	17,702	4,748	15,091	5,717	3,097
V2	80	19,445	4,976	15,839	5,717	3,401
V1	80	18,695	4,879	15,509	5,717	3,270
V2	80	18,453	4,847	15,351	5,719	3,227
V1	80	17,013	4,654	14,822	5,719	2,975
V2	80	19,566	4,991	15,875	5,719	3,421
V1	80	18,223	4,817	15,351	5,724	3,184
V1	80	17,327	4,697	15,030	5,724	3,027
V1	80	19,300	4,957	15,768	5,724	3,372
V1	80	18,586	4,865	15,473	5,724	3,247
V1	80	18,344	4,833	15,351	5,724	3,205
V2	50	20,824	5,149	16,541	6,510	3,199
V2	50	22,155	5,311	16,969	6,303	3,515
V2	50	20,727	5,137	16,332	6,183	3,352
V2	50	20,086	5,057	16,160	5,940	3,381
V2	50	21,187	5,194	16,566	6,182	3,427
V2	50	19,433	4,974	15,839	5,890	3,299
V2	50	21,030	5,175	16,602	6,210	3,386
V2	50	20,582	5,119	16,612	6,510	3,162
V2	50	23,317	5,449	17,523	6,556	3,556
V2	50	21,732	5,260	16,872	6,454	3,367
V2	50	23,111	5,425	17,375	6,488	3,562
V2	50	20,316	5,086	16,674	6,427	3,161
V2	50	18,501	4,853	15,412	5,867	3,153
V2	50	21,284	5,206	16,749	6,373	3,340
V2	50	21,223	5,198	16,836	6,483	3,274

V2	50	20,715	5,136	16,429	6,333	3,271
V2	50	21,090	5,182	16,577	6,274	3,361
V2	50	21,562	5,240	16,663	6,083	3,545
V2	50	20,836	5,151	16,368	6,182	3,370
V2	50	21,841	5,273	16,983	6,400	3,413
V2	50	19,529	4,987	15,890	5,882	3,320
V2	50	21,151	5,189	16,785	6,406	3,302
V2	50	21,659	5,251	16,724	6,358	3,407
V2	50	21,344	5,213	16,612	6,359	3,357
V2	50	19,905	5,034	16,012	5,918	3,363
V2	50	16,577	4,594	14,803	5,490	3,019
V2	50	21,236	5,200	16,562	6,168	3,443
V2	50	21,018	5,173	16,688	6,237	3,370
V2	50	20,292	5,083	16,246	6,262	3,240
V2	50	21,030	5,175	16,490	6,170	3,409
V2	50	21,744	5,262	16,760	6,062	3,587
V2	50	21,284	5,206	16,541	6,223	3,420
V2	50	21,236	5,200	16,562	6,168	3,443
V2	50	21,018	5,173	16,688	6,237	3,370
V2	50	20,292	5,083	16,246	6,262	3,240
V2	50	21,030	5,175	16,490	6,170	3,409
V2	50	21,744	5,262	16,760	6,062	3,587
V2	50	21,284	5,206	16,541	6,223	3,420
V2	50	22,264	5,324	16,897	6,264	3,555
V2	50	20,401	5,097	16,246	6,264	3,257
V2	50	21,490	5,231	16,663	6,222	3,454
V2	50	21,102	5,183	16,541	6,184	3,413
V2	50	18,549	4,860	15,483	5,827	3,183
V2	50	21,465	5,228	16,663	6,352	3,379
V2	50	21,816	5,270	16,739	6,032	3,617
V2	50	22,288	5,327	17,436	6,643	3,355
V2	50	18,344	4,833	15,361	5,772	3,178
V2	50	24,006	5,529	17,965	6,827	3,516
V2	50	24,394	5,573	18,062	6,643	3,672
V2	50	20,957	5,166	16,490	6,111	3,429
V2	50	20,473	5,106	16,160	6,021	3,400
V2	50	19,941	5,039	15,951	6,031	3,307
V2	50	18,779	4,890	15,753	5,927	3,168
V2	50	19,953	5,040	16,048	5,917	3,372
V2	50	20,098	5,059	16,343	6,357	3,162
V2	50	17,388	4,705	15,239	5,955	2,920
V2	50	20,715	5,136	16,846	6,386	3,244
V2	50	22,579	5,362	17,375	6,944	3,251
V2	50	20,255	5,078	16,318	6,168	3,284
V2	50	18,658	4,874	15,534	5,927	3,148
V2	50	21,187	5,194	16,627	6,277	3,375
V2	50	19,445	4,976	16,012	5,918	3,285
V2	50	22,784	5,386	17,289	6,673	3,414
V2	50	18,997	4,918	15,656	5,882	3,230
V2	50	20,473	5,106	16,332	6,148	3,330
V2	50	16,105	4,528	14,415	5,541	2,907
V2	50	23,401	5,459	17,523	6,593	3,549

V2	50	19,082	4,929	15,620	6,030	3,164
V2	50	20,570	5,118	16,490	6,238	3,298
V2	50	19,578	4,993	15,951	5,937	3,298
V2	50	21,478	5,229	16,785	6,241	3,442
V2	50	20,497	5,109	16,256	6,030	3,399
V2	50	20,292	5,083	16,379	6,032	3,364
V2	50	18,211	4,815	15,437	5,865	3,105
V2	50	18,852	4,899	15,620	5,787	3,257
V2	50	20,110	5,060	16,037	6,030	3,335
V2	50	19,747	5,014	15,768	5,772	3,421
V2	50	20,594	5,121	16,307	6,118	3,366
V2	50	22,930	5,403	17,106	6,378	3,595
V2	50	19,965	5,042	16,195	6,083	3,282
V2	50	18,803	4,893	15,570	5,955	3,157
V2	50	21,030	5,175	16,332	6,065	3,468
V2	50	21,744	5,262	16,699	6,227	3,492
V2	50	21,006	5,172	16,541	5,982	3,512
V2	50	16,771	4,621	14,735	5,598	2,996
V2	50	20,243	5,077	16,160	5,917	3,421
V2	50	20,739	5,139	16,393	6,210	3,340
V2	50	21,647	5,250	16,846	6,278	3,448
V2	50	20,679	5,131	16,577	6,381	3,241
V2	50	20,679	5,131	16,282	6,030	3,429
V2	50	20,231	5,075	16,587	6,189	3,269
V2	50	20,776	5,143	16,419	6,275	3,311
V2	50	19,457	4,977	15,865	6,111	3,184
V2	50	21,332	5,212	16,699	6,264	3,405
V2	50	19,675	5,005	15,987	6,072	3,240
V2	50	21,889	5,279	16,994	6,618	3,307
V2	50	20,606	5,122	16,282	6,127	3,363
V2	50	20,207	5,072	16,134	5,928	3,409
V2	50	19,457	4,977	15,829	5,971	3,258
V2	50	19,469	4,979	16,185	6,444	3,021
V2	50	21,804	5,269	16,958	6,363	3,427
V2	50	22,554	5,359	17,289	6,490	3,475
V2	50	20,534	5,113	16,368	6,265	3,277
V2	50	21,187	5,194	16,541	6,128	3,458
V2	50	20,764	5,142	16,419	6,058	3,427
V2	50	22,712	5,377	17,080	6,378	3,561
V2	50	21,671	5,253	16,907	6,514	3,327
V2	50	19,203	4,945	15,681	5,918	3,245
V2	50	20,594	5,121	16,429	6,006	3,429
V2	50	20,304	5,084	16,343	6,218	3,266
V2	50	21,344	5,213	16,566	5,971	3,574
V2	50	20,957	5,166	16,566	6,263	3,346
V2	50	21,018	5,173	16,663	6,301	3,336
V2	50	21,042	5,176	16,490	6,005	3,504
V2	50	19,215	4,946	15,865	5,898	3,258
V2	50	20,824	5,149	16,455	6,221	3,347
V2	50	21,465	5,228	16,836	6,332	3,390
V2	50	23,172	5,432	17,462	6,532	3,548
V2	50	21,647	5,250	16,907	6,363	3,402

V2	50	19,977	5,043	16,185	6,120	3,264
V2	50	19,542	4,988	15,656	5,865	3,332
V2	50	20,933	5,163	16,480	6,217	3,367
V2	50	20,147	5,065	16,307	6,184	3,258
V2	50	20,086	5,057	16,134	5,845	3,436
V2	50	21,635	5,248	16,627	6,317	3,425
V2	50	20,945	5,164	16,455	6,156	3,402
V2	50	21,441	5,225	16,663	6,317	3,394
V2	50	23,535	5,474	17,548	6,333	3,716
V2	50	21,828	5,272	16,836	6,318	3,455
V2	50	21,853	5,275	16,897	6,403	3,413
V2	50	20,352	5,091	16,170	5,918	3,439
V2	50	23,268	5,443	17,375	6,510	3,574
V2	50	22,845	5,393	17,472	6,481	3,525
V2	50	20,860	5,154	16,332	6,057	3,444
V2	50	21,260	5,203	16,638	6,197	3,431
V2	50	19,457	4,977	15,839	5,918	3,287
V2	50	22,518	5,355	17,019	6,096	3,694
V2	50	19,445	4,976	16,037	6,067	3,205
V2	50	22,542	5,357	17,019	6,403	3,520
V2	50	23,147	5,429	17,594	6,663	3,474
V2	50	22,034	5,297	17,004	6,171	3,570
V2	50	20,921	5,161	16,419	6,057	3,454
V2	50	21,683	5,254	16,785	6,197	3,499
V2	50	19,699	5,008	15,839	5,972	3,299
V2	50	23,135	5,427	17,314	6,199	3,732
V2	50	20,449	5,103	16,368	6,247	3,273
V2	50	21,611	5,246	16,775	6,157	3,510
V2	50	20,969	5,167	16,714	6,274	3,342
V2	50	22,724	5,379	17,573	6,756	3,363
V2	50	22,688	5,375	17,314	6,490	3,496
V2	50	21,417	5,222	16,627	6,358	3,369
V2	50	20,994	5,170	16,724	5,962	3,521
V2	50	20,897	5,158	16,419	6,164	3,390
V2	50	20,679	5,131	16,566	6,262	3,302
V2	50	22,010	5,294	16,861	6,318	3,484
V2	50	20,316	5,086	16,271	6,067	3,349
V2	50	21,006	5,172	16,602	6,247	3,363
V2	50	19,166	4,940	15,742	5,850	3,276
V2	50	19,529	4,987	16,195	5,772	3,383
V2	50	20,195	5,071	16,271	6,164	3,276
V2	50	22,857	5,395	17,386	6,483	3,526
V2	50	19,542	4,988	15,987	5,870	3,329
V2	50	20,038	5,051	16,037	6,071	3,300
V2	50	20,074	5,056	16,368	6,042	3,322
V2	50	22,361	5,336	17,350	6,457	3,463
V2	50	20,147	5,065	16,109	6,096	3,305
V2	50	21,744	5,262	17,019	6,454	3,369
V2	50	22,397	5,340	17,228	6,324	3,542
V2	50	19,711	5,010	16,073	6,030	3,269
V2	50	21,695	5,256	16,775	6,357	3,413
V2	50	20,195	5,071	16,195	5,928	3,407

V2	50	20,171	5,068	16,160	6,062	3,327
V2	50	20,618	5,124	16,368	6,171	3,341
V2	50	19,784	5,019	16,292	6,332	3,125
V2	50	19,106	4,932	15,814	6,096	3,134
V2	50	20,739	5,139	16,343	5,890	3,521
V2	50	21,308	5,209	16,627	6,184	3,446
V2	50	20,497	5,109	16,307	5,953	3,443
V2	50	20,776	5,143	16,480	6,094	3,409
V2	50	19,070	4,927	15,803	5,977	3,191
V2	50	20,994	5,170	16,516	6,237	3,366
V2	50	21,066	5,179	16,551	6,266	3,362
V2	50	22,337	5,333	17,253	6,544	3,413
V2	50	20,933	5,163	16,638	6,218	3,367
V2	50	20,110	5,060	16,012	6,096	3,299
V2	50	21,901	5,281	16,983	6,490	3,374
V2	50	19,626	4,999	15,915	6,030	3,255
V2	50	20,473	5,106	16,282	5,985	3,421
V2	50	20,292	5,083	16,282	6,042	3,358
V2	50	21,357	5,215	16,663	6,428	3,322
V2	50	20,413	5,098	16,358	6,087	3,354
V2	50	21,635	5,248	16,775	6,237	3,469
V2	50	19,263	4,952	15,900	6,071	3,173
V2	50	20,497	5,109	16,343	6,083	3,370
V2	50	20,280	5,081	16,246	6,168	3,288
V2	50	19,529	4,987	15,926	6,071	3,217
V2	50	22,022	5,295	16,948	6,223	3,539
V2	50	24,273	5,559	18,357	6,998	3,468
V2	50	22,881	5,398	17,314	6,544	3,497
V2	50	21,490	5,231	16,663	6,391	3,362
V2	50	19,784	5,019	16,109	5,977	3,310
V2	50	22,131	5,308	17,106	6,353	3,484
V2	50	19,554	4,990	16,098	6,183	3,162
V2	50	20,643	5,127	16,455	6,246	3,305
V2	50	20,800	5,146	16,455	6,363	3,269
V2	50	19,590	4,994	15,778	5,865	3,340
V2	50	20,618	5,124	16,551	6,278	3,284
V2	50	18,646	4,872	15,534	5,850	3,188
V2	50	20,159	5,066	16,246	6,156	3,274
V2	65	20,013	5,048	16,256	6,164	3,247
V2	65	17,993	4,786	15,300	5,814	3,095
V2	65	20,425	5,100	16,343	6,112	3,341
V2	65	19,929	5,037	16,368	6,257	3,185
V2	65	19,711	5,010	16,271	6,247	3,155
V2	65	19,590	4,994	16,037	6,087	3,218
V2	65	23,910	5,517	18,113	7,000	3,416
V2	65	19,578	4,993	16,048	6,062	3,230
V2	65	20,013	5,048	16,134	6,062	3,301
V2	65	20,606	5,122	16,465	6,112	3,372
V2	65	20,764	5,142	16,246	6,018	3,450
V2	65	20,013	5,048	16,256	6,164	3,247
V2	65	17,993	4,786	15,300	5,814	3,095
V2	65	20,425	5,100	16,343	6,112	3,341

V2	65	19,929	5,037	16,368	6,257	3,185
V2	65	19,711	5,010	16,271	6,247	3,155
V2	65	19,590	4,994	16,037	6,087	3,218
V2	65	19,578	4,993	16,048	6,062	3,230
V2	65	20,013	5,048	16,134	6,062	3,301
V2	65	20,606	5,122	16,465	6,112	3,372
V2	65	20,764	5,142	16,246	6,018	3,450
V2	65	20,800	5,146	16,358	5,911	3,519
V2	65	20,497	5,109	16,861	6,463	3,171
V2	65	19,457	4,977	15,865	6,034	3,225
V2	65	20,038	5,051	16,271	6,210	3,227
V2	65	19,554	4,990	16,088	6,071	3,221
V2	65	18,985	4,917	15,544	5,852	3,244
V2	65	22,058	5,300	16,785	6,125	3,602
V2	65	19,759	5,016	16,185	6,157	3,209
V2	65	18,767	4,888	16,002	6,438	2,915
V2	65	20,510	5,110	16,282	6,034	3,399
V2	65	20,631	5,125	16,368	6,310	3,269
V2	65	18,065	4,796	15,214	5,772	3,130
V2	65	21,574	5,241	16,872	6,400	3,371
V2	65	20,268	5,080	16,221	6,034	3,359
V2	65	20,497	5,109	16,612	6,260	3,275
V2	65	20,546	5,115	16,505	6,472	3,174
V2	65	25,725	5,723	18,601	6,863	3,748
V2	65	20,461	5,104	16,307	6,171	3,316
V2	65	22,349	5,334	17,131	6,332	3,529
V2	65	17,666	4,743	15,117	5,716	3,090
V2	65	19,517	4,985	15,839	5,815	3,357
V2	65	19,070	4,927	15,534	5,865	3,252
V2	65	20,836	5,151	16,393	6,032	3,454
V2	65	20,268	5,080	16,221	6,034	3,359
V2	65	20,497	5,109	16,612	6,260	3,275
V2	65	20,546	5,115	16,505	6,472	3,174
V2	65	20,461	5,104	16,307	6,171	3,316
V2	65	22,349	5,334	17,131	6,332	3,529
V2	65	17,666	4,743	15,117	5,716	3,090
V2	65	19,517	4,985	15,839	5,815	3,357
V2	65	19,070	4,927	15,534	5,865	3,252
V2	65	20,836	5,151	16,393	6,032	3,454
V2	65	22,034	5,297	16,872	6,356	3,466
V2	65	20,764	5,142	16,505	6,211	3,343
V2	65	19,602	4,996	15,829	5,965	3,286
V2	65	18,271	4,823	15,310	5,765	3,169
V2	65	20,485	5,107	16,455	6,303	3,250
V2	65	19,542	4,988	15,926	5,871	3,329
V2	65	19,251	4,951	15,814	5,793	3,323
V2	65	18,828	4,896	15,509	6,017	3,129
V2	65	19,941	5,039	16,073	6,112	3,262
V2	65	19,747	5,014	16,073	5,954	3,317
V2	65	19,033	4,923	15,768	5,842	3,258
V2	65	20,243	5,077	16,246	6,018	3,364
V2	65	19,675	5,005	16,221	6,218	3,164

V2	65	19,058	4,926	15,717	6,171	3,088
V2	65	19,929	5,037	15,951	5,955	3,347
V2	65	17,727	4,751	15,214	5,786	3,063
V2	65	18,695	4,879	15,595	6,030	3,100
V2	65	19,505	4,983	15,951	6,003	3,249
V2	65	21,949	5,286	17,238	6,545	3,354
V2	65	20,171	5,068	16,271	6,217	3,244
V2	65	20,981	5,169	16,627	6,072	3,455
V2	65	20,159	5,066	16,307	6,120	3,294
V2	65	20,812	5,148	16,429	6,200	3,357
V2	65	20,134	5,063	16,221	6,156	3,271
V2	65	19,602	4,996	16,063	6,148	3,189
V2	65	20,957	5,166	16,393	6,128	3,420
V2	65	20,110	5,060	16,282	6,264	3,210
V2	65	20,957	5,166	16,577	6,246	3,355
V2	65	19,300	4,957	15,865	5,940	3,249
V2	65	22,010	5,294	17,080	6,508	3,382
V2	65	19,372	4,966	15,768	5,918	3,273
V2	65	19,602	4,996	15,865	5,927	3,307
V2	65	22,930	5,403	17,202	6,659	3,443
V2	65	19,989	5,045	16,012	6,018	3,322
V2	65	19,324	4,960	15,839	5,845	3,306
V2	65	21,175	5,192	16,541	6,208	3,411
V2	65	19,675	5,005	16,124	6,111	3,220
V2	65	20,594	5,121	16,602	6,622	3,110
V2	65	22,300	5,329	17,518	6,749	3,304
V2	65	19,324	4,960	15,839	5,845	3,306
V2	65	21,175	5,192	16,541	6,208	3,411
V2	65	19,675	5,005	16,124	6,111	3,220
V2	65	20,594	5,121	16,602	6,622	3,110
V2	65	23,982	5,526	17,670	6,495	3,692
V2	65	22,010	5,294	17,080	6,310	3,488
V2	65	21,465	5,228	16,612	6,162	3,483
V2	65	20,098	5,059	16,429	6,278	3,201
V2	65	20,473	5,106	16,455	6,246	3,278
V2	65	21,877	5,278	16,994	6,298	3,474
V2	65	19,469	4,979	15,915	6,172	3,155
V2	65	21,877	5,278	16,897	6,427	3,404
V2	65	20,401	5,097	16,429	6,277	3,250
V2	65	20,098	5,059	16,160	6,031	3,333
V2	65	18,549	4,860	15,595	5,940	3,123
V2	65	19,082	4,929	15,707	6,018	3,171
V2	65	20,909	5,160	16,368	6,164	3,392
V2	65	18,380	4,838	15,544	5,765	3,188
V2	65	18,126	4,804	15,351	5,748	3,153
V2	65	16,577	4,594	15,066	5,880	2,819
V2	65	17,424	4,710	15,249	5,818	2,995
V2	65	17,158	4,674	15,005	5,686	3,017
V2	65	18,416	4,842	15,544	5,821	3,164
V2	65	15,936	4,504	14,588	5,645	2,823
V2	65	16,638	4,603	14,807	5,504	3,023
V2	65	18,707	4,880	15,768	5,940	3,149

V2	65	19,433	4,974	15,976	6,064	3,205
V2	65	21,006	5,172	16,516	6,331	3,318
V2	65	18,259	4,822	15,692	6,275	2,910
V2	65	19,820	5,023	15,987	6,064	3,268
V2	65	20,981	5,169	16,516	6,083	3,449
V2	65	19,929	5,037	15,976	5,937	3,357
V2	65	18,985	4,917	15,717	5,814	3,265
V2	65	19,929	5,037	16,048	5,845	3,409
V2	65	20,957	5,166	16,577	6,463	3,242
V2	65	18,876	4,902	15,803	6,003	3,144
V2	65	20,195	5,071	16,307	6,111	3,305
V2	65	22,083	5,302	17,243	6,359	3,473
V2	65	19,445	4,976	15,829	5,877	3,308
V2	65	19,154	4,938	15,915	6,057	3,162
V2	65	21,599	5,244	16,638	6,264	3,448
V2	65	18,924	4,909	15,753	5,793	3,267
V2	65	20,425	5,100	16,627	6,517	3,134
V2	65	21,006	5,172	16,612	6,189	3,394
V2	65	21,925	5,284	17,116	6,387	3,433
V2	65	20,304	5,084	16,469	6,208	3,270
V2	65	18,477	4,850	15,631	5,918	3,122
V2	65	19,433	4,974	15,915	6,018	3,229
V2	65	15,234	4,404	14,049	5,278	2,886
V2	65	19,166	4,940	15,692	5,737	3,341
V2	65	18,985	4,917	15,926	6,061	3,132
V2	65	19,021	4,921	15,936	5,977	3,182
V2	65	18,138	4,806	15,325	5,918	3,065
V2	65	19,505	4,983	16,073	6,140	3,177
V2	65	19,287	4,956	16,012	6,073	3,176
V2	65	20,558	5,116	16,221	6,071	3,386
V2	65	17,497	4,720	15,127	5,570	3,141
V2	65	19,614	4,997	15,890	5,850	3,353
V2	65	19,106	4,932	15,829	6,058	3,154
V2	65	15,524	4,446	14,232	5,174	3,001
V2	65	18,682	4,877	15,742	5,953	3,138
V2	65	24,236	5,555	17,904	6,806	3,561
V2	65	17,739	4,752	15,214	5,882	3,016
V2	65	18,852	4,899	15,646	5,748	3,279
V2	65	18,658	4,874	15,692	5,927	3,148
V2	65	18,622	4,869	15,620	5,814	3,203
V2	65	18,888	4,904	15,803	6,096	3,098
V2	65	19,650	5,002	16,160	6,278	3,130
V2	65	19,287	4,956	15,900	6,005	3,212
V2	65	21,611	5,246	16,922	6,524	3,313
V2	65	23,607	5,482	18,077	7,193	3,282
V2	65	20,836	5,151	16,602	6,265	3,326
V2	65	18,598	4,866	15,422	5,686	3,271
V2	65	20,316	5,086	16,256	6,171	3,292
V2	65	21,115	5,185	16,775	6,563	3,217
V2	65	20,873	5,155	16,688	6,483	3,220
V2	65	18,997	4,918	16,073	6,170	3,079
V2	65	19,009	4,920	15,717	5,913	3,215

V2	65	20,364	5,092	16,221	6,275	3,245
V2	65	19,324	4,960	16,037	6,318	3,059
V2	65	17,835	4,765	15,498	6,157	2,897
V2	65	19,106	4,932	15,803	5,982	3,194
V2	65	19,227	4,948	15,742	5,922	3,246
V2	65	20,401	5,097	16,307	6,237	3,271
V2	65	19,106	4,932	15,976	6,073	3,146
V1	80	18,017	4,790	15,351	5,724	3,148
V2	80	18,682	4,877	15,519	5,725	3,263
V2	80	18,537	4,858	15,325	5,725	3,238
V1	80	18,453	4,847	15,509	5,725	3,223
V1	80	19,179	4,942	15,681	5,731	3,346
V2	80	18,900	4,906	15,559	5,731	3,298
V2	80	17,884	4,772	15,239	5,731	3,120
V1	80	17,013	4,654	15,020	5,731	2,968
V2	80	19,421	4,973	15,839	5,736	3,385
V1	80	18,344	4,833	15,580	5,737	3,198
V2	80	18,912	4,907	15,544	5,743	3,293
V1	80	17,642	4,739	15,152	5,743	3,072
V2	80	19,699	5,008	15,768	5,748	3,427
V1	80	19,566	4,991	15,768	5,748	3,404
V2	80	19,892	5,033	15,890	5,751	3,459
V1	80	18,658	4,874	15,473	5,751	3,244
V2	80	18,477	4,850	15,412	5,751	3,213
V1	80	19,820	5,023	16,037	5,760	3,441
V2	80	18,949	4,912	15,707	5,760	3,290
V1	80	19,784	5,019	15,926	5,764	3,432
V2	80	18,840	4,898	15,656	5,764	3,268
V1	80	19,469	4,979	15,814	5,764	3,378
V1	80	18,077	4,798	15,386	5,765	3,136
V1	80	18,598	4,866	15,570	5,765	3,226
V2	80	18,453	4,847	15,412	5,772	3,197
V2	80	17,569	4,730	15,056	5,772	3,044
V1	80	17,436	4,712	15,030	5,772	3,021
V2	80	19,408	4,971	15,742	5,772	3,363
V1	80	17,509	4,722	15,091	5,772	3,034
V1	80	19,408	4,971	15,757	5,772	3,363
V2	80	18,114	4,802	15,152	5,772	3,138
V1	80	17,993	4,786	15,249	5,772	3,117
V1	80	16,589	4,596	14,847	5,772	2,874
V1	80	18,477	4,850	15,361	5,772	3,201
V1	80	19,675	5,005	15,839	5,772	3,408
V2	80	18,803	4,893	15,509	5,772	3,258
V2	80	18,537	4,858	15,483	5,776	3,209
V1	80	18,561	4,861	15,458	5,777	3,213
V1	80	19,602	4,996	15,829	5,786	3,388
V2	80	18,271	4,823	15,239	5,786	3,158
V1	80	17,956	4,782	15,300	5,786	3,103
V2	80	17,932	4,778	15,264	5,786	3,099
V2	80	17,932	4,778	15,264	5,786	3,099
V1	80	19,094	4,931	15,620	5,786	3,300
V1	80	18,937	4,910	15,509	5,786	3,273

V1	80	17,981	4,785	15,275	5,786	3,108
V2	80	18,102	4,801	15,214	5,786	3,128
V1	80	19,856	5,028	15,940	5,786	3,431
V2	80	19,408	4,971	16,098	5,786	3,354
V2	80	19,554	4,990	15,768	5,787	3,379
V2	80	19,408	4,971	15,793	5,787	3,354
V2	80	18,465	4,849	15,534	5,787	3,191
V1	80	17,932	4,778	15,178	5,787	3,099
V2	80	17,860	4,769	15,178	5,787	3,086
V2	80	18,465	4,849	15,412	5,787	3,191
V2	80	18,658	4,874	15,473	5,787	3,224
V1	80	17,835	4,765	15,239	5,787	3,082
V2	80	19,130	4,935	15,742	5,787	3,306
V2	80	18,900	4,906	15,570	5,787	3,266
V2	80	18,658	4,874	15,498	5,787	3,224
V1	80	17,896	4,773	15,239	5,787	3,092
V1	80	19,445	4,976	15,900	5,793	3,357
V2	80	18,840	4,898	15,692	5,793	3,252
V1	80	17,993	4,786	15,300	5,793	3,106
V2	80	19,033	4,923	15,728	5,793	3,286
V1	80	18,900	4,906	15,692	5,793	3,263
V2	80	18,453	4,847	15,605	5,793	3,185
V1	80	18,307	4,828	15,422	5,793	3,160
V1	80	19,626	4,999	15,900	5,793	3,388
V1	80	20,038	5,051	16,012	5,807	3,451
V1	80	18,707	4,880	15,605	5,807	3,222
V2	80	18,961	4,913	15,534	5,812	3,263
V2	80	18,937	4,910	15,559	5,812	3,258
V2	80	18,065	4,796	15,178	5,812	3,108
V2	80	19,396	4,970	15,768	5,814	3,336
V2	80	19,638	5,000	15,900	5,814	3,378
V1	80	18,997	4,918	15,534	5,814	3,267
V1	80	18,900	4,906	15,753	5,814	3,251
V1	80	18,537	4,858	15,544	5,814	3,188
V1	80	17,569	4,730	15,336	5,814	3,022
V1	80	18,949	4,912	15,768	5,814	3,259
V1	80	18,719	4,882	15,768	5,814	3,220
V2	80	18,561	4,861	15,534	5,814	3,192
V2	80	18,816	4,895	15,559	5,814	3,236
V2	80	19,735	5,013	15,839	5,815	3,394
V2	80	19,058	4,926	15,534	5,815	3,278
V2	80	19,759	5,016	15,839	5,818	3,396
V2	80	18,767	4,888	15,534	5,818	3,226
V2	80	18,743	4,885	15,473	5,818	3,221
V1	80	19,699	5,008	15,890	5,819	3,386
V1	80	18,295	4,826	15,325	5,819	3,144
V1	80	18,416	4,842	15,422	5,819	3,165
V2	80	19,626	4,999	15,900	5,821	3,372
V1	80	19,348	4,963	15,717	5,821	3,324
V2	80	19,275	4,954	15,681	5,821	3,311
V1	80	18,416	4,842	15,483	5,821	3,164
V2	80	19,626	4,999	15,961	5,821	3,372

V1	80	19,723	5,011	15,865	5,822	3,388
V2	80	19,517	4,985	15,803	5,822	3,353
V2	80	19,408	4,971	15,839	5,822	3,334
V2	80	19,336	4,962	15,707	5,822	3,321
V2	80	19,142	4,937	15,631	5,822	3,288
V2	80	18,924	4,909	15,631	5,822	3,251
V2	80	20,703	5,134	16,246	5,825	3,554
V2	80	19,856	5,028	16,063	5,825	3,409
V1	80	19,433	4,974	15,803	5,825	3,336
V2	80	19,348	4,963	15,656	5,825	3,322
V1	80	18,900	4,906	15,631	5,825	3,245
V2	80	18,549	4,860	15,386	5,825	3,184
V2	80	17,739	4,752	15,239	5,825	3,045
V1	80	19,626	4,999	15,717	5,826	3,369
V1	80	19,529	4,987	15,742	5,826	3,352
V1	80	18,440	4,846	15,386	5,826	3,165
V2	80	19,275	4,954	15,900	5,826	3,309
V1	80	19,191	4,943	15,814	5,826	3,294
V1	80	20,594	5,121	16,221	5,826	3,535
V2	80	19,094	4,931	15,753	5,826	3,277
V2	80	19,650	5,002	15,742	5,826	3,373
V1	80	19,747	5,014	15,854	5,827	3,389
V1	80	19,493	4,982	15,915	5,827	3,345
V1	80	19,360	4,965	15,692	5,827	3,323
V2	80	19,203	4,945	15,681	5,827	3,296
V2	80	19,118	4,934	15,681	5,827	3,281
V2	80	19,106	4,932	15,620	5,827	3,279
V1	80	18,501	4,853	15,473	5,827	3,175
V1	80	17,727	4,751	15,239	5,827	3,042
V2	80	20,013	5,048	16,037	5,827	3,435
V1	80	19,735	5,013	15,890	5,827	3,387
V1	80	19,650	5,002	15,829	5,827	3,372
V2	80	17,581	4,731	15,030	5,827	3,017
V1	80	17,460	4,715	15,091	5,827	2,996
V2	80	19,784	5,019	15,915	5,841	3,387
V2	80	18,876	4,902	15,559	5,841	3,232
V2	80	19,445	4,976	15,778	5,841	3,329
V1	80	17,702	4,748	15,325	5,841	3,030
V2	80	18,900	4,906	15,595	5,842	3,235
V1	80	18,332	4,831	15,325	5,842	3,138
V1	80	21,296	5,207	16,429	5,845	3,644
V2	80	20,086	5,057	16,012	5,845	3,437
V1	80	19,832	5,025	16,073	5,845	3,393
V1	80	19,433	4,974	15,865	5,845	3,325
V2	80	19,408	4,971	15,839	5,845	3,321
V1	80	19,929	5,037	15,987	5,845	3,410
V2	80	19,796	5,020	15,951	5,845	3,387
V1	80	18,646	4,872	15,631	5,845	3,190
V1	80	18,295	4,826	15,544	5,845	3,130
V1	80	20,812	5,148	16,256	5,845	3,560
V2	80	19,263	4,952	15,961	5,845	3,295
V2	80	19,142	4,937	15,814	5,845	3,275

V1	80	19,070	4,927	15,681	5,850	3,260
V1	80	19,820	5,023	16,048	5,850	3,388
V1	80	20,691	5,133	16,256	5,852	3,536
V1	80	19,844	5,027	16,012	5,852	3,391
V2	80	19,747	5,014	15,839	5,852	3,375
V2	80	19,227	4,948	15,778	5,852	3,286
V1	80	19,021	4,921	15,534	5,852	3,251
V2	80	18,707	4,880	15,570	5,852	3,197
V2	80	18,537	4,858	15,605	5,852	3,168
V2	80	18,453	4,847	15,483	5,852	3,153
V2	80	19,675	5,005	15,803	5,852	3,362
V1	80	18,344	4,833	15,422	5,852	3,135
V2	80	19,832	5,025	15,839	5,852	3,389
V1	80	19,650	5,002	15,829	5,852	3,358
V2	80	19,396	4,970	15,915	5,852	3,314
V1	80	19,179	4,942	15,778	5,852	3,277
V1	80	19,094	4,931	15,570	5,865	3,256
V2	80	18,453	4,847	15,275	5,865	3,146
V2	80	19,554	4,990	15,976	5,867	3,333
V1	80	18,779	4,890	15,595	5,867	3,201
V1	80	17,872	4,770	15,386	5,867	3,046
V2	80	18,803	4,893	15,570	5,867	3,205
V1	80	18,537	4,858	15,447	5,867	3,159
V1	80	20,304	5,084	16,109	5,870	3,459
V2	80	19,650	5,002	15,900	5,870	3,347
V2	80	19,602	4,996	16,023	5,870	3,339
Control	Control	19,300	4,957	15,829	5,918	3,261
Control	Control	18,477	4,850	15,595	5,915	3,124
Control	Control	18,162	4,809	15,239	5,725	3,172
Control	Control	18,646	4,872	15,473	5,731	3,253
Control	Control	18,392	4,839	15,361	5,686	3,234
Control	Control	20,074	5,056	16,246	6,218	3,229
Control	Control	18,985	4,917	15,509	5,683	3,341
Control	Control	20,437	5,101	16,271	6,032	3,388
Control	Control	21,090	5,182	16,627	6,373	3,309
Control	Control	20,691	5,133	16,419	6,171	3,353
Control	Control	19,626	4,999	15,753	5,852	3,354
Control	Control	18,622	4,869	15,570	5,899	3,157
Control	Control	19,396	4,970	15,666	5,502	3,525
Control	Control	19,856	5,028	15,951	6,032	3,292
Control	Control	19,638	5,000	15,900	5,880	3,340
Control	Control	17,291	4,692	14,954	5,645	3,063
Control	Control	15,234	4,404	13,998	5,174	2,944
Control	Control	18,574	4,863	15,544	5,793	3,206
Control	Control	18,259	4,822	15,275	5,748	3,176
Control	Control	18,755	4,887	15,447	5,890	3,184
Control	Control	20,994	5,170	16,490	6,164	3,406
Control	Control	18,392	4,839	15,361	5,748	3,199
Control	Control	21,526	5,235	16,749	6,317	3,408
Control	Control	18,368	4,836	15,483	5,927	3,099
Control	Control	18,356	4,834	15,534	6,030	3,044
Control	Control	18,840	4,898	15,595	5,937	3,173

Control	Control	17,981	4,785	15,152	5,392	3,334
Control	Control	18,622	4,869	15,473	5,772	3,226
Control	Control	18,610	4,868	15,422	5,818	3,199
Control	Control	19,082	4,929	15,865	6,096	3,130
Control	Control	18,937	4,910	15,803	5,967	3,174
Control	Control	18,876	4,902	15,570	5,715	3,303
Control	Control	19,699	5,008	16,037	6,087	3,236
Control	Control	17,763	4,756	15,081	5,582	3,182
Control	Control	17,291	4,692	14,796	5,480	3,155
Control	Control	21,720	5,259	16,811	6,265	3,467
Control	Control	18,586	4,865	15,570	5,937	3,131
Control	Control	18,658	4,874	15,631	5,927	3,148
Control	Control	19,941	5,039	16,195	6,310	3,160
Control	Control	17,690	4,746	15,433	5,850	3,024
Control	Control	18,622	4,869	15,509	5,937	3,136
Control	Control	20,522	5,112	16,429	6,170	3,326
Control	Control	19,009	4,920	15,681	5,882	3,232
Control	Control	18,029	4,791	15,386	5,882	3,065
Control	Control	20,631	5,125	16,429	6,369	3,239
Control	Control	19,868	5,030	16,098	6,003	3,310
Control	Control	20,062	5,054	16,210	6,167	3,253
Control	Control	16,141	4,533	14,268	5,156	3,130
Control	Control	18,900	4,906	15,631	5,658	3,340
Control	Control	18,949	4,912	15,498	5,748	3,296
Control	Control	19,348	4,963	15,656	5,748	3,366
Control	Control	15,052	4,378	13,850	5,274	2,854
Control	Control	17,920	4,777	15,091	5,636	3,180
Control	Control	18,150	4,807	15,386	5,787	3,136
Control	Control	19,118	4,934	15,742	6,059	3,156
Control	Control	19,832	5,025	16,292	6,018	3,296
Control	Control	16,771	4,621	14,710	5,501	3,049
Control	Control	18,779	4,890	15,559	5,446	3,448
Control	Control	18,803	4,893	15,620	5,918	3,177
Control	Control	17,472	4,717	15,030	5,724	3,052
Control	Control	17,884	4,772	15,152	5,607	3,190
Control	Control	17,714	4,749	14,980	5,570	3,181
Control	Control	16,904	4,639	14,624	5,479	3,085
Control	Control	19,529	4,987	16,444	6,390	3,056
Control	Control	17,606	4,735	14,980	5,581	3,154
Control	Control	28,871	6,063	18,972	6,273	4,602
Control	Control	18,840	4,898	15,509	5,867	3,211
Control	Control	20,038	5,051	16,134	6,058	3,308
Control	Control	20,485	5,107	16,134	5,899	3,473
Control	Control	17,364	4,702	15,005	5,686	3,053
Control	Control	20,897	5,158	16,551	6,111	3,419
Control	Control	20,510	5,110	16,246	6,183	3,317
Control	Control	18,622	4,869	15,717	6,034	3,086
Control	Control	19,481	4,980	16,012	6,042	3,224
Control	Control	18,077	4,798	15,300	5,715	3,163
Control	Control	18,670	4,876	15,473	5,819	3,209
Control	Control	18,513	4,855	15,509	5,826	3,178
Control	Control	18,779	4,890	15,534	5,751	3,265

Control	Control	20,159	5,066	16,480	6,072	3,320
Control	Control	19,336	4,962	15,900	5,984	3,231
Control	Control	17,485	4,718	15,041	5,645	3,097
Control	Control	19,856	5,028	16,073	6,091	3,260
Control	Control	18,344	4,833	15,361	5,698	3,219
Control	Control	20,268	5,080	16,185	6,057	3,346
Control	Control	17,097	4,666	14,822	5,526	3,094
Control	Control	15,791	4,484	14,232	5,419	2,914
Control	Control	19,203	4,945	15,717	5,826	3,296
Control	Control	19,408	4,971	16,109	6,072	3,196
Control	Control	17,811	4,762	15,178	5,645	3,155
Control	Control	18,924	4,909	15,595	5,826	3,248
Control	Control	19,154	4,938	15,656	5,872	3,262
Control	Control	17,981	4,785	15,081	5,480	3,281
Control	Control	16,880	4,636	14,771	5,637	2,994
Control	Control	19,808	5,022	16,292	6,275	3,157
Control	Control	18,356	4,834	15,570	5,934	3,093
Control	Control	18,102	4,801	15,509	5,882	3,078
Control	Control	19,650	5,002	16,012	5,977	3,288
Control	Control	21,514	5,234	16,846	6,324	3,402
Control	Control	19,215	4,946	15,742	5,852	3,283
Control	Control	16,867	4,634	14,674	5,479	3,078
Control	Control	18,489	4,852	15,351	5,446	3,395
Control	Control	21,163	5,191	16,775	6,509	3,251
Control	Control	21,211	5,197	16,455	6,112	3,470
Control	Control	19,179	4,942	16,210	6,519	2,942
Control	Control	19,650	5,002	16,098	6,071	3,237
Control	Control	21,369	5,216	16,760	6,274	3,406
Control	Control	18,888	4,904	15,753	5,928	3,186
Control	Control	21,042	5,176	16,627	6,363	3,307
Control	Control	20,098	5,059	16,098	5,993	3,354
Control	Control	22,022	5,295	16,811	6,317	3,486
Control	Control	19,360	4,965	16,048	6,169	3,138
Control	Control	19,929	5,037	16,073	5,871	3,395
Control	Control	21,453	5,226	16,749	6,363	3,371
Control	Control	22,240	5,321	16,969	6,117	3,636
Control	Control	19,796	5,020	16,271	6,273	3,155
Control	Control	21,042	5,176	16,627	6,363	3,307
Control	Control	20,098	5,059	16,098	5,993	3,354
Control	Control	22,022	5,295	16,811	6,317	3,486
Control	Control	19,360	4,965	16,048	6,169	3,138
Control	Control	19,929	5,037	16,073	5,871	3,395
Control	Control	21,453	5,226	16,749	6,363	3,371
Control	Control	19,796	5,020	16,271	6,273	3,155
Control	Control	18,731	4,884	15,742	6,091	3,075
Control	Control	20,376	5,094	16,282	6,162	3,307
Control	Control	19,082	4,929	15,803	6,134	3,111
Control	Control	19,735	5,013	16,419	6,523	3,025
Control	Control	22,615	5,366	17,472	6,602	3,425
Control	Control	18,731	4,884	15,742	6,091	3,075
Control	Control	20,376	5,094	16,282	6,162	3,307
Control	Control	19,082	4,929	15,803	6,134	3,111

Control	Control	19,735	5,013	16,419	6,523	3,025
Control	Control	20,425	5,100	16,332	6,277	3,254
Control	Control	20,582	5,119	16,343	6,207	3,316
Control	Control	18,247	4,820	15,336	5,687	3,209
Control	Control	22,119	5,307	16,933	6,519	3,393
V1	50	18,404	4,841	15,473	5,818	3,163
V1	50	17,194	4,679	14,771	5,520	3,115
V1	50	17,158	4,674	14,883	5,621	3,052
V1	50	18,876	4,902	15,534	5,822	3,242
V1	50	18,162	4,809	15,325	5,852	3,104
V1	50	18,924	4,909	15,793	5,814	3,255
V1	50	18,876	4,902	15,656	5,977	3,158
V1	50	19,614	4,997	15,926	5,956	3,293
V1	50	17,714	4,749	15,066	5,580	3,175
V1	50	18,368	4,836	15,422	5,736	3,202
V1	50	19,481	4,980	15,839	5,673	3,434
V1	50	19,723	5,011	16,221	6,358	3,102
V1	50	17,908	4,775	15,300	5,772	3,102
V1	50	17,218	4,682	14,857	5,504	3,128
V1	50	16,831	4,629	14,796	5,472	3,076
V1	50	18,005	4,788	15,239	5,715	3,150
V1	50	17,666	4,743	15,066	5,523	3,199
V1	50	15,488	4,441	14,110	5,264	2,942
V1	50	19,554	4,990	15,839	5,852	3,342
V1	50	17,146	4,672	14,883	5,571	3,078
V1	50	17,001	4,652	14,857	5,731	2,966
V1	50	18,707	4,880	15,458	5,715	3,273
V1	50	17,751	4,754	15,066	5,570	3,187
V1	50	17,848	4,767	15,336	6,018	2,966
V1	50	16,964	4,648	14,746	5,429	3,125
V1	50	18,041	4,793	15,336	5,764	3,130
V1	50	19,384	4,968	15,803	5,772	3,358
V1	50	18,537	4,858	15,422	5,609	3,305
V1	50	19,106	4,932	15,681	5,724	3,338
V1	50	18,864	4,901	15,631	5,826	3,238
V1	50	21,139	5,188	16,749	6,031	3,505
V1	50	16,988	4,651	14,761	5,580	3,044
V1	50	20,243	5,077	16,419	6,303	3,212
V1	50	16,988	4,651	14,761	5,580	3,044
V1	50	20,243	5,077	16,419	6,303	3,212
V1	50	17,243	4,685	14,969	5,634	3,061
V1	50	18,598	4,866	15,509	5,821	3,195
V1	50	18,755	4,887	15,447	5,697	3,292
V1	50	17,497	4,720	15,127	5,501	3,181
V1	50	17,206	4,681	14,857	5,368	3,206
V1	50	18,380	4,838	15,249	5,672	3,240
V1	50	19,348	4,963	15,681	5,825	3,322
V1	50	18,537	4,858	15,422	5,637	3,289
V1	50	17,606	4,735	15,152	5,590	3,149
V1	50	19,142	4,937	15,631	5,871	3,260
V1	50	17,291	4,692	15,163	5,764	3,000
V1	50	19,832	5,025	15,976	6,030	3,289

V1	50	18,719	4,882	15,595	5,850	3,200
V1	50	17,606	4,735	15,066	5,765	3,054
V1	50	17,097	4,666	14,883	5,520	3,098
V1	50	18,537	4,858	15,605	5,793	3,200
V1	50	17,920	4,777	15,127	5,526	3,243
V1	50	19,493	4,982	15,803	5,937	3,283
V1	50	17,521	4,723	15,066	5,743	3,051
V1	50	20,195	5,071	16,134	5,993	3,370
V1	50	19,832	5,025	15,976	6,030	3,289
V1	50	18,719	4,882	15,595	5,850	3,200
V1	50	17,606	4,735	15,066	5,765	3,054
V1	50	17,097	4,666	14,883	5,520	3,098
V1	50	18,537	4,858	15,605	5,793	3,200
V1	50	17,920	4,777	15,127	5,526	3,243
V1	50	19,493	4,982	15,803	5,937	3,283
V1	50	17,521	4,723	15,066	5,743	3,051
V1	50	20,195	5,071	16,134	5,993	3,370
V1	50	16,420	4,572	14,613	5,645	2,909
V1	50	18,973	4,915	15,447	5,725	3,314
V1	50	19,723	5,011	16,195	6,168	3,198
V1	50	18,791	4,891	15,595	5,965	3,150
V1	50	18,198	4,814	15,239	5,776	3,150
V1	50	19,070	4,927	15,742	5,937	3,212
V1	50	16,976	4,649	14,822	5,673	2,992
V1	50	18,259	4,822	15,214	5,621	3,248
V1	50	18,126	4,804	15,275	5,786	3,132
V1	50	18,077	4,798	15,214	5,661	3,194
V1	50	19,396	4,970	15,742	5,814	3,336
V1	50	17,884	4,772	15,325	5,691	3,143
V1	50	19,796	5,020	16,037	5,995	3,302
V1	50	17,981	4,785	15,224	5,446	3,302
V1	50	19,844	5,027	15,976	5,971	3,323
V1	50	18,779	4,890	15,473	5,692	3,299
V1	50	18,864	4,901	15,595	5,786	3,260
V1	50	18,634	4,871	15,509	5,918	3,149
V1	50	17,642	4,739	15,152	5,590	3,156
V1	50	20,522	5,112	16,160	5,880	3,490
V1	50	19,166	4,940	15,656	5,825	3,290
V1	50	17,932	4,778	15,325	5,881	3,049
V1	50	17,424	4,710	14,908	5,486	3,176
V1	50	19,154	4,938	15,620	5,719	3,349
V1	50	17,049	4,659	14,919	5,446	3,131
V1	50	17,956	4,782	15,152	5,580	3,218
V1	50	22,651	5,370	17,350	6,607	3,428
V1	50	21,611	5,246	17,141	6,459	3,346
V1	50	21,962	5,288	17,177	6,537	3,360
V1	50	18,017	4,790	15,361	5,749	3,134
V1	50	20,679	5,131	16,526	6,127	3,375
V1	50	23,135	5,427	17,314	6,246	3,704
V1	50	18,198	4,814	15,768	6,128	2,970
V1	50	17,956	4,782	15,447	5,904	3,041
V1	50	20,800	5,146	16,307	5,972	3,483

V1	50	18,162	4,809	15,361	5,744	3,162
V1	50	21,828	5,272	16,907	6,171	3,537
V1	50	20,183	5,069	15,865	5,772	3,497
V1	50	18,876	4,902	15,534	5,827	3,239
V1	50	23,934	5,520	18,235	7,189	3,329
V1	50	19,614	4,997	15,926	5,867	3,343
V1	50	19,977	5,043	16,455	6,247	3,198
V1	50	18,852	4,899	15,570	5,673	3,323
V1	50	20,534	5,113	16,307	6,167	3,329
V1	50	18,440	4,846	15,412	5,786	3,187
V1	50	17,315	4,695	14,944	5,619	3,081
V1	50	18,041	4,793	15,351	5,772	3,126
V1	50	18,658	4,874	15,361	5,765	3,236
V1	50	19,191	4,943	15,854	5,940	3,231
V1	50	16,577	4,594	14,735	5,581	2,970
V1	50	19,505	4,983	15,839	5,777	3,376
V1	50	18,670	4,876	15,361	5,683	3,285
V1	50	19,735	5,013	16,109	5,934	3,326
V1	50	19,650	5,002	15,742	5,927	3,315
V1	50	18,428	4,844	15,534	5,918	3,114
V1	50	18,356	4,834	15,214	5,570	3,296
V1	50	19,239	4,949	15,717	5,610	3,430
V1	50	17,654	4,741	15,005	5,580	3,164
V1	50	17,884	4,772	15,188	5,504	3,249
V1	50	15,621	4,460	13,998	5,134	3,043
V1	50	18,937	4,910	15,570	5,736	3,301
V1	50	19,263	4,952	15,778	5,736	3,358
V1	50	18,985	4,917	15,473	5,772	3,289
V1	50	21,272	5,204	16,907	6,197	3,433
V1	50	17,606	4,735	14,944	5,771	3,050
V1	50	20,255	5,078	16,109	5,993	3,380
V1	50	20,038	5,051	16,073	6,121	3,274
V1	50	20,292	5,083	16,282	6,274	3,234
V1	50	20,086	5,057	15,926	5,880	3,416
V1	50	20,219	5,074	16,185	6,167	3,278
V1	50	20,921	5,161	16,566	6,157	3,398
V1	50	19,747	5,014	15,854	5,927	3,332
V1	50	18,646	4,872	15,631	6,058	3,078
V1	50	22,034	5,297	17,055	6,348	3,471
V1	50	18,816	4,895	15,534	5,751	3,272
V1	50	19,614	4,997	15,865	5,899	3,325
V1	50	18,368	4,836	15,325	5,719	3,212
V1	50	19,215	4,946	15,778	5,927	3,242
V1	50	18,949	4,912	15,656	5,882	3,221
V1	50	20,280	5,081	16,246	5,956	3,405
V1	50	17,424	4,710	14,944	5,501	3,168
V1	50	17,376	4,704	14,857	5,607	3,099
V1	50	20,328	5,087	16,587	6,112	3,326
V1	50	18,840	4,898	15,509	5,827	3,233
V1	50	19,203	4,945	15,742	6,118	3,139
V1	50	20,425	5,100	16,195	5,870	3,479
V1	50	19,215	4,946	15,778	5,927	3,242

V1	50	18,949	4,912	15,656	5,882	3,221
V1	50	20,280	5,081	16,246	5,956	3,405
V1	50	17,424	4,710	14,944	5,501	3,168
V1	50	17,376	4,704	14,857	5,607	3,099
V1	50	18,840	4,898	15,509	5,827	3,233
V1	50	19,203	4,945	15,742	6,118	3,139
V1	50	20,425	5,100	16,195	5,870	3,479
V1	50	19,590	4,994	15,926	6,057	3,234
V1	50	18,864	4,901	15,681	5,904	3,195
V1	50	17,920	4,777	15,239	5,717	3,134
V1	50	18,501	4,853	15,717	5,963	3,103
V1	50	17,848	4,767	15,239	5,764	3,096
V1	65	16,988	4,651	14,893	5,638	3,013
V1	65	18,114	4,802	15,091	5,626	3,220
V1	65	19,009	4,920	15,829	5,967	3,185
V1	65	16,783	4,623	14,710	5,265	3,188
V1	65	17,400	4,707	15,102	5,771	3,015
V1	65	18,646	4,872	15,544	6,030	3,092
V1	65	17,666	4,743	15,041	5,532	3,193
V1	65	18,126	4,804	15,275	5,764	3,145
V1	65	18,670	4,876	15,544	5,637	3,312
V1	65	17,001	4,652	14,649	5,480	3,102
V1	65	17,569	4,730	15,275	5,927	2,964
V1	65	17,376	4,704	15,091	5,697	3,050
V1	65	19,300	4,957	16,037	6,011	3,211
V1	65	15,936	4,504	14,268	5,233	3,045
V1	65	17,351	4,700	15,056	5,751	3,017
V1	65	17,642	4,739	15,178	5,772	3,057
V1	65	18,682	4,877	15,753	6,057	3,084
V1	65	17,109	4,667	14,883	5,626	3,041
V1	65	16,553	4,591	14,735	5,570	2,972
V1	65	19,251	4,951	15,976	6,032	3,192
V1	65	18,803	4,893	15,656	5,971	3,149
V1	65	16,565	4,593	14,613	5,391	3,073
V1	65	16,952	4,646	14,796	5,580	3,038
V1	65	20,134	5,063	16,048	5,928	3,397
V1	65	17,037	4,657	14,822	5,626	3,028
V1	65	19,263	4,952	15,717	5,993	3,215
V1	65	17,412	4,708	14,857	5,519	3,155
V1	65	15,960	4,508	14,329	5,204	3,067
V1	65	18,259	4,822	15,264	5,692	3,208
V1	65	16,045	4,520	14,343	5,473	2,932
V1	65	18,392	4,839	15,570	6,017	3,057
V1	65	18,102	4,801	15,275	5,818	3,111
V1	65	17,642	4,739	15,117	5,661	3,116
V1	65	17,400	4,707	14,919	5,570	3,124
V1	65	17,702	4,748	15,066	5,552	3,188
V1	65	16,988	4,651	14,735	5,619	3,023
V1	65	17,606	4,735	15,249	5,718	3,079
V1	65	17,751	4,754	15,127	5,501	3,227
V1	65	17,908	4,775	15,188	5,711	3,136
V1	65	19,312	4,959	15,778	6,018	3,209

V1	65	19,917	5,036	16,098	6,094	3,268
V1	65	19,058	4,926	15,656	5,965	3,195
V1	65	18,380	4,838	15,412	5,724	3,211
V1	65	18,816	4,895	15,559	5,760	3,267
V1	65	18,162	4,809	15,249	5,581	3,255
V1	65	18,586	4,865	15,447	5,872	3,165
V1	65	18,344	4,833	15,386	5,760	3,185
V1	65	17,194	4,679	14,847	5,486	3,134
V1	65	17,654	4,741	15,066	5,581	3,163
V1	65	18,816	4,895	15,620	5,922	3,177
V1	65	18,864	4,901	15,584	5,645	3,341
V1	65	18,041	4,793	15,224	5,581	3,233
V1	65	16,238	4,547	14,354	5,233	3,103
V1	65	16,444	4,576	14,919	5,821	2,825
V1	65	19,723	5,011	15,951	5,993	3,291
V1	65	18,561	4,861	15,361	5,786	3,208
V1	65	18,223	4,817	15,483	5,736	3,177
V1	65	20,522	5,112	16,455	6,247	3,285
V1	65	20,013	5,048	16,012	5,956	3,360
V1	65	18,779	4,890	16,073	6,168	3,044
V1	65	19,082	4,929	15,839	5,724	3,334
V1	65	20,013	5,048	16,012	5,956	3,360
V1	65	16,795	4,624	15,030	5,474	3,068
V1	65	17,388	4,705	15,081	5,724	3,038
V1	65	16,867	4,634	14,761	5,446	3,097
V1	65	20,497	5,109	16,282	6,140	3,338
V1	65	18,138	4,806	15,325	5,787	3,134
V1	65	18,477	4,850	15,397	5,637	3,278
V1	65	20,171	5,068	16,098	6,058	3,330
V1	65	18,549	4,860	15,509	5,850	3,171
V1	65	18,682	4,877	15,422	5,715	3,269
V1	65	17,243	4,685	14,796	5,607	3,075
V1	65	18,077	4,798	15,275	5,609	3,223
V1	65	19,542	4,988	15,829	5,904	3,310
V1	65	17,920	4,777	15,163	5,438	3,295
V1	65	20,050	5,053	15,976	5,872	3,414
V1	65	18,211	4,815	15,264	5,598	3,253
V1	65	17,896	4,773	15,127	5,683	3,149
V1	65	15,294	4,413	14,598	5,503	2,779
V1	65	17,158	4,674	14,832	5,438	3,155
V1	65	18,465	4,849	15,412	5,661	3,262
V1	65	17,896	4,773	15,127	5,683	3,149
V1	65	17,158	4,674	14,832	5,438	3,155
V1	65	18,465	4,849	15,412	5,661	3,262
V1	65	17,678	4,744	15,127	5,609	3,152
V1	65	18,937	4,910	15,666	5,744	3,297
V1	65	19,929	5,037	16,012	5,962	3,342
V1	65	18,622	4,869	15,509	5,777	3,224
V1	65	19,408	4,971	16,429	5,918	3,280
V1	65	17,170	4,676	14,832	5,502	3,120
V1	65	17,920	4,777	15,188	5,552	3,228
V1	65	20,836	5,151	16,393	6,157	3,384

V1	65	18,138	4,806	15,152	5,626	3,224
V1	65	19,445	4,976	15,742	5,880	3,307
V1	65	17,775	4,757	15,285	5,637	3,153
V1	65	18,368	4,836	15,203	5,526	3,324
V1	65	16,674	4,608	14,624	5,526	3,017
V1	65	16,638	4,603	14,527	5,438	3,059
V1	65	17,872	4,770	15,081	5,619	3,181
V1	65	18,295	4,826	15,325	5,724	3,196
V1	65	18,150	4,807	15,386	5,772	3,144
V1	65	15,645	4,463	14,145	5,222	2,996
V1	65	18,634	4,871	15,544	5,736	3,248
V1	65	16,771	4,621	15,041	5,786	2,898
V1	65	17,848	4,767	15,188	5,503	3,243
V1	65	18,973	4,915	15,509	5,814	3,263
V1	65	17,593	4,733	15,005	5,626	3,127
V1	65	19,033	4,923	15,803	5,826	3,267
V1	65	16,287	4,554	14,624	5,445	2,991
V1	65	25,955	5,749	18,296	6,332	4,099
V1	65	16,420	4,572	14,466	5,473	3,000
V1	65	16,988	4,651	14,847	5,571	3,050
V1	65	18,162	4,809	15,300	5,852	3,104
V1	65	17,134	4,671	14,771	5,502	3,114
V1	65	18,138	4,806	15,228	5,504	3,295
V1	65	19,445	4,976	16,322	5,807	3,348
V1	65	19,033	4,923	15,803	5,826	3,267
V1	65	16,287	4,554	14,624	5,445	2,991
V1	65	16,420	4,572	14,466	5,473	3,000
V1	65	16,988	4,651	14,847	5,571	3,050
V1	65	18,162	4,809	15,300	5,852	3,104
V1	65	17,134	4,671	14,771	5,502	3,114
V1	65	18,138	4,806	15,228	5,504	3,295
V1	65	17,448	4,713	15,005	5,523	3,159
V1	65	16,517	4,586	14,771	5,552	2,975
V1	65	25,773	5,728	18,504	6,246	4,126
V1	65	16,323	4,559	14,379	5,566	2,932
V1	65	18,695	4,879	15,473	5,676	3,293
V1	65	18,404	4,841	15,376	5,626	3,271
V1	65	19,118	4,934	16,113	5,788	3,303
V1	65	17,448	4,713	15,005	5,523	3,159
V1	65	16,517	4,586	14,771	5,552	2,975
V1	65	16,323	4,559	14,379	5,566	2,932
V1	65	18,695	4,879	15,473	5,676	3,293
V1	65	18,404	4,841	15,376	5,626	3,271
V1	65	17,122	4,669	14,771	5,619	3,047
V1	65	17,073	4,662	14,771	5,445	3,135
V1	65	19,275	4,954	15,839	6,164	3,127
V1	65	18,428	4,844	15,447	5,812	3,171
V1	65	17,436	4,712	15,041	5,581	3,124
V1	65	17,315	4,695	14,969	5,544	3,123
V1	65	18,525	4,857	15,422	5,772	3,210
V1	65	20,570	5,118	16,134	5,845	3,519
V1	65	18,973	4,915	15,865	5,918	3,206

V1	65	17,230	4,684	14,908	5,571	3,093
V1	65	18,162	4,809	15,631	5,918	3,069
V1	65	16,613	4,599	14,563	5,467	3,039
V1	65	17,073	4,662	14,796	5,572	3,064
V1	65	17,315	4,695	14,919	5,645	3,067
V1	65	16,867	4,634	14,735	5,519	3,056
V1	65	16,746	4,618	14,710	5,552	3,017
V1	65	17,775	4,757	15,336	5,717	3,109
V1	80	19,287	4,956	15,839	5,870	3,286
V2	80	19,082	4,929	15,900	5,870	3,251
V2	80	19,070	4,927	15,692	5,870	3,248
V1	80	20,062	5,054	16,195	5,870	3,417
V2	80	19,529	4,987	16,023	5,870	3,327
V2	80	19,917	5,036	16,048	5,871	3,392
V2	80	19,626	4,999	15,926	5,871	3,343
V2	80	19,324	4,960	15,900	5,871	3,291
V2	80	19,275	4,954	15,778	5,871	3,283
V2	80	18,888	4,904	15,631	5,871	3,217
V1	80	20,497	5,109	16,124	5,871	3,491
V2	80	19,892	5,033	15,951	5,871	3,388
V1	80	19,239	4,949	15,681	5,871	3,277
V1	80	18,489	4,852	15,386	5,871	3,149
V1	80	18,150	4,807	15,422	5,871	3,091
V2	80	19,142	4,937	15,692	5,872	3,260
V1	80	19,021	4,921	15,570	5,872	3,239
V2	80	19,070	4,927	15,656	5,872	3,248
V2	80	19,820	5,023	15,951	5,877	3,372
V1	80	19,360	4,965	15,854	5,877	3,294
V1	80	19,275	4,954	15,768	5,877	3,280
V1	80	18,876	4,902	15,707	5,877	3,212
V1	80	20,122	5,062	16,023	5,880	3,422
V2	80	18,900	4,906	15,666	5,880	3,214
V1	80	18,319	4,830	15,483	5,880	3,116
V1	80	20,159	5,066	15,951	5,880	3,428
V2	80	19,808	5,022	16,048	5,880	3,369
V1	80	19,735	5,013	15,803	5,880	3,356
V2	80	19,433	4,974	15,778	5,880	3,305
V1	80	19,892	5,033	15,976	5,882	3,382
V2	80	19,070	4,927	15,620	5,882	3,242
V2	80	18,368	4,836	15,473	5,882	3,123
V2	80	19,336	4,962	15,803	5,882	3,287
V2	80	18,852	4,899	15,595	5,882	3,205
V1	80	18,283	4,825	15,447	5,882	3,108
V2	80	20,788	5,145	16,185	5,889	3,530
V1	80	19,929	5,037	15,976	5,889	3,384
V1	80	19,687	5,007	15,890	5,889	3,343
V1	80	19,348	4,963	15,753	5,889	3,285
V2	80	19,130	4,935	15,681	5,889	3,248
V1	80	18,961	4,913	15,595	5,889	3,220
V1	80	18,828	4,896	15,656	5,889	3,197
V2	80	18,513	4,855	15,509	5,889	3,143
V2	80	19,771	5,017	15,890	5,890	3,357

V2	80	18,803	4,893	15,570	5,890	3,193
V1	80	18,719	4,882	15,534	5,890	3,178
V1	80	19,759	5,016	16,073	5,896	3,351
V1	80	21,030	5,175	16,480	5,898	3,566
V2	80	19,094	4,931	15,829	5,898	3,237
V2	80	20,134	5,063	16,063	5,899	3,413
V2	80	20,050	5,053	16,012	5,899	3,399
V2	80	19,771	5,017	15,987	5,899	3,352
V2	80	19,687	5,007	15,926	5,899	3,337
V2	80	19,638	5,000	15,865	5,899	3,329
V1	80	19,433	4,974	15,926	5,899	3,294
V2	80	19,433	4,974	15,865	5,899	3,294
V1	80	19,348	4,963	15,987	5,899	3,280
V2	80	18,816	4,895	15,595	5,899	3,190
V1	80	20,352	5,091	16,170	5,899	3,450
V1	80	20,352	5,091	16,170	5,899	3,450
V2	80	19,505	4,983	15,839	5,899	3,307
V1	80	18,356	4,834	15,544	5,899	3,112
V2	80	20,812	5,148	16,282	5,899	3,528
V1	80	20,134	5,063	16,109	5,899	3,413
V2	80	19,445	4,976	15,839	5,899	3,296
V2	80	19,433	4,974	15,778	5,899	3,294
V1	80	19,130	4,935	16,109	5,899	3,243
V2	80	20,255	5,078	16,256	5,904	3,431
V1	80	20,110	5,060	16,271	5,904	3,406
V1	80	18,973	4,915	15,803	5,904	3,214
V2	80	18,937	4,910	15,681	5,904	3,207
V1	80	17,485	4,718	15,239	5,904	2,962
V2	80	19,336	4,962	15,854	5,904	3,275
V2	80	18,888	4,904	15,620	5,904	3,199
V2	80	21,078	5,181	16,419	5,911	3,566
V1	80	18,465	4,849	15,595	5,911	3,124
V1	80	20,449	5,103	16,210	5,911	3,459
V2	80	19,953	5,040	16,160	5,913	3,374
V2	80	19,941	5,039	16,063	5,913	3,372
V2	80	19,263	4,952	15,951	5,913	3,258
V1	80	17,944	4,780	15,361	5,913	3,035
V2	80	19,626	4,999	16,012	5,913	3,319
V1	80	19,300	4,957	15,768	5,913	3,264
V1	80	18,779	4,890	15,692	5,915	3,175
V2	80	19,759	5,016	16,134	5,915	3,340
V2	80	19,759	5,016	16,134	5,915	3,340
V1	80	20,885	5,157	16,379	5,917	3,529
V2	80	19,312	4,959	15,681	5,917	3,264
V1	80	17,763	4,756	15,275	5,917	3,002
V1	80	19,396	4,970	15,890	5,918	3,278
V1	80	18,949	4,912	15,631	5,918	3,202
V2	80	19,517	4,985	15,900	5,918	3,298
V2	80	19,106	4,932	15,631	5,918	3,228
V1	80	19,929	5,037	15,915	5,918	3,367
V1	80	19,663	5,004	15,865	5,918	3,323
V2	80	19,590	4,994	15,829	5,918	3,310

V1	80	19,433	4,974	15,915	5,918	3,284
V2	80	18,174	4,810	15,239	5,918	3,071
V2	80	20,727	5,137	16,343	5,918	3,502
V2	80	19,747	5,014	16,098	5,918	3,337
V2	80	19,227	4,948	15,803	5,918	3,249
V2	80	20,243	5,077	16,124	5,922	3,419
V1	80	19,372	4,966	15,742	5,922	3,271
V2	80	19,868	5,030	16,221	5,922	3,355
V2	80	19,784	5,019	15,926	5,922	3,341
V1	80	19,130	4,935	15,742	5,922	3,230
V1	80	18,900	4,906	15,620	5,922	3,192
V2	80	20,352	5,091	16,160	5,927	3,434
V1	80	20,013	5,048	16,037	5,927	3,377
V1	80	19,771	5,017	16,048	5,927	3,336
V2	80	19,384	4,968	15,681	5,927	3,271
V1	80	19,179	4,942	15,742	5,927	3,236
V1	80	20,026	5,049	15,951	5,927	3,379
V1	80	19,747	5,014	15,839	5,927	3,332
V1	80	19,166	4,940	15,803	5,927	3,234
V1	80	18,477	4,850	15,483	5,927	3,117
V1	80	18,404	4,841	15,422	5,927	3,105
V1	80	20,195	5,071	16,048	5,927	3,407
V1	80	19,941	5,039	16,012	5,927	3,364
V2	80	19,638	5,000	15,926	5,927	3,313
V2	80	19,166	4,940	15,803	5,927	3,234
V1	80	19,590	4,994	15,900	5,927	3,305
V2	80	19,481	4,980	15,778	5,927	3,287
V2	80	20,280	5,081	16,160	5,927	3,421
V1	80	19,953	5,040	15,951	5,928	3,366
V2	80	19,880	5,031	16,048	5,928	3,354
V2	80	20,159	5,066	16,073	5,934	3,397
V1	80	20,122	5,062	16,221	5,934	3,391
V1	80	20,086	5,057	16,195	5,934	3,385
V2	80	20,631	5,125	16,343	5,935	3,476
V2	80	20,618	5,124	16,221	5,935	3,474
V2	80	20,558	5,116	16,221	5,935	3,464
V1	80	20,497	5,109	16,318	5,935	3,454
V2	50	16,033	4,518	14,501	5,607	2,859
V2	50	17,351	4,700	14,822	5,542	3,131
V2	50	17,763	4,756	15,178	5,827	3,048
V2	50	21,671	5,253	16,872	6,563	3,302
V2	50	18,440	4,846	15,447	5,814	3,172
V2	50	18,924	4,909	16,063	6,247	3,029
V2	50	18,053	4,794	15,544	6,071	2,973
V2	50	16,371	4,566	14,761	5,607	2,920
V2	50	18,404	4,841	15,386	5,881	3,129
V2	50	18,888	4,904	15,473	5,814	3,249
V2	50	19,045	4,924	15,742	6,057	3,144
V2	50	16,916	4,641	14,832	5,711	2,962
V2	50	17,388	4,705	15,178	5,827	2,984
V2	50	18,646	4,872	15,717	5,889	3,166
V2	50	17,364	4,702	14,969	5,719	3,036

V2	50	18,138	4,806	15,325	5,814	3,120
V2	50	17,206	4,681	15,005	5,777	2,979
V2	50	15,210	4,401	13,962	5,290	2,875
V2	50	16,976	4,649	14,832	5,645	3,007
V2	50	18,053	4,794	15,214	5,609	3,219
V2	50	16,734	4,616	14,746	5,523	3,030
V2	50	17,134	4,671	14,649	5,373	3,189
V2	50	17,436	4,712	14,908	5,544	3,145
V2	50	16,541	4,589	14,674	5,517	2,998
V2	50	18,126	4,804	15,203	5,731	3,163
V2	50	17,981	4,785	15,325	5,787	3,107
V2	50	15,476	4,439	14,196	5,391	2,871
V2	50	18,828	4,896	15,595	5,826	3,232
V2	50	17,811	4,762	15,214	5,736	3,105
V2	50	16,976	4,649	14,771	5,645	3,007
V2	50	18,779	4,890	15,509	5,715	3,286
V2	50	18,102	4,801	15,239	5,658	3,199
V2	50	17,763	4,756	15,066	5,673	3,131
V2	50	17,448	4,713	15,117	5,760	3,029
V2	50	17,485	4,718	15,030	5,661	3,089
V2	50	16,214	4,544	14,613	5,581	2,905
V2	50	17,291	4,692	14,944	5,732	3,017
V2	50	16,238	4,547	14,440	5,438	2,986
V2	50	16,867	4,634	14,796	5,607	3,008
V2	50	16,831	4,629	14,735	5,732	2,936
V2	50	16,178	4,539	14,491	5,420	2,985
V2	50	17,448	4,713	15,056	5,544	3,148
V2	50	17,884	4,772	15,249	5,715	3,129
V2	50	18,065	4,796	15,300	5,793	3,118
V2	50	17,013	4,654	14,771	5,474	3,108
V2	50	17,557	4,728	15,091	5,827	3,013
V2	50	17,775	4,757	15,239	5,737	3,099
V2	50	16,311	4,557	14,379	5,363	3,041
V2	50	17,061	4,661	14,796	5,339	3,196
V2	50	18,259	4,822	15,361	5,717	3,194
V2	50	18,005	4,788	15,275	5,867	3,069
V2	50	18,670	4,876	15,447	5,936	3,145
V2	50	17,218	4,682	14,796	5,572	3,090
V2	50	20,594	5,121	16,109	5,927	3,475
V2	50	18,029	4,791	15,178	5,786	3,116
V2	50	19,517	4,985	15,961	6,005	3,250
V2	50	18,065	4,796	15,214	5,748	3,143
V2	50	17,001	4,652	14,980	5,609	3,031
V2	50	18,077	4,798	15,300	5,737	3,151
V2	50	17,678	4,744	15,091	5,765	3,066
V2	50	18,005	4,788	15,239	5,812	3,098
V2	50	18,162	4,809	15,412	5,787	3,138
V2	50	17,327	4,697	15,030	5,731	3,023
V2	50	16,686	4,609	14,847	5,771	2,891
V2	50	15,924	4,503	14,440	5,532	2,878
V2	50	18,719	4,882	15,595	5,880	3,184
V2	50	17,630	4,738	14,969	5,526	3,190

V2	50	16,141	4,533	14,440	5,503	2,933
V2	50	18,198	4,814	15,361	5,825	3,124
V2	50	16,347	4,562	14,613	5,672	2,882
V2	50	17,230	4,684	14,908	5,765	2,989
V2	50	18,622	4,869	15,656	5,956	3,127
V2	50	17,109	4,667	14,796	5,580	3,066
V2	50	18,440	4,846	15,473	5,748	3,208
V2	50	19,856	5,028	16,541	6,428	3,089
V2	50	19,142	4,937	15,646	5,719	3,347
V2	50	17,122	4,669	14,883	5,658	3,026
V2	50	17,364	4,702	14,832	5,504	3,154
V2	50	20,401	5,097	16,368	6,096	3,346
V2	50	19,009	4,920	15,681	5,842	3,254
V2	50	17,497	4,720	15,030	5,736	3,050
V2	50	16,166	4,537	14,700	5,672	2,850
V2	50	16,783	4,623	14,796	5,634	2,979
V2	50	18,319	4,830	15,534	5,940	3,084
V2	50	17,702	4,748	15,030	5,580	3,172
V2	50	19,106	4,932	15,717	5,852	3,265
V2	50	18,380	4,838	15,361	5,793	3,173
V2	50	17,001	4,652	14,796	5,523	3,078
V2	50	17,981	4,785	15,167	5,621	3,199
V2	50	17,884	4,772	15,300	5,967	2,997
V2	50	16,601	4,598	14,710	5,683	2,921
V2	50	17,920	4,777	15,239	5,751	3,116
V2	50	17,037	4,657	14,735	5,439	3,133
V2	50	16,650	4,604	14,624	5,502	3,026
V2	50	17,218	4,682	14,944	5,719	3,011
V2	50	16,045	4,520	14,405	5,481	2,927
V2	50	18,029	4,791	15,203	5,544	3,252
V2	50	17,981	4,785	15,239	5,686	3,162
V2	50	16,601	4,598	14,685	5,445	3,049
V2	50	19,227	4,948	16,185	6,264	3,070
V2	50	18,743	4,885	15,803	6,072	3,087
V2	50	17,654	4,741	15,397	6,018	2,934
V2	50	18,924	4,909	15,778	5,927	3,193
V2	50	17,194	4,679	15,030	5,827	2,951
V2	50	18,114	4,802	15,386	5,760	3,145
V2	50	19,747	5,014	16,271	6,310	3,130
V2	50	15,912	4,501	14,354	5,453	2,918
V2	50	17,666	4,743	15,239	5,751	3,072
V2	50	16,335	4,561	14,501	5,526	2,956
V2	50	16,976	4,649	14,944	5,607	3,028
V2	50	16,238	4,547	14,415	5,431	2,990
V2	50	17,448	4,713	14,980	5,396	3,234
V2	50	18,090	4,799	15,264	5,772	3,134
V2	50	17,908	4,775	15,066	5,417	3,306
V2	65	19,953	5,040	16,256	6,151	3,244
V2	65	19,892	5,033	16,073	6,140	3,240
V2	65	19,542	4,988	16,048	6,091	3,208
V2	65	19,106	4,932	15,940	6,148	3,108
V2	65	20,098	5,059	16,073	5,956	3,375

V2	65	19,771	5,017	16,221	6,331	3,123
V2	65	17,884	4,772	15,397	6,031	2,965
V2	65	17,376	4,704	14,883	5,607	3,099
V2	65	19,058	4,926	15,768	6,003	3,174
V2	65	18,852	4,899	15,631	5,917	3,186
V2	65	20,134	5,063	16,256	6,151	3,273
V2	65	18,114	4,802	15,544	6,018	3,010
V2	65	21,732	5,260	17,044	6,531	3,327
V2	65	18,767	4,888	15,768	6,112	3,070
V2	65	19,578	4,993	16,073	6,182	3,167
V2	65	22,373	5,337	16,994	6,368	3,513
V2	65	18,622	4,869	15,875	5,918	3,147
V2	65	17,787	4,759	15,595	6,171	2,882
V2	65	17,545	4,726	15,264	5,967	2,940
V2	65	18,525	4,857	15,681	6,147	3,014
V2	65	18,114	4,802	15,412	5,850	3,096
V2	65	17,134	4,671	14,980	5,918	2,895
V2	65	18,840	4,898	15,717	6,071	3,103
V2	65	20,110	5,060	16,271	6,053	3,322
V2	65	17,460	4,715	15,249	5,765	3,029
V2	65	19,457	4,977	15,961	6,006	3,240
V2	65	21,369	5,216	16,663	6,463	3,306
V2	65	20,122	5,062	16,160	6,164	3,265
V2	65	21,139	5,188	16,577	6,190	3,415
V2	65	26,342	5,791	18,570	6,809	3,868
V2	65	19,469	4,979	16,134	6,410	3,037
V2	65	17,799	4,761	15,325	5,904	3,015
V2	65	19,469	4,979	16,134	6,410	3,037
V2	65	17,799	4,761	15,325	5,904	3,015
V2	65	19,723	5,011	16,134	6,083	3,242
V2	65	17,085	4,664	14,857	5,743	2,975
V2	65	18,586	4,865	15,570	5,889	3,156
V2	65	18,259	4,822	15,447	5,994	3,046
V2	65	18,501	4,853	15,620	6,030	3,068
V2	65	19,179	4,942	15,803	6,030	3,180
V2	65	19,687	5,007	16,098	6,222	3,164
V2	65	18,900	4,906	15,829	6,017	3,141
V2	65	18,440	4,846	15,961	6,274	2,939
V2	65	16,396	4,569	14,883	5,715	2,869
V2	65	18,949	4,912	15,829	6,167	3,072
V2	65	17,182	4,677	15,091	5,904	2,910
V2	65	17,823	4,764	15,325	5,772	3,088
V2	65	19,070	4,927	15,717	6,064	3,145
V2	65	18,380	4,838	15,509	6,021	3,053
V2	65	18,428	4,844	15,570	5,807	3,174
V2	65	19,723	5,011	15,987	6,059	3,255
V2	65	18,392	4,839	15,447	5,872	3,132
V2	65	17,993	4,786	15,178	5,749	3,130
V2	65	18,791	4,891	15,865	6,042	3,110
V2	65	18,549	4,860	15,620	6,058	3,062
V2	65	19,300	4,957	15,829	5,871	3,287
V2	65	18,162	4,809	15,422	5,922	3,067

V2	65	18,453	4,847	15,447	5,818	3,171
V2	65	18,077	4,798	15,412	5,777	3,129
V2	65	19,493	4,982	15,728	5,683	3,430
V2	65	18,356	4,834	15,422	5,825	3,151
V2	65	19,239	4,949	15,742	6,011	3,200
V2	65	17,823	4,764	15,310	5,821	3,062
V2	65	18,368	4,836	15,422	5,814	3,159
V2	65	18,380	4,838	15,473	5,922	3,104
V2	65	18,053	4,794	15,544	5,971	3,023
V2	65	20,013	5,048	16,429	6,403	3,125
V2	65	18,900	4,906	15,753	6,062	3,118
V2	65	19,493	4,982	15,915	6,018	3,239
V2	65	16,553	4,591	14,735	5,711	2,898
V2	65	21,066	5,179	16,699	6,553	3,215
V2	65	18,138	4,806	15,351	5,814	3,120
V2	65	19,917	5,036	16,246	6,274	3,175
V2	65	19,179	4,942	16,185	6,168	3,109
V2	65	16,008	4,515	14,379	5,572	2,873
V2	65	17,642	4,739	15,310	5,793	3,046
V2	65	18,864	4,901	15,681	6,064	3,111
V2	65	19,517	4,985	16,109	5,977	3,266
V2	65	16,734	4,616	15,127	5,870	2,851
V2	65	18,090	4,799	15,336	5,899	3,066
V2	65	18,537	4,858	15,692	6,018	3,080
V2	65	17,630	4,738	15,203	5,724	3,080
V2	65	15,718	4,474	14,268	5,429	2,895
V2	65	18,997	4,918	15,890	6,184	3,072
V2	65	18,307	4,828	15,595	6,003	3,050
V2	65	18,549	4,860	15,681	6,032	3,075
V2	65	17,618	4,736	15,325	5,850	3,011
V2	65	16,964	4,648	15,056	5,865	2,893
V2	65	17,896	4,773	15,325	5,841	3,064
V2	65	17,122	4,669	15,178	5,967	2,869
V2	65	17,182	4,677	14,919	5,777	2,974
V2	65	18,755	4,887	15,793	6,087	3,081
V2	65	16,964	4,648	14,883	5,686	2,983
V2	65	18,586	4,865	15,509	5,786	3,212
V2	65	16,831	4,629	15,056	5,937	2,835
V2	65	18,440	4,846	15,544	5,918	3,116
V2	65	18,489	4,852	15,559	6,058	3,052
V2	65	19,675	5,005	16,048	6,162	3,193
V2	65	19,312	4,959	15,768	5,967	3,236
V2	65	17,606	4,735	15,523	6,011	2,929
V2	65	18,828	4,896	15,631	5,871	3,207
V2	65	20,848	5,152	16,490	6,264	3,329
V2	65	17,122	4,669	14,908	5,571	3,073
V2	65	19,384	4,968	15,926	6,096	3,180
V2	65	18,271	4,823	15,412	5,814	3,142
V2	65	17,775	4,757	15,203	5,871	3,027
V2	65	18,888	4,904	15,742	6,017	3,139
V2	65	17,593	4,733	15,091	5,748	3,061
V2	65	18,368	4,836	15,620	6,064	3,029

V2	65	20,340	5,089	16,221	6,217	3,272
V2	65	18,005	4,788	15,351	5,818	3,095
V2	65	20,425	5,100	16,566	6,094	3,352
V2	65	18,126	4,804	15,447	5,889	3,078
V2	65	19,142	4,937	15,803	5,915	3,236
V2	65	19,880	5,031	16,098	6,134	3,241
V2	65	17,206	4,681	14,883	5,645	3,048
V2	65	19,457	4,977	15,854	6,021	3,232
V2	65	23,389	5,457	17,497	6,083	3,845
V2	65	18,029	4,791	15,239	5,731	3,146
V2	65	18,537	4,858	15,595	5,880	3,153
V2	65	19,505	4,983	15,803	5,922	3,293
V2	65	17,206	4,681	14,883	5,645	3,048
V2	65	19,457	4,977	15,854	6,021	3,232
V2	65	18,029	4,791	15,239	5,731	3,146
V2	65	18,537	4,858	15,595	5,880	3,153
V2	65	19,505	4,983	15,803	5,922	3,293
V2	65	18,828	4,896	15,656	6,021	3,127
V2	65	18,017	4,790	15,239	5,962	3,022
V2	65	20,800	5,146	16,739	6,264	3,321
V2	65	19,699	5,008	16,246	6,391	3,082
V2	65	18,828	4,896	15,656	6,021	3,127
V2	65	18,017	4,790	15,239	5,962	3,022
V2	65	19,699	5,008	16,246	6,391	3,082
V2	65	17,315	4,695	14,969	5,677	3,050
V2	80	20,280	5,081	16,149	5,936	3,416
V2	80	20,255	5,078	16,149	5,936	3,412
V1	80	20,171	5,068	16,073	5,936	3,398
V1	80	19,542	4,988	15,829	5,936	3,292
V1	80	19,130	4,935	15,768	5,936	3,223
V2	80	19,058	4,926	15,656	5,936	3,210
V1	80	18,924	4,909	15,707	5,936	3,188
V1	80	20,764	5,142	16,185	5,937	3,497
V2	80	20,485	5,107	16,297	5,937	3,451
V1	80	19,844	5,027	16,149	5,937	3,343
V1	80	19,179	4,942	15,829	5,937	3,230
V1	80	19,045	4,924	15,768	5,937	3,208
V2	80	20,219	5,074	16,037	5,937	3,405
V2	80	20,159	5,066	16,073	5,937	3,395
V1	80	19,832	5,025	15,976	5,937	3,340
V2	80	19,747	5,014	15,915	5,937	3,326
V1	80	19,154	4,938	15,768	5,937	3,226
V1	80	18,864	4,901	15,681	5,937	3,177
V1	80	20,062	5,054	16,134	5,940	3,377
V1	80	20,038	5,051	16,124	5,940	3,373
V2	80	19,421	4,973	15,829	5,940	3,269
V1	80	19,287	4,956	15,742	5,940	3,247
V2	80	19,542	4,988	15,890	5,940	3,290
V2	80	19,542	4,988	15,890	5,940	3,290
V1	80	19,348	4,963	15,890	5,940	3,257
V2	80	19,179	4,942	15,742	5,940	3,229
V1	80	19,808	5,022	15,951	5,940	3,334

V1	80	19,142	4,937	15,829	5,940	3,222
V1	80	20,618	5,124	16,343	5,953	3,463
V2	80	19,191	4,943	15,803	5,953	3,224
V2	80	19,989	5,045	16,134	5,955	3,357
V1	80	19,312	4,959	15,717	5,955	3,243
V1	80	19,227	4,948	15,778	5,955	3,229
V2	80	19,130	4,935	15,656	5,955	3,212
V2	80	18,937	4,910	15,656	5,955	3,180
V1	80	18,767	4,888	15,570	5,955	3,152
V1	80	18,610	4,868	15,595	5,955	3,125
V1	80	18,368	4,836	15,509	5,955	3,084
V1	80	19,493	4,982	15,829	5,955	3,273
V1	80	19,529	4,987	15,865	5,956	3,279
V1	80	19,493	4,982	15,865	5,956	3,273
V2	80	19,082	4,929	15,717	5,956	3,204
V1	80	20,582	5,119	16,246	5,956	3,456
V2	80	19,905	5,034	16,012	5,956	3,342
V1	80	19,784	5,019	16,073	5,956	3,322
V1	80	19,711	5,010	15,951	5,956	3,310
V1	80	19,493	4,982	15,997	5,956	3,273
V2	80	19,421	4,973	15,890	5,956	3,261
V2	80	19,179	4,942	15,926	5,956	3,220
V2	80	19,166	4,940	15,717	5,956	3,218
V1	80	19,070	4,927	15,803	5,956	3,202
V1	80	20,497	5,109	16,098	5,956	3,442
V1	80	19,977	5,043	15,987	5,956	3,354
V1	80	18,864	4,901	15,717	5,956	3,167
V2	80	20,292	5,083	16,037	5,962	3,404
V1	80	18,465	4,849	15,534	5,962	3,097
V2	80	19,965	5,042	16,012	5,963	3,348
V1	80	20,171	5,068	16,037	5,964	3,382
V1	80	19,784	5,019	15,976	5,964	3,317
V2	80	19,711	5,010	15,890	5,964	3,305
V1	80	19,590	4,994	15,915	5,964	3,284
V1	80	19,408	4,971	15,768	5,964	3,254
V1	80	19,130	4,935	15,656	5,964	3,207
V1	80	19,118	4,934	15,681	5,964	3,205
V1	80	19,106	4,932	15,803	5,964	3,203
V2	80	18,223	4,817	15,422	5,964	3,055
V1	80	20,437	5,101	16,037	5,965	3,426
V1	80	19,663	5,004	15,778	5,965	3,296
V2	80	19,372	4,966	15,768	5,965	3,248
V1	80	19,009	4,920	15,717	5,965	3,187
V1	80	18,961	4,913	15,707	5,965	3,179
V1	80	20,727	5,137	16,246	5,967	3,474
V1	80	20,582	5,119	16,221	5,967	3,449
V1	80	20,304	5,084	16,149	5,967	3,403
V1	80	19,796	5,020	15,951	5,967	3,318
V2	80	19,699	5,008	15,976	5,967	3,301
V1	80	19,227	4,948	15,890	5,967	3,222
V2	80	19,118	4,934	15,854	5,967	3,204
V1	80	19,070	4,927	15,768	5,967	3,196

V2	80	18,937	4,910	15,707	5,967	3,174
V2	80	19,433	4,974	15,879	5,967	3,257
V2	80	20,727	5,137	16,185	5,967	3,473
V2	80	19,421	4,973	15,829	5,967	3,254
V2	80	19,142	4,937	15,793	5,967	3,208
V2	80	18,816	4,895	15,681	5,967	3,153
V1	80	21,502	5,232	17,177	5,971	3,501
V1	80	20,945	5,164	16,271	5,971	3,508
V2	80	19,929	5,037	15,890	5,971	3,337
V2	80	19,892	5,033	15,951	5,971	3,331
V1	80	19,796	5,020	15,865	5,971	3,315
V2	80	19,784	5,019	15,839	5,971	3,313
V1	80	19,614	4,997	15,879	5,971	3,285
V2	80	19,566	4,991	15,829	5,971	3,277
V2	80	19,421	4,973	15,803	5,971	3,252
V1	80	19,239	4,949	15,742	5,971	3,222
V2	80	19,106	4,932	15,717	5,971	3,200
V1	80	18,682	4,877	15,803	5,971	3,129
V2	80	20,062	5,054	16,048	5,971	3,360
V2	80	20,026	5,049	15,926	5,971	3,354
V1	80	19,856	5,028	15,987	5,971	3,325
V2	80	19,796	5,020	15,951	5,971	3,315
V2	80	19,045	4,924	15,875	5,971	3,189
V2	80	18,961	4,913	15,717	5,971	3,175
V1	80	18,561	4,861	15,534	5,971	3,108
V2	80	19,759	5,016	15,951	5,972	3,309
V1	80	19,723	5,011	15,803	5,972	3,303
V1	80	20,800	5,146	16,282	5,977	3,480
V2	80	20,376	5,094	16,195	5,977	3,409
V1	80	20,292	5,083	16,134	5,977	3,395
V1	80	20,122	5,062	16,170	5,977	3,367
V2	80	19,517	4,985	15,987	5,977	3,266
V2	80	19,106	4,932	15,875	5,977	3,197
V1	80	19,009	4,920	15,742	5,977	3,181
V1	80	20,618	5,124	16,160	5,977	3,450
V1	80	19,336	4,962	15,803	5,977	3,235
V1	80	20,921	5,161	16,282	5,977	3,500
V1	80	20,522	5,112	16,256	5,977	3,434
V1	80	20,001	5,046	16,206	5,977	3,347
V1	80	19,626	4,999	15,987	5,977	3,284
V1	80	19,626	4,999	15,987	5,977	3,284
V2	80	20,026	5,049	15,976	5,977	3,350
V2	80	19,638	5,000	15,976	5,977	3,286
V1	80	19,215	4,946	15,829	5,977	3,215
V1	80	17,993	4,786	15,386	5,977	3,010
V2	80	20,631	5,125	16,368	5,977	3,452
V1	80	20,510	5,110	16,343	5,977	3,431
V2	80	19,796	5,020	16,048	5,977	3,312
V1	80	19,663	5,004	15,987	5,977	3,290
V1	80	19,663	5,004	15,987	5,977	3,290
V2	80	19,917	5,036	15,976	5,977	3,332
V2	80	19,820	5,023	15,976	5,977	3,316

V2	80	21,659	5,251	16,653	5,982	3,521
V1	80	19,227	4,948	15,803	5,982	3,214
V1	80	19,203	4,945	15,976	5,982	3,210
V1	80	18,864	4,901	15,717	5,982	3,153
V2	80	19,784	5,019	16,063	5,982	3,307
V2	80	20,062	5,054	16,098	5,982	3,353
V2	80	19,626	4,999	16,037	5,982	3,281
V2	80	21,175	5,192	16,429	5,984	3,538
V1	80	20,437	5,101	16,160	5,984	3,415
V2	80	19,880	5,031	15,987	5,984	3,322
V2	80	19,481	4,980	15,926	5,984	3,255
V1	80	19,433	4,974	15,865	5,984	3,247
V2	80	19,421	4,973	15,865	5,984	3,245
V1	80	19,203	4,945	15,865	5,984	3,209
V2	80	19,275	4,954	15,839	5,984	3,221
V1	80	18,755	4,887	15,753	5,984	3,134
V2	80	20,727	5,137	16,282	5,985	3,463
V2	80	20,497	5,109	16,195	5,985	3,425
V2	80	21,078	5,181	16,393	5,993	3,517
V1	80	20,679	5,131	16,221	5,993	3,451
V2	80	19,759	5,016	15,900	5,993	3,297
V2	80	19,614	4,997	15,865	5,993	3,273
V2	80	18,791	4,891	15,717	5,993	3,136
V1	80	18,271	4,823	15,447	5,993	3,049
V2	80	20,945	5,164	16,393	5,993	3,495
V1	80	20,812	5,148	16,393	5,993	3,473
V2	80	19,868	5,030	16,012	5,993	3,315
V2	80	19,735	5,013	15,940	5,993	3,293
Control	Control	20,873	5,155	16,490	6,062	3,443
Control	Control	18,852	4,899	15,534	5,772	3,266
Control	Control	19,203	4,945	15,839	5,927	3,240
Control	Control	18,997	4,918	15,595	5,927	3,205
Control	Control	18,961	4,913	15,570	5,852	3,240
Control	Control	19,287	4,956	15,681	5,787	3,333
Control	Control	19,070	4,927	15,656	5,821	3,276
Control	Control	20,195	5,071	16,480	5,915	3,414
Control	Control	19,239	4,949	16,037	6,247	3,080
Control	Control	18,658	4,874	15,534	5,814	3,209
Control	Control	17,884	4,772	15,336	5,793	3,087
Control	Control	17,642	4,739	14,995	5,542	3,183
Control	Control	18,610	4,868	15,483	5,917	3,145
Control	Control	19,021	4,921	15,742	6,030	3,154
Control	Control	18,658	4,874	15,386	5,691	3,279
Control	Control	19,856	5,028	15,976	5,877	3,379
Control	Control	18,380	4,838	15,447	5,629	3,265
Control	Control	20,147	5,065	16,124	5,982	3,368
Control	Control	19,759	5,016	15,865	5,922	3,336
Control	Control	18,247	4,820	15,829	6,182	2,951
Control	Control	18,900	4,906	15,570	5,686	3,324
Control	Control	20,582	5,119	16,343	6,140	3,352
Control	Control	17,279	4,690	14,944	5,634	3,067
Control	Control	18,465	4,849	15,386	5,825	3,170

Control	Control	16,892	4,638	15,346	5,918	2,854
Control	Control	20,292	5,083	16,160	5,937	3,418
Control	Control	19,469	4,979	15,961	6,030	3,228
Control	Control	18,404	4,841	15,595	5,841	3,151
Control	Control	18,077	4,798	15,300	5,749	3,145
Control	Control	20,909	5,160	16,602	6,222	3,360
Control	Control	20,195	5,071	16,393	6,182	3,267
Control	Control	21,478	5,229	16,958	6,580	3,264
Control	Control	18,779	4,890	15,386	5,552	3,382
Control	Control	18,392	4,839	15,447	5,814	3,163
Control	Control	18,731	4,884	15,447	5,743	3,261
Control	Control	19,106	4,932	15,681	5,692	3,357
Control	Control	20,848	5,152	16,368	6,172	3,378
Control	Control	21,139	5,188	16,541	6,277	3,367
Control	Control	19,082	4,929	15,595	5,890	3,240
Control	Control	18,755	4,887	15,559	5,732	3,272
Control	Control	20,582	5,119	16,098	6,021	3,418
Control	Control	19,614	4,997	16,012	5,977	3,281
Control	Control	18,489	4,852	15,300	5,826	3,174
Control	Control	18,223	4,817	15,203	5,486	3,322
Control	Control	18,561	4,861	15,422	5,889	3,152
Control	Control	19,263	4,952	15,865	5,993	3,214
Control	Control	20,304	5,084	16,098	5,956	3,409
Control	Control	17,182	4,677	14,908	5,508	3,120
Control	Control	20,038	5,051	16,098	6,072	3,300
Control	Control	18,719	4,882	15,483	5,743	3,259
Control	Control	18,646	4,872	15,753	5,899	3,161
Control	Control	19,045	4,924	15,707	5,827	3,268
Control	Control	19,251	4,951	15,656	5,827	3,304
Control	Control	19,699	5,008	16,073	6,199	3,178
Control	Control	22,458	5,347	17,350	6,607	3,399
Control	Control	20,413	5,098	16,271	6,066	3,365
Control	Control	19,094	4,931	15,595	5,872	3,252
Control	Control	18,283	4,825	15,473	5,911	3,093
Control	Control	17,751	4,754	15,163	5,719	3,104
Control	Control	19,614	4,997	15,976	6,032	3,252
Control	Control	17,775	4,757	15,152	5,819	3,055
Control	Control	16,940	4,644	14,674	5,284	3,206
Control	Control	18,065	4,796	15,386	5,827	3,100
Control	Control	20,981	5,169	16,541	6,211	3,378
Control	Control	20,739	5,139	16,455	6,275	3,305
Control	Control	17,291	4,692	14,969	5,676	3,046
Control	Control	18,404	4,841	15,519	5,748	3,202
Control	Control	18,380	4,838	15,473	5,904	3,113
Control	Control	20,376	5,094	16,134	6,011	3,390
Control	Control	18,755	4,887	15,422	5,772	3,249
Control	Control	19,058	4,926	15,681	5,867	3,248
Control	Control	20,800	5,146	16,404	5,977	3,480
Control	Control	20,001	5,046	16,195	5,977	3,347
Control	Control	19,929	5,037	15,839	5,598	3,560
Control	Control	18,949	4,912	15,707	5,977	3,170
Control	Control	19,929	5,037	15,890	5,877	3,391

Control	Control	19,130	4,935	15,681	5,786	3,306
Control	Control	21,139	5,188	16,516	6,140	3,443
Control	Control	21,937	5,285	17,070	6,508	3,371
Control	Control	20,389	5,095	16,124	5,882	3,466
Control	Control	20,280	5,081	16,160	5,977	3,393
Control	Control	19,711	5,010	15,951	6,021	3,274
Control	Control	20,183	5,069	16,160	6,197	3,257
Control	Control	18,247	4,820	15,336	5,581	3,270
Control	Control	18,658	4,874	15,778	5,870	3,178
Control	Control	20,062	5,054	16,185	6,004	3,342
Control	Control	18,949	4,912	15,534	5,827	3,252
Control	Control	19,372	4,966	15,865	5,870	3,300
Control	Control	19,493	4,982	15,803	5,918	3,294
Control	Control	19,300	4,957	15,803	5,984	3,225
Control	Control	18,695	4,879	15,666	5,821	3,212
Control	Control	19,542	4,988	15,951	5,915	3,304
Control	Control	19,844	5,027	16,063	6,112	3,246
Control	Control	18,622	4,869	15,620	5,937	3,137
Control	Control	18,271	4,823	15,544	5,776	3,163
Control	Control	18,864	4,901	15,803	5,985	3,152
Control	Control	19,275	4,954	15,803	5,977	3,225
Control	Control	20,050	5,053	16,048	5,880	3,410
Control	Control	18,501	4,853	15,544	5,658	3,270
Control	Control	20,255	5,078	16,134	5,971	3,392
Control	Control	18,102	4,801	15,447	5,697	3,178
Control	Control	19,215	4,946	15,692	5,852	3,284
V1	50	18,840	4,898	15,620	5,971	3,155
V1	50	17,593	4,733	14,944	5,473	3,214
V1	50	19,699	5,008	15,829	5,841	3,372
V1	50	18,404	4,841	15,325	5,765	3,192
V1	50	18,852	4,899	15,620	5,672	3,323
V1	50	19,324	4,960	15,839	5,927	3,260
V1	50	25,422	5,689	18,332	6,262	4,059
V1	50	20,255	5,078	15,951	5,917	3,423
V1	50	19,517	4,985	15,961	5,815	3,357
V1	50	21,175	5,192	16,455	6,134	3,452
V1	50	19,542	4,988	15,839	5,871	3,329
V1	50	19,324	4,960	15,839	5,927	3,260
V1	50	20,255	5,078	15,951	5,917	3,423
V1	50	19,517	4,985	15,961	5,815	3,357
V1	50	21,175	5,192	16,455	6,134	3,452
V1	50	19,542	4,988	15,839	5,871	3,329
V1	50	18,997	4,918	15,717	6,011	3,160
V1	50	19,227	4,948	15,829	5,940	3,237
V1	50	19,251	4,951	15,865	5,807	3,315
V1	50	18,525	4,857	15,386	5,719	3,239
V1	50	20,098	5,059	16,221	6,005	3,347
V1	50	20,812	5,148	16,455	6,083	3,421
V1	50	18,453	4,847	15,666	6,017	3,067
V1	50	18,198	4,814	15,275	5,630	3,232
V1	50	15,004	4,371	14,034	5,119	2,931
V1	50	19,106	4,932	15,666	5,772	3,310

V1	50	18,126	4,804	15,336	5,715	3,172
V1	50	20,352	5,091	16,195	5,956	3,417
V1	50	21,623	5,247	16,994	6,237	3,467
V1	50	21,478	5,229	16,933	6,264	3,429
V1	50	19,844	5,027	16,221	6,003	3,306
V1	50	18,126	4,804	15,361	5,826	3,111
V1	50	18,150	4,807	15,178	5,582	3,252
V1	50	18,658	4,874	15,534	5,793	3,221
V1	50	20,485	5,107	16,073	5,977	3,428
V1	50	18,997	4,918	15,534	5,607	3,388
V1	50	20,594	5,121	16,404	6,034	3,413
V1	50	20,824	5,149	16,455	6,211	3,353
V1	50	18,719	4,882	15,544	5,523	3,389
V1	50	19,517	4,985	15,829	5,927	3,293
V1	50	18,816	4,895	15,839	6,005	3,133
V1	50	19,614	4,997	15,753	5,609	3,497
V1	50	19,058	4,926	15,544	5,645	3,376
V1	50	19,856	5,028	15,987	5,918	3,355
V1	50	20,183	5,069	16,073	5,871	3,438
V1	50	20,098	5,059	15,951	5,956	3,375
V1	50	20,643	5,127	16,332	6,128	3,369
V1	50	18,477	4,850	15,361	5,674	3,257
V1	50	19,348	4,963	15,890	5,940	3,257
V1	50	20,546	5,115	16,393	6,137	3,348
V1	50	17,388	4,705	15,285	5,717	3,042
V1	50	18,924	4,909	15,742	5,850	3,235
V1	50	19,348	4,963	15,839	5,927	3,264
V1	50	18,138	4,806	15,249	5,552	3,267
V1	50	18,561	4,861	15,483	5,825	3,187
V1	50	19,905	5,034	15,890	5,882	3,384
V1	50	20,086	5,057	16,383	6,247	3,215
V1	50	19,941	5,039	15,951	6,018	3,314
V1	50	19,711	5,010	16,073	6,164	3,198
V1	50	19,784	5,019	16,098	6,200	3,191
V1	50	20,425	5,100	16,343	5,977	3,417
V1	50	22,119	5,307	17,253	6,463	3,422
V1	50	19,408	4,971	15,890	6,018	3,225
V1	50	20,074	5,056	15,976	5,977	3,359
V1	50	19,227	4,948	15,778	5,793	3,319
V1	50	19,880	5,031	16,124	5,994	3,317
V1	50	20,183	5,069	16,098	6,118	3,299
V1	50	19,820	5,023	16,073	5,788	3,424
V1	50	18,174	4,810	15,336	5,766	3,152
V1	50	23,280	5,444	17,533	6,550	3,554
V1	50	20,558	5,116	16,271	5,967	3,445
V1	50	18,695	4,879	15,473	5,661	3,303
V1	50	16,855	4,633	15,045	5,519	3,054
V1	50	23,643	5,487	17,487	6,428	3,678
V1	50	19,602	4,996	16,246	6,391	3,067
V1	50	20,401	5,097	16,455	6,151	3,317
V1	50	19,578	4,993	15,890	6,017	3,254
V1	50	19,058	4,926	15,681	5,629	3,385

V1	50	22,131	5,308	17,289	6,523	3,393
V1	50	18,949	4,912	15,595	5,474	3,461
V1	50	19,130	4,935	15,717	5,793	3,303
V1	50	19,554	4,990	15,890	5,882	3,324
V1	50	20,376	5,094	16,282	6,032	3,378
V1	50	21,828	5,272	16,872	6,279	3,477
V1	50	20,534	5,113	16,699	6,604	3,110
V1	50	17,872	4,770	15,152	5,786	3,089
V1	50	18,816	4,895	16,048	6,005	3,133
V1	50	19,602	4,996	16,098	6,057	3,236
V1	50	19,759	5,016	15,926	5,962	3,314
V1	50	20,860	5,154	16,419	5,965	3,497
V1	50	20,897	5,158	16,541	6,456	3,237
V1	50	19,796	5,020	16,012	5,867	3,374
V1	50	21,042	5,176	16,541	6,274	3,354
V1	50	18,017	4,790	15,473	5,731	3,144
V1	50	19,481	4,980	15,768	5,867	3,321
V1	50	19,408	4,971	15,681	5,676	3,419
V1	50	19,699	5,008	15,768	5,311	3,709
V1	50	18,816	4,895	15,559	5,691	3,306
V1	50	17,557	4,728	15,214	5,608	3,131
V1	50	18,719	4,882	15,620	6,018	3,111
V1	50	18,864	4,901	15,656	5,852	3,224
V1	50	16,783	4,623	14,761	5,580	3,008
V1	50	17,230	4,684	14,710	5,427	3,175
V1	50	18,586	4,865	15,458	5,715	3,252
V1	50	17,485	4,718	15,152	5,629	3,106
V1	50	19,614	4,997	16,160	6,164	3,182
V1	50	18,126	4,804	15,336	5,666	3,199
V1	50	18,211	4,815	15,509	5,786	3,147
V1	50	19,287	4,956	15,951	6,096	3,164
V1	50	19,493	4,982	15,829	5,962	3,269
V1	50	19,747	5,014	16,109	6,042	3,268
V1	50	19,251	4,951	15,717	5,850	3,291
V1	50	19,239	4,949	15,778	5,845	3,292
V1	50	19,844	5,027	15,951	5,927	3,348
V1	50	17,739	4,752	15,030	5,772	3,073
V1	50	18,840	4,898	15,534	5,691	3,310
V1	50	17,460	4,715	15,030	5,544	3,149
V1	50	19,675	5,005	16,465	6,613	2,975
V1	50	17,981	4,785	15,264	5,697	3,156
V1	50	19,384	4,968	15,732	5,661	3,424
V1	50	18,924	4,909	15,509	5,819	3,252
V1	50	20,062	5,054	16,134	6,162	3,256
V1	50	19,396	4,970	15,839	5,890	3,293
V1	50	18,924	4,909	15,620	5,867	3,226
V1	50	19,396	4,970	15,717	5,890	3,293
V1	50	20,389	5,095	16,134	6,096	3,345
V1	50	19,578	4,993	15,778	5,852	3,345
V1	50	20,243	5,077	16,124	5,877	3,445
V1	50	19,832	5,025	16,048	5,956	3,330
V1	50	19,457	4,977	15,890	6,017	3,234

V1	50	20,183	5,069	16,012	5,956	3,389
V1	50	17,303	4,694	14,980	5,748	3,010
V1	50	21,841	5,273	16,882	6,168	3,541
V1	50	18,707	4,880	15,717	5,934	3,152
V1	50	19,614	4,997	15,926	5,818	3,371
V1	50	20,134	5,063	16,073	5,913	3,405
V1	50	20,122	5,062	16,134	6,091	3,304
V1	65	18,779	4,890	15,742	6,057	3,100
V1	65	21,139	5,188	16,775	6,376	3,315
V1	65	20,873	5,155	16,393	6,210	3,361
V1	65	19,372	4,966	15,890	5,977	3,241
V1	65	21,006	5,172	16,551	5,977	3,515
V1	65	20,897	5,158	16,429	6,112	3,419
V1	65	22,095	5,304	16,872	6,336	3,487
V1	65	20,195	5,071	16,185	5,994	3,369
V1	65	19,445	4,976	15,768	5,786	3,360
V1	65	20,304	5,084	16,073	5,984	3,393
V1	65	19,251	4,951	15,570	5,776	3,333
V1	65	18,888	4,904	15,498	5,827	3,241
V1	65	20,582	5,119	16,271	5,904	3,486
V1	65	19,287	4,956	15,839	5,793	3,329
V1	65	19,844	5,027	16,195	6,162	3,220
V1	65	20,510	5,110	16,297	6,087	3,370
V1	65	19,856	5,028	16,073	6,275	3,165
V1	65	18,428	4,844	15,519	5,852	3,149
V1	65	18,319	4,830	15,422	5,744	3,189
V1	65	18,634	4,871	15,473	5,827	3,198
V1	65	19,408	4,971	15,865	5,927	3,274
V1	65	19,336	4,962	15,915	5,937	3,257
V1	65	18,392	4,839	15,498	5,786	3,178
V1	65	20,280	5,081	16,134	6,018	3,370
V1	65	18,888	4,904	15,620	5,882	3,211
V1	65	19,965	5,042	16,282	6,170	3,236
V1	65	17,969	4,783	15,656	6,042	2,974
V1	65	21,429	5,223	16,724	6,264	3,421
V1	65	19,505	4,983	15,890	6,032	3,233
V1	65	21,248	5,201	16,699	6,006	3,538
V1	65	18,670	4,876	15,534	5,772	3,234
V1	65	19,820	5,023	16,195	6,414	3,090
V1	65	21,841	5,273	17,044	6,754	3,234
V1	65	19,215	4,946	15,829	5,850	3,284
V1	65	20,497	5,109	16,221	5,867	3,494
V1	65	19,977	5,043	16,185	6,157	3,245
V1	65	17,206	4,681	15,005	5,661	3,040
V1	65	21,211	5,197	16,490	6,168	3,439
V1	65	19,094	4,931	15,839	6,032	3,165
V1	65	18,150	4,807	15,386	5,814	3,122
V1	65	18,973	4,915	15,926	5,917	3,206
V1	65	19,626	4,999	15,976	5,994	3,274
V1	65	19,638	5,000	15,940	6,017	3,264
V1	65	22,857	5,395	17,411	6,503	3,515
V1	65	21,102	5,183	16,566	6,183	3,413

V1	65	19,166	4,940	15,803	5,977	3,207
V1	65	19,094	4,931	15,768	5,850	3,264
V1	65	18,150	4,807	15,361	5,743	3,160
V1	65	20,715	5,136	16,480	6,183	3,350
V1	65	20,994	5,170	16,480	6,067	3,461
V1	65	21,163	5,191	16,663	6,368	3,323
V1	65	19,578	4,993	15,976	6,031	3,246
V1	65	19,917	5,036	15,926	5,917	3,366
V1	65	20,933	5,163	16,455	6,157	3,400
V1	65	21,030	5,175	16,846	6,487	3,242
V1	65	20,086	5,057	16,160	6,217	3,231
V1	65	20,739	5,139	16,393	6,164	3,365
V1	65	21,066	5,179	16,480	6,032	3,493
V1	65	15,403	4,429	14,293	5,661	2,721
V1	65	19,287	4,956	15,656	5,814	3,317
V1	65	20,461	5,104	16,332	6,211	3,295
V1	65	19,917	5,036	15,865	6,018	3,310
V1	65	18,138	4,806	15,336	5,717	3,172
V1	65	20,159	5,066	16,332	6,318	3,191
V1	65	19,699	5,008	15,987	6,059	3,251
V1	65	19,905	5,034	16,170	6,021	3,306
V1	65	20,268	5,080	16,149	5,964	3,398
V1	65	18,489	4,852	15,534	5,636	3,280
V1	65	19,142	4,937	15,839	6,111	3,133
V1	65	19,566	4,991	15,900	6,167	3,173
V1	65	18,682	4,877	15,620	5,904	3,164
V1	65	19,421	4,973	15,976	6,121	3,173
V1	65	20,147	5,065	16,124	6,073	3,318
V1	65	18,198	4,814	15,447	5,826	3,124
V1	65	18,912	4,907	15,966	5,872	3,221
V1	65	20,134	5,063	16,073	5,845	3,445
V1	65	22,494	5,352	17,080	6,310	3,565
V1	65	20,364	5,092	16,246	6,147	3,313
V1	65	21,913	5,282	16,749	6,310	3,473
V1	65	20,764	5,142	16,455	6,332	3,279
V1	65	18,235	4,818	15,534	5,918	3,081
V1	65	20,219	5,074	16,221	6,091	3,320
V1	65	20,582	5,119	16,318	6,200	3,320
V1	65	19,953	5,040	16,195	6,083	3,280
V1	65	20,026	5,049	16,170	5,899	3,395
V1	65	21,151	5,189	16,577	6,303	3,356
V1	65	22,252	5,323	17,141	6,495	3,426
V1	65	20,945	5,164	16,429	6,168	3,396
V1	65	20,909	5,160	16,404	5,821	3,592
V1	65	19,844	5,027	16,073	6,005	3,305
V1	65	18,743	4,885	15,875	6,005	3,121
V1	65	21,659	5,251	16,749	6,246	3,467
V1	65	20,074	5,056	16,098	6,059	3,313
V1	65	20,558	5,116	16,358	6,094	3,374
V1	65	22,131	5,308	17,375	6,264	3,533
V1	65	19,336	4,962	15,987	6,062	3,190
V1	65	20,800	5,146	16,465	5,934	3,505

V1	65	18,803	4,893	15,570	5,486	3,427
V1	65	19,832	5,025	16,098	6,067	3,269
V1	65	24,164	5,547	17,523	6,563	3,682
V1	65	21,683	5,254	16,541	5,985	3,623
V1	65	21,647	5,250	16,724	6,237	3,471
V1	65	21,744	5,262	16,882	6,274	3,465
V1	65	19,154	4,938	15,839	5,928	3,231
V1	65	19,469	4,979	15,890	6,003	3,243
V1	65	22,675	5,373	17,080	6,482	3,498
V1	65	20,328	5,087	16,037	5,928	3,429
V1	65	19,711	5,010	16,012	5,964	3,305
V1	65	22,010	5,294	17,030	6,331	3,476
V1	65	18,561	4,861	15,483	5,793	3,204
V1	65	19,566	4,991	15,717	5,852	3,343
V1	65	21,659	5,251	16,663	6,356	3,407
V1	65	21,732	5,260	16,760	6,301	3,449
V1	65	20,921	5,161	16,490	6,124	3,416
V1	65	18,949	4,912	15,681	5,882	3,221
V1	65	20,183	5,069	16,160	6,124	3,296
V1	65	18,707	4,880	15,447	5,621	3,328
V1	65	18,307	4,828	15,473	5,760	3,178
V1	65	21,042	5,176	16,638	6,189	3,400
V1	65	18,356	4,834	15,422	5,822	3,153
V1	65	20,171	5,068	16,134	6,117	3,297
V1	65	20,776	5,143	16,516	6,111	3,399
V1	65	19,094	4,931	15,839	5,764	3,312
V1	65	19,408	4,971	15,915	6,032	3,218
V1	65	15,887	4,498	14,440	5,542	2,867
V1	65	18,755	4,887	15,498	5,661	3,313
V1	65	20,691	5,133	16,368	6,140	3,370
V1	65	16,867	4,634	14,563	5,380	3,135
V1	65	20,352	5,091	16,419	6,363	3,198
V1	65	20,631	5,125	16,419	6,217	3,318
V1	65	20,074	5,056	16,098	6,073	3,306
V1	65	18,198	4,814	15,483	5,814	3,130
V1	65	18,973	4,915	15,793	5,787	3,279
V1	65	18,561	4,861	15,595	5,877	3,158
V1	65	20,110	5,060	16,221	6,310	3,187
V1	65	20,207	5,072	16,073	6,096	3,315
V1	65	18,053	4,794	15,386	5,882	3,069
V1	65	18,053	4,794	15,264	5,692	3,172
V1	65	19,021	4,921	15,829	5,977	3,182
V1	65	17,848	4,767	15,275	5,638	3,166
V1	65	18,900	4,906	15,818	6,032	3,134
V1	65	18,186	4,812	15,300	5,676	3,204
V1	65	18,549	4,860	15,509	5,826	3,184
V1	65	18,622	4,869	15,620	6,018	3,094
V1	65	17,533	4,725	15,188	5,673	3,090
V1	65	19,965	5,042	16,221	6,111	3,267
V1	65	18,755	4,887	15,865	6,199	3,025
V1	65	19,892	5,033	16,246	6,238	3,189
V1	65	17,315	4,695	14,883	5,673	3,052

V1	65	18,670	4,876	15,483	5,609	3,329
V1	65	18,816	4,895	15,915	6,094	3,088
V1	65	17,751	4,754	15,127	5,717	3,105
V2	80	19,517	4,985	15,900	5,993	3,257
V1	80	19,372	4,966	15,900	5,993	3,233
V1	80	19,191	4,943	15,829	5,993	3,202
V1	80	20,812	5,148	16,368	5,993	3,473
V2	80	20,522	5,112	16,124	5,993	3,424
V2	80	20,171	5,068	16,012	5,993	3,366
V2	80	20,001	5,046	16,023	5,993	3,337
V2	80	19,445	4,976	15,778	5,993	3,244
V2	80	19,009	4,920	15,753	5,993	3,172
V2	80	21,078	5,181	16,861	5,994	3,517
V2	80	20,461	5,104	16,231	5,994	3,414
V2	80	20,050	5,053	16,124	5,994	3,345
V2	80	19,747	5,014	16,037	5,994	3,294
V2	80	19,529	4,987	16,002	5,994	3,258
V2	80	19,215	4,946	15,803	5,994	3,206
V2	80	19,070	4,927	15,768	5,994	3,181
V1	80	19,796	5,020	15,951	5,994	3,302
V1	80	20,909	5,160	16,490	5,995	3,488
V1	80	20,243	5,077	16,185	6,003	3,372
V1	80	20,219	5,074	16,012	6,003	3,368
V1	80	20,062	5,054	16,098	6,003	3,342
V1	80	19,905	5,034	16,063	6,003	3,316
V1	80	19,287	4,956	15,976	6,003	3,213
V2	80	19,166	4,940	15,717	6,003	3,193
V1	80	20,122	5,062	16,073	6,003	3,352
V2	80	19,953	5,040	15,976	6,004	3,324
V1	80	21,296	5,207	16,455	6,005	3,546
V2	80	20,558	5,116	16,246	6,005	3,423
V2	80	20,352	5,091	16,256	6,005	3,389
V2	80	20,147	5,065	16,195	6,005	3,355
V1	80	19,917	5,036	16,048	6,005	3,317
V2	80	19,820	5,023	16,012	6,005	3,301
V2	80	19,699	5,008	16,012	6,005	3,280
V2	80	19,602	4,996	16,012	6,005	3,264
V1	80	20,086	5,057	16,134	6,005	3,345
V1	80	20,001	5,046	16,134	6,005	3,331
V1	80	19,699	5,008	16,073	6,005	3,280
V1	80	19,384	4,968	16,023	6,005	3,228
V1	80	21,998	5,292	16,775	6,006	3,663
V2	80	21,841	5,273	16,749	6,006	3,537
V2	80	20,497	5,109	16,343	6,006	3,413
V1	80	20,413	5,098	16,195	6,006	3,399
V1	80	20,219	5,074	16,195	6,006	3,367
V1	80	20,183	5,069	16,134	6,006	3,361
V1	80	19,977	5,043	16,073	6,006	3,326
V2	80	19,759	5,016	15,926	6,006	3,290
V1	80	20,050	5,053	15,951	6,011	3,336
V2	80	19,493	4,982	15,951	6,011	3,243
V2	80	19,154	4,938	15,803	6,011	3,187

V1	80	18,549	4,860	15,534	6,011	3,086
V2	80	20,497	5,109	16,246	6,011	3,410
V2	80	20,110	5,060	16,063	6,011	3,345
V2	80	19,033	4,923	15,768	6,011	3,166
V2	80	19,735	5,013	15,951	6,011	3,283
V2	80	19,735	5,013	15,951	6,011	3,283
V1	80	19,566	4,991	15,854	6,011	3,255
V1	80	20,691	5,133	16,358	6,017	3,439
V2	80	19,154	4,938	15,793	6,017	3,183
V2	80	17,872	4,770	15,473	6,017	2,970
V1	80	21,175	5,192	16,455	6,017	3,519
V1	80	19,917	5,036	16,124	6,017	3,310
V2	80	19,808	5,022	16,002	6,017	3,292
V2	80	19,784	5,019	16,160	6,017	3,288
V1	80	19,735	5,013	16,063	6,017	3,280
V2	80	19,421	4,973	15,900	6,017	3,227
V1	80	19,118	4,934	15,717	6,017	3,177
V1	80	18,368	4,836	15,534	6,017	3,053
V2	80	21,187	5,194	16,358	6,018	3,521
V1	80	20,885	5,157	16,282	6,018	3,471
V1	80	20,655	5,128	16,185	6,018	3,432
V1	80	20,510	5,110	16,170	6,018	3,408
V2	80	20,497	5,109	16,318	6,018	3,406
V1	80	20,074	5,056	16,149	6,018	3,336
V1	80	19,941	5,039	16,063	6,018	3,314
V1	80	19,892	5,033	15,976	6,018	3,306
V1	80	19,868	5,030	16,073	6,018	3,302
V2	80	19,759	5,016	15,961	6,018	3,284
V2	80	19,638	5,000	15,890	6,018	3,263
V1	80	19,348	4,963	15,915	6,018	3,215
V1	80	20,570	5,118	16,246	6,018	3,418
V2	80	20,461	5,104	16,221	6,018	3,400
V1	80	19,917	5,036	16,012	6,018	3,310
V1	80	20,715	5,136	16,246	6,018	3,442
V2	80	19,626	4,999	15,951	6,018	3,261
V1	80	19,445	4,976	15,829	6,018	3,231
V1	80	19,227	4,948	15,803	6,018	3,195
V1	80	19,009	4,920	15,707	6,018	3,159
V1	80	21,090	5,182	16,526	6,021	3,503
V1	80	20,268	5,080	16,073	6,021	3,366
V1	80	20,231	5,075	16,195	6,021	3,360
V2	80	20,231	5,075	16,160	6,021	3,360
V2	80	19,953	5,040	15,936	6,021	3,314
V1	80	19,820	5,023	15,926	6,021	3,292
V1	80	19,372	4,966	15,839	6,021	3,218
V2	80	19,324	4,960	15,839	6,021	3,210
V1	80	20,546	5,115	16,282	6,021	3,412
V2	80	20,449	5,103	16,073	6,021	3,396
V2	80	19,820	5,023	16,023	6,021	3,292
V2	80	20,364	5,092	16,073	6,021	3,382
V1	80	19,917	5,036	15,951	6,021	3,308
V1	80	20,631	5,125	16,393	6,023	3,425

V1	80	20,631	5,125	16,393	6,023	3,425
V2	80	20,110	5,060	16,195	6,023	3,339
V1	80	19,965	5,042	16,246	6,024	3,314
V1	80	19,808	5,022	16,256	6,024	3,288
V1	80	20,764	5,142	16,332	6,030	3,443
V2	80	20,304	5,084	16,098	6,030	3,367
V2	80	20,062	5,054	16,098	6,030	3,327
V1	80	19,771	5,017	16,037	6,030	3,279
V2	80	19,747	5,014	16,037	6,030	3,275
V2	80	19,179	4,942	15,854	6,030	3,180
V2	80	18,864	4,901	15,742	6,030	3,128
V1	80	18,513	4,855	15,742	6,030	3,070
V2	80	20,752	5,140	16,221	6,030	3,441
V1	80	18,162	4,809	15,483	6,030	3,012
V1	80	20,062	5,054	16,124	6,030	3,327
V2	80	19,396	4,970	15,803	6,030	3,216
V2	80	18,985	4,917	15,768	6,030	3,148
V1	80	20,231	5,075	16,210	6,031	3,355
V2	80	19,529	4,987	15,951	6,031	3,238
V2	80	21,115	5,185	16,429	6,031	3,501
V1	80	20,679	5,131	16,185	6,031	3,429
V2	80	20,340	5,089	16,185	6,031	3,373
V1	80	20,292	5,083	16,073	6,031	3,365
V2	50	18,803	4,893	15,692	5,821	3,230
V2	50	19,106	4,932	15,717	5,890	3,244
V2	50	19,045	4,924	15,803	5,904	3,226
V2	50	21,127	5,186	17,080	6,514	3,243
V2	50	20,001	5,046	16,073	5,636	3,549
V2	50	19,505	4,983	16,063	5,965	3,270
V2	50	17,497	4,720	15,163	5,725	3,056
V2	50	18,961	4,913	15,666	5,793	3,273
V2	50	20,981	5,169	16,455	6,257	3,353
V2	50	19,529	4,987	15,865	5,880	3,321
V2	50	20,376	5,094	16,221	5,956	3,421
V2	50	18,211	4,815	15,325	5,691	3,200
V2	50	20,110	5,060	16,134	6,071	3,313
V2	50	20,534	5,113	16,282	5,993	3,426
V2	50	20,873	5,155	16,699	5,911	3,531
V2	50	19,566	4,991	15,753	5,814	3,365
V2	50	19,118	4,934	15,544	5,765	3,316
V2	50	20,304	5,084	16,134	5,865	3,462
V2	50	19,082	4,929	15,681	5,904	3,232
V2	50	19,529	4,987	15,890	5,787	3,375
V2	50	18,380	4,838	15,336	5,786	3,177
V2	50	19,421	4,973	15,778	5,821	3,336
V2	50	19,433	4,974	15,692	5,827	3,335
V2	50	18,174	4,810	15,473	5,787	3,141
V2	50	20,534	5,113	16,419	6,120	3,355
V2	50	18,211	4,815	15,336	5,504	3,308
V2	50	19,590	4,994	16,098	6,210	3,154
V2	50	21,526	5,235	16,836	6,300	3,417
V2	50	19,602	4,996	15,926	5,928	3,307

V2	50	19,469	4,979	15,865	6,072	3,206
V2	50	19,275	4,954	15,951	6,147	3,136
V2	50	17,497	4,720	15,005	5,772	3,031
V2	50	18,332	4,831	15,605	5,777	3,173
V2	50	15,815	4,487	14,084	5,373	2,943
V2	50	18,561	4,861	15,447	5,636	3,293
V2	50	19,009	4,920	15,778	6,034	3,150
V2	50	20,836	5,151	16,455	6,087	3,423
V2	50	20,691	5,133	16,221	5,956	3,474
V2	50	20,594	5,121	16,526	6,275	3,282
V2	50	20,171	5,068	16,149	5,826	3,462
V2	50	19,457	4,977	15,926	5,899	3,298
V2	50	18,803	4,893	15,509	5,787	3,249
V2	50	19,154	4,938	15,865	6,032	3,175
V2	50	18,380	4,838	15,595	5,904	3,113
V2	50	20,485	5,107	16,551	6,265	3,270
V2	50	18,428	4,844	15,494	5,725	3,219
V2	50	15,149	4,392	13,825	5,274	2,873
V2	50	21,139	5,188	16,429	6,162	3,431
V2	50	18,646	4,872	15,595	5,927	3,146
V2	50	18,465	4,849	15,397	5,814	3,176
V2	50	17,751	4,754	15,386	5,882	3,018
V2	50	18,743	4,885	15,631	5,899	3,177
V2	50	19,106	4,932	15,692	5,821	3,282
V2	50	20,606	5,122	16,246	6,083	3,388
V2	50	18,102	4,801	15,473	5,850	3,094
V2	50	18,307	4,828	15,656	6,058	3,022
V2	50	18,368	4,836	15,707	5,918	3,104
V2	50	19,009	4,920	15,976	6,111	3,111
V2	50	22,905	5,400	17,594	6,481	3,534
V2	50	19,239	4,949	15,890	6,062	3,174
V2	50	21,405	5,220	16,785	6,170	3,469
V2	50	16,613	4,599	15,224	5,502	3,019
V2	50	21,949	5,286	16,907	6,275	3,498
V2	50	18,937	4,910	15,595	5,964	3,175
V2	50	19,735	5,013	15,926	5,793	3,407
V2	50	22,070	5,301	17,116	6,459	3,417
V2	50	20,534	5,113	16,332	6,134	3,348
V2	50	19,130	4,935	15,839	5,850	3,270
V2	50	19,275	4,954	15,742	5,841	3,300
V2	50	20,255	5,078	16,210	6,017	3,366
V2	50	15,077	4,381	13,962	5,327	2,830
V2	50	19,396	4,970	16,109	5,977	3,245
V2	50	22,046	5,298	17,080	6,217	3,546
V2	50	21,441	5,225	16,688	5,955	3,601
V2	50	21,562	5,240	17,030	6,189	3,484
V2	50	16,843	4,631	14,807	5,504	3,060
V2	50	19,687	5,007	16,332	6,277	3,136
V2	50	19,287	4,956	15,875	5,870	3,286
V2	50	25,809	5,732	18,652	6,998	3,688
V2	50	19,421	4,973	15,926	5,913	3,284
V2	50	18,005	4,788	15,325	5,748	3,132

V2	50	21,853	5,275	16,994	6,387	3,422
V2	50	19,529	4,987	15,951	5,940	3,288
V2	50	21,381	5,218	16,551	6,200	3,449
V2	50	20,122	5,062	16,307	6,264	3,213
V2	50	18,670	4,876	15,646	5,818	3,209
V2	50	19,711	5,010	15,900	6,059	3,253
V2	50	17,279	4,690	14,969	5,571	3,102
V2	50	17,956	4,782	15,336	5,630	3,189
V2	50	21,187	5,194	16,551	6,168	3,435
V2	50	19,590	4,994	15,951	5,967	3,283
V2	50	17,787	4,759	15,239	5,724	3,107
V2	50	19,312	4,959	15,778	5,687	3,396
V2	50	20,038	5,051	16,185	6,067	3,303
V2	50	22,409	5,342	17,080	6,454	3,472
V2	50	20,013	5,048	16,037	5,937	3,371
V2	50	20,643	5,127	16,332	6,017	3,431
V2	50	19,481	4,980	15,742	5,786	3,367
V2	50	20,497	5,109	16,455	6,030	3,399
V2	50	20,134	5,063	16,073	6,021	3,344
V2	50	19,542	4,988	15,778	5,815	3,361
V2	50	20,933	5,163	16,551	6,275	3,336
V2	50	19,578	4,993	15,865	5,899	3,319
V2	50	20,376	5,094	16,185	5,911	3,447
V2	50	19,433	4,974	15,900	5,826	3,335
V2	50	20,981	5,169	16,368	6,096	3,442
V2	50	20,074	5,056	16,109	5,917	3,392
V2	50	21,478	5,229	16,627	6,183	3,474
V2	50	19,251	4,951	15,778	5,993	3,212
V2	50	21,707	5,257	16,785	6,189	3,507
V2	50	19,638	5,000	15,976	5,962	3,294
V2	50	19,179	4,942	15,742	6,058	3,166
V2	50	20,231	5,075	16,256	5,953	3,398
V2	50	21,236	5,200	16,602	6,087	3,489
V2	50	19,070	4,927	15,447	5,772	3,304
V2	50	21,284	5,206	16,602	6,121	3,477
V2	50	15,561	4,451	14,227	4,884	3,186
V2	50	21,526	5,235	16,699	6,228	3,456
V2	50	21,841	5,273	16,922	6,414	3,405
V2	50	21,236	5,200	16,602	6,087	3,489
V2	50	19,070	4,927	15,447	5,772	3,304
V2	50	21,284	5,206	16,602	6,121	3,477
V2	50	21,526	5,235	16,699	6,228	3,456
V2	50	21,841	5,273	16,922	6,414	3,405
V2	50	20,376	5,094	16,307	6,148	3,315
V2	50	20,679	5,131	16,332	6,032	3,428
V2	50	20,195	5,071	16,271	6,211	3,252
V2	50	22,821	5,390	16,958	6,275	3,637
V2	50	19,687	5,007	15,742	5,825	3,380
V2	50	19,154	4,938	15,742	5,918	3,237
V2	50	22,954	5,406	17,141	6,456	3,555
V2	50	18,283	4,825	15,437	5,719	3,197
V2	50	16,166	4,537	14,527	5,431	2,977

V2	50	20,836	5,151	16,541	6,157	3,384
V2	50	19,542	4,988	15,742	5,841	3,345
V2	50	19,493	4,982	15,839	5,793	3,365
V2	50	19,312	4,959	15,742	5,871	3,289
V2	50	20,994	5,170	16,627	6,183	3,395
V2	50	16,855	4,633	14,771	5,579	3,021
V2	50	20,195	5,071	16,098	5,962	3,387
V2	50	20,739	5,139	16,368	6,124	3,387
V2	50	18,949	4,912	15,656	5,819	3,257
V2	50	20,122	5,062	16,160	5,928	3,395
V2	50	21,671	5,253	16,663	6,064	3,574
V2	50	20,280	5,081	16,429	6,151	3,297
V2	50	20,594	5,121	16,343	6,140	3,354
V2	50	18,779	4,890	15,559	5,826	3,224
V2	50	21,417	5,222	16,811	6,278	3,411
V2	50	21,260	5,203	16,882	6,352	3,347
V2	50	20,413	5,098	16,724	6,158	3,315
V2	50	18,949	4,912	15,631	5,786	3,275
V2	50	20,316	5,086	16,307	6,157	3,300
V2	50	18,307	4,828	15,386	5,871	3,118
V2	50	20,425	5,100	16,455	6,364	3,210
V2	50	20,316	5,086	16,170	6,210	3,271
V2	50	19,989	5,045	16,048	5,917	3,378
V2	50	23,789	5,504	17,792	6,711	3,545
V2	50	20,945	5,164	16,516	6,113	3,426
V2	50	21,998	5,292	16,846	6,183	3,558
V2	50	23,692	5,492	17,843	6,850	3,458
V2	50	18,912	4,907	15,692	5,967	3,169
V2	50	20,255	5,078	16,160	6,018	3,366
V2	65	18,549	4,860	15,692	5,899	3,145
V2	65	18,356	4,834	15,351	5,865	3,130
V2	65	18,973	4,915	15,620	5,918	3,206
V2	65	20,098	5,059	16,221	6,118	3,285
V2	65	19,094	4,931	15,681	5,889	3,242
V2	65	19,977	5,043	16,073	6,071	3,291
V2	65	18,465	4,849	15,570	5,852	3,155
V2	65	18,283	4,825	15,178	5,626	3,250
V2	65	19,021	4,921	15,692	5,850	3,251
V2	65	19,626	4,999	15,865	5,984	3,280
V2	65	18,997	4,918	15,768	6,032	3,149
V2	65	17,835	4,765	15,275	5,731	3,112
V2	65	16,783	4,623	14,883	5,582	3,007
V2	65	19,021	4,921	15,976	6,032	3,153
V2	65	17,775	4,757	15,030	5,637	3,153
V2	65	17,969	4,783	15,692	6,134	2,930
V2	65	19,263	4,952	15,839	5,927	3,250
V2	65	19,505	4,983	16,012	6,096	3,200
V2	65	20,800	5,146	16,379	6,091	3,415
V2	65	20,292	5,083	16,551	6,472	3,135
V2	65	18,344	4,833	15,422	5,822	3,151
V2	65	18,489	4,852	15,707	5,882	3,143
V2	65	17,472	4,717	15,091	5,481	3,188

V2	65	19,735	5,013	16,195	6,091	3,240
V2	65	19,614	4,997	16,221	6,275	3,126
V2	65	19,590	4,994	16,037	6,199	3,160
V2	65	19,433	4,974	16,027	6,032	3,222
V2	65	18,622	4,869	15,753	5,934	3,138
V2	65	18,489	4,852	15,814	6,162	3,001
V2	65	19,917	5,036	16,160	6,164	3,231
V2	65	16,347	4,562	14,577	5,336	3,064
V2	65	18,138	4,806	15,498	5,940	3,053
V2	65	18,888	4,904	15,951	6,222	3,035
V2	65	18,174	4,810	15,422	5,852	3,106
V2	65	20,098	5,059	16,343	6,217	3,233
V2	65	17,315	4,695	15,127	5,807	2,982
V2	65	20,159	5,066	16,271	6,093	3,308
V2	65	19,033	4,923	15,570	5,726	3,324
V2	65	16,117	4,530	14,268	5,185	3,108
V2	65	19,287	4,956	15,890	5,826	3,310
V2	65	17,908	4,775	15,336	5,658	3,165
V2	65	18,997	4,918	15,509	5,748	3,305
V2	65	19,880	5,031	16,185	6,067	3,277
V2	65	19,614	4,997	16,343	6,237	3,145
V2	65	19,820	5,023	16,048	6,030	3,287
V2	65	19,493	4,982	16,023	5,819	3,350
V2	65	19,239	4,949	15,839	5,776	3,331
V2	65	20,328	5,087	16,185	6,087	3,340
V2	65	18,586	4,865	15,447	5,786	3,212
V2	65	19,699	5,008	16,048	5,845	3,370
V2	65	23,147	5,429	17,594	6,481	3,571
V2	65	18,428	4,844	15,447	5,787	3,184
V2	65	18,695	4,879	15,483	5,786	3,231
V2	65	18,465	4,849	15,509	5,872	3,145
V2	65	20,147	5,065	16,210	6,094	3,306
V2	65	19,179	4,942	15,793	5,882	3,261
V2	65	19,505	4,983	15,926	6,031	3,234
V2	65	21,066	5,179	16,455	6,032	3,492
V2	65	17,158	4,674	14,796	5,504	3,117
V2	65	19,747	5,014	16,048	5,918	3,337
V2	65	19,215	4,946	15,890	5,918	3,247
V2	65	19,578	4,993	15,839	5,889	3,324
V2	65	19,517	4,985	15,854	5,765	3,385
V2	65	19,820	5,023	15,951	5,994	3,307
V2	65	20,159	5,066	16,195	5,927	3,401
V2	65	19,287	4,956	15,803	5,927	3,254
V2	65	19,021	4,921	15,717	5,793	3,283
V2	65	20,860	5,154	16,368	6,217	3,355
V2	65	19,058	4,926	15,717	5,821	3,274
V2	65	19,033	4,923	15,595	5,927	3,211
V2	65	19,287	4,956	15,681	5,825	3,311
V2	65	19,747	5,014	16,088	5,955	3,316
V2	65	20,449	5,103	16,221	6,117	3,343
V2	65	20,159	5,066	16,221	5,977	3,373
V2	65	17,751	4,754	15,203	5,786	3,068

V2	65	18,997	4,918	15,656	5,715	3,324
V2	65	21,018	5,173	16,749	6,386	3,291
V2	65	20,050	5,053	15,976	5,922	3,386
V2	65	18,924	4,909	15,656	5,743	3,295
V2	65	20,860	5,154	16,444	6,264	3,330
V2	65	18,731	4,884	15,717	6,017	3,113
V2	65	19,868	5,030	16,307	6,463	3,074
V2	65	19,493	4,982	16,012	5,982	3,258
V2	65	20,631	5,125	16,455	6,151	3,354
V2	65	19,905	5,034	16,134	6,034	3,299
V2	65	23,365	5,454	17,584	6,409	3,646
V2	65	20,788	5,145	16,480	6,184	3,362
V2	65	20,147	5,065	16,160	6,199	3,250
V2	65	22,228	5,320	17,141	6,140	3,620
V2	65	20,026	5,049	16,210	6,094	3,286
V2	65	19,977	5,043	16,282	6,304	3,169
V2	65	19,868	5,030	16,160	5,994	3,315
V2	65	20,897	5,158	16,541	6,266	3,335
V2	65	21,139	5,188	16,760	6,367	3,320
V2	65	19,711	5,010	16,221	6,208	3,175
V2	65	22,470	5,349	17,228	6,517	3,448
V2	65	19,614	4,997	16,037	5,994	3,272
V2	65	20,848	5,152	16,785	6,348	3,284
V2	65	22,179	5,314	17,044	6,523	3,400
V2	65	19,965	5,042	16,124	5,964	3,347
V2	65	19,154	4,938	15,753	5,880	3,257
V2	65	18,985	4,917	15,534	5,827	3,258
V2	65	19,989	5,045	16,332	6,363	3,141
V2	65	18,271	4,823	15,509	5,764	3,170
V2	65	19,191	4,943	15,926	5,882	3,262
V2	65	20,752	5,140	16,160	5,850	3,547
V2	65	17,690	4,746	15,310	5,673	3,118
V2	65	17,823	4,764	15,458	5,845	3,049
V2	65	19,070	4,927	15,915	6,058	3,148
V2	65	18,489	4,852	15,620	6,017	3,073
V2	65	19,614	4,997	15,865	5,890	3,330
V2	65	18,223	4,817	15,386	5,772	3,157
V2	65	21,369	5,216	17,156	6,510	3,282
V2	65	20,292	5,083	16,195	6,157	3,296
V2	65	18,695	4,879	15,620	5,922	3,157
V2	65	18,779	4,890	15,570	5,956	3,153
V2	65	17,460	4,715	15,091	5,766	3,028
V2	65	18,017	4,790	15,412	5,882	3,063
V2	65	18,719	4,882	15,666	5,918	3,163
V2	65	17,666	4,743	15,091	5,717	3,090
V2	65	18,247	4,820	15,473	5,772	3,161
V2	65	20,147	5,065	16,124	6,030	3,341
V1	80	19,868	5,030	15,951	6,031	3,294
V2	80	19,868	5,030	16,098	6,031	3,294
V2	80	19,808	5,022	16,048	6,031	3,284
V1	80	19,275	4,954	15,839	6,031	3,196
V1	80	19,251	4,951	15,865	6,031	3,192

V2	80	20,582	5,119	16,271	6,032	3,412
V1	80	20,461	5,104	16,160	6,032	3,392
V2	80	19,554	4,990	15,951	6,032	3,242
V1	80	20,788	5,145	16,307	6,032	3,446
V2	80	20,425	5,100	16,185	6,032	3,386
V2	80	20,280	5,081	16,210	6,032	3,362
V2	80	20,219	5,074	16,073	6,032	3,352
V2	80	19,929	5,037	16,012	6,032	3,304
V2	80	19,820	5,023	16,063	6,032	3,286
V2	80	19,711	5,010	16,012	6,032	3,268
V2	80	19,505	4,983	15,890	6,032	3,234
V1	80	20,727	5,137	16,332	6,032	3,436
V2	80	20,159	5,066	16,124	6,032	3,342
V1	80	18,126	4,804	15,473	6,032	3,005
V1	80	21,707	5,257	16,735	6,034	3,598
V1	80	20,316	5,086	16,221	6,034	3,367
V1	80	19,844	5,027	16,048	6,034	3,289
V1	80	19,808	5,022	16,109	6,034	3,283
V1	80	19,808	5,022	16,109	6,034	3,283
V2	80	19,372	4,966	15,839	6,034	3,211
V2	80	19,058	4,926	15,753	6,034	3,159
V1	80	20,631	5,125	16,393	6,034	3,419
V1	80	20,074	5,056	16,109	6,034	3,327
V2	80	19,965	5,042	16,073	6,034	3,309
V2	80	19,699	5,008	15,987	6,034	3,265
V2	80	19,675	5,005	16,048	6,034	3,261
V2	80	19,578	4,993	15,926	6,034	3,245
V2	80	19,058	4,926	15,778	6,034	3,159
V1	80	20,824	5,149	16,343	6,034	3,451
V2	80	19,892	5,033	16,134	6,042	3,292
V1	80	21,550	5,238	16,699	6,042	3,566
V2	80	20,667	5,130	16,429	6,042	3,420
V2	80	20,606	5,122	16,307	6,042	3,410
V2	80	20,328	5,087	16,343	6,042	3,364
V2	80	20,195	5,071	16,195	6,042	3,342
V1	80	19,566	4,991	16,134	6,042	3,238
V1	80	20,873	5,155	16,404	6,043	3,454
V1	80	20,776	5,143	16,429	6,043	3,438
V1	80	18,622	4,869	15,681	6,053	3,077
V1	80	20,655	5,128	16,419	6,057	3,410
V1	80	20,122	5,062	16,098	6,057	3,322
V1	80	20,074	5,056	16,149	6,057	3,314
V2	80	19,989	5,045	16,098	6,057	3,300
V1	80	19,808	5,022	16,063	6,057	3,270
V1	80	19,699	5,008	15,976	6,057	3,252
V2	80	19,396	4,970	15,890	6,057	3,202
V2	80	20,268	5,080	16,098	6,057	3,346
V1	80	19,663	5,004	15,951	6,057	3,246
V1	80	19,360	4,965	15,829	6,057	3,196
V2	80	21,127	5,186	16,480	6,058	3,488
V1	80	20,776	5,143	16,307	6,058	3,430
V1	80	20,546	5,115	16,419	6,058	3,392

V2	80	19,820	5,023	16,037	6,058	3,272
V1	80	19,650	5,002	15,951	6,058	3,244
V2	80	19,905	5,034	16,037	6,058	3,286
V2	80	21,502	5,232	16,627	6,058	3,549
V2	80	20,401	5,097	16,256	6,058	3,367
V2	80	19,590	4,994	15,987	6,058	3,234
V2	80	19,396	4,970	15,865	6,058	3,202
V2	80	19,070	4,927	15,900	6,058	3,148
V1	80	19,941	5,039	16,098	6,059	3,291
V1	80	19,832	5,025	15,951	6,059	3,273
V1	80	20,522	5,112	16,160	6,059	3,387
V1	80	20,110	5,060	16,145	6,059	3,319
V2	80	19,699	5,008	16,037	6,059	3,251
V2	80	21,417	5,222	16,699	6,061	3,533
V2	80	19,542	4,988	15,987	6,061	3,224
V2	80	19,372	4,966	16,231	6,061	3,196
V2	80	20,122	5,062	16,134	6,062	3,320
V1	80	19,747	5,014	16,073	6,062	3,258
V1	80	21,465	5,228	16,577	6,062	3,541
V2	80	20,510	5,110	16,195	6,062	3,383
V2	80	20,497	5,109	16,221	6,062	3,381
V2	80	20,122	5,062	16,073	6,062	3,319
V2	80	19,759	5,016	16,073	6,062	3,260
V1	80	19,469	4,979	16,084	6,062	3,212
V1	80	18,743	4,885	15,753	6,062	3,092
V1	80	20,655	5,128	16,343	6,062	3,407
V2	80	20,340	5,089	16,221	6,062	3,355
V1	80	20,413	5,098	16,195	6,062	3,367
V2	80	20,110	5,060	16,134	6,062	3,317
V2	80	20,933	5,163	16,465	6,063	3,453
V2	80	20,800	5,146	16,343	6,063	3,431
V2	80	20,667	5,130	16,440	6,063	3,409
V2	80	19,614	4,997	16,063	6,063	3,235
V2	80	20,739	5,139	16,221	6,064	3,420
V1	80	20,594	5,121	16,231	6,064	3,396
V1	80	20,594	5,121	16,185	6,064	3,396
V2	80	20,304	5,084	16,235	6,064	3,348
V2	80	20,086	5,057	15,976	6,064	3,312
V2	80	19,905	5,034	16,063	6,064	3,282
V2	80	20,376	5,094	16,246	6,064	3,360
V2	80	19,433	4,974	15,829	6,064	3,204
V1	80	20,413	5,098	16,358	6,066	3,365
V2	80	20,098	5,059	16,098	6,066	3,313
V2	80	20,098	5,059	16,098	6,066	3,313
V2	80	20,522	5,112	16,332	6,067	3,383
V2	80	19,808	5,022	16,073	6,067	3,265
V2	80	21,284	5,206	16,541	6,071	3,506
V2	80	20,921	5,161	16,368	6,071	3,446
V2	80	20,655	5,128	16,221	6,071	3,402
V1	80	20,546	5,115	16,246	6,071	3,384
V2	80	20,147	5,065	16,195	6,071	3,319
V2	80	20,086	5,057	16,098	6,071	3,309

V2	80	19,892	5,033	16,134	6,071	3,277
V2	80	19,759	5,016	15,976	6,071	3,255
V2	80	20,667	5,130	16,282	6,071	3,404
V2	80	20,534	5,113	16,195	6,071	3,382
V2	80	20,473	5,106	16,134	6,071	3,372
V2	80	20,219	5,074	16,098	6,071	3,330
V1	80	19,070	4,927	15,865	6,071	3,141
V1	80	18,695	4,879	15,631	6,071	3,079
V2	80	21,175	5,192	16,440	6,071	3,488
V2	80	20,800	5,146	16,526	6,071	3,426
V1	80	20,643	5,127	16,246	6,071	3,400
V1	80	20,437	5,101	16,185	6,071	3,366
V2	80	21,962	5,288	17,350	6,072	3,517
V2	80	20,921	5,161	16,419	6,072	3,446
V2	80	20,050	5,053	16,073	6,072	3,302
V2	80	19,542	4,988	16,063	6,072	3,218
V1	80	21,175	5,192	16,505	6,072	3,487
V2	80	20,691	5,133	16,332	6,072	3,407
V1	80	20,643	5,127	16,307	6,072	3,400
V1	80	20,570	5,118	16,297	6,072	3,388
V2	80	20,207	5,072	16,124	6,072	3,328
V2	80	20,739	5,139	16,419	6,073	3,415
V1	80	19,154	4,938	15,915	6,073	3,154
V2	80	20,921	5,161	16,429	6,083	3,439
V2	80	20,921	5,161	16,516	6,083	3,439
V2	80	20,836	5,151	16,429	6,083	3,425
V1	80	20,497	5,109	16,516	6,083	3,370
V2	80	20,316	5,086	16,134	6,083	3,340
V2	80	20,473	5,106	16,455	6,083	3,366
V2	80	20,473	5,106	16,455	6,083	3,366
V1	80	21,102	5,183	16,490	6,083	3,469
V2	80	20,074	5,056	16,134	6,083	3,300
V1	80	20,389	5,095	16,297	6,087	3,350
V2	80	20,510	5,110	16,160	6,087	3,369
V1	80	20,001	5,046	16,210	6,087	3,286
V1	80	19,675	5,005	16,037	6,087	3,232
V1	80	19,517	4,985	15,890	6,087	3,206
V1	80	19,517	4,985	15,976	6,087	3,206
Control	Control	19,191	4,943	15,631	5,927	3,238
Control	Control	18,198	4,814	15,422	5,764	3,157
Control	Control	15,415	4,430	14,084	5,327	2,894
Control	Control	18,598	4,866	15,605	5,764	3,226
Control	Control	20,981	5,169	16,393	6,062	3,461
Control	Control	18,852	4,899	15,707	6,017	3,133
Control	Control	22,458	5,347	17,202	6,536	3,436
Control	Control	17,303	4,694	15,066	5,658	3,058
Control	Control	20,945	5,164	16,516	6,352	3,297
Control	Control	16,432	4,574	14,598	5,570	2,950
Control	Control	20,098	5,059	16,221	6,053	3,321
Control	Control	18,501	4,853	15,386	5,743	3,221
Control	Control	17,593	4,733	15,203	5,826	3,020
Control	Control	21,671	5,253	16,872	6,386	3,393

Control	Control	19,711	5,010	16,210	6,182	3,188
Control	Control	18,223	4,817	15,483	5,764	3,161
Control	Control	16,323	4,559	14,638	5,431	3,006
Control	Control	18,791	4,891	15,692	5,955	3,156
Control	Control	18,900	4,906	15,926	6,164	3,066
Control	Control	18,840	4,898	15,717	5,993	3,144
Control	Control	19,094	4,931	15,768	5,904	3,234
Control	Control	20,522	5,112	16,455	6,170	3,326
Control	Control	17,073	4,662	14,893	5,609	3,044
Control	Control	18,332	4,831	15,447	5,760	3,183
Control	Control	18,029	4,791	15,422	5,793	3,112
Control	Control	19,324	4,960	15,926	5,850	3,303
Control	Control	19,626	4,999	15,976	5,940	3,304
Control	Control	19,711	5,010	16,073	6,124	3,219
Control	Control	18,574	4,863	15,559	5,882	3,158
Control	Control	18,053	4,794	15,152	5,580	3,235
Control	Control	19,759	5,016	16,134	6,083	3,248
Control	Control	19,784	5,019	16,221	6,042	3,274
Control	Control	18,985	4,917	16,246	5,967	3,182
Control	Control	19,227	4,948	15,778	5,880	3,270
Control	Control	21,320	5,210	16,638	6,301	3,384
Control	Control	19,941	5,039	16,343	6,170	3,232
Control	Control	18,211	4,815	15,289	5,772	3,155
Control	Control	18,090	4,799	15,178	5,582	3,241
Control	Control	17,702	4,748	15,178	5,786	3,059
Control	Control	19,626	4,999	16,271	6,301	3,115
Control	Control	18,162	4,809	15,473	5,882	3,088
Control	Control	18,186	4,812	15,534	5,995	3,034
Control	Control	19,372	4,966	15,900	5,928	3,268
Control	Control	22,482	5,350	17,177	6,317	3,559
Control	Control	18,646	4,872	15,534	5,732	3,253
Control	Control	20,715	5,136	16,465	6,197	3,343
Control	Control	18,695	4,879	16,134	6,413	2,915
Control	Control	19,372	4,966	15,951	6,087	3,183
Control	Control	18,344	4,833	15,412	5,697	3,220
Control	Control	20,062	5,054	16,307	6,356	3,156
Control	Control	18,912	4,907	15,753	5,793	3,265
Control	Control	21,066	5,179	16,638	6,170	3,414
Control	Control	18,682	4,877	15,498	5,818	3,211
Control	Control	20,231	5,075	16,246	6,237	3,244
Control	Control	18,223	4,817	15,605	5,826	3,128
Control	Control	19,989	5,045	16,073	6,171	3,239
Control	Control	18,743	4,885	15,447	5,815	3,223
Control	Control	18,731	4,884	15,620	5,927	3,160
Control	Control	20,981	5,169	16,490	6,140	3,417
Control	Control	18,186	4,812	15,361	5,918	3,073
Control	Control	18,041	4,793	15,483	5,826	3,097
Control	Control	19,421	4,973	15,854	5,977	3,249
Control	Control	21,659	5,251	17,044	6,531	3,316
Control	Control	18,259	4,822	15,412	5,672	3,219
Control	Control	19,227	4,948	15,717	5,825	3,301
Control	Control	19,324	4,960	15,656	5,865	3,295

Control	Control	17,134	4,671	15,001	5,433	3,153
Control	Control	19,360	4,965	15,768	5,841	3,314
Control	Control	19,445	4,976	15,829	5,937	3,275
Control	Control	17,993	4,786	15,336	5,786	3,110
Control	Control	18,598	4,866	15,473	5,786	3,214
Control	Control	18,646	4,872	15,681	5,940	3,139
Control	Control	17,025	4,656	14,872	5,526	3,081
Control	Control	20,098	5,059	16,358	6,238	3,222
Control	Control	20,316	5,086	16,185	5,927	3,427
Control	Control	18,077	4,798	15,447	6,064	2,981
Control	Control	18,186	4,812	15,214	5,622	3,235
Control	Control	19,808	5,022	16,012	5,826	3,400
Control	Control	18,779	4,890	15,534	5,882	3,193
Control	Control	18,198	4,814	15,544	5,918	3,075
Control	Control	19,142	4,937	15,778	5,899	3,245
Control	Control	18,235	4,818	15,361	5,777	3,156
Control	Control	18,731	4,884	15,692	5,764	3,250
Control	Control	19,045	4,924	15,778	5,985	3,182
Control	Control	19,215	4,946	15,926	5,977	3,215
Control	Control	18,223	4,817	15,264	5,719	3,186
Control	Control	18,816	4,895	15,915	6,120	3,074
Control	Control	19,965	5,042	15,951	5,956	3,352
Control	Control	20,655	5,128	16,541	6,337	3,259
Control	Control	17,763	4,756	15,544	5,772	3,078
Control	Control	16,674	4,608	14,746	5,692	2,930
Control	Control	19,675	5,005	16,160	6,117	3,216
Control	Control	20,171	5,068	16,663	6,061	3,328
Control	Control	19,179	4,942	15,875	6,034	3,179
Control	Control	20,497	5,109	16,256	6,111	3,354
Control	Control	19,372	4,966	16,221	6,358	3,047
Control	Control	18,997	4,918	15,753	6,156	3,086
Control	Control	19,578	4,993	15,951	6,003	3,261
Control	Control	19,832	5,025	16,124	6,064	3,270
Control	Control	19,711	5,010	15,951	6,017	3,276
Control	Control	19,590	4,994	16,027	5,922	3,308
Control	Control	21,683	5,254	16,811	6,317	3,432
Control	Control	18,162	4,809	15,178	5,645	3,217
Control	Control	23,147	5,429	17,497	6,867	3,371
Control	Control	19,493	4,982	15,915	5,940	3,281
Control	Control	20,606	5,122	16,282	6,117	3,369
Control	Control	17,956	4,782	15,203	5,683	3,160
Control	Control	18,525	4,857	15,681	5,913	3,133
Control	Control	19,880	5,031	15,951	5,971	3,329
Control	Control	19,699	5,008	15,951	5,977	3,296
Control	Control	19,832	5,025	16,124	6,094	3,255
Control	Control	19,469	4,979	16,002	6,067	3,209
Control	Control	19,045	4,924	15,753	5,821	3,272
Control	Control	20,086	5,057	16,210	6,094	3,296
Control	Control	16,359	4,564	14,501	5,380	3,041
Control	Control	21,586	5,243	16,627	6,127	3,523
Control	Control	19,505	4,983	16,012	6,062	3,218
Control	Control	19,058	4,926	15,681	5,865	3,249

Control	Control	20,534	5,113	16,318	6,134	3,348
Control	Control	21,272	5,204	16,739	6,519	3,263
Control	Control	19,021	4,921	15,879	6,017	3,161
Control	Control	19,965	5,042	16,063	5,814	3,434
Control	Control	18,319	4,830	15,372	5,673	3,229
Control	Control	19,263	4,952	15,875	5,899	3,266
Control	Control	18,307	4,828	15,361	5,777	3,169
Control	Control	19,566	4,991	15,961	5,984	3,269
Control	Control	22,143	5,310	17,044	6,309	3,510
Control	Control	19,880	5,031	16,149	6,222	3,195
Control	Control	20,110	5,060	16,134	5,899	3,409
Control	Control	18,537	4,858	15,605	5,629	3,293
Control	Control	20,945	5,164	16,516	6,275	3,338
Control	Control	20,800	5,146	16,343	6,030	3,449
V1	50	18,150	4,807	15,361	5,717	3,175
V1	50	19,082	4,929	15,656	5,825	3,276
V1	50	20,328	5,087	16,332	6,147	3,307
V1	50	17,364	4,702	14,919	5,466	3,176
V1	50	16,601	4,598	14,761	5,636	2,945
V1	50	18,767	4,888	15,631	5,845	3,211
V1	50	20,183	5,069	16,134	5,956	3,389
V1	50	20,522	5,112	16,246	6,164	3,329
V1	50	19,784	5,019	16,012	6,111	3,237
V1	50	19,033	4,923	15,890	5,994	3,175
V1	50	19,566	4,991	16,210	6,257	3,127
V1	50	19,203	4,945	15,768	5,881	3,265
V1	50	19,070	4,927	15,692	5,793	3,292
V1	50	16,335	4,561	14,613	5,571	2,932
V1	50	19,929	5,037	16,124	6,032	3,304
V1	50	20,594	5,121	16,332	6,112	3,369
V1	50	20,655	5,128	16,602	6,210	3,326
V1	50	21,127	5,186	16,638	6,310	3,348
V1	50	18,670	4,876	15,742	5,918	3,155
V1	50	21,139	5,188	16,663	6,309	3,350
V1	50	20,945	5,164	16,343	6,062	3,455
V1	50	19,058	4,926	15,778	5,927	3,215
V1	50	17,739	4,752	15,264	5,724	3,099
V1	50	21,357	5,215	16,577	6,333	3,372
V1	50	19,529	4,987	15,890	5,922	3,298
V1	50	19,711	5,010	16,098	6,032	3,268
V1	50	20,364	5,092	16,343	6,034	3,375
V1	50	19,892	5,033	16,195	5,743	3,464
V1	50	19,058	4,926	15,890	5,922	3,218
V1	50	19,929	5,037	16,134	6,134	3,249
V1	50	18,864	4,901	15,681	5,918	3,188
V1	50	19,675	5,005	16,185	6,032	3,262
V1	50	18,537	4,858	15,483	5,776	3,209
V1	50	19,239	4,949	16,124	6,263	3,072
V1	50	21,296	5,207	16,688	6,428	3,313
V1	50	19,687	5,007	16,109	6,134	3,209
V1	50	18,840	4,898	15,778	5,607	3,360
V1	50	18,670	4,876	15,631	5,776	3,232

V1	50	21,320	5,210	16,480	6,200	3,439
V1	50	16,202	4,542	14,440	5,480	2,957
V1	50	19,650	5,002	16,073	6,167	3,186
V1	50	18,646	4,872	15,570	5,777	3,228
V1	50	20,110	5,060	16,160	5,934	3,389
V1	50	19,009	4,920	15,768	6,011	3,162
V1	50	22,470	5,349	17,167	6,488	3,463
V1	50	18,658	4,874	15,778	5,993	3,113
V1	50	20,994	5,170	16,393	6,124	3,428
V1	50	19,675	5,005	16,292	6,140	3,204
V1	50	19,529	4,987	15,890	5,936	3,290
V1	50	20,280	5,081	16,221	6,128	3,309
V1	50	20,401	5,097	16,455	6,222	3,279
V1	50	20,062	5,054	16,170	5,956	3,368
V1	50	19,590	4,994	15,961	5,899	3,321
V1	50	19,118	4,934	15,778	6,032	3,170
V1	50	20,800	5,146	16,455	6,222	3,343
V1	50	19,505	4,983	15,976	6,032	3,234
V1	50	20,788	5,145	16,343	6,064	3,428
V1	50	20,340	5,089	16,195	6,005	3,387
V1	50	20,691	5,133	16,429	6,090	3,397
V1	50	18,501	4,853	15,646	5,826	3,175
V1	50	17,690	4,746	15,066	5,748	3,077
V1	50	17,981	4,785	15,188	5,542	3,244
V1	50	18,344	4,833	15,631	5,807	3,159
V1	50	19,312	4,959	15,987	6,042	3,196
V1	50	18,247	4,820	15,336	5,502	3,316
V1	50	19,759	5,016	15,987	6,058	3,262
V1	50	19,771	5,017	16,012	6,117	3,232
V1	50	19,820	5,023	16,037	6,065	3,268
V1	50	20,739	5,139	16,749	6,301	3,291
V1	50	18,029	4,791	15,509	6,199	2,908
V1	50	19,445	4,976	16,073	5,870	3,312
V1	50	19,880	5,031	16,037	5,936	3,349
V1	50	19,457	4,977	15,803	5,877	3,311
V1	50	20,788	5,145	16,465	6,363	3,267
V1	50	19,421	4,973	15,900	5,918	3,282
V1	50	19,759	5,016	16,012	5,912	3,342
V1	50	20,534	5,113	16,195	6,096	3,368
V1	50	18,695	4,879	15,458	5,726	3,265
V1	50	19,929	5,037	16,124	6,127	3,252
V1	50	19,675	5,005	15,976	6,111	3,219
V1	50	19,723	5,011	15,987	5,927	3,328
V1	50	20,558	5,116	16,343	6,169	3,333
V1	50	20,643	5,127	16,429	6,096	3,386
V1	50	20,074	5,056	16,282	6,043	3,322
V1	50	18,259	4,822	15,386	5,691	3,208
V1	50	20,352	5,091	16,379	6,112	3,330
V1	50	19,324	4,960	15,742	5,889	3,281
V1	50	19,130	4,935	15,768	6,018	3,179
V1	50	19,844	5,027	16,073	6,111	3,247
V1	50	16,129	4,532	14,232	5,227	3,086

V1	50	19,251	4,951	15,692	5,818	3,309
V1	50	19,505	4,983	15,951	6,087	3,205
V1	50	21,102	5,183	16,775	6,531	3,231
V1	50	20,098	5,059	16,221	6,063	3,315
V1	50	19,784	5,019	16,160	6,358	3,112
V1	50	16,686	4,609	14,735	5,544	3,010
V1	50	19,106	4,932	15,646	5,927	3,224
V1	50	19,929	5,037	15,951	6,096	3,269
V1	50	18,731	4,884	15,631	5,904	3,173
V1	50	21,042	5,176	16,455	6,168	3,411
V1	50	22,784	5,386	17,116	6,438	3,539
V1	50	16,831	4,629	14,761	5,661	2,973
V1	50	18,852	4,899	15,498	5,786	3,258
V1	50	19,844	5,027	15,976	6,091	3,258
V1	50	18,598	4,866	15,473	5,882	3,162
V1	50	20,885	5,157	16,332	6,118	3,414
V1	50	22,579	5,362	16,969	6,332	3,566
V1	50	20,001	5,046	15,951	5,993	3,337
V1	50	17,763	4,756	15,152	5,582	3,182
V1	50	20,401	5,097	16,221	6,169	3,307
V1	50	19,251	4,951	15,865	6,059	3,178
V1	50	18,283	4,825	15,361	5,725	3,193
V1	50	17,400	4,707	15,005	5,552	3,134
V1	50	18,465	4,849	15,717	5,956	3,100
V1	50	18,428	4,844	15,620	6,018	3,062
V1	50	19,336	4,962	15,829	6,058	3,192
V1	50	19,868	5,030	16,012	6,018	3,302
V1	50	21,175	5,192	16,541	6,278	3,373
V1	50	20,703	5,134	16,440	6,124	3,381
V1	50	21,732	5,260	16,836	6,376	3,408
V1	50	18,319	4,830	15,361	5,626	3,256
V1	50	18,937	4,910	15,803	5,985	3,164
V1	50	20,606	5,122	16,271	6,112	3,371
V1	50	20,110	5,060	16,185	6,073	3,312
V1	50	20,183	5,069	16,343	6,151	3,281
V1	50	18,767	4,888	15,559	5,731	3,275
V1	50	20,679	5,131	16,419	6,087	3,397
V1	50	17,351	4,700	14,847	5,480	3,166
V1	65	18,876	4,902	15,742	6,005	3,143
V1	65	17,364	4,702	15,249	5,717	3,037
V1	65	17,848	4,767	15,188	5,927	3,011
V1	65	20,546	5,115	16,465	6,172	3,329
V1	65	17,037	4,657	14,944	5,715	2,981
V1	65	17,666	4,743	15,188	5,608	3,150
V1	65	20,994	5,170	16,933	6,487	3,236
V1	65	19,844	5,027	16,012	6,120	3,242
V1	65	19,251	4,951	15,854	5,904	3,261
V1	65	19,796	5,020	16,063	6,032	3,282
V1	65	20,316	5,086	16,526	6,227	3,262
V1	65	19,650	5,002	16,170	6,167	3,186
V1	65	20,510	5,110	16,541	6,337	3,237
V1	65	18,912	4,907	15,839	5,934	3,187

V1	65	18,876	4,902	15,814	6,058	3,116
V1	65	17,364	4,702	14,919	5,572	3,116
V1	65	19,045	4,924	15,793	6,211	3,067
V1	65	19,033	4,923	15,753	6,017	3,163
V1	65	19,953	5,040	16,343	6,164	3,237
V1	65	17,727	4,751	15,214	5,658	3,133
V1	65	20,013	5,048	16,256	5,953	3,362
V1	65	21,236	5,200	16,612	6,083	3,491
V1	65	18,077	4,798	15,412	5,904	3,062
V1	65	20,268	5,080	16,541	6,400	3,167
V1	65	20,171	5,068	16,383	6,364	3,170
V1	65	15,754	4,479	14,343	5,486	2,872
V1	65	17,472	4,717	15,127	5,765	3,031
V1	65	17,920	4,777	15,239	5,827	3,075
V1	65	18,525	4,857	15,422	5,715	3,242
V1	65	18,198	4,814	15,707	6,112	2,977
V1	65	19,663	5,004	16,098	6,137	3,204
V1	65	18,791	4,891	15,509	5,850	3,212
V1	65	18,211	4,815	15,422	5,822	3,128
V1	65	20,691	5,133	16,699	6,410	3,228
V1	65	19,203	4,945	15,778	5,927	3,240
V1	65	19,578	4,993	16,160	6,259	3,128
V1	65	20,026	5,049	16,516	6,456	3,102
V1	65	19,348	4,963	16,037	6,168	3,137
V1	65	18,332	4,831	15,987	6,062	3,024
V1	65	19,566	4,991	16,048	6,071	3,223
V1	65	20,074	5,056	16,012	6,162	3,258
V1	65	20,207	5,072	16,602	6,510	3,104
V1	65	20,389	5,095	16,429	6,273	3,250
V1	65	18,114	4,802	15,483	5,927	3,056
V1	65	19,554	4,990	16,124	5,984	3,267
V1	65	20,461	5,104	16,663	6,409	3,192
V1	65	19,481	4,980	15,839	5,922	3,289
V1	65	19,239	4,949	15,620	5,872	3,277
V1	65	17,085	4,664	14,980	5,532	3,088
V1	65	19,796	5,020	16,134	6,072	3,260
V1	65	19,179	4,942	15,987	6,111	3,138
V1	65	17,702	4,748	15,336	5,870	3,016
V1	65	15,742	4,477	14,181	5,321	2,959
V1	65	19,868	5,030	16,073	6,021	3,300
V1	65	19,275	4,954	15,692	5,814	3,315
V1	65	17,230	4,684	15,214	5,825	2,958
V1	65	18,150	4,807	15,361	5,841	3,107
V1	65	18,973	4,915	15,778	5,995	3,165
V1	65	19,602	4,996	16,134	6,140	3,193
V1	65	18,416	4,842	15,483	5,852	3,147
V1	65	21,127	5,186	16,612	6,246	3,382
V1	65	18,077	4,798	15,570	5,953	3,037
V1	65	17,775	4,757	15,264	5,724	3,105
V1	65	18,440	4,846	15,584	6,011	3,068
V1	65	20,001	5,046	16,516	6,112	3,273
V1	65	16,734	4,616	14,944	5,658	2,958

V1	65	22,409	5,342	17,080	6,453	3,473
V1	65	18,598	4,866	15,570	5,918	3,142
V1	65	18,174	4,810	15,264	5,872	3,095
V1	65	18,719	4,882	15,768	6,157	3,040
V1	65	20,122	5,062	16,480	6,247	3,221
V1	65	19,663	5,004	16,098	5,967	3,295
V1	65	19,687	5,007	15,987	5,880	3,348
V1	65	16,783	4,623	14,685	5,542	3,028
V1	65	19,892	5,033	16,134	6,072	3,276
V1	65	19,251	4,951	15,753	5,786	3,327
V1	65	19,372	4,966	16,134	6,042	3,206
V1	65	20,873	5,155	16,429	6,168	3,384
V1	65	17,896	4,773	15,275	5,764	3,105
V1	65	18,259	4,822	15,397	5,645	3,234
V1	65	20,691	5,133	16,419	6,184	3,346
V1	65	17,811	4,762	15,127	5,686	3,132
V1	65	18,259	4,822	15,458	5,524	3,306
V1	65	18,489	4,852	15,447	5,818	3,178
V1	65	17,787	4,759	15,188	5,748	3,094
V1	65	18,949	4,912	15,742	5,927	3,197
V1	65	18,937	4,910	15,666	5,687	3,330
V1	65	18,549	4,860	15,422	5,877	3,156
V1	65	18,549	4,860	15,620	5,940	3,123
V1	65	20,848	5,152	16,516	6,246	3,338
V1	65	18,053	4,794	15,214	5,609	3,219
V1	65	18,513	4,855	15,509	5,737	3,227
V1	65	19,082	4,929	15,707	5,882	3,244
V1	65	19,021	4,921	15,692	5,826	3,265
V1	65	19,977	5,043	16,307	6,157	3,245
V1	65	18,150	4,807	15,422	5,927	3,062
V1	65	18,549	4,860	15,692	5,852	3,170
V1	65	18,090	4,799	15,386	5,841	3,097
V1	65	18,392	4,839	15,386	5,827	3,157
V1	65	19,711	5,010	16,098	6,067	3,249
V1	65	18,211	4,815	15,865	6,011	3,030
V1	65	17,388	4,705	15,056	5,677	3,063
V1	65	21,526	5,235	17,202	6,369	3,380
V1	65	21,502	5,232	16,846	6,297	3,414
V1	65	17,315	4,695	15,005	5,865	2,952
V1	65	18,912	4,907	15,656	5,751	3,289
V1	65	19,457	4,977	15,865	5,927	3,283
V1	65	17,194	4,679	14,919	5,474	3,141
V1	65	19,578	4,993	16,023	5,918	3,308
V1	65	17,485	4,718	15,127	5,676	3,080
V1	65	20,098	5,059	16,404	6,278	3,201
V1	80	19,396	4,970	15,951	6,087	3,186
V1	80	19,094	4,931	15,926	6,087	3,137
V2	80	20,933	5,163	16,429	6,090	3,437
V1	80	20,643	5,127	16,343	6,090	3,389
V2	80	20,449	5,103	16,221	6,090	3,358
V1	80	20,147	5,065	16,343	6,090	3,308
V2	80	20,098	5,059	16,221	6,090	3,300

V1	80	19,917	5,036	16,221	6,090	3,270
V2	80	19,784	5,019	15,951	6,090	3,248
V2	80	19,759	5,016	16,048	6,090	3,244
V1	80	19,590	4,994	16,073	6,090	3,217
V1	80	21,308	5,209	16,612	6,091	3,499
V2	80	20,606	5,122	16,246	6,091	3,383
V1	80	19,844	5,027	16,048	6,091	3,258
V1	80	19,832	5,025	16,195	6,091	3,256
V2	80	18,779	4,890	15,717	6,091	3,083
V2	80	21,260	5,203	16,627	6,093	3,489
V1	80	20,691	5,133	16,429	6,093	3,396
V2	80	20,606	5,122	16,307	6,093	3,382
V2	80	20,582	5,119	16,332	6,093	3,378
V2	80	20,292	5,083	16,246	6,093	3,330
V2	80	20,050	5,053	16,210	6,093	3,291
V1	80	19,614	4,997	16,012	6,093	3,219
V2	80	20,522	5,112	16,332	6,094	3,368
V1	80	20,231	5,075	16,210	6,094	3,320
V2	80	20,546	5,115	16,393	6,094	3,372
V2	80	18,501	4,853	15,681	6,094	3,036
V2	80	21,320	5,210	16,465	6,096	3,497
V2	80	20,752	5,140	16,282	6,096	3,404
V1	80	20,485	5,107	16,282	6,096	3,360
V2	80	20,473	5,106	16,221	6,096	3,359
V2	80	20,280	5,081	16,098	6,096	3,327
V2	80	20,038	5,051	16,185	6,096	3,287
V2	80	20,038	5,051	16,160	6,096	3,287
V1	80	19,856	5,028	16,098	6,096	3,257
V1	80	18,513	4,855	15,987	6,096	3,037
V2	80	21,465	5,228	16,551	6,096	3,521
V1	80	20,038	5,051	16,037	6,096	3,287
V2	80	19,808	5,022	15,951	6,096	3,249
V1	80	19,590	4,994	16,073	6,096	3,213
V1	80	19,372	4,966	16,012	6,096	3,178
V1	80	18,985	4,917	15,839	6,096	3,114
V2	80	21,284	5,206	16,516	6,096	3,491
V2	80	20,643	5,127	16,246	6,096	3,386
V1	80	20,631	5,125	16,343	6,096	3,384
V2	80	19,880	5,031	16,073	6,096	3,261
V1	80	21,090	5,182	16,393	6,111	3,451
V1	80	19,542	4,988	15,976	6,111	3,198
V2	80	20,643	5,127	16,282	6,111	3,378
V2	80	20,618	5,124	16,246	6,111	3,374
V2	80	21,393	5,219	16,612	6,111	3,501
V1	80	21,272	5,204	16,429	6,111	3,481
V1	80	20,691	5,133	16,307	6,111	3,386
V2	80	20,268	5,080	16,256	6,111	3,316
V1	80	20,159	5,066	16,256	6,111	3,299
V2	80	20,026	5,049	16,098	6,111	3,277
V1	80	20,001	5,046	16,231	6,111	3,273
V2	80	19,759	5,016	16,134	6,111	3,233
V2	80	19,590	4,994	16,109	6,111	3,206

V1	80	21,151	5,189	16,516	6,111	3,461
V2	80	20,945	5,164	16,429	6,111	3,427
V1	80	20,606	5,122	16,541	6,111	3,372
V2	80	20,328	5,087	16,318	6,111	3,326
V2	80	20,328	5,087	16,318	6,111	3,326
V2	80	20,292	5,083	16,282	6,111	3,320
V1	80	19,408	4,971	16,048	6,111	3,176
V1	80	19,408	4,971	16,048	6,111	3,176
V1	80	19,215	4,946	16,023	6,111	3,144
V1	80	20,776	5,143	16,602	6,112	3,399
V2	80	20,631	5,125	16,343	6,112	3,375
V1	80	20,582	5,119	16,404	6,112	3,368
V1	80	20,473	5,106	16,516	6,112	3,350
V2	80	20,098	5,059	16,256	6,112	3,288
V1	80	19,880	5,031	16,160	6,112	3,253
V2	80	19,372	4,966	15,926	6,112	3,169
V2	80	21,260	5,203	16,490	6,112	3,478
V2	80	20,292	5,083	16,185	6,112	3,320
V1	80	20,038	5,051	16,037	6,112	3,278
V1	80	19,929	5,037	16,160	6,112	3,260
V1	80	19,638	5,000	16,063	6,112	3,213
V2	80	20,159	5,066	16,160	6,113	3,298
V1	80	20,969	5,167	16,419	6,113	3,430
V2	80	20,715	5,136	16,307	6,113	3,389
V1	80	19,844	5,027	16,160	6,113	3,246
V2	80	20,316	5,086	16,282	6,117	3,321
V1	80	20,231	5,075	16,160	6,117	3,307
V2	80	19,941	5,039	16,160	6,117	3,260
V1	80	19,892	5,033	16,012	6,117	3,252
V2	80	19,856	5,028	16,048	6,117	3,246
V2	80	20,134	5,063	16,160	6,118	3,291
V2	80	20,824	5,149	16,429	6,118	3,404
V2	80	20,739	5,139	16,490	6,118	3,390
V1	80	20,461	5,104	16,271	6,118	3,344
V1	80	20,171	5,068	16,098	6,118	3,297
V2	80	19,917	5,036	16,037	6,118	3,255
V2	80	21,562	5,240	16,688	6,120	3,523
V1	80	21,054	5,178	16,566	6,120	3,440
V2	80	20,752	5,140	16,393	6,120	3,391
V2	80	20,558	5,116	16,307	6,120	3,359
V1	80	19,602	4,996	16,124	6,120	3,203
V2	80	20,122	5,062	16,185	6,121	3,288
V2	80	19,759	5,016	16,124	6,121	3,228
V2	80	19,469	4,979	15,951	6,121	3,181
V1	80	21,429	5,223	16,688	6,121	3,501
V2	80	21,393	5,219	16,602	6,121	3,495
V1	80	20,981	5,169	16,566	6,121	3,428
V1	80	20,618	5,124	16,282	6,124	3,367
V1	80	20,473	5,106	16,221	6,124	3,343
V2	80	20,449	5,103	16,221	6,124	3,339
V2	80	20,449	5,103	16,221	6,124	3,339
V1	80	19,832	5,025	16,134	6,124	3,238

V2	80	19,493	4,982	16,048	6,124	3,183
V1	80	19,227	4,948	15,768	6,124	3,140
V2	80	20,340	5,089	16,221	6,124	3,321
V2	80	20,969	5,167	16,465	6,125	3,424
V2	80	20,969	5,167	16,465	6,125	3,424
V1	80	20,691	5,133	16,393	6,125	3,378
V2	80	19,832	5,025	16,073	6,125	3,238
V2	80	19,663	5,004	16,073	6,125	3,210
V2	80	20,679	5,131	16,393	6,127	3,375
V1	80	20,534	5,113	16,185	6,127	3,351
V2	80	20,413	5,098	16,246	6,127	3,332
V2	80	20,328	5,087	16,246	6,127	3,318
V2	80	19,687	5,007	16,063	6,127	3,213
V2	80	20,134	5,063	16,210	6,127	3,286
V1	80	19,784	5,019	16,124	6,127	3,229
V1	80	19,687	5,007	16,037	6,127	3,213
V2	80	19,082	4,929	15,890	6,127	3,114
V2	80	19,880	5,031	16,098	6,128	3,244
V1	80	20,413	5,098	16,343	6,132	3,329
V2	80	20,026	5,049	16,343	6,132	3,266
V2	80	21,478	5,229	16,490	6,134	3,502
V1	80	21,284	5,206	16,480	6,134	3,470
V1	80	21,139	5,188	16,455	6,134	3,446
V1	80	20,485	5,107	16,282	6,134	3,340
V2	80	20,255	5,078	16,160	6,134	3,302
V2	80	19,965	5,042	16,124	6,134	3,255
V2	80	19,602	4,996	15,951	6,134	3,196
V2	80	19,517	4,985	15,951	6,134	3,182
V2	80	20,776	5,143	16,256	6,134	3,387
V1	80	20,594	5,121	16,368	6,134	3,358
V1	80	19,517	4,985	16,012	6,134	3,182
V2	80	19,348	4,963	15,987	6,134	3,154
V1	80	21,030	5,175	16,404	6,134	3,428
V2	80	20,340	5,089	16,160	6,134	3,316
V2	80	20,352	5,091	16,332	6,137	3,316
V1	80	19,566	4,991	16,149	6,137	3,188
V2	80	20,001	5,046	16,160	6,137	3,259
V1	80	21,841	5,273	16,749	6,140	3,557
V2	80	21,369	5,216	16,577	6,140	3,480
V2	80	21,042	5,176	16,612	6,140	3,427
V1	80	20,812	5,148	16,404	6,140	3,390
V2	80	20,715	5,136	16,368	6,140	3,374
V1	80	20,582	5,119	16,343	6,140	3,352
V1	80	20,570	5,118	16,490	6,140	3,350
V1	80	20,534	5,113	16,343	6,140	3,344
V2	80	20,534	5,113	16,393	6,140	3,344
V1	80	20,207	5,072	16,379	6,140	3,291
V2	50	20,631	5,125	16,419	6,157	3,351
V2	50	17,424	4,710	14,995	5,683	3,066
V2	50	19,179	4,942	15,839	5,807	3,303
V2	50	19,699	5,008	16,073	6,034	3,265
V2	50	21,163	5,191	16,551	6,200	3,414

V2	50	17,557	4,728	15,066	5,609	3,130
V2	50	18,828	4,896	15,620	5,841	3,223
V2	50	15,742	4,477	14,354	5,446	2,891
V2	50	18,380	4,838	15,559	5,882	3,125
V2	50	18,695	4,879	15,595	5,872	3,184
V2	50	18,368	4,836	15,336	5,872	3,128
V2	50	19,735	5,013	15,976	5,967	3,307
V2	50	18,695	4,879	15,570	5,867	3,186
V2	50	19,457	4,977	15,803	5,927	3,283
V2	50	18,876	4,902	15,570	5,786	3,262
V2	50	19,481	4,980	16,012	6,073	3,208
V2	50	18,912	4,907	15,666	5,927	3,191
V2	50	17,194	4,679	15,005	5,697	3,018
V2	50	18,670	4,876	15,839	5,934	3,146
V2	50	19,179	4,942	15,976	5,867	3,269
V2	50	21,949	5,286	17,339	6,728	3,262
V2	50	18,465	4,849	15,519	5,619	3,286
V2	50	19,130	4,935	15,681	6,030	3,172
V2	50	19,735	5,013	16,109	5,852	3,372
V2	50	18,598	4,866	15,361	5,749	3,235
V2	50	19,723	5,011	15,829	5,852	3,371
V2	50	21,151	5,189	16,663	6,303	3,356
V2	50	18,743	4,885	15,580	5,743	3,263
V2	50	19,287	4,956	15,753	5,880	3,280
V2	50	20,159	5,066	16,098	6,071	3,321
V2	50	19,784	5,019	16,246	6,121	3,232
V2	50	20,159	5,066	16,048	5,971	3,376
V2	50	19,336	4,962	15,976	6,167	3,135
V2	50	19,977	5,043	16,124	5,977	3,342
V2	50	18,307	4,828	15,483	5,870	3,119
V2	50	19,287	4,956	15,778	5,927	3,254
V2	50	20,171	5,068	16,455	6,157	3,276
V2	50	19,578	4,993	15,951	6,167	3,175
V2	50	20,086	5,057	16,587	6,340	3,168
V2	50	18,900	4,906	15,692	5,927	3,189
V2	50	20,921	5,161	16,983	6,692	3,126
V2	50	16,299	4,555	14,354	5,021	3,246
V2	50	18,961	4,913	15,839	6,071	3,123
V2	50	19,723	5,011	16,343	6,197	3,183
V2	50	17,606	4,735	15,178	5,772	3,050
V2	50	19,965	5,042	16,037	5,977	3,340
V2	50	19,832	5,025	16,271	6,112	3,244
V2	50	17,424	4,710	15,386	6,058	2,876
V2	50	20,824	5,149	16,688	6,337	3,286
V2	50	18,005	4,788	15,264	5,964	3,019
V2	50	20,510	5,110	16,195	6,167	3,326
V2	50	21,223	5,198	16,627	6,273	3,383
V2	50	19,372	4,966	15,890	5,937	3,263
V2	50	18,949	4,912	15,778	6,031	3,142
V2	50	18,198	4,814	15,325	5,841	3,115
V2	50	18,319	4,830	15,447	5,786	3,166
V2	50	17,267	4,689	14,944	5,571	3,100

V2	50	19,711	5,010	16,048	6,058	3,254
V2	50	16,722	4,614	14,685	5,619	2,976
V2	50	18,295	4,826	15,361	5,772	3,170
V2	50	20,570	5,118	16,307	6,264	3,284
V2	50	19,941	5,039	16,073	6,071	3,285
V2	50	20,062	5,054	16,073	5,850	3,429
V2	50	20,183	5,069	16,221	6,087	3,316
V2	50	19,663	5,004	16,023	5,927	3,317
V2	50	19,082	4,929	15,926	6,071	3,143
V2	50	18,695	4,879	15,631	5,917	3,159
V2	50	19,892	5,033	16,037	5,967	3,334
V2	50	20,401	5,097	16,393	6,237	3,271
V2	50	18,114	4,802	15,310	5,552	3,263
V2	50	21,635	5,248	16,688	6,210	3,484
V2	50	17,267	4,689	14,919	5,683	3,038
V2	50	16,855	4,633	14,857	5,676	2,969
V2	50	18,767	4,888	15,646	5,787	3,243
V2	50	19,529	4,987	15,717	5,917	3,300
V2	50	19,154	4,938	15,631	5,673	3,376
V2	50	16,819	4,628	14,664	5,273	3,189
V2	50	18,162	4,809	15,351	5,661	3,208
V2	50	17,630	4,738	15,163	5,580	3,159
V2	50	19,336	4,962	15,839	5,956	3,247
V2	50	20,183	5,069	16,063	5,972	3,380
V2	50	17,775	4,757	15,102	5,580	3,185
V2	50	18,404	4,841	15,336	5,749	3,201
V2	50	18,561	4,861	15,498	5,904	3,144
V2	50	17,243	4,685	15,041	5,552	3,106
V2	50	17,351	4,700	15,056	5,691	3,049
V2	50	16,577	4,594	15,041	5,819	2,849
V2	50	21,441	5,225	16,688	6,311	3,398
V2	50	20,606	5,122	16,429	6,111	3,372
V2	50	18,634	4,871	15,742	6,011	3,100
V2	50	19,868	5,030	16,124	6,073	3,272
V2	50	18,912	4,907	15,544	5,581	3,389
V2	50	18,319	4,830	15,483	5,717	3,204
V2	50	20,800	5,146	16,688	6,363	3,269
V2	50	19,396	4,970	15,987	6,006	3,230
V2	50	20,340	5,089	16,160	5,934	3,428
V2	50	19,469	4,979	15,803	5,880	3,311
V2	50	19,735	5,013	15,926	5,993	3,293
V2	50	20,594	5,121	16,282	6,064	3,396
V2	50	20,631	5,125	16,455	6,363	3,242
V2	50	20,389	5,095	16,404	5,977	3,411
V2	50	19,469	4,979	15,742	5,922	3,288
V2	50	20,243	5,077	16,195	6,005	3,371
V2	50	21,671	5,253	16,699	6,237	3,474
V2	50	18,090	4,799	15,228	5,507	3,285
V2	50	17,364	4,702	15,152	5,825	2,981
V2	50	18,876	4,902	15,473	5,744	3,286
V2	50	19,457	4,977	15,915	5,881	3,308
V2	50	15,222	4,402	14,135	5,090	2,990

V2	50	18,610	4,868	15,620	5,787	3,216
V2	50	19,166	4,940	15,717	5,764	3,325
V2	50	18,029	4,791	15,275	5,937	3,037
V2	50	17,376	4,704	15,030	5,672	3,063
V2	50	18,077	4,798	15,483	5,890	3,069
V2	50	18,912	4,907	15,829	6,030	3,136
V2	50	19,324	4,960	16,124	6,147	3,143
V2	50	19,614	4,997	15,951	5,963	3,289
V2	50	20,013	5,048	16,404	6,071	3,297
V2	50	18,356	4,834	15,509	5,871	3,126
V2	50	22,070	5,301	17,080	6,447	3,423
V2	50	21,018	5,173	16,368	6,162	3,411
V2	50	20,667	5,130	16,638	6,487	3,186
V2	50	18,186	4,812	15,498	5,877	3,095
V2	50	19,106	4,932	16,098	6,118	3,123
V2	50	18,695	4,879	15,570	5,812	3,216
V2	50	21,768	5,265	16,785	6,157	3,535
V2	50	18,924	4,909	15,717	6,087	3,109
V2	50	17,956	4,782	15,178	5,748	3,124
V2	50	17,690	4,746	15,264	5,634	3,140
V2	50	18,307	4,828	15,239	5,598	3,271
V2	50	18,840	4,898	15,595	5,697	3,307
V2	50	18,888	4,904	15,605	5,786	3,264
V2	50	18,489	4,852	15,473	5,852	3,160
V2	50	18,985	4,917	15,595	5,716	3,321
V2	50	19,892	5,033	16,012	5,913	3,364
V2	65	18,126	4,804	15,498	5,994	3,024
V2	65	17,860	4,769	15,214	5,609	3,184
V2	65	20,873	5,155	16,444	6,168	3,384
V2	65	18,453	4,847	15,437	5,726	3,223
V2	65	19,142	4,937	15,829	6,003	3,189
V2	65	17,642	4,739	15,275	5,867	3,007
V2	65	19,033	4,923	15,692	5,772	3,298
V2	65	16,311	4,557	14,552	5,626	2,899
V2	65	17,751	4,754	15,178	5,719	3,104
V2	65	18,090	4,799	15,422	5,825	3,105
V2	65	19,735	5,013	16,124	5,977	3,302
V2	65	17,811	4,762	15,239	5,872	3,033
V2	65	19,638	5,000	16,012	6,021	3,262
V2	65	16,952	4,646	14,807	5,446	3,113
V2	65	20,134	5,063	16,185	6,157	3,270
V2	65	17,243	4,685	15,005	5,502	3,134
V2	65	18,017	4,790	15,300	5,889	3,059
V2	65	19,880	5,031	16,109	6,034	3,295
V2	65	18,465	4,849	15,498	5,882	3,139
V2	65	18,332	4,831	15,275	5,676	3,229
V2	65	17,920	4,777	15,361	5,922	3,026
V2	65	19,844	5,027	16,098	6,117	3,244
V2	65	21,405	5,220	16,638	6,170	3,469
V2	65	18,501	4,853	15,239	5,683	3,256
V2	65	19,626	4,999	15,976	6,011	3,265
V2	65	18,307	4,828	15,584	5,940	3,082

V2	65	18,695	4,879	15,584	5,825	3,209
V2	65	18,598	4,866	15,534	5,772	3,222
V2	65	19,360	4,965	15,854	6,017	3,217
V2	65	18,670	4,876	15,534	5,871	3,180
V2	65	19,045	4,924	15,768	6,003	3,172
V2	65	18,259	4,822	15,386	5,871	3,110
V2	65	18,767	4,888	15,498	5,619	3,340
V2	65	18,646	4,872	15,793	6,003	3,106
V2	65	18,428	4,844	15,386	5,825	3,164
V2	65	17,509	4,722	14,995	5,486	3,192
V2	65	19,735	5,013	16,195	6,222	3,172
V2	65	19,880	5,031	16,098	5,940	3,347
V2	65	18,271	4,823	15,361	5,911	3,091
V2	65	20,280	5,081	16,393	6,336	3,200
V2	65	16,710	4,613	14,466	5,298	3,154
V2	65	18,065	4,796	15,519	5,812	3,108
V2	65	17,521	4,723	15,005	5,637	3,108
V2	65	20,171	5,068	16,271	6,137	3,287
V2	65	17,509	4,722	15,127	5,619	3,116
V2	65	18,428	4,844	15,300	5,711	3,227
V2	65	17,581	4,731	15,188	5,629	3,123
V2	65	19,820	5,023	16,379	6,030	3,287
V2	65	18,453	4,847	15,473	5,751	3,209
V2	65	20,364	5,092	16,307	6,222	3,273
V2	65	18,586	4,865	15,814	5,889	3,156
V2	65	19,275	4,954	15,742	5,955	3,237
V2	65	17,436	4,712	14,832	5,580	3,125
V2	65	18,755	4,887	15,584	5,716	3,281
V2	65	21,393	5,219	16,638	6,062	3,529
V2	65	18,307	4,828	15,422	5,725	3,198
V2	65	21,526	5,235	16,897	6,303	3,415
V2	65	20,764	5,142	16,577	6,137	3,383
V2	65	18,755	4,887	15,778	6,030	3,110
V2	65	20,425	5,100	16,490	6,083	3,358
V2	65	17,218	4,682	14,919	5,731	3,004
V2	65	23,801	5,505	17,940	6,961	3,419
V2	65	19,844	5,027	16,160	6,257	3,172
V2	65	19,554	4,990	15,976	6,112	3,199
V2	65	17,388	4,705	15,041	5,579	3,117
V2	65	19,650	5,002	15,926	5,764	3,409
V2	65	18,719	4,882	15,656	5,993	3,123
V2	65	17,206	4,681	15,285	5,940	2,897
V2	65	18,501	4,853	15,570	5,870	3,152
V2	65	21,115	5,185	16,480	6,211	3,400
V2	65	20,401	5,097	16,134	6,021	3,388
V2	65	19,106	4,932	15,829	6,032	3,168
V2	65	19,905	5,034	15,987	6,021	3,306
V2	65	19,868	5,030	16,124	6,134	3,239
V2	65	18,840	4,898	15,656	6,011	3,134
V2	65	19,771	5,017	16,037	6,032	3,277
V2	65	19,396	4,970	15,692	5,849	3,316
V2	65	21,090	5,182	16,566	6,222	3,390

V2	65	19,215	4,946	15,742	6,064	3,168
V2	65	19,554	4,990	15,854	5,877	3,327
V2	65	20,739	5,139	16,699	6,057	3,424
V2	65	18,126	4,804	15,325	5,572	3,253
V2	65	19,517	4,985	15,854	5,814	3,357
V2	65	19,021	4,921	15,605	5,743	3,312
V2	65	20,086	5,057	16,073	6,031	3,331
V2	65	17,714	4,749	15,127	5,748	3,082
V2	65	21,502	5,232	16,663	6,359	3,382
V2	65	19,421	4,973	15,742	5,871	3,308
V2	65	19,856	5,028	15,987	5,807	3,420
V2	65	17,944	4,780	15,127	5,417	3,312
V2	65	21,647	5,250	17,019	6,427	3,368
V2	65	18,186	4,812	15,412	5,827	3,121
V2	65	18,755	4,887	15,483	5,777	3,247
V2	65	18,912	4,907	15,681	6,018	3,143
V2	65	18,525	4,857	15,523	5,719	3,239
V2	65	19,796	5,020	16,012	5,904	3,353
V2	65	21,974	5,289	16,724	6,318	3,478
V2	65	17,835	4,765	15,214	5,825	3,062
V2	65	20,606	5,122	16,429	6,403	3,218
V2	65	21,720	5,259	16,897	6,304	3,446
V2	65	20,655	5,128	16,440	6,172	3,347
V2	65	20,038	5,051	16,124	6,111	3,279
V2	65	17,666	4,743	15,117	5,772	3,061
V2	65	18,840	4,898	15,890	5,922	3,181
V2	65	21,320	5,210	16,516	6,168	3,457
V2	65	19,082	4,929	15,707	5,772	3,306
V2	65	21,526	5,235	16,785	6,403	3,362
V2	65	22,252	5,323	17,044	6,317	3,523
V2	65	17,170	4,676	14,995	5,691	3,017
V2	65	18,513	4,855	15,534	5,751	3,219
V2	65	19,251	4,951	16,256	5,927	3,248
V2	65	20,328	5,087	16,480	6,400	3,176
V2	65	20,824	5,149	16,638	6,157	3,382
V2	65	19,215	4,946	15,681	5,827	3,298
V2	65	19,469	4,979	15,951	6,032	3,228
V2	65	19,953	5,040	15,987	5,928	3,366
V2	65	19,626	4,999	15,814	5,889	3,333
V2	65	20,752	5,140	16,393	6,223	3,335
V2	65	18,259	4,822	15,214	5,726	3,189
V2	65	19,009	4,920	15,631	5,749	3,307
V2	65	18,453	4,847	15,372	5,580	3,307
V2	65	17,835	4,765	14,944	5,519	3,231
V2	65	16,420	4,572	14,476	5,297	3,100
V2	65	17,727	4,751	15,336	5,870	3,020
V2	65	19,058	4,926	15,803	6,087	3,131
V2	65	19,191	4,943	15,976	6,032	3,181
V2	65	19,542	4,988	16,023	5,899	3,312
V2	65	18,973	4,915	15,829	6,067	3,127
V2	65	20,848	5,152	16,455	6,157	3,386
V2	65	16,940	4,644	14,796	5,523	3,067

V2	65	16,916	4,641	14,649	5,645	2,997
V2	65	19,856	5,028	15,987	6,096	3,257
V2	65	18,961	4,913	15,778	6,003	3,158
V2	65	20,340	5,089	16,343	6,199	3,281
V2	65	18,428	4,844	15,559	5,967	3,088
V2	65	18,344	4,833	15,534	5,786	3,170
V2	65	19,905	5,034	15,865	5,890	3,380
V2	65	20,376	5,094	16,332	6,247	3,262
V2	65	19,844	5,027	16,210	6,128	3,238
V2	65	19,747	5,014	15,976	5,922	3,335
V2	65	18,392	4,839	15,473	5,760	3,193
V2	65	19,880	5,031	15,997	6,018	3,304
V2	65	20,389	5,095	16,256	5,953	3,425
V2	65	20,691	5,133	16,419	6,183	3,347
V2	65	19,917	5,036	15,976	6,017	3,310
V2	65	20,800	5,146	16,663	6,454	3,223
V2	65	19,965	5,042	16,037	6,058	3,296
V2	65	19,542	4,988	15,753	5,825	3,355
V2	80	19,965	5,042	16,073	6,140	3,252
V2	80	21,260	5,203	16,516	6,140	3,463
V2	80	20,945	5,164	16,516	6,140	3,411
V1	80	19,929	5,037	16,134	6,140	3,246
V1	80	19,529	4,987	15,987	6,140	3,181
V1	80	21,707	5,257	16,663	6,140	3,535
V2	80	20,836	5,151	16,465	6,140	3,393
V2	80	20,764	5,142	16,404	6,140	3,382
V2	80	20,389	5,095	16,307	6,140	3,320
V2	80	21,332	5,212	16,566	6,147	3,470
V2	80	20,873	5,155	16,419	6,147	3,395
V1	80	20,510	5,110	16,246	6,147	3,336
V1	80	20,364	5,092	16,307	6,147	3,313
V2	80	22,155	5,311	17,177	6,148	3,504
V1	80	21,127	5,186	16,541	6,148	3,437
V1	80	20,195	5,071	16,332	6,148	3,285
V2	80	19,868	5,030	16,012	6,148	3,232
V2	80	19,759	5,016	16,124	6,148	3,214
V1	80	19,251	4,951	15,915	6,148	3,131
V2	80	21,381	5,218	16,760	6,151	3,476
V2	80	20,691	5,133	16,429	6,151	3,364
V2	80	20,570	5,118	16,404	6,151	3,344
V1	80	20,364	5,092	16,282	6,151	3,311
V1	80	20,364	5,092	16,282	6,151	3,311
V2	80	19,663	5,004	16,195	6,151	3,197
V2	80	21,344	5,213	16,699	6,151	3,470
V2	80	20,497	5,109	16,368	6,151	3,332
V1	80	21,332	5,212	16,602	6,156	3,465
V2	80	21,115	5,185	16,455	6,156	3,430
V2	80	20,631	5,125	16,627	6,156	3,351
V2	80	20,062	5,054	16,271	6,156	3,259
V2	80	19,554	4,990	16,185	6,156	3,176
V2	80	18,670	4,876	15,742	6,156	3,033
V1	80	20,691	5,133	16,480	6,157	3,361

V1	80	19,784	5,019	16,124	6,157	3,213
V1	80	20,086	5,057	16,160	6,157	3,262
V2	80	21,381	5,218	16,602	6,157	3,473
V2	80	20,836	5,151	16,307	6,157	3,384
V2	80	20,147	5,065	16,124	6,157	3,272
V1	80	19,711	5,010	16,048	6,157	3,201
V1	80	19,650	5,002	16,170	6,157	3,191
V1	80	21,332	5,212	16,541	6,162	3,462
V1	80	21,139	5,188	16,505	6,162	3,431
V1	80	20,836	5,151	16,393	6,162	3,382
V1	80	19,118	4,934	15,987	6,162	3,103
V2	80	21,248	5,201	16,490	6,162	3,448
V1	80	20,957	5,166	16,429	6,162	3,401
V1	80	20,461	5,104	16,282	6,162	3,320
V2	80	20,449	5,103	16,429	6,162	3,318
V1	80	21,223	5,198	16,455	6,164	3,443
V2	80	21,211	5,197	16,429	6,164	3,441
V2	80	21,211	5,197	16,429	6,164	3,441
V2	80	20,086	5,057	16,195	6,164	3,259
V2	80	19,239	4,949	15,865	6,164	3,121
V1	80	19,844	5,027	16,221	6,164	3,219
V2	80	20,534	5,113	16,271	6,164	3,331
V2	80	20,074	5,056	16,063	6,164	3,256
V2	80	19,856	5,028	16,124	6,164	3,221
V2	80	20,328	5,087	16,307	6,167	3,296
V2	80	20,280	5,081	16,221	6,167	3,288
V1	80	20,897	5,158	16,541	6,167	3,388
V2	80	20,316	5,086	16,307	6,167	3,294
V2	80	20,243	5,077	16,246	6,167	3,282
V2	80	20,147	5,065	16,160	6,167	3,267
V2	80	20,110	5,060	16,124	6,167	3,261
V2	80	19,372	4,966	15,951	6,167	3,141
V1	80	18,223	4,817	15,717	6,167	2,955
V1	80	20,376	5,094	16,246	6,168	3,304
V1	80	19,626	4,999	16,098	6,168	3,182
V1	80	20,897	5,158	16,490	6,168	3,388
V2	80	20,824	5,149	16,404	6,168	3,376
V2	80	20,752	5,140	16,343	6,168	3,364
V2	80	20,159	5,066	16,256	6,168	3,268
V1	80	21,683	5,254	16,735	6,168	3,515
V1	80	21,066	5,179	16,612	6,168	3,415
V2	80	20,485	5,107	16,379	6,168	3,321
V1	80	20,969	5,167	16,551	6,170	3,399
V1	80	20,715	5,136	16,429	6,170	3,358
V1	80	20,159	5,066	16,256	6,170	3,267
V2	80	21,574	5,241	16,785	6,170	3,497
V2	80	21,574	5,241	16,785	6,170	3,497
V1	80	21,659	5,251	16,760	6,170	3,510
V1	80	21,151	5,189	16,490	6,170	3,428
V2	80	21,030	5,175	16,551	6,170	3,408
V1	80	21,199	5,195	16,541	6,171	3,435
V1	80	21,151	5,189	16,541	6,171	3,427

V1	80	21,115	5,185	16,551	6,171	3,421
V2	80	20,957	5,166	16,429	6,171	3,396
V1	80	20,836	5,151	16,429	6,171	3,376
V2	80	20,715	5,136	16,332	6,171	3,357
V1	80	20,195	5,071	16,246	6,171	3,272
V1	80	21,344	5,213	16,541	6,171	3,459
V2	80	21,054	5,178	16,429	6,171	3,412
V2	80	21,006	5,172	16,490	6,171	3,404
V2	80	20,606	5,122	16,307	6,171	3,339
V2	80	20,376	5,094	16,246	6,171	3,302
V1	80	20,122	5,062	16,160	6,171	3,261
V1	80	20,001	5,046	16,124	6,171	3,241
V2	80	21,478	5,229	16,699	6,172	3,480
V1	80	20,921	5,161	16,526	6,172	3,390
V2	80	20,921	5,161	16,368	6,172	3,390
V2	80	20,727	5,137	16,368	6,172	3,358
V1	80	20,570	5,118	16,307	6,172	3,333
V2	80	20,546	5,115	16,221	6,172	3,329
V1	80	22,034	5,297	16,724	6,182	3,564
V2	80	20,389	5,095	16,271	6,182	3,298
V2	80	22,288	5,327	16,861	6,182	3,505
V2	80	21,187	5,194	16,602	6,182	3,427
V2	80	20,800	5,146	16,393	6,182	3,364
V1	80	20,401	5,097	16,307	6,182	3,300
V2	80	20,340	5,089	16,297	6,182	3,290
V2	80	20,280	5,081	16,185	6,182	3,280
V2	80	20,038	5,051	16,297	6,182	3,241
V2	80	19,650	5,002	15,951	6,182	3,179
V2	80	21,623	5,247	16,724	6,183	3,497
V1	80	21,054	5,178	16,480	6,183	3,405
V2	80	21,018	5,173	16,480	6,183	3,399
V2	80	20,921	5,161	16,480	6,183	3,383
V2	80	20,739	5,139	16,480	6,183	3,354
V1	80	20,703	5,134	16,444	6,183	3,348
V2	80	20,207	5,072	16,185	6,183	3,268
V2	80	20,836	5,151	16,480	6,184	3,370
V2	80	21,647	5,250	16,749	6,184	3,501
V2	80	20,800	5,146	16,332	6,184	3,364
V2	80	20,522	5,112	16,358	6,184	3,319
V2	80	19,408	4,971	16,037	6,184	3,139
V2	80	22,010	5,294	16,933	6,189	3,556
V2	80	21,211	5,197	16,674	6,189	3,427
V1	80	21,175	5,192	16,612	6,189	3,421
V2	80	21,151	5,189	16,490	6,189	3,417
V2	80	22,083	5,302	16,811	6,190	3,568
V2	80	21,659	5,251	16,749	6,190	3,499
V2	80	21,417	5,222	16,638	6,190	3,460
V2	80	21,078	5,181	16,674	6,190	3,405
V2	80	20,340	5,089	16,318	6,190	3,286
V2	80	21,429	5,223	16,638	6,197	3,458
V2	80	21,139	5,188	16,551	6,197	3,411
V2	80	20,618	5,124	16,429	6,197	3,327

V2	80	20,110	5,060	16,160	6,197	3,245
V2	80	20,134	5,063	16,282	6,197	3,249
V1	80	21,974	5,289	16,872	6,197	3,546
V1	80	21,296	5,207	16,648	6,197	3,436
V2	80	20,860	5,154	16,393	6,197	3,366
V1	80	20,752	5,140	16,551	6,197	3,348
V2	80	21,974	5,289	16,724	6,199	3,545
V1	80	21,720	5,259	16,688	6,199	3,504
V1	80	21,260	5,203	16,602	6,199	3,429
V2	80	21,187	5,194	16,490	6,199	3,418
V2	80	20,981	5,169	16,551	6,199	3,384
V2	80	20,800	5,146	16,455	6,199	3,355
V1	80	20,546	5,115	16,368	6,199	3,314
V2	80	20,425	5,100	16,246	6,199	3,295
V1	80	20,352	5,091	16,185	6,199	3,283
V1	80	20,340	5,089	16,343	6,199	3,281
V2	80	20,328	5,087	16,282	6,199	3,279
V1	80	20,050	5,053	16,195	6,199	3,234
V1	80	19,784	5,019	16,160	6,199	3,191
V1	80	19,287	4,956	15,865	6,199	3,111
V1	80	19,033	4,923	16,098	6,200	3,070
V1	80	21,078	5,181	16,332	6,200	3,400
V1	80	20,655	5,128	16,343	6,200	3,331
V2	80	20,074	5,056	16,256	6,200	3,238
V2	80	22,204	5,317	16,775	6,207	3,577
V2	80	21,163	5,191	16,480	6,207	3,409
V1	80	20,945	5,164	16,480	6,207	3,374
V2	80	20,860	5,154	16,429	6,208	3,360
V2	80	20,364	5,092	16,256	6,208	3,280
V1	80	19,880	5,031	16,012	6,208	3,203
V1	80	21,502	5,232	16,627	6,208	3,463
V2	80	19,590	4,994	16,012	6,208	3,155
V1	80	20,885	5,157	16,419	6,210	3,363
V1	80	19,626	4,999	16,124	6,210	3,160
V2	80	21,623	5,247	16,663	6,210	3,482
V1	80	20,727	5,137	16,332	6,210	3,338
V1	80	20,618	5,124	16,368	6,210	3,320
V2	80	20,570	5,118	16,368	6,210	3,312
V2	80	20,268	5,080	16,221	6,210	3,264
V2	80	19,421	4,973	15,987	6,210	3,127
V2	80	22,107	5,305	16,872	6,211	3,560
V1	80	21,054	5,178	16,429	6,211	3,390
V1	80	19,905	5,034	16,124	6,211	3,205
V1	80	20,836	5,151	16,455	6,211	3,355
Control	Control	16,275	4,552	14,624	5,311	3,064
Control	Control	18,404	4,841	15,336	5,731	3,211
Control	Control	16,226	4,545	14,451	5,290	3,067
Control	Control	20,582	5,119	16,358	6,057	3,398
Control	Control	18,622	4,869	15,300	5,579	3,338
Control	Control	19,771	5,017	15,890	5,977	3,308
Control	Control	20,643	5,127	16,246	5,956	3,466
Control	Control	17,811	4,762	15,127	5,581	3,192

Control	Control	20,098	5,059	16,037	5,967	3,368
Control	Control	20,074	5,056	16,109	5,953	3,372
Control	Control	20,752	5,140	16,419	6,066	3,421
Control	Control	19,191	4,943	15,620	5,772	3,325
Control	Control	18,416	4,842	15,422	5,658	3,255
Control	Control	19,747	5,014	15,926	5,870	3,364
Control	Control	19,977	5,043	16,124	6,032	3,312
Control	Control	19,856	5,028	16,012	6,021	3,298
Control	Control	22,603	5,365	17,217	6,585	3,432
Control	Control	20,231	5,075	16,210	6,211	3,258
Control	Control	22,252	5,323	16,861	6,237	3,567
Control	Control	21,514	5,234	16,627	6,071	3,544
Control	Control	20,098	5,059	16,012	5,849	3,436
Control	Control	20,909	5,160	16,393	6,147	3,401
Control	Control	19,614	4,997	15,961	5,827	3,366
Control	Control	19,977	5,043	16,037	5,827	3,428
Control	Control	17,799	4,761	15,056	5,526	3,221
Control	Control	19,687	5,007	15,900	5,711	3,447
Control	Control	18,779	4,890	15,509	5,827	3,223
Control	Control	20,098	5,059	16,002	5,977	3,363
Control	Control	19,844	5,027	15,900	5,777	3,435
Control	Control	19,542	4,988	15,865	5,882	3,322
Control	Control	18,900	4,906	15,605	5,736	3,295
Control	Control	22,700	5,376	17,055	6,303	3,602
Control	Control	24,987	5,640	18,408	6,977	3,581
Control	Control	22,930	5,403	17,462	6,575	3,487
Control	Control	19,626	4,999	16,012	5,953	3,297
Control	Control	21,804	5,269	16,983	6,184	3,526
Control	Control	20,425	5,100	16,231	5,927	3,446
Control	Control	22,119	5,307	16,907	6,189	3,574
Control	Control	20,425	5,100	16,231	5,927	3,446
Control	Control	22,119	5,307	16,907	6,189	3,574
Control	Control	19,602	4,996	15,987	5,918	3,313
Control	Control	20,413	5,098	16,185	5,977	3,415
Control	Control	20,219	5,074	16,343	6,170	3,277
Control	Control	18,682	4,877	15,534	5,672	3,294
Control	Control	18,949	4,912	15,768	5,967	3,176
Control	Control	19,493	4,982	15,926	5,845	3,335
Control	Control	19,287	4,956	15,768	5,904	3,267
Control	Control	16,456	4,577	14,415	5,311	3,099
Control	Control	21,478	5,229	16,969	6,265	3,428
Control	Control	19,578	4,993	15,865	6,032	3,246
Control	Control	20,582	5,119	16,393	6,199	3,320
Control	Control	19,118	4,934	15,681	5,904	3,238
Control	Control	18,985	4,917	15,656	5,956	3,188
Control	Control	17,690	4,746	15,214	5,776	3,063
Control	Control	19,384	4,968	16,023	6,118	3,169
Control	Control	20,667	5,130	16,368	6,134	3,369
Control	Control	19,590	4,994	16,012	6,162	3,179
Control	Control	19,033	4,923	15,753	5,821	3,270
Control	Control	20,510	5,110	16,343	6,208	3,304
Control	Control	21,635	5,248	16,724	6,162	3,511

Control	Control	18,259	4,822	15,559	5,904	3,092
Control	Control	20,292	5,083	16,246	6,171	3,288
Control	Control	19,626	4,999	15,926	5,890	3,332
Control	Control	19,215	4,946	15,793	5,731	3,353
Control	Control	19,784	5,019	16,124	6,127	3,229
Control	Control	22,179	5,314	16,958	6,363	3,486
Control	Control	19,493	4,982	15,778	5,764	3,382
Control	Control	18,561	4,861	15,447	5,772	3,216
Control	Control	19,771	5,017	16,012	5,984	3,304
Control	Control	20,461	5,104	16,195	5,872	3,485
Control	Control	19,735	5,013	15,976	6,018	3,280
Control	Control	18,634	4,871	15,422	5,580	3,340
Control	Control	18,029	4,791	15,473	5,882	3,065
Control	Control	20,098	5,059	16,048	5,870	3,424
Control	Control	19,880	5,031	15,987	5,964	3,333
Control	Control	18,501	4,853	15,509	6,030	3,068
Control	Control	20,558	5,116	16,526	6,358	3,233
Control	Control	19,626	4,999	16,221	6,112	3,211
Control	Control	18,658	4,874	15,742	5,977	3,122
Control	Control	19,590	4,994	15,865	5,956	3,289
Control	Control	21,187	5,194	16,577	6,064	3,494
Control	Control	21,877	5,278	16,933	6,222	3,516
Control	Control	19,094	4,931	15,926	5,977	3,195
Control	Control	18,598	4,866	15,570	5,877	3,165
Control	Control	21,090	5,182	16,785	6,297	3,349
Control	Control	19,989	5,045	16,134	6,112	3,270
Control	Control	20,945	5,164	16,343	5,927	3,534
Control	Control	19,082	4,929	15,717	5,918	3,225
Control	Control	16,843	4,631	14,832	5,417	3,109
Control	Control	18,077	4,798	15,325	5,772	3,132
Control	Control	19,360	4,965	15,742	5,927	3,266
Control	Control	18,392	4,839	15,631	6,034	3,048
Control	Control	20,376	5,094	16,271	5,913	3,446
Control	Control	17,085	4,664	14,761	5,566	3,069
Control	Control	18,949	4,912	15,534	5,672	3,341
Control	Control	19,179	4,942	15,656	5,849	3,279
Control	Control	20,897	5,158	16,307	6,172	3,386
Control	Control	18,126	4,804	15,178	5,731	3,163
Control	Control	19,699	5,008	15,987	5,956	3,307
Control	Control	20,715	5,136	16,221	6,111	3,390
Control	Control	21,066	5,179	16,602	6,318	3,334
Control	Control	22,325	5,331	17,044	6,430	3,472
Control	Control	15,331	4,418	13,973	5,184	2,957
Control	Control	19,033	4,923	15,768	5,913	3,219
Control	Control	17,630	4,738	15,066	5,519	3,194
Control	Control	20,098	5,059	16,012	5,967	3,368
Control	Control	18,864	4,901	15,559	5,691	3,315
Control	Control	19,856	5,028	15,926	5,918	3,355
Control	Control	18,755	4,887	15,534	5,725	3,276
Control	Control	20,026	5,049	16,185	6,264	3,197
Control	Control	21,102	5,183	16,490	6,112	3,452
Control	Control	18,235	4,818	15,275	5,686	3,207

Control	Control	18,586	4,865	15,412	5,772	3,220
Control	Control	19,868	5,030	16,002	5,936	3,347
Control	Control	19,796	5,020	16,012	6,058	3,268
Control	Control	19,590	4,994	15,951	5,967	3,283
Control	Control	17,593	4,733	15,117	5,724	3,074
Control	Control	25,616	5,711	17,818	5,937	4,315
Control	Control	19,481	4,980	15,890	5,913	3,294
Control	Control	19,711	5,010	15,839	5,871	3,357
Control	Control	20,933	5,163	16,393	6,112	3,425
Control	Control	21,720	5,259	16,663	6,199	3,504
Control	Control	21,211	5,197	16,429	6,171	3,437
Control	Control	17,920	4,777	15,178	5,527	3,242
Control	Control	20,425	5,100	16,419	6,067	3,367
Control	Control	20,171	5,068	16,210	6,057	3,330
Control	Control	19,215	4,946	15,886	5,818	3,302
Control	Control	19,856	5,028	16,012	6,127	3,241
Control	Control	19,263	4,952	15,854	5,867	3,283
Control	Control	19,505	4,983	15,854	5,882	3,316
Control	Control	20,001	5,046	16,124	5,955	3,359
Control	Control	21,586	5,243	16,663	6,340	3,405
Control	Control	18,501	4,853	15,361	5,626	3,288
Control	Control	19,324	4,960	15,839	5,744	3,364
Control	Control	18,852	4,899	15,768	5,904	3,193
V1	50	18,924	4,909	15,976	5,972	3,169
V1	50	19,711	5,010	15,865	5,993	3,289
V1	50	16,746	4,618	14,771	5,532	3,027
V1	50	19,239	4,949	15,753	5,889	3,267
V1	50	20,631	5,125	16,246	5,912	3,490
V1	50	18,610	4,868	15,509	5,638	3,301
V1	50	21,223	5,198	16,699	6,259	3,391
V1	50	19,384	4,968	15,865	5,971	3,246
V1	50	19,905	5,034	15,803	5,867	3,393
V1	50	19,469	4,979	16,063	6,093	3,195
V1	50	17,884	4,772	15,300	5,815	3,076
V1	50	21,078	5,181	16,602	6,373	3,308
V1	50	18,767	4,888	15,473	5,787	3,243
V1	50	17,727	4,751	15,239	5,867	3,021
V1	50	19,469	4,979	15,951	6,156	3,162
V1	50	19,191	4,943	16,002	5,937	3,233
V1	50	20,497	5,109	16,393	6,237	3,286
V1	50	18,428	4,844	15,351	5,621	3,278
V1	50	18,779	4,890	15,681	5,881	3,193
V1	50	18,937	4,910	15,559	5,732	3,304
V1	50	19,263	4,952	15,936	6,059	3,179
V1	50	18,852	4,899	15,483	5,776	3,264
V1	50	20,292	5,083	16,160	6,057	3,350
V1	50	22,893	5,399	17,350	6,585	3,477
V1	50	20,727	5,137	16,541	6,140	3,376
V1	50	22,397	5,340	17,131	6,523	3,434
V1	50	20,461	5,104	16,098	5,971	3,427
V1	50	19,929	5,037	16,073	5,821	3,424
V1	50	20,219	5,074	16,195	5,871	3,444

V1	50	21,223	5,198	16,480	6,011	3,531
V1	50	19,820	5,023	15,890	5,918	3,349
V1	50	19,796	5,020	15,951	6,018	3,290
V1	50	19,832	5,025	16,048	5,953	3,331
V1	50	19,481	4,980	15,865	5,993	3,251
V1	50	20,207	5,072	16,073	5,977	3,381
V1	50	19,445	4,976	15,717	5,821	3,340
V1	50	20,836	5,151	16,480	6,183	3,370
V1	50	20,449	5,103	16,160	6,018	3,398
V1	50	19,880	5,031	15,951	5,911	3,363
V1	50	18,937	4,910	15,692	5,715	3,314
V1	50	20,243	5,077	16,185	6,093	3,322
V1	50	18,295	4,826	15,275	5,772	3,169
V1	50	18,029	4,791	15,239	5,737	3,143
V1	50	22,022	5,295	17,228	6,410	3,436
V1	50	21,707	5,257	16,663	6,208	3,497
V1	50	20,873	5,155	16,368	6,096	3,424
V1	50	19,215	4,946	15,778	5,737	3,349
V1	50	19,844	5,027	16,073	5,953	3,333
V1	50	19,856	5,028	15,976	5,937	3,344
V1	50	20,304	5,084	16,246	6,096	3,331
V1	50	18,670	4,876	15,681	5,937	3,145
V1	50	21,102	5,183	16,602	6,310	3,344
V1	50	18,937	4,910	15,768	5,940	3,188
V1	50	19,771	5,017	15,926	6,125	3,228
V1	50	21,526	5,235	17,009	6,514	3,304
V1	50	22,349	5,334	17,044	6,509	3,433
V1	50	18,489	4,852	15,275	5,608	3,297
V1	50	18,961	4,913	15,620	5,787	3,276
V1	50	20,038	5,051	16,124	6,087	3,292
V1	50	18,997	4,918	15,681	5,760	3,298
V1	50	21,744	5,262	16,958	6,406	3,394
V1	50	22,216	5,318	16,907	6,275	3,540
V1	50	20,086	5,057	16,195	6,062	3,313
V1	50	19,808	5,022	16,012	5,918	3,347
V1	50	19,808	5,022	15,940	5,880	3,369
V1	50	20,001	5,046	16,134	5,953	3,360
V1	50	22,627	5,367	17,141	6,259	3,615
V1	50	19,578	4,993	15,951	5,955	3,288
V1	50	17,751	4,754	15,152	5,772	3,075
V1	50	21,102	5,183	16,612	6,222	3,391
V1	50	18,997	4,918	15,570	5,917	3,210
V1	50	19,348	4,963	15,778	5,880	3,291
V1	50	15,028	4,374	13,912	5,119	2,936
V1	50	20,558	5,116	16,393	6,167	3,333
V1	50	18,900	4,906	15,509	5,889	3,209
V1	50	21,962	5,288	16,872	6,221	3,530
V1	50	19,312	4,959	15,839	5,845	3,304
V1	50	18,876	4,902	15,717	5,807	3,251
V1	50	19,868	5,030	15,865	5,818	3,415
V1	50	20,122	5,062	16,134	5,807	3,465
V1	50	21,913	5,282	16,836	6,184	3,544

V1	50	20,062	5,054	16,246	6,087	3,296
V1	50	18,924	4,909	15,605	5,849	3,235
V1	50	21,574	5,241	16,785	6,240	3,457
V1	50	21,514	5,234	16,775	6,237	3,449
V1	50	21,853	5,275	16,821	6,063	3,605
V1	50	20,909	5,160	16,480	6,094	3,431
V1	50	18,223	4,817	15,437	5,786	3,149
V1	50	19,542	4,988	15,915	5,977	3,269
V1	50	20,304	5,084	16,221	5,993	3,388
V1	50	19,578	4,993	15,987	5,826	3,361
V1	50	19,989	5,045	16,037	5,877	3,401
V1	50	18,937	4,910	15,509	5,683	3,332
V1	50	19,965	5,042	16,124	6,147	3,248
V1	50	21,792	5,268	16,897	6,468	3,369
V1	50	20,848	5,152	16,465	6,030	3,457
V1	50	21,707	5,257	16,688	6,275	3,459
V1	50	21,199	5,195	16,551	6,042	3,508
V1	50	20,352	5,091	16,160	6,112	3,330
V1	50	18,307	4,828	15,447	5,760	3,178
V1	50	23,571	5,478	17,217	6,363	3,704
V1	50	18,126	4,804	15,224	5,542	3,271
V1	50	20,594	5,121	16,455	6,183	3,331
V1	50	17,896	4,773	15,300	5,787	3,092
V1	50	18,537	4,858	15,595	5,877	3,154
V1	50	17,944	4,780	15,397	5,821	3,083
V1	50	18,126	4,804	15,361	5,658	3,204
V1	50	20,969	5,167	16,444	6,238	3,362
V1	50	18,332	4,831	15,534	5,842	3,138
V1	50	18,682	4,877	15,768	5,967	3,131
V1	50	18,767	4,888	15,620	5,922	3,169
V1	65	21,054	5,178	16,368	5,904	3,566
V1	65	23,353	5,453	17,462	6,618	3,529
V1	65	23,450	5,464	17,426	6,482	3,617
V1	65	19,481	4,980	15,829	5,786	3,367
V1	65	19,602	4,996	15,753	5,748	3,410
V1	65	21,490	5,231	16,455	6,164	3,486
V1	65	20,655	5,128	16,318	6,030	3,425
V1	65	22,312	5,330	16,811	6,303	3,540
V1	65	22,385	5,339	17,080	6,468	3,461
V1	65	20,703	5,134	16,404	6,237	3,319
V1	65	21,732	5,260	16,872	6,274	3,463
V1	65	21,211	5,197	16,429	6,118	3,467
V1	65	20,788	5,145	16,282	5,880	3,535
V1	65	23,535	5,474	17,436	6,545	3,596
V1	65	20,606	5,122	16,271	6,004	3,432
V1	65	18,416	4,842	15,275	5,580	3,300
V1	65	23,038	5,416	17,167	6,317	3,647
V1	65	20,292	5,083	16,195	5,934	3,420
V1	65	22,494	5,352	17,055	6,331	3,553
V1	65	20,727	5,137	16,393	6,148	3,372
V1	65	17,460	4,715	14,980	5,580	3,129
V1	65	21,187	5,194	16,429	6,083	3,483

V1	65	21,465	5,228	16,749	6,157	3,486
V1	65	21,671	5,253	16,836	6,277	3,452
V1	65	19,892	5,033	16,088	5,977	3,328
V1	65	18,888	4,904	15,544	5,715	3,305
V1	65	20,437	5,101	16,160	5,956	3,431
V1	65	20,691	5,133	16,465	6,006	3,445
V1	65	16,795	4,624	14,466	5,426	3,095
V1	65	22,216	5,318	16,749	6,265	3,546
V1	65	23,873	5,513	17,645	6,428	3,714
V1	65	19,215	4,946	15,681	5,787	3,320
V1	65	20,219	5,074	15,926	5,826	3,471
V1	65	23,801	5,505	17,548	6,454	3,688
V1	65	21,913	5,282	16,836	6,072	3,609
V1	65	22,954	5,406	17,167	6,510	3,526
V1	65	22,119	5,307	16,749	6,237	3,546
V1	65	26,039	5,758	18,433	6,860	3,796
V1	65	24,890	5,629	17,904	6,618	3,761
V1	65	20,389	5,095	16,124	5,918	3,445
V1	65	22,615	5,366	17,177	6,381	3,544
V1	65	23,414	5,460	17,289	6,363	3,679
V1	65	21,078	5,181	16,526	5,934	3,552
V1	65	22,264	5,324	17,156	6,310	3,528
V1	65	23,365	5,454	17,278	6,222	3,755
V1	65	20,243	5,077	16,073	6,113	3,312
V1	65	21,877	5,278	16,724	6,199	3,529
V1	65	22,155	5,311	16,983	6,318	3,507
V1	65	18,489	4,852	15,412	5,636	3,280
V1	65	20,969	5,167	16,282	6,030	3,477
V1	65	21,635	5,248	16,663	6,218	3,480
V1	65	19,130	4,935	15,803	6,071	3,151
V1	65	20,546	5,115	16,221	6,017	3,415
V1	65	20,836	5,151	16,393	6,113	3,409
V1	65	20,739	5,139	16,256	5,917	3,505
V1	65	21,127	5,186	16,516	6,061	3,485
V1	65	20,969	5,167	16,541	6,120	3,426
V1	65	21,320	5,210	16,332	6,018	3,543
V1	65	22,446	5,346	17,106	6,337	3,542
V1	65	19,312	4,959	15,692	5,993	3,222
V1	65	20,594	5,121	16,098	5,899	3,491
V1	65	22,736	5,380	17,080	6,378	3,565
V1	65	25,241	5,669	18,260	6,902	3,657
V1	65	20,449	5,103	16,185	5,922	3,453
V1	65	19,142	4,937	15,646	5,676	3,372
V1	65	21,707	5,257	16,958	6,510	3,334
V1	65	21,768	5,265	16,724	6,264	3,475
V1	65	20,413	5,098	16,185	6,032	3,384
V1	65	20,691	5,133	16,221	5,993	3,452
V1	65	20,897	5,158	16,221	6,072	3,441
V1	65	21,998	5,292	16,821	6,172	3,564
V1	65	21,611	5,246	16,627	6,172	3,502
V1	65	20,957	5,166	16,307	6,005	3,490
V1	65	23,051	5,417	17,228	6,444	3,577

V1	65	21,465	5,228	16,724	6,228	3,447
V1	65	20,376	5,094	16,332	6,093	3,344
V1	65	21,090	5,182	16,541	6,118	3,447
V1	65	22,409	5,342	16,994	6,331	3,539
V1	65	19,602	4,996	15,829	5,772	3,396
V1	65	19,796	5,020	16,012	5,698	3,474
V1	65	21,877	5,278	16,796	6,210	3,523
V1	65	21,066	5,179	16,566	6,128	3,438
V1	65	21,586	5,243	16,760	6,237	3,461
V1	65	20,110	5,060	16,063	5,922	3,396
V1	65	21,163	5,191	16,368	5,965	3,548
V1	65	21,417	5,222	16,638	6,217	3,445
V1	65	21,429	5,223	16,516	6,217	3,447
V1	65	17,315	4,695	14,919	5,581	3,103
V1	65	20,316	5,086	16,185	6,030	3,369
V1	65	18,368	4,836	15,361	5,814	3,159
V1	65	17,981	4,785	15,178	5,683	3,164
V1	65	21,332	5,212	16,688	6,317	3,377
V1	65	18,791	4,891	15,595	5,814	3,232
V1	65	20,752	5,140	16,221	6,030	3,441
V1	65	19,650	5,002	15,951	6,030	3,259
V1	65	20,546	5,115	16,577	6,094	3,372
V1	65	17,485	4,718	14,857	5,474	3,194
V1	65	20,873	5,155	16,332	5,965	3,499
V1	65	20,147	5,065	16,195	5,935	3,395
V1	65	19,009	4,920	15,717	5,852	3,248
V1	65	20,038	5,051	16,048	5,927	3,381
V1	65	21,804	5,269	16,724	6,217	3,507
V1	65	18,271	4,823	15,214	5,446	3,355
V1	65	19,094	4,931	15,605	5,744	3,324
V1	65	18,549	4,860	15,498	5,765	3,217
V1	65	20,679	5,131	16,271	6,064	3,410
V1	65	19,421	4,973	15,753	5,814	3,340
V1	65	20,582	5,119	16,368	6,072	3,390
V1	65	18,803	4,893	15,707	6,018	3,125
V1	65	18,888	4,904	15,336	5,580	3,385
V2	80	20,752	5,140	16,282	6,211	3,341
V1	80	20,691	5,133	16,505	6,211	3,331
V2	80	21,284	5,206	16,429	6,211	3,427
V2	80	20,715	5,136	16,368	6,211	3,335
V1	80	20,461	5,104	16,221	6,211	3,294
V1	80	19,566	4,991	16,098	6,211	3,150
V1	80	20,921	5,161	16,465	6,217	3,365
V1	80	20,703	5,134	16,343	6,217	3,330
V1	80	20,389	5,095	16,368	6,217	3,279
V2	80	20,352	5,091	16,282	6,217	3,274
V1	80	20,304	5,084	16,307	6,217	3,266
V1	80	20,183	5,069	16,160	6,217	3,246
V2	80	19,977	5,043	16,037	6,217	3,213
V1	80	19,892	5,033	16,073	6,217	3,200
V2	80	19,868	5,030	16,160	6,217	3,196
V2	80	21,732	5,260	16,821	6,217	3,495

V2	80	21,066	5,179	16,490	6,217	3,388
V1	80	20,824	5,149	16,368	6,217	3,349
V1	80	22,143	5,310	16,933	6,218	3,561
V2	80	21,369	5,216	16,785	6,218	3,437
V1	80	21,260	5,203	16,638	6,218	3,419
V1	80	21,102	5,183	16,490	6,218	3,394
V2	80	21,030	5,175	16,577	6,218	3,382
V1	80	20,618	5,124	16,465	6,218	3,316
V2	80	20,086	5,057	16,256	6,218	3,231
V2	80	19,602	4,996	16,195	6,218	3,153
V2	80	20,994	5,170	16,551	6,218	3,376
V2	80	20,255	5,078	16,271	6,218	3,258
V2	80	19,239	4,949	15,951	6,218	3,094
V2	80	20,921	5,161	16,638	6,218	3,365
V2	80	20,933	5,163	16,541	6,222	3,365
V2	80	20,788	5,145	16,541	6,222	3,341
V2	80	20,147	5,065	16,358	6,222	3,238
V1	80	21,441	5,225	16,663	6,222	3,446
V1	80	21,429	5,223	16,516	6,222	3,444
V1	80	21,381	5,218	16,566	6,222	3,436
V2	80	20,280	5,081	16,271	6,222	3,259
V1	80	19,203	4,945	16,012	6,222	3,086
V1	80	21,526	5,235	16,714	6,222	3,459
V2	80	21,151	5,189	16,541	6,222	3,399
V2	80	21,042	5,176	16,455	6,222	3,382
V1	80	20,885	5,157	16,516	6,222	3,356
V1	80	20,848	5,152	16,429	6,222	3,350
V2	80	20,268	5,080	16,358	6,222	3,257
V2	80	20,171	5,068	16,271	6,222	3,242
V2	80	19,965	5,042	16,185	6,222	3,209
V2	80	21,078	5,181	16,393	6,223	3,387
V2	80	20,969	5,167	17,080	6,223	3,370
V1	80	20,183	5,069	16,307	6,223	3,243
V2	80	22,252	5,323	16,994	6,227	3,573
V2	80	20,933	5,163	16,551	6,227	3,361
V2	80	20,703	5,134	16,490	6,237	3,319
V1	80	22,772	5,385	17,044	6,237	3,651
V1	80	22,772	5,385	17,044	6,237	3,651
V2	80	21,369	5,216	16,653	6,237	3,426
V2	80	21,175	5,192	16,627	6,237	3,395
V2	80	21,054	5,178	16,602	6,237	3,376
V1	80	20,812	5,148	16,455	6,237	3,337
V1	80	20,304	5,084	16,221	6,237	3,255
V1	80	21,526	5,235	16,811	6,237	3,451
V2	80	21,236	5,200	16,551	6,237	3,404
V2	80	20,449	5,103	16,343	6,237	3,278
V1	80	20,425	5,100	16,404	6,237	3,275
V1	80	20,425	5,100	16,393	6,238	3,274
V1	80	19,675	5,005	16,073	6,238	3,154
V2	80	23,571	5,478	17,533	6,238	3,679
V2	80	21,042	5,176	16,505	6,238	3,373
V2	80	20,679	5,131	16,541	6,238	3,315

V2	80	19,130	4,935	16,002	6,238	3,067
V1	80	20,485	5,107	16,368	6,240	3,283
V1	80	20,631	5,125	16,551	6,240	3,306
V1	80	20,631	5,125	16,551	6,240	3,306
V1	80	21,478	5,229	16,674	6,246	3,439
V1	80	20,945	5,164	16,551	6,246	3,353
V1	80	21,332	5,212	16,699	6,246	3,415
V1	80	21,284	5,206	16,760	6,246	3,408
V2	80	21,199	5,195	16,674	6,246	3,394
V1	80	21,127	5,186	16,490	6,246	3,382
V1	80	21,127	5,186	16,490	6,246	3,382
V1	80	20,945	5,164	16,648	6,246	3,353
V2	80	20,268	5,080	16,429	6,246	3,245
V1	80	21,441	5,225	16,714	6,246	3,433
V1	80	21,332	5,212	16,602	6,246	3,415
V2	80	20,764	5,142	16,455	6,246	3,324
V2	80	19,820	5,023	16,246	6,246	3,173
V2	80	21,030	5,175	16,612	6,247	3,367
V2	80	19,917	5,036	16,318	6,247	3,188
V2	80	20,497	5,109	16,368	6,247	3,281
V1	80	20,473	5,106	16,408	6,247	3,277
V1	80	20,473	5,106	16,419	6,247	3,277
V1	80	20,231	5,075	16,307	6,247	3,239
V1	80	20,231	5,075	16,307	6,247	3,239
V1	80	19,421	4,973	16,124	6,247	3,109
V2	80	21,937	5,285	16,872	6,247	3,512
V2	80	21,211	5,197	16,627	6,247	3,396
V2	80	20,449	5,103	16,480	6,247	3,273
V2	80	21,490	5,231	16,663	6,257	3,435
V1	80	21,248	5,201	16,577	6,257	3,396
V2	80	21,139	5,188	16,516	6,257	3,379
V2	80	20,945	5,164	16,541	6,257	3,348
V1	80	20,449	5,103	16,307	6,257	3,268
V1	80	20,667	5,130	16,566	6,257	3,303
V2	80	19,735	5,013	16,282	6,259	3,153
V1	80	21,562	5,240	16,699	6,260	3,445
V1	80	21,042	5,176	16,490	6,262	3,360
V1	80	20,582	5,119	16,393	6,262	3,287
V2	80	20,268	5,080	16,404	6,262	3,236
V2	80	19,844	5,027	16,109	6,262	3,169
V1	80	21,768	5,265	16,749	6,263	3,476
V2	80	21,127	5,186	16,627	6,263	3,373
V2	80	21,127	5,186	16,577	6,263	3,373
V1	80	20,836	5,151	16,480	6,263	3,327
V1	80	20,268	5,080	16,332	6,263	3,236
V2	80	21,502	5,232	16,602	6,263	3,433
V2	80	20,594	5,121	16,505	6,263	3,288
V1	80	20,304	5,084	16,368	6,263	3,242
V1	80	21,332	5,212	16,627	6,264	3,406
V2	80	20,981	5,169	16,541	6,264	3,350
V2	80	21,308	5,209	16,638	6,264	3,402
V2	80	20,909	5,160	16,455	6,264	3,338

V1	80	21,550	5,238	16,612	6,264	3,440
V2	80	19,977	5,043	16,037	6,264	3,189
V2	80	19,977	5,043	16,037	6,264	3,189
V2	80	19,917	5,036	16,210	6,264	3,179
V1	80	21,139	5,188	16,724	6,265	3,374
V2	80	21,042	5,176	16,516	6,265	3,359
V2	80	20,546	5,115	16,379	6,265	3,279
V2	80	21,393	5,219	16,627	6,265	3,415
V2	80	20,994	5,170	16,455	6,265	3,351
V1	80	20,643	5,127	16,587	6,266	3,295
V1	80	20,909	5,160	16,455	6,273	3,333
V1	80	20,909	5,160	16,455	6,273	3,333
V2	80	20,800	5,146	16,393	6,273	3,316
V2	80	20,437	5,101	16,393	6,273	3,258
V2	80	21,151	5,189	16,602	6,274	3,371
V2	80	20,413	5,098	16,393	6,274	3,254
V2	80	20,401	5,097	16,368	6,274	3,252
V1	80	20,255	5,078	16,653	6,274	3,229
V1	80	22,010	5,294	16,846	6,274	3,508
V2	80	21,792	5,268	17,004	6,274	3,473
V1	80	21,877	5,278	16,821	6,275	3,487
V2	80	21,562	5,240	16,663	6,275	3,436
V2	80	21,357	5,215	16,602	6,275	3,404
V2	80	21,030	5,175	16,541	6,275	3,352
V2	80	20,788	5,145	16,307	6,275	3,313
V1	80	20,752	5,140	16,480	6,275	3,307
V1	80	19,566	4,991	16,134	6,275	3,118
V1	80	20,727	5,137	16,735	6,275	3,303
V1	80	21,151	5,189	16,490	6,275	3,371
V2	80	20,812	5,148	16,429	6,275	3,317
V1	80	22,337	5,333	16,897	6,275	3,560
V1	80	21,853	5,275	16,811	6,275	3,482
V2	80	20,219	5,074	16,221	6,275	3,222
V1	80	21,986	5,291	16,922	6,277	3,502
V1	80	21,744	5,262	16,688	6,277	3,464
V2	80	20,994	5,170	16,566	6,277	3,344
V2	80	20,800	5,146	16,480	6,277	3,313
V2	80	20,534	5,113	16,455	6,277	3,271
V2	80	22,240	5,321	16,958	6,278	3,542
V2	80	22,240	5,321	16,958	6,278	3,542
V1	80	21,550	5,238	16,724	6,278	3,433
V1	80	20,086	5,057	16,271	6,278	3,199
V2	80	22,530	5,356	17,141	6,278	3,589
V2	80	21,030	5,175	16,638	6,278	3,350
V2	80	20,631	5,125	16,429	6,278	3,286
V2	80	21,998	5,292	16,907	6,278	3,504
V2	80	21,683	5,254	16,846	6,278	3,454
V1	80	21,102	5,183	16,551	6,278	3,361
V2	80	20,921	5,161	16,577	6,278	3,332
V2	80	21,865	5,276	16,872	6,279	3,482
V2	80	21,453	5,226	16,785	6,279	3,417
V1	80	21,623	5,247	16,785	6,292	3,437

V2	80	21,465	5,228	16,749	6,292	3,412
V2	80	21,707	5,257	16,760	6,297	3,447
V2	80	21,381	5,218	16,724	6,297	3,395
V2	80	20,401	5,097	16,429	6,297	3,240
V2	80	20,219	5,074	16,404	6,297	3,211
V1	80	21,078	5,181	16,699	6,298	3,347
V2	80	21,586	5,243	16,749	6,300	3,426
V2	80	21,453	5,226	16,602	6,300	3,405
V1	80	21,066	5,179	16,653	6,300	3,344
V1	80	20,522	5,112	16,480	6,300	3,257
V2	80	20,195	5,071	16,368	6,300	3,205
V1	80	20,437	5,101	16,393	6,301	3,244
V2	80	21,308	5,209	16,714	6,301	3,382
V2	80	21,175	5,192	16,541	6,301	3,361
V2	80	21,078	5,181	16,541	6,303	3,344
V2	80	20,885	5,157	16,541	6,303	3,314
V1	80	20,389	5,095	16,404	6,303	3,235
V2	80	21,102	5,183	16,577	6,303	3,348
V1	80	21,925	5,284	16,846	6,303	3,478
V2	80	21,066	5,179	16,627	6,303	3,342
V2	80	20,389	5,095	16,332	6,303	3,235
V2	80	21,296	5,207	16,688	6,304	3,378
V2	80	20,715	5,136	16,393	6,304	3,286
V2	50	15,391	4,427	14,206	5,233	2,941
V2	50	16,262	4,550	14,685	5,580	2,914
V2	50	18,356	4,834	15,509	5,870	3,127
V2	50	16,008	4,515	14,405	5,380	2,975
V2	50	15,597	4,456	14,613	5,445	2,864
V2	50	18,186	4,812	15,570	5,971	3,046
V2	50	18,549	4,860	15,666	5,865	3,163
V2	50	16,396	4,569	14,527	5,363	3,057
V2	50	18,864	4,901	15,509	5,743	3,285
V2	50	19,408	4,971	16,124	6,263	3,099
V2	50	17,255	4,687	15,239	5,786	2,982
V2	50	20,134	5,063	16,185	6,217	3,238
V2	50	17,279	4,690	15,005	5,609	3,081
V2	50	19,045	4,924	15,692	5,764	3,304
V2	50	18,767	4,888	15,692	5,737	3,271
V2	50	20,522	5,112	16,210	5,962	3,442
V2	50	17,412	4,708	15,005	5,479	3,178
V2	50	19,675	5,005	16,195	6,083	3,234
V2	50	22,942	5,405	17,375	6,427	3,570
V2	50	20,413	5,098	16,332	6,087	3,354
V2	50	17,097	4,666	15,239	5,760	2,968
V2	50	19,832	5,025	16,098	5,993	3,309
V2	50	20,195	5,071	16,332	6,057	3,334
V2	50	20,013	5,048	16,210	6,059	3,303
V2	50	22,518	5,355	17,325	6,406	3,515
V2	50	19,348	4,963	16,185	5,911	3,273
V2	50	23,232	5,439	17,558	6,622	3,508
V2	50	20,643	5,127	16,577	6,073	3,399
V2	50	19,989	5,045	16,231	6,111	3,271

V2	50	18,223	4,817	15,666	6,162	2,957
V2	50	17,908	4,775	15,631	5,776	3,100
V2	50	20,473	5,106	16,379	6,182	3,312
V2	50	21,054	5,178	16,602	6,032	3,490
V2	50	20,352	5,091	16,246	5,956	3,417
V2	50	19,868	5,030	16,185	5,967	3,330
V2	50	19,905	5,034	16,037	6,032	3,300
V2	50	19,880	5,031	15,951	5,748	3,458
V2	50	19,239	4,949	15,656	5,955	3,231
V2	50	17,618	4,736	15,544	5,899	2,987
V2	50	18,344	4,833	15,509	5,845	3,138
V2	50	20,219	5,074	16,490	6,094	3,318
V2	50	16,250	4,549	14,771	5,417	3,000
V2	50	19,578	4,993	15,803	5,937	3,298
V2	50	21,163	5,191	16,907	6,378	3,318
V2	50	17,956	4,782	15,214	5,786	3,103
V2	50	19,360	4,965	16,170	6,140	3,153
V2	50	17,146	4,672	15,447	6,134	2,795
V2	50	16,855	4,633	15,056	5,637	2,990
V2	50	15,149	4,392	14,171	5,295	2,861
V2	50	19,239	4,949	15,926	6,030	3,191
V2	50	18,041	4,793	15,422	5,826	3,096
V2	50	16,976	4,649	15,015	5,626	3,018
V2	50	17,860	4,769	15,336	5,725	3,119
V2	50	18,259	4,822	15,534	5,827	3,133
V2	50	18,731	4,884	15,753	5,852	3,201
V2	50	20,606	5,122	16,393	5,995	3,438
V2	50	19,554	4,990	16,048	6,118	3,196
V2	50	19,759	5,016	16,134	5,673	3,483
V2	50	17,122	4,669	15,127	5,446	3,144
V2	50	20,389	5,095	16,368	6,062	3,363
V2	50	16,710	4,613	14,649	5,431	3,077
V2	50	19,082	4,929	15,814	5,899	3,235
V2	50	22,010	5,294	17,141	6,257	3,518
V2	50	19,263	4,952	15,728	5,743	3,354
V2	50	16,601	4,598	14,883	5,571	2,980
V2	50	16,722	4,614	14,796	5,572	3,001
V2	50	16,444	4,576	15,091	5,567	2,954
V2	50	16,311	4,557	14,624	5,502	2,964
V2	65	19,227	4,948	15,681	5,661	3,396
V2	65	19,771	5,017	15,987	6,059	3,263
V2	65	20,727	5,137	16,393	6,237	3,323
V2	65	19,626	4,999	15,742	5,719	3,432
V2	65	21,175	5,192	16,455	6,117	3,461
V2	65	20,873	5,155	16,221	6,030	3,461
V2	65	19,251	4,951	15,814	6,059	3,177
V2	65	19,759	5,016	15,951	5,899	3,350
V2	65	17,630	4,738	15,066	5,533	3,187
V2	65	20,195	5,071	16,185	6,112	3,304
V2	65	18,247	4,820	15,152	5,501	3,317
V2	65	18,622	4,869	15,447	5,772	3,226
V2	65	20,909	5,160	16,429	6,183	3,382

V2	65	20,534	5,113	16,455	6,218	3,302
V2	65	19,203	4,945	15,778	5,899	3,255
V2	65	17,944	4,780	15,412	5,814	3,086
V2	65	19,396	4,970	15,829	5,936	3,267
V2	65	19,856	5,028	16,134	6,111	3,249
V2	65	18,477	4,850	15,509	5,956	3,102
V2	65	19,360	4,965	15,707	5,787	3,345
V2	65	20,352	5,091	16,195	5,977	3,405
V2	65	21,115	5,185	16,526	6,021	3,507
V2	65	19,856	5,028	16,012	6,018	3,300
V2	65	19,868	5,030	16,037	5,977	3,324
V2	65	17,835	4,765	15,249	5,815	3,067
V2	65	19,953	5,040	15,951	5,927	3,366
V2	65	20,425	5,100	16,210	6,087	3,355
V2	65	20,824	5,149	16,465	6,134	3,395
V2	65	21,502	5,232	16,612	6,237	3,447
V2	65	18,900	4,906	15,473	5,609	3,370
V2	65	18,731	4,884	15,544	5,819	3,219
V2	65	20,147	5,065	16,221	6,111	3,297
V2	65	19,917	5,036	16,048	5,956	3,344
V2	65	20,352	5,091	16,368	6,151	3,309
V2	65	16,952	4,646	14,761	5,481	3,093
V2	65	18,767	4,888	15,620	5,787	3,243
V2	65	18,912	4,907	15,544	5,715	3,309
V2	65	21,538	5,237	16,602	6,118	3,521
V2	65	16,238	4,547	14,685	5,772	2,813
V2	65	19,711	5,010	15,976	6,073	3,246
V2	65	19,626	4,999	15,890	5,842	3,360
V2	65	19,166	4,940	15,681	5,827	3,289
V2	65	18,295	4,826	15,376	5,725	3,196
V2	65	21,211	5,197	16,541	6,222	3,409
V2	65	20,255	5,078	16,393	6,222	3,256
V2	65	20,401	5,097	16,073	5,927	3,442
V2	65	19,045	4,924	15,570	5,793	3,288
V2	65	19,554	4,990	15,987	5,984	3,268
V2	65	20,522	5,112	16,393	6,222	3,298
V2	65	18,392	4,839	15,361	5,772	3,186
V2	65	21,683	5,254	16,836	6,372	3,403
V2	65	21,732	5,260	16,836	6,004	3,620
V2	65	20,086	5,057	16,002	5,865	3,425
V2	65	20,425	5,100	16,663	6,364	3,210
V2	65	18,428	4,844	15,509	5,821	3,166
V2	65	21,115	5,185	16,577	6,357	3,321
V2	65	20,945	5,164	16,663	6,373	3,286
V2	65	19,699	5,008	16,037	5,743	3,430
V2	65	18,211	4,815	15,300	5,787	3,147
V2	65	18,211	4,815	15,361	5,786	3,147
V2	65	18,767	4,888	15,631	5,845	3,211
V2	65	18,695	4,879	15,386	5,715	3,271
V2	65	19,929	5,037	15,961	5,927	3,362
V2	65	22,409	5,342	17,131	6,428	3,486
V2	65	19,505	4,983	15,890	5,993	3,255

V2	65	19,118	4,934	15,717	5,993	3,190
V2	65	20,655	5,128	16,343	6,034	3,423
V2	65	21,586	5,243	16,688	6,277	3,439
V2	65	17,654	4,741	15,091	5,772	3,059
V2	65	21,030	5,175	16,602	6,292	3,342
V2	65	19,892	5,033	15,803	5,786	3,438
V2	65	20,631	5,125	16,368	6,190	3,333
V2	65	17,763	4,756	15,239	5,793	3,066
V2	65	21,623	5,247	17,278	6,714	3,220
V2	65	20,437	5,101	16,185	6,017	3,397
V2	65	20,134	5,063	16,210	6,067	3,319
V2	65	20,885	5,157	16,541	6,164	3,388
V2	65	16,795	4,624	14,832	5,609	2,995
V2	65	18,876	4,902	15,681	6,003	3,144
V2	65	18,090	4,799	15,178	5,749	3,147
V2	65	19,457	4,977	15,742	5,825	3,340
V2	65	19,832	5,025	16,012	6,087	3,258
V2	65	18,380	4,838	15,397	5,524	3,328
V2	65	18,549	4,860	15,498	5,751	3,226
V2	65	18,356	4,834	15,509	5,607	3,273
V2	65	18,344	4,833	15,447	5,711	3,212
V2	65	21,127	5,186	16,638	6,170	3,424
V2	65	16,988	4,651	14,771	5,466	3,108
V2	65	19,457	4,977	15,875	5,793	3,359
V2	65	18,852	4,899	15,778	5,928	3,180
V2	65	20,231	5,075	16,185	6,058	3,340
V2	65	20,449	5,103	16,098	5,937	3,445
V2	65	17,642	4,739	15,289	5,867	3,007
V2	65	17,981	4,785	15,214	5,552	3,239
V2	65	19,517	4,985	15,926	5,953	3,279
V2	65	20,364	5,092	16,271	6,003	3,392
V2	65	20,255	5,078	16,271	6,208	3,263
V2	65	19,408	4,971	15,875	5,956	3,259
V2	65	16,686	4,609	14,613	5,363	3,111
V2	65	19,626	4,999	15,890	5,940	3,304
V2	65	21,550	5,238	16,663	6,274	3,435
V2	65	18,065	4,796	15,361	5,717	3,160
V2	65	19,880	5,031	16,134	5,962	3,335
V2	65	20,026	5,049	15,951	5,993	3,342
V2	65	18,090	4,799	15,351	5,661	3,196
V2	65	17,872	4,770	15,203	5,772	3,096
V2	65	19,602	4,996	15,778	5,815	3,371
V2	65	16,952	4,646	14,735	5,526	3,068
V2	65	17,642	4,739	15,030	5,598	3,152
V2	65	18,803	4,893	15,509	5,715	3,290
V2	65	22,978	5,409	17,325	6,565	3,500
V2	65	18,489	4,852	15,422	5,743	3,219
V2	65	20,243	5,077	16,063	6,072	3,334
V2	65	20,679	5,131	16,271	5,995	3,450
V2	65	21,127	5,186	16,490	6,034	3,501
V2	65	19,082	4,929	15,534	5,737	3,326
V2	65	20,413	5,098	16,393	6,237	3,273

V2	65	20,013	5,048	16,012	5,955	3,361
V1	80	20,449	5,103	16,246	6,304	3,244
V1	80	22,264	5,324	17,070	6,309	3,529
V1	80	21,381	5,218	16,775	6,309	3,389
V2	80	19,832	5,025	16,282	6,309	3,143
V2	80	21,877	5,278	16,897	6,310	3,467
V2	80	21,877	5,278	16,897	6,310	3,467
V1	80	21,962	5,288	16,724	6,310	3,480
V1	80	21,913	5,282	16,811	6,310	3,473
V1	80	21,006	5,172	16,602	6,310	3,329
V2	80	20,776	5,143	16,429	6,310	3,293
V1	80	20,147	5,065	16,195	6,310	3,193
V1	80	21,853	5,275	16,821	6,310	3,463
V2	80	21,913	5,282	16,724	6,311	3,472
V2	80	21,357	5,215	16,638	6,311	3,384
V1	80	22,337	5,333	16,897	6,317	3,536
V2	80	21,865	5,276	16,749	6,317	3,461
V1	80	21,550	5,238	16,907	6,317	3,412
V1	80	21,332	5,212	16,638	6,317	3,377
V1	80	21,599	5,244	16,663	6,317	3,419
V1	80	22,107	5,305	16,958	6,318	3,499
V2	80	21,163	5,191	16,516	6,318	3,350
V2	80	20,026	5,049	16,307	6,318	3,170
V2	80	19,287	4,956	16,098	6,318	3,053
V1	80	21,090	5,182	16,663	6,318	3,338
V2	80	20,606	5,122	16,516	6,324	3,259
V1	80	21,913	5,282	16,846	6,324	3,465
V2	80	19,082	4,929	16,012	6,329	3,015
V1	80	22,724	5,379	17,080	6,331	3,589
V2	80	22,228	5,320	17,080	6,331	3,511
V2	80	21,744	5,262	16,760	6,331	3,434
V1	80	21,453	5,226	16,785	6,331	3,388
V2	80	20,643	5,127	16,404	6,331	3,260
V2	80	21,635	5,248	16,821	6,332	3,417
V2	80	22,905	5,400	17,106	6,332	3,517
V2	80	21,901	5,281	16,922	6,332	3,459
V2	80	21,006	5,172	16,602	6,332	3,317
V1	80	21,369	5,216	16,688	6,333	3,374
V2	80	20,860	5,154	16,455	6,333	3,294
V2	80	21,478	5,229	16,714	6,336	3,390
V1	80	20,631	5,125	16,566	6,336	3,256
V2	80	20,885	5,157	16,566	6,337	3,296
V1	80	20,570	5,118	16,480	6,337	3,246
V1	80	20,570	5,118	16,480	6,337	3,246
V1	80	21,683	5,254	16,785	6,340	3,420
V2	80	21,308	5,209	16,796	6,340	3,361
V1	80	20,050	5,053	16,307	6,340	3,162
V2	80	21,695	5,256	16,872	6,340	3,422
V2	80	21,006	5,172	16,663	6,340	3,313
V2	80	20,389	5,095	16,551	6,348	3,212
V2	80	22,772	5,385	17,116	6,352	3,585
V2	80	22,397	5,340	17,019	6,352	3,526

V2	80	21,357	5,215	16,663	6,352	3,362
V1	80	20,873	5,155	16,933	6,352	3,286
V1	80	20,606	5,122	16,612	6,352	3,244
V1	80	22,252	5,323	16,872	6,353	3,503
V2	80	21,732	5,260	16,811	6,356	3,419
V2	80	21,139	5,188	16,516	6,357	3,325
V2	80	21,090	5,182	16,541	6,357	3,318
V1	80	21,720	5,259	16,775	6,358	3,416
V2	80	21,695	5,256	16,749	6,358	3,412
V1	80	21,526	5,235	16,724	6,358	3,386
V2	80	20,715	5,136	16,480	6,358	3,258
V1	80	21,974	5,289	16,958	6,358	3,456
V1	80	21,090	5,182	16,638	6,358	3,317
V1	80	20,836	5,151	16,602	6,358	3,277
V2	80	22,300	5,329	17,131	6,363	3,505
V2	80	21,828	5,272	16,922	6,363	3,431
V2	80	21,756	5,263	16,811	6,363	3,419
V2	80	22,276	5,326	17,059	6,363	3,501
V2	80	22,276	5,326	17,059	6,363	3,501
V2	80	21,635	5,248	16,760	6,363	3,400
V2	80	21,272	5,204	16,577	6,363	3,343
V2	80	21,248	5,201	16,663	6,363	3,339
V1	80	20,703	5,134	16,592	6,363	3,253
V1	80	22,058	5,300	16,836	6,364	3,466
V2	80	22,046	5,298	16,836	6,364	3,464
V2	80	20,752	5,140	16,455	6,364	3,261
V1	80	22,458	5,347	17,116	6,367	3,527
V2	80	21,381	5,218	16,724	6,367	3,358
V1	80	19,687	5,007	16,465	6,367	3,092
V2	80	22,675	5,373	17,141	6,368	3,561
V2	80	21,090	5,182	16,785	6,368	3,312
V1	80	21,623	5,247	16,724	6,369	3,395
V2	80	22,663	5,372	16,922	6,369	3,558
V2	80	21,248	5,201	16,627	6,369	3,336
V2	80	21,490	5,231	16,775	6,372	3,372
V1	80	20,727	5,137	16,566	6,372	3,253
V2	80	21,816	5,270	16,811	6,373	3,423
V2	80	20,691	5,133	16,566	6,373	3,247
V2	80	21,974	5,289	17,070	6,373	3,448
V2	80	21,115	5,185	16,663	6,376	3,312
V1	80	22,700	5,376	17,106	6,377	3,560
V2	80	21,683	5,254	16,811	6,377	3,400
V1	80	22,409	5,342	16,983	6,378	3,514
V2	80	21,151	5,189	16,602	6,378	3,316
V1	80	22,724	5,379	17,655	6,381	3,561
V2	80	21,889	5,279	16,983	6,381	3,430
V2	80	21,841	5,273	17,019	6,381	3,423
V2	80	20,582	5,119	16,429	6,381	3,226
V2	80	22,724	5,379	17,264	6,381	3,561
V2	80	23,075	5,420	17,192	6,390	3,511
V1	80	21,042	5,176	16,627	6,390	3,293
V2	80	20,885	5,157	16,516	6,390	3,268

V1	80	21,514	5,234	16,836	6,391	3,366
V1	80	21,223	5,198	16,948	6,391	3,321
V2	80	21,320	5,210	16,688	6,391	3,336
V2	80	21,030	5,175	16,688	6,400	3,286
V2	80	22,046	5,298	17,095	6,400	3,445
V2	80	22,216	5,318	16,983	6,400	3,471
V2	80	21,296	5,207	16,638	6,403	3,326
V2	80	21,248	5,201	16,627	6,404	3,318
V2	80	21,889	5,279	16,907	6,406	3,417
V1	80	21,877	5,278	16,907	6,406	3,415
V1	80	23,656	5,488	17,436	6,406	3,693
V2	80	22,591	5,363	17,202	6,409	3,525
V2	80	21,441	5,225	16,699	6,409	3,345
V2	80	22,022	5,295	16,872	6,409	3,436
V1	80	22,046	5,298	16,969	6,410	3,440
V2	80	22,107	5,305	16,933	6,410	3,449
V1	80	21,659	5,251	16,785	6,410	3,379
V2	80	21,308	5,209	16,688	6,410	3,324
V2	80	21,078	5,181	16,663	6,410	3,288
V2	80	23,898	5,516	17,523	6,410	3,628
V1	80	22,409	5,342	17,278	6,410	3,496
V2	80	22,349	5,334	17,019	6,413	3,485
V2	80	22,276	5,326	16,933	6,413	3,474
V2	80	21,284	5,206	16,775	6,413	3,319
V1	80	20,812	5,148	16,516	6,413	3,245
V2	80	22,361	5,336	17,152	6,414	3,486
V2	80	22,361	5,336	17,152	6,414	3,486
V1	80	22,034	5,297	16,897	6,416	3,434
V1	80	21,078	5,181	16,663	6,416	3,285
Control	Control	19,203	4,945	15,742	5,940	3,233
Control	Control	19,312	4,959	15,829	6,058	3,188
Control	Control	21,453	5,226	16,541	6,125	3,503
Control	Control	19,372	4,966	15,778	5,965	3,248
Control	Control	21,006	5,172	16,541	6,274	3,348
Control	Control	19,687	5,007	15,890	5,814	3,386
Control	Control	18,271	4,823	15,519	5,880	3,107
Control	Control	20,715	5,136	16,307	6,071	3,412
Control	Control	18,682	4,877	15,570	6,073	3,076
Control	Control	19,638	5,000	16,012	6,094	3,223
Control	Control	19,191	4,943	16,098	5,717	3,357
Control	Control	19,287	4,956	15,803	5,918	3,259
Control	Control	18,053	4,794	15,178	5,582	3,234
Control	Control	17,787	4,759	15,285	5,579	3,188
Control	Control	20,086	5,057	16,221	6,157	3,262
Control	Control	19,058	4,926	15,717	6,059	3,146
Control	Control	20,594	5,121	16,490	6,260	3,290
Control	Control	19,009	4,920	15,839	5,935	3,203
Control	Control	19,650	5,002	15,987	6,183	3,178
Control	Control	19,481	4,980	15,829	5,956	3,271
Control	Control	20,631	5,125	16,368	6,111	3,376
Control	Control	20,994	5,170	16,455	6,223	3,374
Control	Control	21,925	5,284	16,969	5,965	3,676

Control	Control	20,098	5,059	16,134	5,977	3,363
Control	Control	19,953	5,040	16,134	6,061	3,292
Control	Control	23,668	5,489	17,558	6,430	3,681
Control	Control	21,828	5,272	16,760	6,509	3,353
Control	Control	20,352	5,091	16,368	6,151	3,309
Control	Control	21,381	5,218	16,577	6,200	3,449
Control	Control	20,231	5,075	16,185	6,004	3,370
Control	Control	18,852	4,899	15,620	5,911	3,189
Control	Control	19,759	5,016	16,073	6,210	3,182
Control	Control	16,722	4,614	14,857	5,590	2,991
Control	Control	22,022	5,295	16,943	6,265	3,515
Control	Control	19,336	4,962	15,595	5,748	3,364
Control	Control	18,924	4,909	15,473	5,619	3,368
Control	Control	19,929	5,037	16,098	6,134	3,249
Control	Control	20,159	5,066	16,037	5,850	3,446
Control	Control	20,376	5,094	16,170	6,030	3,379
Control	Control	19,251	4,951	15,753	5,793	3,323
Control	Control	19,844	5,027	15,951	6,018	3,298
Control	Control	19,033	4,923	15,605	5,726	3,324
Control	Control	18,634	4,871	15,559	5,697	3,271
Control	Control	20,739	5,139	16,318	6,034	3,437
Control	Control	21,853	5,275	17,080	6,643	3,289
Control	Control	19,542	4,988	16,037	6,217	3,143
Control	Control	20,824	5,149	16,455	6,120	3,403
Control	Control	20,038	5,051	16,073	5,845	3,428
Control	Control	20,159	5,066	16,195	6,168	3,268
Control	Control	18,561	4,861	15,717	5,826	3,186
Control	Control	23,159	5,430	17,497	6,604	3,507
Control	Control	19,469	4,979	15,595	5,665	3,436
Control	Control	20,122	5,062	16,221	6,147	3,273
Control	Control	20,510	5,110	16,332	6,156	3,331
Control	Control	20,534	5,113	16,195	5,977	3,435
Control	Control	24,684	5,606	17,879	6,406	3,853
Control	Control	21,296	5,207	16,724	6,337	3,361
Control	Control	22,942	5,405	17,584	7,003	3,276
Control	Control	22,264	5,324	16,922	6,246	3,564
Control	Control	17,013	4,654	14,735	5,502	3,092
Control	Control	20,304	5,084	16,256	6,034	3,365
Control	Control	20,074	5,056	16,221	6,309	3,182
Control	Control	20,413	5,098	16,246	6,031	3,385
Control	Control	21,187	5,194	16,602	6,278	3,375
Control	Control	21,042	5,176	16,551	6,170	3,410
Control	Control	21,187	5,194	16,516	6,237	3,397
Control	Control	20,594	5,121	16,429	6,189	3,328
Control	Control	23,184	5,433	17,558	6,585	3,521
Control	Control	20,147	5,065	15,742	5,731	3,515
Control	Control	20,534	5,113	16,307	6,222	3,300
Control	Control	20,788	5,145	16,393	6,246	3,328
Control	Control	20,885	5,157	16,282	6,059	3,447
Control	Control	23,305	5,447	17,192	6,265	3,720
Control	Control	20,449	5,103	16,393	6,247	3,274
Control	Control	22,675	5,373	17,523	6,982	3,248

Control	Control	23,087	5,422	17,314	6,376	3,621
Control	Control	20,110	5,060	16,246	6,164	3,262
Control	Control	21,429	5,223	16,724	6,157	3,481
Control	Control	20,195	5,071	16,282	6,275	3,219
Control	Control	19,239	4,949	15,681	5,899	3,261
Control	Control	21,841	5,273	16,882	6,275	3,481
Control	Control	18,598	4,866	15,386	5,772	3,222
Control	Control	21,296	5,207	16,724	6,263	3,400
Control	Control	20,715	5,136	16,490	6,238	3,321
Control	Control	21,211	5,197	16,638	6,140	3,455
Control	Control	20,461	5,104	16,404	6,303	3,246
Control	Control	19,941	5,039	16,037	5,940	3,357
Control	Control	19,747	5,014	15,951	6,072	3,252
Control	Control	19,469	4,979	15,926	6,096	3,194
Control	Control	20,122	5,062	16,221	6,171	3,261
Control	Control	21,211	5,197	16,663	6,463	3,282
Control	Control	20,304	5,084	16,098	6,058	3,351
Control	Control	19,239	4,949	15,656	5,870	3,277
Control	Control	21,127	5,186	16,526	6,168	3,425
Control	Control	18,077	4,798	15,264	5,619	3,217
Control	Control	20,788	5,145	16,368	6,168	3,370
Control	Control	21,344	5,213	16,699	6,238	3,422
Control	Control	21,308	5,209	16,760	6,111	3,487
Control	Control	20,727	5,137	16,551	6,369	3,255
Control	Control	19,445	4,976	15,829	5,904	3,293
Control	Control	19,529	4,987	15,926	6,134	3,184
Control	Control	20,086	5,057	16,221	6,237	3,221
Control	Control	21,344	5,213	16,699	6,463	3,303
Control	Control	20,824	5,149	16,332	6,162	3,380
Control	Control	19,312	4,959	15,717	5,899	3,274
Control	Control	21,453	5,226	16,760	6,324	3,392
Control	Control	17,727	4,751	15,030	5,626	3,151
Control	Control	20,957	5,166	16,429	6,128	3,420
Control	Control	21,744	5,262	16,846	6,238	3,486
Control	Control	21,320	5,210	16,785	6,062	3,517
Control	Control	20,800	5,146	16,577	6,510	3,195
Control	Control	19,699	5,008	16,037	5,940	3,316
V1	50	17,666	4,743	15,178	5,724	3,086
V1	50	16,069	4,523	14,573	5,335	3,012
V1	50	17,642	4,739	15,289	5,726	3,081
V1	50	15,851	4,492	14,771	5,609	2,826
V1	50	18,803	4,893	15,483	5,609	3,353
V1	50	17,376	4,704	15,412	5,911	2,939
V1	50	16,456	4,577	15,127	5,777	2,849
V1	50	15,766	4,480	14,782	5,526	2,853
V1	50	15,766	4,480	14,659	5,523	2,855
V1	50	15,960	4,508	14,756	5,429	2,940
V1	50	18,186	4,812	15,386	5,645	3,222
V1	50	16,940	4,644	14,944	5,552	3,051
V1	50	17,146	4,672	15,081	5,581	3,072
V1	50	17,170	4,676	15,030	5,676	3,025
V1	50	16,456	4,577	14,919	5,697	2,888

V1	50	18,174	4,810	15,325	5,636	3,225
V1	50	16,311	4,557	14,771	5,786	2,819
V1	50	18,065	4,796	15,264	5,544	3,259
V1	50	16,819	4,628	14,883	5,573	3,018
V1	65	21,320	5,210	16,541	6,211	3,433
V1	65	20,800	5,146	16,465	6,063	3,431
V1	65	18,586	4,865	15,473	5,661	3,283
V1	65	19,033	4,923	15,717	5,977	3,184
V1	65	20,328	5,087	16,256	5,977	3,401
V1	65	19,554	4,990	15,951	5,937	3,294
V1	65	18,247	4,820	15,422	5,821	3,135
V1	65	19,433	4,974	15,936	5,889	3,300
V1	65	18,755	4,887	15,681	5,977	3,138
V1	65	18,924	4,909	15,620	5,911	3,201
V1	65	19,070	4,927	15,707	5,922	3,220
V1	65	17,557	4,728	15,091	5,523	3,179
V1	65	19,590	4,994	15,814	5,927	3,305
V1	65	18,743	4,885	15,620	5,882	3,187
V1	65	18,743	4,885	15,742	6,032	3,107
V1	65	19,941	5,039	16,002	5,955	3,348
V1	65	20,340	5,089	16,307	6,211	3,275
V1	65	20,437	5,101	16,674	6,403	3,192
V1	65	20,038	5,051	16,195	6,096	3,287
V1	65	18,380	4,838	15,559	5,786	3,176
V1	65	16,323	4,559	14,598	5,327	3,064
V1	65	18,477	4,850	15,987	5,934	3,114
V1	65	16,226	4,545	14,735	5,552	2,923
V1	65	18,816	4,895	16,084	6,059	3,105
V1	65	18,174	4,810	15,422	5,731	3,171
V1	65	17,460	4,715	15,030	5,519	3,163
V1	65	16,795	4,624	14,868	5,607	2,995
V1	65	21,296	5,207	17,152	6,623	3,216
V1	65	17,521	4,723	15,361	5,807	3,017
V1	65	18,525	4,857	15,523	5,581	3,319
V1	65	17,860	4,769	15,361	5,918	3,018
V1	65	18,332	4,831	15,656	5,940	3,086
V1	65	18,332	4,831	15,631	5,927	3,093
V1	65	19,179	4,942	15,900	5,793	3,311
V1	65	18,295	4,826	15,336	5,749	3,182
V1	65	18,162	4,809	15,361	5,812	3,125
V1	65	19,784	5,019	16,465	6,278	3,151
V1	65	18,247	4,820	15,275	5,725	3,187
V1	65	18,150	4,807	15,458	5,658	3,208
V1	65	18,198	4,814	15,300	5,751	3,164
V1	65	20,110	5,060	16,246	6,094	3,300
V1	65	18,150	4,807	15,509	5,793	3,133
V1	65	19,578	4,993	16,037	5,937	3,298
V1	65	20,316	5,086	16,332	6,218	3,267
V1	65	20,558	5,116	16,429	6,237	3,296
V1	65	20,534	5,113	16,551	6,278	3,271
V1	65	18,561	4,861	15,681	5,977	3,105
V1	65	20,582	5,119	16,307	6,162	3,340

V1	65	19,578	4,993	16,073	6,167	3,174
V1	65	19,808	5,022	15,987	5,993	3,305
V1	65	18,428	4,844	15,447	5,849	3,150
V1	65	16,275	4,552	14,796	5,619	2,896
V1	65	19,953	5,040	16,465	6,278	3,178
V1	65	19,626	4,999	16,195	6,132	3,201
V1	65	18,416	4,842	15,473	5,581	3,300
V1	65	17,460	4,715	15,056	5,677	3,076
V1	65	18,561	4,861	15,631	5,871	3,161
V1	65	18,440	4,846	15,386	5,826	3,165
V1	65	18,453	4,847	15,458	5,889	3,133
V1	65	19,650	5,002	16,063	6,096	3,223
V1	65	17,884	4,772	15,214	5,572	3,209
V1	65	18,864	4,901	15,707	6,091	3,097
V1	65	19,239	4,949	15,778	5,826	3,302
V1	65	18,682	4,877	15,656	5,814	3,213
V1	65	19,469	4,979	15,900	5,880	3,311
V1	65	17,400	4,707	14,969	5,508	3,159
V1	65	20,909	5,160	16,490	6,265	3,337
V1	65	18,803	4,893	15,915	6,030	3,118
V1	65	19,433	4,974	15,926	5,977	3,251
V1	65	19,590	4,994	15,976	6,087	3,219
V1	65	20,243	5,077	16,404	5,977	3,387
V1	65	20,243	5,077	16,307	6,167	3,282
V1	80	20,437	5,101	16,551	6,417	3,185
V2	80	21,974	5,289	17,019	6,417	3,424
V2	80	21,453	5,226	16,811	6,427	3,338
V2	80	22,191	5,316	17,141	6,427	3,453
V2	80	21,586	5,243	16,872	6,427	3,358
V2	80	23,002	5,412	17,253	6,428	3,579
V2	80	21,042	5,176	16,541	6,428	3,274
V1	80	23,232	5,439	17,339	6,428	3,514
V1	80	22,494	5,352	17,070	6,428	3,499
V1	80	22,397	5,340	17,044	6,428	3,484
V1	80	20,219	5,074	16,480	6,428	3,145
V2	80	22,095	5,304	17,019	6,430	3,436
V1	80	19,905	5,034	16,465	6,430	3,096
V2	80	22,107	5,305	17,080	6,431	3,438
V2	80	21,284	5,206	16,699	6,438	3,306
V1	80	22,506	5,353	17,213	6,444	3,493
V2	80	22,095	5,304	16,958	6,444	3,429
V1	80	20,715	5,136	16,663	6,444	3,215
V1	80	20,715	5,136	16,663	6,444	3,215
V2	80	23,801	5,505	17,660	6,444	3,693
V1	80	22,482	5,350	16,922	6,453	3,484
V1	80	21,248	5,201	16,688	6,454	3,292
V2	80	20,994	5,170	16,688	6,454	3,253
V2	80	21,937	5,285	16,811	6,454	3,399
V1	80	21,562	5,240	16,811	6,454	3,341
V2	80	22,373	5,337	17,044	6,454	3,467
V1	80	21,090	5,182	16,688	6,454	3,268
V1	80	20,086	5,057	16,541	6,454	3,112

V1	80	23,837	5,509	17,472	6,456	3,692
V2	80	20,800	5,146	16,760	6,458	3,221
V1	80	20,171	5,068	16,368	6,458	3,123
V2	80	22,603	5,365	17,106	6,463	3,497
V2	80	20,812	5,148	16,577	6,463	3,220
V2	80	21,163	5,191	16,836	6,463	3,274
V2	80	21,828	5,272	16,836	6,463	3,377
V1	80	21,381	5,218	16,724	6,463	3,308
V2	80	22,264	5,324	17,044	6,468	3,442
V2	80	23,147	5,429	17,339	6,469	3,578
V2	80	21,574	5,241	17,019	6,469	3,335
V1	80	21,018	5,173	16,663	6,469	3,249
V1	80	21,623	5,247	16,872	6,481	3,336
V2	80	21,006	5,172	16,688	6,481	3,241
V1	80	22,869	5,396	17,289	6,481	3,528
V2	80	22,470	5,349	17,350	6,481	3,467
V2	80	24,152	5,545	17,767	6,482	3,626
V1	80	22,954	5,406	17,278	6,483	3,541
V1	80	22,022	5,295	16,983	6,483	3,397
V2	80	21,127	5,186	16,724	6,483	3,259
V2	80	21,683	5,254	17,055	6,487	3,342
V2	80	22,627	5,367	17,192	6,490	3,487
V2	80	22,482	5,350	17,192	6,490	3,464
V2	80	21,405	5,220	16,836	6,490	3,298
V2	80	24,490	5,584	18,001	6,495	3,671
V1	80	21,841	5,273	16,897	6,503	3,359
V2	80	22,639	5,369	17,573	6,503	3,481
V2	80	23,692	5,492	17,742	6,509	3,640
V1	80	21,502	5,232	16,811	6,509	3,303
V2	80	20,715	5,136	16,688	6,509	3,183
V2	80	23,196	5,434	17,375	6,509	3,563
V2	80	21,066	5,179	16,749	6,509	3,236
V2	50	18,053	4,794	15,509	5,852	3,085
V2	50	16,807	4,626	15,249	5,880	2,858
V2	50	17,920	4,777	15,544	5,865	3,055
V2	50	15,343	4,420	14,120	5,396	2,843
V2	50	17,896	4,773	15,631	6,208	2,883
V2	50	16,250	4,549	14,822	5,634	2,884
V2	50	21,066	5,179	16,688	6,162	3,419
V2	50	19,614	4,997	16,037	6,208	3,160
V2	50	17,243	4,685	15,214	5,826	2,960
V2	50	19,203	4,945	15,793	5,922	3,243
V2	50	18,090	4,799	15,325	5,826	3,105
V2	50	18,029	4,791	15,422	5,645	3,194
V2	50	17,388	4,705	15,020	5,637	3,085
V2	50	20,643	5,127	16,562	6,275	3,290
V2	50	17,956	4,782	15,559	6,127	2,931
V2	50	17,533	4,725	15,310	5,927	2,958
V2	50	19,130	4,935	16,002	6,096	3,138
V2	50	18,126	4,804	15,595	5,912	3,066
V2	50	19,759	5,016	15,926	5,852	3,377
V2	50	18,828	4,896	15,829	6,121	3,076

V2	50	18,876	4,902	15,656	5,889	3,205
V2	50	19,711	5,010	16,393	5,928	3,325
V2	50	18,090	4,799	15,681	6,167	2,933
V2	50	20,352	5,091	16,465	6,218	3,273
V2	50	17,460	4,715	15,498	5,819	3,001
V2	50	18,937	4,910	15,702	5,786	3,273
V2	50	19,445	4,976	15,915	5,904	3,294
V2	50	22,736	5,380	17,325	6,536	3,478
V2	50	21,441	5,225	16,785	6,275	3,417
V2	50	19,045	4,924	15,890	6,030	3,158
V2	50	19,191	4,943	16,048	6,200	3,096
V2	50	19,542	4,988	15,926	6,111	3,198
V2	50	19,118	4,934	15,926	6,071	3,149
V2	50	22,579	5,362	17,080	6,413	3,521
V2	50	20,473	5,106	16,501	6,096	3,359
V2	50	19,360	4,965	15,926	5,899	3,282
V2	50	21,199	5,195	16,714	6,318	3,355
V2	50	19,191	4,943	15,839	6,164	3,113
V2	50	20,062	5,054	16,358	6,211	3,230
V2	50	20,631	5,125	16,404	6,157	3,351
V2	50	19,445	4,976	16,109	6,034	3,223
V2	50	19,711	5,010	15,940	5,841	3,375
V2	50	16,783	4,623	14,980	5,658	2,966
V2	50	19,542	4,988	16,037	5,749	3,399
V2	50	16,698	4,611	15,224	5,899	2,831
V2	50	19,021	4,921	15,605	5,819	3,269
V2	50	18,949	4,912	15,692	5,880	3,223
V2	50	20,038	5,051	16,012	5,993	3,343
V2	50	19,675	5,005	15,900	5,852	3,362
V2	50	17,908	4,775	15,249	5,743	3,118
V2	50	18,150	4,807	15,681	5,918	3,067
V2	50	21,320	5,210	16,516	6,164	3,459
V2	50	20,691	5,133	16,343	6,182	3,347
V2	50	20,231	5,075	16,124	6,112	3,310
V2	50	25,325	5,678	18,591	7,166	3,534
V2	50	19,638	5,000	16,037	5,845	3,360
V2	50	22,312	5,330	17,451	6,517	3,424
V2	50	21,320	5,210	16,846	6,476	3,292
V2	50	21,308	5,209	17,141	6,706	3,178
V2	50	21,102	5,183	16,760	6,430	3,282
V2	50	22,191	5,316	17,253	6,564	3,381
V2	50	18,912	4,907	15,961	6,148	3,076
V2	50	20,969	5,167	16,785	6,428	3,262
V2	50	18,561	4,861	15,753	6,162	3,012
V2	50	19,529	4,987	16,084	6,091	3,206
V2	50	18,247	4,820	15,544	5,845	3,122
V2	50	22,567	5,360	17,289	6,457	3,495
V2	50	20,945	5,164	16,566	6,157	3,402
V2	50	21,115	5,185	16,724	6,297	3,353
V2	50	19,784	5,019	16,480	6,391	3,096
V2	50	19,215	4,946	15,915	6,112	3,144
V2	50	19,070	4,927	15,940	5,977	3,191

V2	50	18,380	4,838	15,498	6,111	3,008
V2	50	19,505	4,983	15,976	5,940	3,283
V2	50	16,057	4,522	14,405	5,426	2,959
V2	50	18,646	4,872	15,631	5,814	3,207
V2	50	19,542	4,988	16,109	6,062	3,224
V2	50	21,768	5,265	16,969	6,297	3,457
V2	50	18,695	4,879	15,631	5,870	3,185
V2	50	21,417	5,222	17,080	6,564	3,263
V2	50	19,142	4,937	15,951	6,006	3,187
V2	50	20,655	5,128	16,648	6,381	3,237
V2	50	18,997	4,918	15,814	5,955	3,190
V2	50	20,183	5,069	16,368	6,223	3,243
V2	50	19,324	4,960	15,839	5,917	3,266
V2	65	19,130	4,935	15,728	5,852	3,269
V2	65	21,478	5,229	16,516	6,157	3,488
V2	65	18,380	4,838	15,188	5,479	3,354
V2	65	20,752	5,140	16,368	6,167	3,365
V2	65	20,497	5,109	16,368	6,162	3,326
V2	65	19,348	4,963	15,717	5,751	3,364
V2	65	19,626	4,999	15,961	5,793	3,388
V2	65	20,655	5,128	16,684	6,164	3,351
V2	65	22,167	5,313	16,811	6,318	3,508
V2	65	18,695	4,879	15,559	5,697	3,281
V2	65	19,445	4,976	15,915	5,850	3,324
V2	65	19,602	4,996	15,915	5,787	3,387
V2	65	18,537	4,858	15,264	5,526	3,354
V2	65	21,199	5,195	16,444	5,927	3,577
V2	65	19,687	5,007	15,915	5,877	3,350
V2	65	23,172	5,432	17,508	6,509	3,560
V2	65	19,529	4,987	15,829	5,841	3,343
V2	65	19,421	4,973	16,210	6,247	3,109
V2	65	19,130	4,935	15,707	5,731	3,338
V2	65	20,038	5,051	15,987	5,984	3,348
V2	65	18,755	4,887	15,570	5,825	3,220
V2	65	20,522	5,112	16,688	6,378	3,218
V2	65	19,033	4,923	15,803	5,956	3,196
V2	65	20,038	5,051	16,048	5,956	3,364
V2	65	20,183	5,069	16,393	6,301	3,203
V2	65	21,018	5,173	16,627	6,340	3,315
V2	65	20,026	5,049	16,048	5,956	3,362
V2	65	18,211	4,815	15,239	5,580	3,264
V2	65	19,529	4,987	16,109	5,918	3,300
V2	65	20,364	5,092	16,160	6,134	3,320
V2	65	19,336	4,962	15,865	5,899	3,278
V2	65	18,828	4,896	15,605	5,744	3,278
V2	65	18,791	4,891	15,961	6,011	3,126
V2	65	20,207	5,072	16,073	5,956	3,393
V2	65	21,514	5,234	16,638	6,059	3,551
V2	65	18,695	4,879	15,666	5,890	3,174
V2	65	20,727	5,137	16,332	5,977	3,468
V2	65	17,751	4,754	15,163	5,645	3,145
V2	65	18,767	4,888	15,976	6,072	3,091

V2	65	19,421	4,973	15,900	5,977	3,249
V2	65	17,714	4,749	15,056	5,454	3,248
V2	65	19,844	5,027	16,231	5,985	3,316
V2	65	19,687	5,007	15,865	5,825	3,380
V2	65	19,142	4,937	15,742	5,963	3,210
V2	65	17,884	4,772	15,310	5,609	3,188
V2	65	21,054	5,178	16,490	6,034	3,489
V2	65	20,195	5,071	16,221	5,972	3,382
V2	65	20,026	5,049	16,124	5,977	3,350
V2	65	21,211	5,197	16,577	6,111	3,471
V2	65	20,171	5,068	16,271	5,982	3,372
V2	65	19,300	4,957	15,803	6,171	3,127
V2	65	20,485	5,107	16,271	6,113	3,351
V2	65	19,058	4,926	15,570	5,743	3,318
V2	65	22,591	5,363	17,167	6,349	3,558
V2	65	20,546	5,115	16,271	5,940	3,459
V2	65	19,917	5,036	16,048	6,030	3,303
V2	65	20,679	5,131	16,419	6,094	3,394
V2	65	19,856	5,028	15,976	5,814	3,415
V2	65	20,304	5,084	16,098	5,881	3,452
V2	65	20,715	5,136	16,541	6,337	3,269
V2	65	19,263	4,952	15,656	5,711	3,373
V2	65	20,788	5,145	16,246	5,972	3,481
V2	65	20,764	5,142	16,602	6,132	3,386
V2	65	19,905	5,034	15,915	5,826	3,416
V2	65	21,127	5,186	16,577	6,124	3,450
V2	65	19,179	4,942	15,717	5,880	3,262
V2	65	20,764	5,142	16,307	5,993	3,465
V2	65	19,445	4,976	15,890	6,168	3,153
V2	65	19,336	4,962	15,717	5,793	3,338
V2	65	19,808	5,022	15,951	5,911	3,351
V2	65	18,453	4,847	15,544	5,880	3,138
V2	65	20,401	5,097	16,404	6,062	3,366
V2	65	20,800	5,146	16,332	6,066	3,429
V2	65	19,880	5,031	16,023	5,825	3,413
V2	65	20,098	5,059	16,048	6,171	3,257
V2	65	19,868	5,030	16,063	5,955	3,336
V2	65	19,457	4,977	15,915	5,882	3,308
V2	65	18,332	4,831	15,325	5,634	3,254
V2	65	19,179	4,942	15,839	5,889	3,257
V2	65	19,687	5,007	15,900	5,814	3,386
V2	65	21,465	5,228	16,602	5,867	3,659
V2	65	18,598	4,866	15,509	5,726	3,248
V2	65	19,711	5,010	15,890	5,871	3,357
V2	65	19,481	4,980	15,890	5,882	3,312
V2	65	20,449	5,103	16,429	6,246	3,274
V2	65	17,581	4,731	15,117	5,766	3,049
V2	65	19,759	5,016	16,023	5,899	3,350
V2	65	19,384	4,968	15,707	5,871	3,301
V2	65	19,336	4,962	15,717	5,912	3,271
V2	65	20,207	5,072	16,271	6,067	3,331
V2	65	19,771	5,017	16,063	6,018	3,285

V2	65	20,691	5,133	16,566	6,182	3,347
V2	65	19,796	5,020	16,027	5,865	3,375
V2	65	19,723	5,011	15,915	6,111	3,228
V2	65	20,425	5,100	16,195	5,899	3,463
V2	65	18,731	4,884	15,534	5,827	3,215
V2	65	20,159	5,066	16,124	6,021	3,348
V2	65	18,852	4,899	15,534	5,827	3,235
V2	65	21,211	5,197	16,541	6,112	3,470
V2	65	19,941	5,039	16,037	6,030	3,307
V2	65	18,707	4,880	15,605	5,607	3,336
V2	65	19,929	5,037	16,037	5,977	3,334
V2	65	20,497	5,109	16,332	6,264	3,272
V2	65	19,457	4,977	15,976	5,850	3,326
V2	65	19,360	4,965	15,768	5,927	3,266
V2	65	18,973	4,915	15,768	5,850	3,243
V2	65	18,961	4,913	15,544	5,819	3,259
V2	65	19,905	5,034	15,976	5,971	3,333
V2	65	20,643	5,127	16,307	6,183	3,339
V2	65	18,997	4,918	15,620	5,658	3,358
V2	65	21,611	5,246	16,907	6,407	3,373
V2	65	21,465	5,228	16,872	6,246	3,436
V2	65	18,791	4,891	15,717	5,845	3,215
V2	65	17,956	4,782	15,188	5,609	3,202
V2	65	20,062	5,054	16,429	6,189	3,241
V2	65	20,231	5,075	16,098	5,922	3,416
V2	65	20,147	5,065	16,195	6,091	3,308
V2	65	21,236	5,200	16,566	6,058	3,506
V2	65	19,203	4,945	16,098	5,977	3,213
V2	65	20,449	5,103	16,160	5,955	3,434
V2	65	21,381	5,218	16,612	6,005	3,560
V2	65	18,731	4,884	15,509	5,814	3,222
V2	65	21,090	5,182	16,541	5,995	3,518
V2	65	20,062	5,054	16,160	6,030	3,327
V2	65	19,989	5,045	15,890	5,764	3,468
V2	65	18,803	4,893	15,620	5,827	3,227
V2	65	20,631	5,125	16,271	5,865	3,518
V2	65	19,759	5,016	15,926	5,918	3,339
V2	65	19,856	5,028	15,803	5,786	3,432
V2	65	20,171	5,068	16,235	5,967	3,380
V2	65	19,275	4,954	15,814	5,821	3,311
V2	65	20,304	5,084	16,048	5,827	3,485
V2	65	22,470	5,349	16,969	6,169	3,642
V2	65	18,501	4,853	15,473	5,918	3,126
V2	65	20,195	5,071	16,109	5,927	3,407
V2	80	21,066	5,179	16,749	6,509	3,236
V2	80	21,502	5,232	16,872	6,516	3,300
V1	80	21,332	5,212	17,004	6,516	3,274
V2	80	21,526	5,235	16,836	6,517	3,303
V1	80	21,236	5,200	16,760	6,517	3,258
V1	80	22,675	5,373	17,365	6,517	3,479
V1	80	22,675	5,373	17,365	6,517	3,479
V1	80	22,276	5,326	17,095	6,517	3,418

V2	80	23,172	5,432	17,314	6,519	3,555
V1	80	23,704	5,494	17,523	6,519	3,536
V1	80	23,486	5,468	17,462	6,519	3,502
V1	80	23,777	5,502	17,573	6,523	3,645
V1	80	21,877	5,278	16,897	6,523	3,354
V1	80	21,562	5,240	16,846	6,523	3,306
V1	80	21,151	5,189	16,836	6,523	3,243
V1	80	22,046	5,298	17,070	6,523	3,380
V2	80	21,768	5,265	16,933	6,523	3,337
V2	80	20,425	5,100	16,836	6,523	3,131
V1	80	22,337	5,333	17,217	6,532	3,420
V2	80	27,503	5,918	19,033	6,536	3,770
V1	80	24,636	5,601	17,757	6,544	3,665
V2	80	22,760	5,383	17,350	6,544	3,478
V2	80	22,760	5,383	17,350	6,544	3,478
V2	80	21,865	5,276	17,091	6,545	3,341
V1	80	23,087	5,422	17,192	6,547	3,526
V2	80	21,828	5,272	16,933	6,547	3,334
V2	80	22,784	5,386	17,253	6,556	3,475
V1	80	23,837	5,509	17,523	6,563	3,532
V1	80	22,397	5,340	17,167	6,563	3,413
V2	80	22,579	5,362	17,177	6,563	3,440
V1	80	22,494	5,352	17,228	6,563	3,427
V1	80	22,518	5,355	17,202	6,565	3,430
V2	80	22,119	5,307	17,264	6,575	3,364
V1	80	21,393	5,219	16,907	6,575	3,254
V2	80	22,748	5,382	17,339	6,580	3,457
V1	80	22,228	5,320	17,167	6,580	3,378
V2	80	23,014	5,413	17,411	6,585	3,495
V2	80	23,135	5,427	17,365	6,602	3,504
V2	80	22,942	5,405	17,192	6,602	3,475
V1	80	21,974	5,289	17,177	6,603	3,328
V2	80	22,361	5,336	17,141	6,604	3,386
V2	80	22,070	5,301	17,167	6,604	3,342
V1	80	20,352	5,091	16,490	6,607	3,081
V2	80	20,957	5,166	16,785	6,609	3,171
V1	80	23,087	5,422	17,375	6,613	3,491
V1	80	21,901	5,281	16,969	6,613	3,312
V1	80	22,724	5,379	17,685	6,613	3,436
V1	80	22,337	5,333	17,192	6,618	3,375
V1	80	22,337	5,333	17,192	6,618	3,375
V2	80	22,131	5,308	17,141	6,622	3,342
V2	80	22,155	5,311	17,141	6,622	3,346
V2	80	21,853	5,275	17,325	6,622	3,300
V1	80	23,643	5,487	18,001	6,634	3,564
V1	80	23,075	5,420	17,401	6,634	3,478
V2	80	21,296	5,207	17,202	6,643	3,206
V2	80	22,796	5,388	17,573	6,643	3,432
V2	80	21,308	5,209	17,044	6,643	3,208
V2	80	23,825	5,508	17,523	6,649	3,583
V2	80	23,075	5,420	17,411	6,650	3,470
V1	80	23,220	5,437	17,487	6,659	3,487

V1	80	22,204	5,317	17,177	6,659	3,334
V1	80	21,586	5,243	16,872	6,659	3,242
V1	80	21,986	5,291	17,447	6,663	3,300
V2	80	29,185	6,096	19,389	6,673	3,873
V2	80	23,474	5,467	17,548	6,674	3,517
V2	80	22,131	5,308	17,253	6,688	3,309
V2	80	24,055	5,534	17,818	6,697	3,592
V2	80	25,422	5,689	18,433	6,700	3,694
V1	80	22,409	5,342	17,365	6,706	3,342
V1	80	23,462	5,466	17,609	6,709	3,497
V1	80	23,716	5,495	17,634	6,714	3,533
V2	80	21,478	5,229	17,181	6,714	3,199
V2	80	23,668	5,489	17,548	6,714	3,525
V1	80	23,377	5,456	17,767	6,733	3,472
V1	80	23,293	5,446	17,573	6,733	3,459
V1	80	21,986	5,291	17,202	6,754	3,255
V1	80	23,329	5,450	17,462	6,754	3,454
V1	80	23,777	5,502	17,620	6,754	3,520
V2	80	23,607	5,482	17,558	6,754	3,495
V2	80	22,893	5,399	17,401	6,769	3,382
V1	80	22,204	5,317	17,573	6,788	3,271
V2	80	23,462	5,466	17,782	6,796	3,452
V1	80	25,809	5,732	18,245	6,802	3,695
V2	80	23,450	5,464	17,645	6,809	3,444
V1	80	22,772	5,385	17,523	6,809	3,345
V2	80	22,700	5,376	17,390	6,849	3,315
V1	80	22,591	5,363	17,742	6,913	3,268
V1	80	23,208	5,436	17,609	6,919	3,354
V2	80	25,216	5,666	18,209	6,941	3,533
V2	80	26,475	5,806	18,713	7,166	3,695
V1	80	27,128	5,877	19,339	7,600	3,570

MaxFeret	MinFeret	Circularity	Elongation	Macho	Object
6,602	4,311	0,911	1,532	M1	3
5,881	4,233	0,973	1,389	M1	7
6,941	5,187	0,953	1,338	M1	9
6,218	4,510	0,949	1,379	M1	10
5,870	4,070	0,959	1,442	M1	6
6,923	5,048	0,947	1,372	M1	12
6,510	4,284	0,929	1,520	M1	5
6,208	4,232	0,957	1,467	M1	8
5,786	4,622	0,973	1,252	M1	10
6,453	4,302	0,940	1,500	M1	9
5,995	4,388	0,964	1,366	M1	12
6,137	4,232	0,957	1,450	M1	14
5,867	4,077	0,960	1,439	M1	7
6,017	4,310	0,968	1,396	M1	13
6,164	4,084	0,942	1,509	M1	3
6,032	4,682	0,953	1,288	M1	7
6,699	4,070	0,919	1,646	M1	9
6,428	4,155	0,931	1,547	M1	14
5,645	3,960	0,962	1,426	M1	3
6,018	4,298	0,970	1,400	M1	5
6,128	4,310	0,958	1,422	M1	14
6,034	4,180	0,964	1,444	M1	17
5,871	4,123	0,965	1,424	M1	3
5,870	3,960	0,950	1,482	M1	15
6,199	4,180	0,963	1,483	M1	3
6,004	4,310	0,964	1,393	M1	7
6,403	4,552	0,957	1,407	M1	16
6,018	4,390	0,968	1,371	M1	9
6,618	4,311	0,925	1,535	M1	11
6,490	4,466	0,945	1,453	M1	5
5,526	3,745	0,960	1,476	M1	9
5,971	4,310	0,978	1,385	M1	14
5,993	4,290	0,959	1,397	M1	2
6,413	4,311	0,938	1,488	M1	3
6,378	4,589	0,966	1,390	M1	8
6,111	4,290	0,955	1,425	M1	10
6,210	4,311	0,946	1,441	M1	12
5,748	4,466	0,991	1,287	M1	14
6,356	4,470	0,952	1,422	M1	4
6,378	4,508	0,971	1,415	M1	5
6,262	4,388	0,953	1,427	M1	13
5,818	4,155	0,971	1,400	M1	16
6,034	4,510	0,975	1,338	M1	19
6,760	4,388	0,915	1,540	M1	10
6,120	4,388	0,967	1,395	M1	13
5,845	4,290	0,973	1,362	M1	3
5,911	4,311	0,962	1,371	M1	6
5,982	4,388	0,967	1,363	M1	9
6,124	4,180	0,968	1,465	M1	10
6,332	4,400	0,965	1,439	M1	12
5,636	4,233	0,978	1,332	M1	16

7,014	5,166	0,971	1,358	M1	11
6,275	4,510	0,986	1,391	M1	12
5,967	4,155	0,958	1,436	M1	8
6,134	4,283	0,976	1,432	M1	3
5,993	4,400	0,991	1,362	M1	6
6,030	4,400	0,978	1,371	M1	10
6,183	4,466	0,961	1,384	M1	12
7,075	5,260	0,963	1,345	M1	8
6,063	4,400	0,971	1,378	M1	13
6,404	4,563	0,955	1,403	M1	3
6,140	4,510	0,965	1,361	M1	4
6,162	4,510	0,974	1,366	M1	6
6,264	4,567	0,965	1,371	M1	11
6,304	4,438	0,959	1,420	M1	14
5,807	4,807	0,903	1,208	M1	5
6,227	4,510	0,969	1,381	M1	7
6,120	4,466	0,963	1,370	M1	8
6,483	4,544	0,950	1,427	M1	10
6,217	4,356	0,952	1,427	M1	11
6,728	4,534	0,950	1,484	M1	19
6,428	4,544	0,972	1,415	M1	22
6,227	4,510	0,969	1,381	M1	11
6,120	4,466	0,963	1,370	M1	12
6,483	4,544	0,950	1,427	M1	14
6,217	4,356	0,952	1,427	M1	15
6,728	4,534	0,950	1,484	M1	23
6,428	4,544	0,972	1,415	M1	26
6,164	4,403	0,973	1,400	M1	6
5,994	4,311	0,969	1,391	M1	7
5,927	4,284	0,977	1,384	M1	6
6,061	4,180	0,953	1,450	M1	5
6,096	4,510	0,967	1,352	M1	8
6,410	4,503	0,970	1,423	M1	6
6,353	4,400	0,966	1,444	M1	7
6,064	4,620	1,000	1,313	M1	8
6,247	4,400	0,967	1,420	M1	6
6,034	4,290	0,962	1,407	M1	7
6,428	4,367	0,927	1,472	M1	11
6,278	4,639	0,984	1,353	M1	12
6,032	4,544	0,973	1,327	M1	16
6,134	4,508	0,975	1,361	M1	17
6,510	4,649	0,961	1,400	M1	4
6,304	4,620	0,966	1,364	M1	6
6,170	4,290	0,961	1,438	M1	8
5,993	4,400	0,979	1,362	M1	3
6,169	4,510	0,962	1,368	M1	6
5,918	4,290	0,982	1,379	M1	5
6,634	4,544	0,944	1,460	M1	8
6,757	4,658	0,927	1,451	M1	14
6,387	4,400	0,968	1,452	M1	4
5,871	4,290	0,978	1,369	M1	6
6,367	4,400	0,953	1,447	M1	5

6,259	4,400	0,952	1,423	M1	5
6,189	3,960	0,933	1,563	M1	7
5,889	4,400	0,991	1,338	M1	6
6,769	4,720	0,941	1,434	M1	7
6,438	4,400	0,948	1,463	M1	8
6,238	4,232	0,957	1,474	M1	3
6,341	4,366	0,959	1,452	M1	7
6,483	4,388	0,962	1,477	M1	9
6,457	4,515	0,976	1,430	M1	15
6,363	4,588	0,972	1,387	M1	17
5,927	4,070	0,968	1,456	M1	12
6,222	4,466	0,961	1,393	M1	7
5,877	4,311	0,973	1,363	M1	6
6,197	4,510	0,985	1,374	M1	11
6,148	4,232	0,944	1,452	M1	2
5,927	4,290	0,966	1,382	M1	5
5,967	4,310	0,950	1,384	M1	12
5,636	3,999	0,965	1,409	M1	5
6,157	4,310	0,964	1,428	M1	10
6,237	4,466	0,948	1,397	M1	5
6,301	4,388	0,956	1,436	M1	6
6,238	4,466	0,960	1,397	M1	8
6,792	5,895	0,925	1,152	M1	9
6,125	4,379	0,953	1,399	M1	11
6,237	4,466	0,948	1,397	M1	5
6,301	4,388	0,956	1,436	M1	6
6,238	4,466	0,960	1,397	M1	8
6,125	4,379	0,953	1,399	M1	12
6,303	4,474	0,963	1,409	M1	6
5,772	4,400	0,977	1,312	M1	3
6,162	4,290	0,960	1,436	M1	4
6,618	4,390	0,942	1,508	M1	5
6,240	4,290	0,958	1,455	M1	10
6,277	4,077	0,935	1,540	M1	12
6,199	4,423	0,963	1,401	M1	13
5,786	4,394	0,989	1,317	M1	14
6,057	4,544	0,973	1,333	M1	16
5,418	3,999	0,966	1,355	M1	3
6,278	4,180	0,946	1,502	M1	5
5,889	4,189	0,958	1,406	M1	9
6,111	4,290	0,947	1,425	M1	11
5,748	4,070	0,975	1,412	M1	12
6,140	4,180	0,964	1,469	M1	14
6,218	4,400	0,949	1,413	M1	5
6,171	4,264	0,957	1,447	M1	6
6,197	4,400	0,954	1,408	M1	8
6,257	4,544	0,974	1,377	M1	7
6,157	4,311	0,920	1,428	M1	5
6,427	4,314	0,921	1,490	M1	6
5,744	4,070	0,958	1,411	M1	9
6,222	4,311	0,937	1,444	M1	10
6,034	4,180	0,953	1,443	M1	12

6,170	4,180	0,937	1,476	M1	13
6,062	4,290	0,977	1,413	M1	5
5,845	4,180	0,948	1,398	M1	10
6,277	4,311	0,955	1,456	M1	4
6,482	4,250	0,934	1,525	M1	5
5,994	4,264	0,956	1,406	M1	6
5,826	4,290	0,977	1,358	M1	7
6,182	4,228	0,940	1,462	M1	7
5,473	3,984	0,980	1,374	M1	4
5,965	4,235	0,950	1,408	M1	6
6,148	4,155	0,932	1,480	M1	7
6,218	4,551	0,953	1,366	M1	5
6,096	4,290	0,962	1,421	M1	9
6,063	4,510	0,948	1,344	M1	7
5,936	4,155	0,972	1,429	M1	11
6,021	4,699	0,966	1,281	M1	7
5,718	4,180	0,976	1,368	M1	5
5,686	4,180	0,962	1,360	M1	9
5,786	4,077	0,970	1,419	M1	10
5,977	4,290	0,953	1,393	M1	4
5,636	3,922	0,982	1,437	M1	7
5,815	4,400	0,990	1,321	M1	9
6,164	4,393	0,970	1,403	M1	7
6,317	4,310	0,925	1,466	M1	8
6,071	4,510	0,991	1,346	M1	9
6,057	4,155	0,963	1,458	M1	13
6,118	4,423	0,958	1,383	M1	3
6,406	4,157	0,915	1,541	M1	6
6,032	4,155	0,958	1,452	M1	5
6,556	4,489	0,951	1,461	M1	5
6,157	4,232	0,953	1,455	M1	9
6,469	4,466	0,949	1,448	M1	10
5,922	5,170	0,980	1,146	M1	2
6,266	4,620	0,981	1,356	M1	7
7,148	5,167	0,901	1,383	M1	8
6,227	4,620	0,906	1,348	M1	12
6,454	4,388	0,951	1,471	M1	14
6,409	4,395	0,929	1,458	M1	16
6,257	4,425	0,957	1,414	M1	18
6,266	4,620	0,981	1,356	M1	8
6,227	4,620	0,906	1,348	M1	14
6,454	4,388	0,951	1,471	M1	16
6,409	4,395	0,929	1,458	M1	18
6,257	4,425	0,957	1,414	M1	20
5,934	4,217	0,943	1,407	M1	6
6,032	5,120	0,949	1,178	M1	7
6,096	4,269	0,964	1,428	M1	8
5,899	4,070	0,951	1,449	M1	16
6,167	4,233	0,944	1,457	M1	5
6,111	4,180	0,943	1,462	M1	11
6,211	4,516	0,958	1,375	M1	12
5,984	4,180	0,954	1,432	M1	4

5,737	4,070	0,963	1,409	M1	9
5,967	4,310	0,952	1,384	M1	6
6,297	4,180	0,940	1,507	M1	10
5,971	4,424	0,976	1,350	M1	5
5,607	3,960	0,972	1,416	M1	7
6,358	4,203	0,926	1,513	M1	8
6,358	4,203	0,926	1,513	M1	9
6,713	4,350	0,932	1,543	M1	7
6,602	4,620	0,953	1,429	M1	8
6,096	4,290	0,963	1,421	M1	5
6,300	4,466	0,960	1,411	M1	13
5,852	4,405	0,922	1,328	M1	3
6,483	4,388	0,946	1,477	M1	8
6,406	4,400	0,977	1,456	M1	10
5,480	3,850	0,974	1,423	M1	14
6,221	4,388	0,956	1,418	M1	17
6,147	4,233	0,949	1,452	M1	4
6,083	4,290	0,958	1,418	M1	9
6,218	4,180	0,928	1,487	M1	5
5,953	4,180	0,976	1,424	M1	10
6,211	4,544	0,968	1,367	M1	11
6,310	4,699	0,977	1,343	M1	14
5,871	4,498	0,962	1,305	M1	5
6,170	4,730	0,933	1,304	M1	9
5,882	3,999	0,955	1,471	M1	13
6,333	4,462	0,944	1,419	M1	5
6,643	5,077	0,930	1,308	M1	14
6,333	4,462	0,944	1,419	M1	5
6,064	4,544	0,975	1,335	M1	6
6,317	4,950	0,922	1,276	M1	7
6,218	4,510	0,963	1,379	M1	6
6,222	4,310	0,945	1,443	M1	8
6,111	4,400	0,965	1,389	M1	6
6,094	4,310	0,955	1,414	M1	8
6,237	4,574	0,978	1,364	M1	9
6,134	4,489	0,982	1,366	M1	7
5,826	4,070	0,950	1,431	M1	9
6,167	4,699	0,978	1,312	M1	4
5,899	4,290	0,965	1,375	M1	9
6,454	5,307	0,927	1,216	M1	10
6,210	4,466	0,955	1,391	M1	12
6,031	4,466	0,977	1,350	M1	18
6,167	4,699	0,978	1,312	M1	4
5,899	4,290	0,965	1,375	M1	9
6,210	4,466	0,955	1,391	M1	13
6,031	4,466	0,977	1,350	M1	19
6,483	4,542	0,955	1,427	M1	4
6,034	4,400	0,961	1,371	M1	5
6,218	4,180	0,956	1,487	M1	7
6,162	4,290	0,965	1,436	M1	10
6,117	4,518	0,980	1,354	M1	13
6,400	4,155	0,921	1,540	M1	14

6,864	5,088	0,980	1,349	M1	16
6,264	4,529	0,978	1,383	M1	20
6,260	4,510	0,964	1,388	M1	22
6,700	4,180	0,909	1,603	M1	7
6,183	4,388	0,969	1,409	M1	3
6,021	4,400	0,986	1,368	M1	4
6,368	4,476	0,931	1,423	M1	5
5,922	4,453	0,976	1,330	M1	7
6,754	4,180	0,931	1,616	M1	17
6,618	4,354	0,945	1,520	M1	6
6,169	4,603	0,973	1,340	M1	12
6,264	4,310	0,976	1,453	M1	4
6,324	4,400	0,937	1,437	M1	6
5,937	4,388	0,968	1,353	M1	9
6,563	4,310	0,940	1,523	M1	8
6,575	4,430	0,950	1,484	M1	7
6,021	4,180	0,952	1,440	M1	5
5,927	4,290	0,991	1,382	M1	6
5,956	4,510	0,983	1,321	M1	7
6,553	4,544	0,938	1,442	M1	11
6,381	4,406	0,920	1,448	M1	15
6,363	4,777	0,951	1,332	M1	27
6,266	4,290	0,954	1,461	M1	28
5,927	4,290	0,976	1,382	M1	3
6,156	4,311	0,963	1,428	M1	10
6,381	4,290	0,937	1,487	M1	14
5,927	4,400	0,956	1,347	M1	15
6,237	4,339	0,964	1,438	M1	3
6,457	4,529	0,948	1,426	M1	7
5,772	4,496	0,994	1,284	M1	9
6,459	4,474	0,943	1,444	M1	11
6,275	4,510	0,970	1,391	M1	17
5,683	4,180	0,965	1,360	M1	6
6,483	4,388	0,938	1,478	M1	7
6,623	4,400	0,917	1,505	M1	9
6,428	4,398	0,945	1,462	M1	11
6,222	4,188	0,940	1,486	M1	13
6,072	4,620	0,989	1,314	M1	19
6,124	4,510	0,953	1,358	M1	22
6,364	4,466	0,969	1,425	M1	23
6,656	4,620	0,941	1,441	M1	6
5,977	4,180	0,967	1,430	M1	8
6,317	4,264	0,965	1,482	M1	5
6,003	4,388	0,968	1,368	M1	6
5,977	4,290	0,963	1,393	M1	7
5,841	4,310	0,961	1,355	M1	8
6,509	4,510	0,942	1,443	M1	13
6,120	4,466	0,970	1,370	M1	18
6,895	4,289	0,910	1,608	M1	7
5,967	4,155	0,971	1,436	M1	9
6,373	4,388	0,964	1,452	M1	6
6,400	4,700	0,970	1,362	M1	8

6,428	4,724	0,964	1,361	M1	11
6,509	4,290	0,957	1,517	M1	16
6,246	4,622	0,969	1,352	M1	6
6,531	4,232	0,937	1,543	M1	9
6,011	3,848	0,948	1,562	M1	3
6,463	4,310	0,939	1,499	M1	8
6,769	4,624	0,948	1,464	M1	9
6,111	4,510	0,966	1,355	M1	7
6,200	4,290	0,963	1,445	M1	8
6,613	4,290	0,943	1,541	M1	14
6,237	4,290	0,951	1,454	M1	17
6,787	4,433	0,926	1,531	M1	4
6,127	4,615	0,979	1,328	M1	5
6,218	4,290	0,957	1,449	M1	11
6,386	4,290	0,920	1,489	M1	15
6,062	4,070	0,959	1,489	M1	19
6,324	4,400	0,949	1,437	M1	10
6,417	4,689	0,969	1,369	M1	12
5,977	4,180	0,966	1,430	M1	13
6,728	4,400	0,914	1,529	M1	15
6,111	4,070	0,948	1,502	M1	17
6,373	4,533	0,965	1,406	M1	20
5,472	3,813	0,963	1,435	M1	7
6,509	4,466	0,935	1,458	M1	5
6,514	4,620	0,943	1,410	M1	12
6,140	4,383	0,965	1,401	M1	3
6,148	4,232	0,949	1,453	M1	5
5,882	4,310	0,965	1,365	M1	7
6,264	4,510	0,973	1,389	M1	12
6,059	3,986	0,935	1,520	M1	17
7,101	5,399	0,957	1,315	M1	2
6,210	4,933	0,958	1,259	M1	7
6,127	4,353	0,964	1,407	M1	10
6,157	4,232	0,947	1,455	M1	12
6,580	4,519	0,919	1,456	M1	14
6,495	4,730	0,946	1,373	M1	16
6,111	4,290	0,963	1,425	M1	17
6,584	4,620	0,961	1,425	M1	20
5,922	4,388	0,971	1,350	M1	24
6,011	4,199	0,963	1,432	M1	27
6,324	4,400	0,956	1,437	M1	3
6,278	4,699	0,963	1,336	M1	8
6,171	4,403	0,959	1,402	M1	9
5,899	3,960	0,931	1,490	M1	11
5,928	4,400	0,976	1,347	M1	6
6,164	4,366	0,959	1,412	M1	8
6,787	4,310	0,912	1,575	M1	14
5,918	4,254	0,973	1,391	M1	5
6,125	4,400	0,976	1,392	M1	12
6,062	4,290	0,945	1,413	M1	6
6,072	4,388	0,972	1,384	M1	9
5,845	4,180	0,961	1,398	M1	10

5,904	4,310	0,967	1,370	M1	16
6,336	4,155	0,940	1,525	M1	20
5,786	4,077	0,966	1,419	M1	8
6,087	4,388	0,966	1,387	M1	12
6,156	4,155	0,936	1,482	M1	14
5,852	4,070	0,963	1,438	M1	13
6,111	4,180	0,962	1,462	M1	15
6,279	4,290	0,950	1,464	M1	7
6,127	4,544	0,975	1,348	M1	8
6,062	4,290	0,965	1,413	M1	3
6,222	4,232	0,955	1,470	M1	6
6,059	4,400	0,970	1,377	M1	7
6,034	4,400	0,941	1,371	M1	11
5,772	4,329	0,983	1,333	M1	12
6,297	4,400	0,961	1,431	M1	14
6,222	4,291	0,938	1,450	M1	3
6,096	4,510	0,972	1,352	M1	7
6,127	4,273	0,965	1,434	M1	15
6,217	4,436	0,963	1,402	M1	19
6,472	4,290	0,950	1,509	M1	3
5,956	4,290	0,986	1,388	M1	10
6,835	5,211	0,915	1,312	M1	12
5,899	4,290	0,962	1,375	M1	8
6,649	4,426	0,941	1,502	M1	9
5,645	4,070	0,972	1,387	M1	3
6,364	4,232	0,910	1,504	M1	12
6,171	4,400	0,971	1,403	M1	13
6,030	4,544	0,972	1,327	M1	15
6,189	4,510	0,951	1,372	M1	4
5,743	4,290	0,962	1,339	M1	7
6,140	4,180	0,957	1,469	M1	8
6,087	4,466	0,960	1,363	M1	9
5,732	4,232	0,961	1,354	M1	11
5,971	4,354	0,974	1,372	M1	18
6,208	4,310	0,958	1,440	M1	4
6,227	4,400	0,959	1,415	M1	8
6,087	4,077	0,942	1,493	M1	5
6,699	5,633	0,904	1,189	M1	7
6,456	4,435	0,945	1,456	M1	13
6,217	4,488	0,969	1,385	M1	17
6,087	4,077	0,942	1,493	M1	5
6,456	4,435	0,945	1,456	M1	14
6,217	4,488	0,969	1,385	M1	18
6,167	4,233	0,952	1,457	M1	6
6,310	4,290	0,948	1,471	M1	3
6,337	4,466	0,966	1,419	M1	8
6,456	4,255	0,939	1,517	M1	9
6,157	4,466	0,970	1,379	M1	10
5,927	4,320	0,971	1,372	M1	1
5,977	4,400	0,973	1,358	M1	4
6,580	4,388	0,938	1,499	M1	7
6,189	4,290	0,942	1,443	M1	12

6,510	4,290	0,934	1,517	M1	15
6,157	4,388	0,957	1,403	M1	23
6,363	4,463	0,965	1,426	M1	24
6,363	4,622	0,943	1,377	M1	27
6,057	4,388	0,958	1,380	M1	35
6,211	4,544	0,957	1,367	M1	38
5,815	4,400	0,981	1,321	M1	39
6,184	4,466	0,969	1,385	M1	40
5,899	4,290	0,976	1,375	M1	4
6,023	4,290	0,964	1,404	M1	12
6,257	4,400	0,944	1,422	M1	8
6,364	4,310	0,927	1,477	M1	2
6,003	4,372	0,952	1,373	M1	7
6,622	4,290	0,933	1,544	M1	18
5,977	4,400	0,975	1,358	M1	11
5,977	4,155	0,964	1,439	M1	3
5,849	4,400	0,973	1,329	M1	11
6,463	4,466	0,941	1,447	M1	3
5,880	4,180	0,954	1,407	M1	7
6,125	4,510	0,964	1,358	M1	9
5,995	4,621	0,975	1,297	M1	14
6,218	4,620	0,972	1,346	M1	4
6,164	4,248	0,966	1,451	M1	5
5,977	4,180	0,944	1,430	M1	6
6,032	4,232	0,954	1,425	M1	6
6,373	4,232	0,939	1,506	M1	9
6,510	4,527	0,967	1,438	M1	6
6,363	4,290	0,930	1,483	M1	6
6,604	4,549	0,966	1,452	M1	11
5,787	4,303	0,971	1,345	M1	4
6,603	4,508	0,944	1,465	M1	4
6,417	4,682	0,966	1,371	M1	15
6,340	4,180	0,935	1,517	M1	17
5,880	4,290	0,984	1,371	M1	5
6,472	4,070	0,936	1,590	M1	7
6,318	4,423	0,946	1,428	M1	3
6,318	4,228	0,934	1,494	M1	4
6,168	4,451	0,964	1,386	M1	7
6,164	4,400	0,963	1,401	M1	3
6,490	4,233	0,930	1,533	M1	6
6,094	4,155	0,964	1,467	M1	9
5,967	4,077	0,943	1,464	M1	3
6,218	4,290	0,950	1,449	M1	5
5,927	3,960	0,960	1,497	M1	4
5,940	4,388	0,955	1,354	M1	8
6,266	4,400	0,942	1,424	M1	11
6,264	4,589	0,979	1,365	M1	14
6,096	4,362	0,961	1,397	M1	8
5,724	4,077	0,963	1,404	M1	6
5,426	3,910	0,976	1,388	M1	3
5,962	4,403	0,965	1,354	M1	10
6,430	4,290	0,935	1,499	M1	2

5,955	5,332	0,983	1,117	M1	10
6,218	4,132	0,941	1,505	M1	11
5,504	4,620	0,976	1,191	M1	2
6,228	4,510	0,956	1,381	M1	5
6,228	4,510	0,956	1,381	M1	6
6,454	4,155	0,923	1,553	M1	3
6,030	4,310	0,977	1,399	M1	8
5,967	4,310	0,974	1,384	M1	6
5,731	4,388	0,989	1,306	M1	9
6,278	4,290	0,957	1,463	M1	20
5,871	4,339	0,969	1,353	M1	9
6,427	4,310	0,942	1,491	M1	2
6,481	4,432	0,958	1,462	M1	9
5,081	4,070	0,917	1,248	M1	10
6,427	4,310	0,942	1,491	M1	2
6,481	4,432	0,958	1,462	M1	9
5,841	4,232	0,959	1,380	M1	6
5,827	4,233	0,970	1,377	M1	12
6,275	4,510	0,949	1,391	M1	3
6,125	4,510	0,971	1,358	M1	11
4,805	4,310	1,000	1,115	M1	6
5,127	4,070	0,969	1,260	M4	8
5,205	4,090	0,984	1,272	M4	15
5,261	3,740	0,961	1,407	M6	2
5,290	4,180	0,999	1,266	M3	3
5,296	4,180	0,950	1,267	M4	19
5,327	4,360	0,953	1,222	M5	17
5,336	3,999	0,975	1,334	M6	3
5,339	4,070	0,996	1,312	M2	14
5,380	4,068	0,985	1,322	M6	15
5,396	4,070	0,974	1,326	M2	6
5,401	3,850	0,973	1,403	M6	30
5,401	3,960	0,978	1,364	M2	4
5,419	4,400	0,908	1,232	M1	7
5,429	3,740	0,967	1,452	M4	7
5,438	4,544	1,000	1,197	M5	7
5,439	4,064	0,992	1,338	M2	6
5,445	4,180	0,989	1,303	M2	14
5,466	4,057	0,972	1,347	M2	15
5,471	3,999	0,981	1,368	M2	3
5,471	3,873	0,970	1,413	M1	9
5,471	3,873	0,970	1,413	M1	9
5,473	4,077	0,980	1,342	M4	13
5,473	4,077	0,989	1,342	M2	6
5,480	3,850	0,920	1,423	M1	8
5,486	3,850	0,954	1,425	M2	6
5,501	3,850	0,975	1,429	M4	2
5,504	4,070	0,980	1,352	M2	6
5,526	4,077	0,984	1,355	M2	6
5,532	4,070	0,981	1,359	M6	8
5,541	3,999	0,986	1,385	M2	9
5,544	4,155	0,987	1,334	M2	12

5,552	4,180	0,981	1,328	M3	5
5,552	4,070	0,967	1,364	M2	5
5,552	4,070	0,976	1,364	M2	10
5,566	4,180	0,989	1,332	M2	16
5,572	4,235	0,966	1,316	M2	11
5,573	4,136	0,969	1,347	M6	2
5,579	4,400	0,982	1,268	M6	10
5,579	4,180	0,967	1,335	M2	4
5,580	4,070	0,987	1,371	M5	9
5,580	4,070	0,974	1,371	M2	2
5,580	4,070	0,965	1,371	M6	19
5,580	3,960	0,978	1,409	M6	6
5,581	4,180	0,974	1,335	M6	4
5,581	4,077	0,978	1,369	M2	9
5,582	4,070	0,960	1,371	M4	17
5,598	4,310	0,987	1,299	M3	4
5,607	3,843	0,956	1,459	M2	8
5,607	3,850	0,978	1,456	M2	3
5,609	4,180	0,985	1,342	M5	9
5,609	4,180	0,969	1,342	M6	8
5,609	4,180	0,992	1,342	M2	10
5,609	3,850	0,946	1,457	M6	15
5,609	4,400	0,972	1,275	M3	13
5,609	4,070	0,979	1,378	M6	9
5,609	4,070	0,988	1,378	M2	6
5,619	4,077	0,988	1,378	M4	6
5,622	4,310	0,985	1,304	M6	6
5,622	4,111	0,980	1,368	M2	9
5,626	4,257	0,998	1,321	M2	7
5,626	4,248	0,995	1,324	M2	16
5,629	3,960	0,954	1,422	M2	6
5,629	4,290	0,985	1,312	M2	9
5,634	4,233	0,992	1,331	M5	6
5,634	4,077	0,968	1,382	M6	7
5,636	4,197	0,976	1,343	M4	3
5,636	4,155	0,983	1,356	M2	5
5,637	4,388	0,991	1,285	M2	6
5,637	4,232	0,981	1,332	M6	7
5,637	4,290	0,968	1,314	M3	6
5,637	4,070	0,968	1,385	M4	10
5,637	3,960	0,965	1,423	M6	4
5,637	4,070	0,981	1,385	M6	11
5,645	4,388	0,989	1,286	M5	12
5,658	3,850	0,965	1,470	M1	3
5,658	4,070	0,979	1,390	M6	6
5,661	4,155	0,971	1,362	M3	7
5,661	4,310	0,983	1,313	M2	5
5,661	3,999	0,981	1,416	M2	10
5,661	3,999	0,969	1,416	M2	3
5,672	4,264	0,982	1,330	M2	10
5,672	4,070	0,984	1,394	M2	7
5,673	4,134	0,977	1,372	M2	5

5,673	4,070	0,968	1,394	M2	5
5,673	3,960	0,967	1,433	M6	4
5,676	4,388	0,981	1,293	M3	11
5,676	4,399	0,996	1,290	M2	7
5,676	4,197	0,990	1,353	M2	7
5,677	4,077	0,982	1,392	M2	8
5,683	4,145	0,991	1,371	M2	10
5,683	4,290	0,983	1,325	M5	12
5,683	4,237	0,981	1,341	M6	9
5,686	4,400	0,977	1,292	M4	11
5,686	4,180	0,979	1,360	M5	4
5,686	3,850	0,961	1,477	M4	7
5,691	4,310	0,992	1,320	M6	8
5,691	4,148	0,972	1,372	M2	35
5,697	4,232	0,983	1,346	M2	9
5,698	3,960	0,952	1,439	M2	6
5,711	4,269	0,981	1,338	M6	14
5,711	3,960	0,980	1,442	M6	13
5,711	4,282	0,994	1,334	M4	6
5,711	4,070	0,965	1,403	M6	3
5,715	4,180	0,979	1,367	M3	7
5,715	4,180	0,987	1,367	M4	11
5,715	4,180	0,980	1,367	M2	19
5,715	4,070	0,965	1,404	M2	9
5,715	4,180	0,978	1,367	M6	18
5,715	4,282	0,980	1,335	M5	3
5,717	4,094	0,977	1,396	M4	14
5,717	4,290	0,974	1,333	M2	15
5,717	4,180	0,977	1,368	M5	9
5,719	4,237	0,984	1,350	M2	2
5,719	4,052	0,973	1,411	M3	13
5,719	4,290	0,976	1,333	M6	7
5,724	4,077	0,972	1,404	M4	4
5,724	3,999	0,964	1,431	M2	7
5,724	4,363	0,975	1,312	M4	13
5,724	4,232	0,976	1,352	M2	6
5,724	4,077	0,978	1,404	M4	14
6,510	4,356	0,956	1,494	M1	9
6,303	4,510	0,967	1,398	M1	10
6,183	4,388	0,976	1,409	M1	16
5,940	4,466	0,967	1,330	M1	5
6,182	4,466	0,970	1,384	M1	7
5,890	4,400	0,973	1,339	M1	11
6,210	4,610	0,959	1,347	M1	3
6,510	4,180	0,937	1,557	M1	8
6,556	4,790	0,954	1,369	M1	13
6,454	4,544	0,959	1,421	M1	7
6,488	4,730	0,962	1,372	M1	8
6,427	4,275	0,918	1,504	M1	10
5,867	4,155	0,979	1,412	M1	11
6,373	4,544	0,953	1,403	M1	12
6,483	4,388	0,941	1,477	M1	13

6,333	4,354	0,964	1,454	M1	4
6,274	4,400	0,964	1,426	M1	5
6,083	4,620	0,976	1,317	M1	7
6,182	4,527	0,977	1,366	M1	8
6,400	4,466	0,952	1,433	M1	4
5,882	4,388	0,972	1,340	M1	6
6,406	4,290	0,943	1,493	M1	8
6,358	4,589	0,973	1,386	M1	10
6,359	4,559	0,972	1,395	M1	12
5,918	4,544	0,976	1,303	M1	19
5,490	4,180	0,951	1,313	M1	2
6,168	4,730	0,973	1,304	M1	3
6,237	4,699	0,948	1,327	M1	6
6,262	4,383	0,966	1,429	M1	7
6,170	4,290	0,972	1,438	M1	12
6,062	4,510	0,973	1,344	M1	18
6,223	4,544	0,978	1,370	M1	20
6,168	4,730	0,973	1,304	M1	4
6,237	4,699	0,948	1,327	M1	7
6,262	4,383	0,966	1,429	M1	8
6,170	4,290	0,972	1,438	M1	13
6,062	4,510	0,973	1,344	M1	19
6,223	4,544	0,978	1,370	M1	21
6,264	4,706	0,980	1,331	M1	3
6,264	4,353	0,971	1,439	M1	7
6,222	4,544	0,973	1,369	M1	4
6,184	4,462	0,969	1,386	M1	5
5,827	4,250	0,972	1,371	M1	7
6,352	4,400	0,971	1,444	M1	8
6,032	4,544	0,978	1,328	M1	5
6,643	4,510	0,921	1,473	M1	7
5,772	4,203	0,977	1,373	M1	9
6,827	4,622	0,935	1,477	M1	4
6,643	4,840	0,940	1,372	M1	6
6,111	4,400	0,968	1,389	M1	13
6,021	4,510	0,985	1,335	M1	9
6,031	4,444	0,985	1,357	M1	3
5,927	4,070	0,951	1,456	M1	5
5,917	4,290	0,974	1,379	M1	7
6,357	4,254	0,946	1,494	M1	8
5,955	3,911	0,941	1,522	M1	9
6,386	4,290	0,917	1,489	M1	10
6,944	4,525	0,940	1,535	M1	15
6,168	4,180	0,956	1,476	M1	16
5,927	4,241	0,972	1,398	M1	17
6,277	4,449	0,963	1,411	M1	18
5,918	4,364	0,953	1,356	M1	19
6,673	4,639	0,958	1,438	M1	20
5,882	4,378	0,974	1,343	M1	21
6,148	4,307	0,964	1,427	M1	23
5,541	3,936	0,974	1,408	M1	24
6,593	4,593	0,958	1,435	M1	29

6,030	4,313	0,983	1,398	M1	30
6,238	4,408	0,951	1,415	M1	31
5,937	4,302	0,967	1,380	M1	36
6,241	4,400	0,958	1,418	M1	40
6,030	4,400	0,975	1,371	M1	41
6,032	4,476	0,951	1,348	M1	42
5,865	4,077	0,960	1,439	M1	43
5,787	4,232	0,971	1,367	M1	2
6,030	4,444	0,983	1,357	M1	6
5,772	4,466	0,998	1,292	M1	7
6,118	4,433	0,973	1,380	M1	14
6,378	4,800	0,985	1,329	M1	5
6,083	4,180	0,957	1,455	M1	7
5,955	4,180	0,975	1,425	M1	3
6,065	4,621	0,991	1,312	M1	6
6,227	4,510	0,980	1,381	M1	8
5,982	4,622	0,965	1,294	M1	15
5,598	3,999	0,971	1,400	M1	16
5,917	4,510	0,974	1,312	M1	4
6,210	4,388	0,970	1,415	M1	6
6,278	4,688	0,959	1,339	M1	7
6,381	4,290	0,946	1,487	M1	9
6,030	4,510	0,980	1,337	M1	10
6,189	4,180	0,924	1,481	M1	18
6,275	4,493	0,968	1,397	M1	19
6,111	4,367	0,971	1,399	M1	20
6,264	4,474	0,961	1,400	M1	21
6,072	4,339	0,967	1,400	M1	23
6,618	4,550	0,952	1,455	M1	27
6,127	4,544	0,977	1,349	M1	34
5,928	4,400	0,975	1,347	M1	36
5,971	4,388	0,976	1,361	M1	4
6,444	4,199	0,934	1,534	M1	6
6,363	4,503	0,953	1,413	M1	9
6,490	4,777	0,948	1,359	M1	10
6,265	4,400	0,963	1,424	M1	11
6,128	4,606	0,973	1,330	M1	17
6,058	4,589	0,968	1,320	M1	20
6,378	4,645	0,978	1,373	M1	21
6,514	4,180	0,953	1,558	M1	22
5,918	4,386	0,981	1,350	M1	27
6,006	4,510	0,959	1,332	M1	5
6,218	4,180	0,955	1,487	M1	6
5,971	4,621	0,977	1,292	M1	3
6,263	4,388	0,960	1,427	M1	5
6,301	4,544	0,951	1,387	M1	10
6,005	4,510	0,972	1,332	M1	11
5,898	4,388	0,959	1,344	M1	13
6,221	4,388	0,967	1,418	M1	15
6,332	4,466	0,952	1,418	M1	18
6,532	4,699	0,955	1,390	M1	20
6,363	4,683	0,952	1,359	M1	1

6,120	4,311	0,958	1,420	M1	4
5,865	4,400	1,000	1,333	M1	7
6,217	4,544	0,969	1,368	M1	11
6,184	4,388	0,952	1,409	M1	12
5,845	4,400	0,970	1,328	M1	13
6,317	4,589	0,983	1,377	M1	14
6,156	4,466	0,972	1,378	M1	18
6,317	4,496	0,970	1,405	M1	19
6,333	4,932	0,960	1,284	M1	25
6,318	4,544	0,968	1,390	M1	26
6,403	4,544	0,962	1,409	M1	3
5,918	4,290	0,978	1,379	M1	4
6,510	4,752	0,969	1,370	M1	7
6,481	4,653	0,940	1,393	M1	9
6,057	4,466	0,983	1,356	M1	11
6,197	4,510	0,965	1,374	M1	15
5,918	4,354	0,975	1,359	M1	17
6,096	4,808	0,977	1,268	M1	19
6,067	4,232	0,950	1,433	M1	21
6,403	4,687	0,978	1,366	M1	5
6,663	4,628	0,940	1,440	M1	6
6,171	4,620	0,958	1,336	M1	7
6,057	4,466	0,975	1,356	M1	8
6,197	4,510	0,967	1,374	M1	12
5,972	4,400	0,987	1,357	M1	14
6,199	4,836	0,970	1,282	M1	15
6,247	4,388	0,959	1,424	M1	17
6,157	4,544	0,965	1,355	M1	21
6,274	4,477	0,943	1,402	M1	22
6,756	4,349	0,925	1,554	M1	3
6,490	4,686	0,951	1,385	M1	5
6,358	4,466	0,973	1,424	M1	9
5,962	5,024	0,943	1,187	M1	10
6,164	4,388	0,974	1,405	M1	11
6,262	4,388	0,947	1,427	M1	12
6,318	4,544	0,973	1,390	M1	22
6,067	4,310	0,964	1,407	M1	24
6,247	4,466	0,958	1,399	M1	2
5,850	4,311	0,972	1,357	M1	3
5,772	4,730	0,936	1,220	M1	5
6,164	4,388	0,959	1,405	M1	7
6,483	4,686	0,950	1,383	M1	12
5,870	4,290	0,961	1,368	M1	17
6,071	4,400	0,979	1,380	M1	3
6,042	4,284	0,942	1,410	M1	8
6,457	4,602	0,933	1,403	M1	10
6,096	4,290	0,976	1,421	M1	11
6,454	4,621	0,943	1,397	M1	15
6,324	4,730	0,948	1,337	M1	3
6,030	4,290	0,959	1,406	M1	5
6,357	4,525	0,969	1,405	M1	7
5,928	4,400	0,968	1,347	M1	3

6,062	4,290	0,971	1,413	M1	4
6,171	4,536	0,967	1,361	M1	5
6,332	3,960	0,937	1,599	M1	7
6,096	4,070	0,960	1,498	M1	11
5,890	4,510	0,976	1,306	M1	16
6,184	4,466	0,969	1,385	M1	18
5,953	4,510	0,969	1,320	M1	6
6,094	4,466	0,961	1,365	M1	7
5,977	4,180	0,960	1,430	M1	9
6,237	4,536	0,967	1,375	M1	10
6,266	4,400	0,966	1,424	M1	12
6,544	4,519	0,943	1,448	M1	23
6,218	4,400	0,950	1,413	M1	7
6,096	4,290	0,986	1,421	M1	9
6,490	4,388	0,954	1,479	M1	10
6,030	4,350	0,974	1,386	M1	7
5,985	4,400	0,970	1,360	M1	9
6,042	4,290	0,962	1,408	M1	12
6,428	4,462	0,967	1,441	M1	13
6,087	4,310	0,959	1,412	M1	4
6,237	4,544	0,966	1,373	M1	5
6,071	4,220	0,957	1,439	M1	6
6,083	4,290	0,964	1,418	M1	10
6,168	4,388	0,966	1,406	M1	11
6,071	4,290	0,968	1,415	M1	17
6,223	4,544	0,964	1,370	M1	21
6,998	4,640	0,905	1,508	M1	3
6,544	4,583	0,959	1,428	M1	4
6,391	4,466	0,973	1,431	M1	6
5,977	4,180	0,958	1,430	M1	8
6,353	4,595	0,950	1,382	M1	12
6,183	4,233	0,948	1,461	M1	14
6,246	4,400	0,958	1,420	M1	15
6,363	4,390	0,965	1,450	M1	18
5,865	4,400	0,989	1,333	M1	19
6,278	4,180	0,946	1,502	M1	24
5,850	4,180	0,971	1,399	M1	25
6,156	4,311	0,960	1,428	M1	26
6,164	4,284	0,952	1,439	M1	4
5,814	4,155	0,966	1,399	M1	7
6,112	4,554	0,961	1,342	M1	8
6,257	4,269	0,935	1,465	M1	13
6,247	4,155	0,936	1,504	M1	14
6,087	4,298	0,957	1,416	M1	16
7,000	4,566	0,916	1,533	M1	17
6,062	4,070	0,955	1,489	M1	19
6,062	4,290	0,966	1,413	M1	20
6,112	4,290	0,955	1,425	M1	22
6,018	4,544	0,989	1,325	M1	26
6,164	4,284	0,952	1,439	M1	4
5,814	4,155	0,966	1,399	M1	7
6,112	4,554	0,961	1,342	M1	8

6,257	4,269	0,935	1,465	M1	13
6,247	4,155	0,936	1,504	M1	14
6,087	4,298	0,957	1,416	M1	16
6,062	4,070	0,955	1,489	M1	21
6,062	4,290	0,966	1,413	M1	22
6,112	4,290	0,955	1,425	M1	24
6,018	4,544	0,989	1,325	M1	28
5,911	4,519	0,977	1,308	M1	3
6,463	4,372	0,906	1,478	M1	5
6,034	4,180	0,971	1,443	M1	6
6,210	4,466	0,951	1,391	M1	10
6,071	4,233	0,949	1,434	M1	12
5,852	4,180	0,987	1,400	M1	14
6,125	4,702	0,984	1,303	M1	16
6,157	4,232	0,948	1,455	M1	17
6,438	4,138	0,921	1,556	M1	19
6,034	4,400	0,972	1,371	M1	21
6,310	4,357	0,968	1,448	M1	5
5,772	4,180	0,981	1,381	M1	7
6,400	4,471	0,952	1,431	M1	8
6,034	4,400	0,968	1,371	M1	6
6,260	4,180	0,933	1,498	M1	8
6,472	4,320	0,948	1,498	M1	9
6,863	5,047	0,934	1,360	M1	11
6,171	4,450	0,967	1,387	M1	13
6,332	4,622	0,957	1,370	M1	14
5,716	4,077	0,971	1,402	M1	15
5,815	4,290	0,978	1,355	M1	17
5,865	4,233	0,993	1,386	M1	18
6,032	4,478	0,974	1,347	M1	24
6,034	4,400	0,968	1,371	M1	6
6,260	4,180	0,933	1,498	M1	8
6,472	4,320	0,948	1,498	M1	9
6,171	4,450	0,967	1,387	M1	14
6,332	4,622	0,957	1,370	M1	15
5,716	4,077	0,971	1,402	M1	16
5,815	4,290	0,978	1,355	M1	18
5,865	4,233	0,993	1,386	M1	19
6,032	4,478	0,974	1,347	M1	25
6,356	4,640	0,973	1,370	M1	3
6,211	4,399	0,958	1,412	M1	4
5,965	4,356	0,983	1,369	M1	8
5,765	4,070	0,979	1,417	M1	9
6,303	4,297	0,951	1,467	M1	10
5,871	4,290	0,968	1,369	M1	13
5,793	4,180	0,967	1,386	M1	16
6,017	4,233	0,984	1,421	M1	18
6,112	4,456	0,970	1,372	M1	3
5,954	4,290	0,961	1,388	M1	5
5,842	4,310	0,962	1,355	M1	7
6,018	4,544	0,964	1,324	M1	11
6,218	4,180	0,940	1,488	M1	5

6,171	4,180	0,969	1,476	M1	7
5,955	4,440	0,984	1,341	M1	8
5,786	4,152	0,962	1,394	M1	10
6,030	4,233	0,966	1,425	M1	13
6,003	4,311	0,963	1,393	M1	16
6,545	4,400	0,928	1,487	M1	17
6,217	4,388	0,957	1,417	M1	18
6,072	4,517	0,954	1,344	M1	22
6,120	4,463	0,953	1,371	M1	23
6,200	4,400	0,969	1,409	M1	24
6,156	4,388	0,962	1,403	M1	3
6,148	4,155	0,955	1,480	M1	6
6,128	4,462	0,980	1,373	M1	7
6,264	4,354	0,953	1,439	M1	8
6,246	4,544	0,958	1,375	M1	10
5,940	4,363	0,964	1,361	M1	11
6,508	4,550	0,948	1,431	M1	14
5,918	4,343	0,979	1,363	M1	15
5,927	4,400	0,979	1,347	M1	17
6,659	4,730	0,974	1,408	M1	21
6,018	4,423	0,980	1,360	M1	7
5,845	4,180	0,968	1,398	M1	4
6,208	4,544	0,973	1,366	M1	7
6,111	4,351	0,951	1,404	M1	9
6,622	4,217	0,939	1,570	M1	10
6,749	4,392	0,913	1,537	M1	12
5,845	4,180	0,968	1,398	M1	4
6,208	4,544	0,973	1,366	M1	7
6,111	4,351	0,951	1,404	M1	9
6,622	4,217	0,939	1,570	M1	10
6,495	4,840	0,965	1,342	M1	7
6,310	4,699	0,948	1,343	M1	8
6,162	4,510	0,977	1,366	M1	10
6,278	4,180	0,936	1,502	M1	5
6,246	4,388	0,950	1,423	M1	6
6,298	4,510	0,952	1,396	M1	8
6,172	4,257	0,966	1,450	M1	9
6,427	4,466	0,963	1,439	M1	10
6,277	4,466	0,950	1,406	M1	11
6,031	4,466	0,967	1,350	M1	12
5,940	4,155	0,958	1,430	M1	3
6,018	4,164	0,972	1,445	M1	11
6,164	4,510	0,981	1,367	M1	5
5,765	4,070	0,956	1,416	M1	1
5,748	4,229	0,967	1,359	M1	12
5,880	3,850	0,918	1,527	M1	3
5,818	4,021	0,942	1,447	M1	5
5,686	3,960	0,958	1,436	M1	7
5,821	4,070	0,958	1,430	M1	8
5,645	3,850	0,941	1,466	M1	9
5,504	3,850	0,954	1,430	M1	15
5,940	4,155	0,946	1,430	M1	16

6,064	4,311	0,957	1,407	M1	17
6,331	4,400	0,968	1,439	M1	3
6,275	3,960	0,932	1,584	M1	5
6,064	4,290	0,975	1,414	M1	6
6,083	4,510	0,967	1,349	M1	11
5,937	4,388	0,981	1,353	M1	3
5,814	4,290	0,966	1,355	M1	4
5,845	4,290	0,972	1,363	M1	12
6,463	4,383	0,958	1,475	M1	14
6,003	4,233	0,950	1,418	M1	6
6,111	4,400	0,954	1,389	M1	6
6,359	4,544	0,933	1,399	M1	8
5,877	4,310	0,975	1,364	M1	14
6,057	4,155	0,950	1,458	M1	5
6,264	4,590	0,980	1,365	M1	11
5,793	4,180	0,958	1,386	M1	5
6,517	4,150	0,928	1,570	M1	4
6,189	4,290	0,956	1,443	M1	7
6,387	4,400	0,940	1,452	M1	11
6,208	4,232	0,941	1,467	M1	5
5,918	4,132	0,950	1,432	M1	6
6,018	4,311	0,964	1,396	M1	13
5,278	3,688	0,970	1,431	M1	4
5,737	4,180	0,978	1,372	M1	8
6,061	4,070	0,941	1,489	M1	5
5,977	3,960	0,941	1,509	M1	6
5,918	4,157	0,970	1,424	M1	15
6,140	4,180	0,949	1,469	M1	7
6,073	4,310	0,945	1,409	M1	6
6,071	4,510	0,982	1,346	M1	10
5,570	4,070	0,961	1,369	M1	5
5,850	4,400	0,976	1,330	M1	11
6,058	4,155	0,958	1,458	M1	5
5,174	4,144	0,963	1,248	M1	6
5,953	4,180	0,947	1,424	M1	12
6,806	4,840	0,950	1,406	M1	4
5,882	4,155	0,963	1,416	M1	5
5,748	4,363	0,968	1,318	M1	7
5,927	4,070	0,952	1,456	M1	9
5,814	4,232	0,959	1,374	M1	15
6,096	4,180	0,950	1,458	M1	5
6,278	4,291	0,946	1,463	M1	6
6,005	4,070	0,959	1,475	M1	7
6,524	4,310	0,948	1,514	M1	9
7,193	4,310	0,908	1,669	M1	11
6,265	4,509	0,950	1,389	M1	12
5,686	4,180	0,983	1,360	M1	15
6,171	4,400	0,966	1,403	M1	17
6,563	4,310	0,943	1,523	M1	19
6,483	4,311	0,942	1,504	M1	20
6,170	4,070	0,924	1,516	M1	4
5,913	4,310	0,967	1,372	M1	7

6,275	4,400	0,973	1,426	M1	3
6,318	4,155	0,944	1,521	M1	5
6,157	3,921	0,933	1,570	M1	8
5,982	4,232	0,961	1,413	M1	11
5,922	4,310	0,975	1,374	M1	13
6,237	4,339	0,964	1,438	M1	18
6,073	4,192	0,941	1,449	M1	20
5,724	4,077	0,961	1,404	M4	13
5,725	4,070	0,975	1,407	M6	10
5,725	4,308	0,992	1,329	M6	9
5,725	4,290	0,964	1,335	M6	19
5,731	4,368	0,980	1,312	M1	7
5,731	4,233	0,981	1,354	M6	12
5,731	4,123	0,968	1,390	M2	15
5,731	3,844	0,948	1,491	M4	8
5,736	4,290	0,973	1,337	M6	8
5,737	3,960	0,950	1,449	M6	10
5,743	4,180	0,984	1,374	M4	4
5,743	4,070	0,966	1,411	M2	3
5,748	4,443	0,996	1,294	M5	11
5,748	4,489	0,989	1,281	M5	10
5,751	4,520	0,990	1,272	M5	6
5,751	4,232	0,979	1,359	M3	8
5,751	4,155	0,978	1,384	M6	5
5,760	4,544	0,968	1,268	M5	10
5,760	4,232	0,965	1,361	M6	9
5,764	4,400	0,980	1,310	M2	15
5,764	4,290	0,966	1,344	M5	4
5,764	4,180	0,978	1,379	M2	3
5,765	4,180	0,960	1,379	M2	8
5,765	4,311	0,964	1,337	M2	8
5,772	4,233	0,976	1,364	M6	3
5,772	3,999	0,974	1,443	M4	5
5,772	4,068	0,970	1,419	M6	24
5,772	4,466	0,984	1,292	M5	5
5,772	4,114	0,966	1,403	M2	4
5,772	4,232	0,982	1,364	M5	24
5,772	4,204	0,991	1,373	M3	17
5,772	4,070	0,972	1,418	M2	8
5,772	3,831	0,946	1,506	M2	4
5,772	4,257	0,984	1,356	M2	6
5,772	4,395	0,985	1,313	M3	5
5,772	4,354	0,982	1,326	M6	13
5,776	4,070	0,972	1,419	M5	15
5,777	4,070	0,976	1,419	M4	9
5,786	4,433	0,983	1,305	M5	10
5,786	4,264	0,989	1,357	M4	3
5,786	4,189	0,964	1,381	M2	5
5,786	4,142	0,967	1,397	M1	9
5,786	4,142	0,967	1,397	M1	10
5,786	4,379	0,983	1,321	M2	16
5,786	4,290	0,989	1,349	M6	7

5,786	4,070	0,968	1,422	M3	10
5,786	4,180	0,983	1,384	M4	14
5,786	4,388	0,982	1,319	M3	13
5,786	4,544	0,941	1,273	M6	7
5,787	4,311	0,988	1,342	M2	16
5,787	4,233	0,978	1,367	M4	12
5,787	4,233	0,962	1,367	M6	7
5,787	3,999	0,978	1,447	M5	14
5,787	3,999	0,974	1,447	M6	6
5,787	4,155	0,977	1,393	M6	6
5,787	4,155	0,979	1,393	M5	14
5,787	4,077	0,965	1,420	M2	6
5,787	4,388	0,970	1,319	M6	7
5,787	4,388	0,980	1,319	M2	8
5,787	4,077	0,976	1,420	M5	3
5,787	4,077	0,968	1,420	M2	6
5,793	4,290	0,966	1,350	M3	8
5,793	4,180	0,961	1,386	M1	12
5,793	4,070	0,966	1,423	M4	5
5,793	4,070	0,967	1,423	M6	10
5,793	4,180	0,965	1,386	M2	8
5,793	4,070	0,952	1,423	M6	5
5,793	4,070	0,967	1,423	M3	10
5,793	4,290	0,976	1,350	M1	5
5,807	4,400	0,982	1,320	M5	8
5,807	3,960	0,965	1,466	M2	6
5,812	4,299	0,987	1,352	M6	3
5,812	4,232	0,983	1,373	M2	2
5,812	4,155	0,985	1,399	M2	4
5,814	4,311	0,980	1,349	M5	5
5,814	4,290	0,976	1,355	M5	5
5,814	4,299	0,989	1,353	M1	6
5,814	4,180	0,957	1,391	M6	27
5,814	4,180	0,964	1,391	M4	4
5,814	3,960	0,939	1,468	M2	5
5,814	4,310	0,958	1,349	M3	7
5,814	4,155	0,946	1,399	M3	10
5,814	4,232	0,967	1,374	M6	4
5,814	4,155	0,977	1,399	M5	4
5,815	4,290	0,989	1,355	M4	10
5,815	4,400	0,992	1,321	M2	18
5,818	4,552	0,990	1,278	M3	11
5,818	4,257	0,977	1,367	M6	5
5,818	4,299	0,984	1,354	M2	13
5,819	4,449	0,980	1,308	M5	18
5,819	4,174	0,979	1,394	M5	9
5,819	4,290	0,973	1,356	M2	6
5,821	4,290	0,976	1,357	M4	4
5,821	4,290	0,984	1,357	M5	25
5,821	4,347	0,985	1,339	M2	13
5,821	4,070	0,965	1,430	M2	5
5,821	4,290	0,968	1,357	M6	10

5,822	4,400	0,985	1,323	M5	17
5,822	4,375	0,982	1,331	M4	3
5,822	4,290	0,972	1,357	M6	4
5,822	4,449	0,985	1,308	M3	9
5,822	4,180	0,985	1,393	M2	6
5,822	4,180	0,973	1,393	M1	17
5,825	4,800	0,986	1,214	M3	7
5,825	4,388	0,967	1,327	M4	9
5,825	4,386	0,978	1,328	M5	4
5,825	4,400	0,992	1,324	M3	13
5,825	4,290	0,972	1,358	M3	7
5,825	4,274	0,985	1,363	M6	3
5,825	4,157	0,960	1,401	M6	3
5,826	4,510	0,998	1,292	M2	5
5,826	4,410	0,990	1,321	M2	18
5,826	4,310	0,979	1,352	M5	15
5,826	4,180	0,958	1,394	M6	7
5,826	4,070	0,964	1,431	M5	14
5,826	4,510	0,984	1,292	M3	7
5,826	4,070	0,967	1,432	M4	11
5,826	4,388	0,996	1,328	M2	26
5,827	4,310	0,987	1,352	M5	20
5,827	4,466	0,967	1,305	M5	17
5,827	4,449	0,988	1,310	M5	15
5,827	4,388	0,981	1,328	M1	8
5,827	4,354	0,977	1,338	M5	17
5,827	4,232	0,984	1,377	M4	22
5,827	4,232	0,971	1,377	M2	5
5,827	4,094	0,959	1,423	M2	7
5,827	4,466	0,978	1,305	M6	7
5,827	4,453	0,982	1,309	M5	13
5,827	4,409	0,986	1,322	M5	4
5,827	3,999	0,978	1,457	M4	5
5,827	3,999	0,963	1,457	M2	2
5,841	4,388	0,981	1,331	M5	19
5,841	4,155	0,980	1,406	M6	16
5,841	4,466	0,982	1,308	M5	6
5,841	4,077	0,947	1,433	M2	8
5,842	4,310	0,977	1,355	M6	4
5,842	4,077	0,981	1,433	M2	7
5,845	4,620	0,991	1,265	M5	22
5,845	4,400	0,984	1,328	M2	26
5,845	4,400	0,965	1,328	M2	8
5,845	4,290	0,970	1,362	M3	4
5,845	4,180	0,972	1,398	M4	20
5,845	4,290	0,980	1,362	M5	23
5,845	4,400	0,978	1,328	M3	4
5,845	4,070	0,959	1,436	M2	13
5,845	3,960	0,951	1,476	M4	7
5,845	4,400	0,990	1,328	M5	22
5,845	4,180	0,950	1,398	M3	7
5,845	4,070	0,962	1,436	M4	4

5,850	4,233	0,975	1,382	M2	5
5,850	4,290	0,967	1,364	M3	8
5,852	4,510	0,984	1,297	M5	16
5,852	4,450	0,973	1,315	M5	17
5,852	4,290	0,989	1,364	M5	8
5,852	4,290	0,971	1,364	M1	6
5,852	4,310	0,991	1,358	M1	6
5,852	4,180	0,970	1,400	M1	7
5,852	4,070	0,957	1,438	M6	8
5,852	4,180	0,967	1,400	M6	2
5,852	4,462	0,990	1,311	M6	6
5,852	4,070	0,969	1,438	M2	7
5,852	4,400	0,993	1,330	M6	7
5,852	4,350	0,986	1,345	M4	5
5,852	4,530	0,962	1,292	M3	11
5,852	4,290	0,968	1,364	M1	13
5,865	4,373	0,990	1,341	M2	10
5,865	4,235	0,994	1,385	M4	7
5,867	4,364	0,963	1,344	M3	8
5,867	4,299	0,970	1,365	M5	10
5,867	4,232	0,949	1,386	M6	6
5,867	4,381	0,975	1,339	M1	11
5,867	4,310	0,976	1,361	M6	25
5,870	4,290	0,983	1,368	M5	16
5,870	4,180	0,977	1,404	M4	7
5,870	4,180	0,960	1,404	M6	4
5,918	4,299	0,968	1,377	M2	8
5,915	4,070	0,955	1,453	M2	10
5,725	4,180	0,983	1,370	M2	6
5,731	4,232	0,979	1,354	M2	7
5,686	4,180	0,979	1,360	M2	8
6,218	4,256	0,956	1,461	M2	4
5,683	4,400	0,992	1,291	M2	6
6,032	4,388	0,970	1,374	M2	7
6,373	4,388	0,959	1,452	M2	6
6,171	4,464	0,965	1,382	M2	7
5,852	4,180	0,994	1,400	M2	4
5,899	4,070	0,965	1,449	M2	5
5,502	4,400	0,993	1,251	M2	15
6,032	4,363	0,981	1,382	M2	5
5,880	4,290	0,976	1,371	M2	7
5,645	3,850	0,972	1,466	M2	9
5,174	3,999	0,977	1,294	M2	14
5,793	4,070	0,966	1,423	M2	4
5,748	4,180	0,983	1,375	M2	6
5,890	4,290	0,988	1,373	M2	6
6,164	4,510	0,970	1,367	M2	6
5,748	4,180	0,979	1,375	M2	7
6,317	4,409	0,964	1,433	M2	5
5,927	4,070	0,963	1,456	M2	7
6,030	3,999	0,956	1,508	M2	4
5,937	4,232	0,973	1,403	M2	5

5,392	4,290	0,984	1,257	M2	3
5,772	4,299	0,977	1,343	M2	8
5,818	4,290	0,983	1,356	M2	2
6,096	4,180	0,953	1,458	M2	7
5,967	4,311	0,953	1,384	M2	10
5,715	4,290	0,979	1,332	M2	14
6,087	4,294	0,962	1,418	M2	5
5,582	4,077	0,981	1,369	M2	6
5,480	4,180	0,992	1,311	M2	5
6,265	4,689	0,966	1,336	M2	4
5,937	4,310	0,963	1,377	M2	6
5,927	4,070	0,960	1,456	M2	6
6,310	4,400	0,955	1,434	M2	6
5,850	4,400	0,933	1,330	M2	11
5,937	4,232	0,973	1,403	M2	4
6,170	4,290	0,955	1,438	M2	5
5,882	4,307	0,971	1,366	M2	3
5,882	4,077	0,957	1,443	M2	7
6,369	4,290	0,960	1,485	M2	3
6,003	4,388	0,963	1,368	M2	8
6,167	4,233	0,959	1,457	M2	6
5,156	3,960	0,996	1,302	M2	6
5,658	4,290	0,972	1,319	M2	9
5,748	4,358	0,991	1,319	M2	3
5,748	4,464	0,992	1,288	M2	12
5,274	3,850	0,986	1,370	M2	13
5,636	4,233	0,989	1,331	M2	4
5,787	4,155	0,963	1,393	M2	6
6,059	4,180	0,969	1,449	M2	7
6,018	4,390	0,939	1,371	M2	9
5,501	3,850	0,974	1,429	M2	5
5,446	4,601	0,975	1,183	M2	7
5,918	4,310	0,968	1,373	M2	10
5,724	3,999	0,972	1,431	M2	3
5,607	4,232	0,979	1,325	M2	8
5,570	4,070	0,992	1,368	M2	5
5,479	4,070	0,993	1,346	M2	8
6,390	3,999	0,908	1,598	M2	6
5,581	4,257	0,986	1,311	M2	2
6,273	5,944	1,000	1,055	M2	5
5,867	4,264	0,984	1,376	M2	7
6,058	4,404	0,967	1,375	M2	6
5,899	4,400	0,989	1,341	M2	12
5,686	3,960	0,969	1,436	M2	5
6,111	4,400	0,959	1,389	M2	6
6,183	4,311	0,977	1,434	M2	11
6,034	4,070	0,947	1,482	M2	7
6,042	4,180	0,955	1,446	M2	8
5,715	4,180	0,970	1,367	M2	6
5,819	4,310	0,980	1,350	M2	7
5,826	4,070	0,967	1,431	M2	7
5,751	4,307	0,978	1,335	M2	5

6,072	4,311	0,933	1,409	M2	7
5,984	4,180	0,961	1,432	M2	6
5,645	4,070	0,971	1,387	M2	5
6,091	4,290	0,966	1,420	M2	6
5,698	4,070	0,977	1,400	M2	6
6,057	4,466	0,972	1,356	M2	4
5,526	4,077	0,978	1,356	M2	6
5,419	3,897	0,980	1,391	M2	11
5,826	4,180	0,977	1,394	M2	15
6,072	4,290	0,940	1,415	M2	3
5,645	4,150	0,972	1,360	M2	5
5,826	4,180	0,978	1,394	M2	9
5,872	4,388	0,982	1,338	M2	14
5,480	4,232	0,993	1,295	M2	4
5,637	3,850	0,972	1,464	M2	3
6,275	4,070	0,938	1,542	M2	5
5,934	3,960	0,952	1,499	M2	6
5,882	4,161	0,946	1,414	M2	8
5,977	4,290	0,963	1,393	M2	12
6,324	4,400	0,953	1,437	M2	5
5,852	4,310	0,974	1,358	M2	5
5,479	4,079	0,984	1,343	M2	4
5,446	4,388	0,986	1,241	M2	6
6,509	4,304	0,945	1,513	M2	3
6,112	4,661	0,984	1,311	M2	4
6,519	4,044	0,917	1,612	M2	5
6,071	4,108	0,953	1,478	M2	7
6,274	4,290	0,956	1,463	M2	11
5,928	4,070	0,956	1,456	M2	7
6,363	4,311	0,956	1,476	M2	4
5,993	4,396	0,975	1,363	M2	6
6,317	4,599	0,979	1,373	M2	7
6,169	4,070	0,945	1,516	M2	8
5,871	4,400	0,969	1,334	M2	9
6,363	4,463	0,961	1,426	M2	11
6,117	4,730	0,971	1,293	M2	12
6,273	4,155	0,940	1,510	M2	17
6,363	4,311	0,956	1,476	M2	4
5,993	4,396	0,975	1,363	M2	6
6,317	4,599	0,979	1,373	M2	7
6,169	4,070	0,945	1,516	M2	8
5,871	4,400	0,969	1,334	M2	9
6,363	4,463	0,961	1,426	M2	11
6,273	4,155	0,940	1,510	M2	18
6,091	4,037	0,950	1,509	M2	8
6,162	4,354	0,966	1,415	M2	13
6,134	4,113	0,960	1,491	M2	14
6,523	4,068	0,920	1,603	M2	17
6,602	4,639	0,931	1,423	M2	18
6,091	4,037	0,950	1,509	M2	8
6,162	4,354	0,966	1,415	M2	13
6,134	4,113	0,960	1,491	M2	14

6,523	4,068	0,920	1,603	M2	17
6,277	4,273	0,962	1,469	M2	7
6,207	4,330	0,968	1,434	M2	8
5,687	4,070	0,975	1,397	M2	5
6,519	4,510	0,969	1,445	M2	13
5,818	4,284	0,966	1,358	M2	3
5,520	4,123	0,990	1,339	M2	6
5,621	4,077	0,973	1,379	M2	7
5,822	4,290	0,983	1,357	M2	8
5,852	4,254	0,972	1,376	M2	10
5,814	4,155	0,953	1,399	M2	12
5,977	4,297	0,968	1,391	M2	5
5,956	4,290	0,972	1,388	M2	7
5,580	4,070	0,981	1,371	M2	12
5,736	4,070	0,970	1,409	M2	2
5,673	4,400	0,976	1,289	M2	4
6,358	4,248	0,942	1,497	M2	5
5,772	4,180	0,961	1,381	M2	6
5,504	4,067	0,980	1,353	M2	10
5,472	4,077	0,966	1,342	M2	12
5,715	4,180	0,974	1,367	M2	13
5,523	4,070	0,978	1,357	M2	16
5,264	3,844	0,978	1,370	M2	4
5,852	4,290	0,979	1,364	M2	8
5,571	4,077	0,973	1,366	M2	2
5,731	4,077	0,968	1,406	M2	7
5,715	4,070	0,984	1,404	M2	8
5,570	4,180	0,983	1,333	M2	9
6,018	4,070	0,954	1,479	M2	4
5,429	3,960	0,980	1,371	M2	5
5,764	3,960	0,964	1,456	M2	6
5,772	4,469	0,975	1,291	M2	9
5,609	4,180	0,979	1,342	M2	11
5,724	4,388	0,976	1,304	M2	4
5,826	4,070	0,970	1,431	M2	6
6,031	4,950	0,947	1,218	M2	3
5,580	4,070	0,980	1,371	M2	5
6,303	4,375	0,944	1,441	M2	12
5,580	4,070	0,980	1,371	M2	7
6,303	4,375	0,944	1,441	M2	14
5,634	3,999	0,967	1,409	M2	5
5,821	4,180	0,972	1,393	M2	7
5,697	4,311	0,988	1,322	M2	2
5,501	4,070	0,961	1,352	M2	4
5,368	4,180	0,979	1,284	M2	6
5,672	4,252	0,993	1,334	M2	7
5,825	4,397	0,989	1,325	M2	9
5,637	4,290	0,979	1,314	M2	11
5,590	4,070	0,964	1,374	M2	15
5,871	4,310	0,985	1,362	M2	17
5,764	3,740	0,945	1,541	M2	1
6,030	4,417	0,976	1,365	M2	4

5,850	4,232	0,967	1,382	M2	6
5,765	4,214	0,975	1,368	M2	8
5,520	4,095	0,970	1,348	M2	9
5,793	4,070	0,957	1,423	M2	10
5,526	4,290	0,984	1,288	M2	11
5,937	4,388	0,981	1,353	M2	12
5,743	3,960	0,970	1,450	M2	14
5,993	4,400	0,975	1,362	M2	20
6,030	4,417	0,976	1,365	M2	5
5,850	4,232	0,967	1,382	M2	7
5,765	4,214	0,975	1,368	M2	9
5,520	4,095	0,970	1,348	M2	10
5,793	4,070	0,957	1,423	M2	11
5,526	4,290	0,984	1,288	M2	12
5,937	4,388	0,981	1,353	M2	13
5,743	3,960	0,970	1,450	M2	15
5,993	4,400	0,975	1,362	M2	21
5,645	3,994	0,966	1,413	M2	3
5,725	4,466	0,999	1,282	M2	4
6,168	4,180	0,945	1,476	M2	7
5,965	4,226	0,971	1,411	M2	9
5,776	4,180	0,985	1,382	M2	10
5,937	4,310	0,967	1,377	M2	12
5,673	4,048	0,971	1,402	M2	2
5,621	4,345	0,991	1,294	M2	3
5,786	4,070	0,976	1,422	M2	6
5,661	4,297	0,981	1,317	M2	7
5,814	4,388	0,984	1,325	M2	9
5,691	4,155	0,957	1,370	M2	10
5,995	4,388	0,967	1,366	M2	11
5,446	4,070	0,975	1,338	M2	13
5,971	4,496	0,977	1,328	M2	14
5,692	4,310	0,986	1,321	M2	15
5,786	4,388	0,975	1,319	M2	17
5,918	4,233	0,974	1,398	M2	21
5,590	4,070	0,966	1,373	M2	6
5,880	4,510	0,988	1,304	M2	9
5,825	4,363	0,983	1,335	M2	12
5,881	4,077	0,959	1,443	M2	13
5,486	4,155	0,985	1,320	M2	19
5,719	4,449	0,987	1,286	M2	4
5,446	4,070	0,963	1,338	M2	6
5,580	4,180	0,983	1,335	M2	9
6,607	4,777	0,946	1,383	M2	6
6,459	4,510	0,924	1,432	M2	7
6,537	4,290	0,935	1,524	M2	9
5,749	4,290	0,960	1,340	M2	10
6,127	4,510	0,951	1,359	M2	14
6,246	4,840	0,970	1,290	M2	16
6,128	4,077	0,920	1,503	M2	18
5,904	4,077	0,946	1,448	M2	5
5,972	4,660	0,983	1,281	M2	7

5,744	4,180	0,967	1,374	M2	8
6,171	4,702	0,960	1,312	M2	9
5,772	4,622	1,000	1,249	M2	7
5,827	4,232	0,983	1,377	M2	10
7,189	4,777	0,905	1,505	M2	11
5,867	4,582	0,972	1,280	M2	13
6,247	4,388	0,927	1,424	M2	1
5,673	4,290	0,977	1,322	M2	5
6,167	4,466	0,970	1,381	M2	8
5,786	4,155	0,976	1,393	M2	11
5,619	4,095	0,974	1,372	M2	12
5,772	4,125	0,962	1,399	M2	16
5,765	4,290	0,994	1,344	M2	5
5,940	4,155	0,959	1,430	M2	7
5,581	4,058	0,959	1,375	M2	8
5,777	4,290	0,977	1,347	M2	9
5,683	4,290	0,994	1,325	M2	16
5,934	4,180	0,956	1,420	M2	5
5,927	4,449	0,996	1,332	M2	7
5,918	4,232	0,960	1,398	M2	10
5,570	4,290	0,997	1,298	M2	12
5,610	4,400	0,979	1,275	M2	13
5,580	4,070	0,985	1,371	M2	14
5,504	4,180	0,974	1,317	M2	16
5,134	3,850	1,000	1,333	M2	17
5,736	4,180	0,982	1,372	M2	20
5,736	4,290	0,972	1,337	M2	4
5,772	4,388	0,997	1,315	M2	8
6,197	4,437	0,935	1,397	M2	9
5,771	4,077	0,991	1,415	M2	11
5,993	4,290	0,981	1,397	M2	13
6,121	4,388	0,975	1,395	M2	7
6,274	4,388	0,962	1,430	M2	5
5,880	4,400	0,995	1,336	M2	9
6,167	4,388	0,970	1,406	M2	3
6,157	4,388	0,958	1,403	M2	6
5,927	4,388	0,987	1,351	M2	8
6,058	4,310	0,959	1,405	M2	9
6,348	4,400	0,952	1,443	M2	13
5,751	4,310	0,980	1,334	M2	12
5,899	4,290	0,979	1,375	M2	7
5,719	4,232	0,983	1,351	M2	8
5,927	4,180	0,970	1,418	M2	4
5,882	4,310	0,971	1,365	M2	5
5,956	4,620	0,966	1,289	M2	8
5,501	4,070	0,980	1,352	M2	9
5,607	4,070	0,989	1,378	M2	10
6,112	4,510	0,928	1,355	M2	11
5,827	4,369	0,984	1,334	M2	12
6,118	4,257	0,974	1,437	M2	13
5,870	4,400	0,979	1,334	M2	20
5,927	4,180	0,970	1,418	M2	4

5,882	4,310	0,971	1,365	M2	5
5,956	4,620	0,966	1,289	M2	8
5,501	4,070	0,980	1,352	M2	9
5,607	4,070	0,989	1,378	M2	10
5,827	4,369	0,984	1,334	M2	13
6,118	4,257	0,974	1,437	M2	14
5,870	4,400	0,979	1,334	M2	21
6,057	4,383	0,971	1,382	M2	4
5,904	4,233	0,964	1,395	M2	7
5,717	4,070	0,970	1,405	M2	8
5,963	4,204	0,941	1,418	M2	8
5,764	4,070	0,966	1,416	M2	10
5,638	3,850	0,962	1,464	M2	3
5,626	4,290	0,999	1,311	M2	5
5,967	4,232	0,953	1,410	M2	9
5,265	4,070	0,975	1,294	M2	10
5,771	4,070	0,959	1,418	M2	11
6,030	4,070	0,970	1,482	M2	12
5,532	4,070	0,981	1,359	M2	13
5,764	3,960	0,976	1,456	M2	19
5,637	4,180	0,971	1,349	M2	3
5,480	4,070	0,996	1,346	M2	5
5,927	3,850	0,946	1,540	M2	7
5,697	4,077	0,959	1,397	M2	8
6,011	4,237	0,943	1,419	M2	10
5,233	3,850	0,984	1,359	M2	12
5,751	3,922	0,962	1,466	M2	13
5,772	4,148	0,962	1,391	M2	16
6,057	4,326	0,946	1,400	M2	23
5,626	4,048	0,971	1,390	M2	8
5,570	3,960	0,958	1,407	M2	5
6,032	4,232	0,948	1,425	M2	6
5,971	4,204	0,964	1,420	M2	9
5,391	3,999	0,975	1,348	M2	13
5,580	3,960	0,973	1,409	M2	4
5,928	4,290	0,982	1,382	M2	6
5,626	4,077	0,975	1,380	M2	8
5,993	4,269	0,980	1,404	M2	10
5,519	4,170	0,991	1,323	M2	11
5,204	3,850	0,977	1,352	M2	12
5,692	4,155	0,985	1,370	M2	14
5,473	3,844	0,980	1,424	M2	8
6,017	4,097	0,953	1,469	M2	9
5,818	4,179	0,975	1,392	M2	11
5,661	4,077	0,970	1,389	M2	12
5,570	4,070	0,982	1,368	M2	7
5,552	4,070	0,980	1,364	M2	9
5,619	4,138	0,983	1,358	M2	11
5,718	3,850	0,951	1,485	M2	13
5,501	4,070	0,975	1,352	M2	14
5,711	4,070	0,976	1,403	M2	16
6,018	4,350	0,975	1,383	M2	17

6,094	4,310	0,966	1,414	M2	22
5,965	4,290	0,977	1,391	M2	4
5,724	4,155	0,972	1,378	M2	8
5,760	4,155	0,977	1,386	M2	10
5,581	4,070	0,981	1,371	M2	11
5,872	4,277	0,979	1,373	M2	4
5,760	4,155	0,974	1,386	M2	6
5,486	4,077	0,980	1,345	M2	8
5,581	4,070	0,977	1,371	M2	9
5,922	4,232	0,969	1,399	M2	11
5,645	4,327	0,976	1,305	M2	5
5,581	3,960	0,978	1,409	M2	6
5,233	3,960	0,990	1,322	M2	7
5,821	3,740	0,928	1,556	M2	10
5,993	4,390	0,974	1,365	M2	4
5,786	4,290	0,989	1,349	M2	8
5,736	4,070	0,955	1,409	M2	9
6,247	4,388	0,952	1,424	M2	14
5,956	4,400	0,981	1,354	M2	8
6,168	4,163	0,913	1,482	M2	9
5,724	4,679	0,956	1,223	M2	13
5,956	4,400	0,981	1,354	M2	8
5,474	4,163	0,934	1,315	M2	9
5,724	3,843	0,961	1,489	M2	15
5,446	4,077	0,973	1,336	M2	3
6,140	4,290	0,972	1,431	M2	10
5,787	4,077	0,970	1,420	M2	3
5,637	4,180	0,979	1,349	M2	5
6,058	4,388	0,978	1,381	M2	6
5,850	4,233	0,969	1,382	M2	7
5,715	4,180	0,987	1,367	M2	8
5,607	4,070	0,990	1,378	M2	11
5,609	4,070	0,974	1,378	M2	12
5,904	4,311	0,980	1,370	M2	16
5,438	4,070	0,979	1,336	M2	18
5,872	4,345	0,987	1,351	M2	19
5,598	4,232	0,982	1,323	M2	10
5,683	4,180	0,983	1,359	M2	3
5,503	3,630	0,902	1,516	M2	4
5,438	3,960	0,980	1,373	M2	5
5,661	4,233	0,977	1,337	M2	8
5,683	4,180	0,983	1,359	M2	3
5,438	3,960	0,980	1,373	M2	5
5,661	4,233	0,977	1,337	M2	9
5,609	4,070	0,971	1,378	M2	5
5,744	4,180	0,970	1,374	M2	7
5,962	4,544	0,977	1,312	M2	9
5,777	4,269	0,973	1,353	M2	13
5,918	4,483	0,904	1,320	M2	18
5,502	3,960	0,981	1,389	M2	3
5,552	4,070	0,976	1,364	M2	5
6,157	4,621	0,974	1,332	M2	8

5,626	4,264	0,993	1,319	M2	11
5,880	4,396	0,986	1,338	M2	10
5,637	3,960	0,956	1,424	M2	5
5,526	4,310	0,999	1,282	M2	7
5,526	3,960	0,980	1,396	M2	8
5,438	4,109	0,991	1,323	M2	5
5,619	4,077	0,987	1,378	M2	8
5,724	4,155	0,979	1,378	M2	9
5,772	4,197	0,963	1,375	M2	10
5,222	3,901	0,983	1,339	M2	6
5,736	4,070	0,969	1,409	M2	8
5,786	3,860	0,932	1,499	M2	9
5,503	4,180	0,972	1,316	M2	10
5,814	4,290	0,991	1,355	M2	15
5,626	4,170	0,982	1,349	M2	19
5,826	4,290	0,958	1,358	M2	7
5,445	3,850	0,957	1,414	M2	8
6,332	5,500	0,974	1,151	M2	9
5,473	3,999	0,986	1,368	M2	12
5,571	3,921	0,968	1,421	M2	14
5,852	4,180	0,975	1,400	M2	15
5,502	3,960	0,987	1,389	M2	17
5,504	4,262	0,983	1,291	M2	21
5,807	4,702	0,917	1,235	M2	23
5,826	4,290	0,958	1,358	M2	7
5,445	3,850	0,957	1,414	M2	8
5,473	3,999	0,986	1,368	M2	13
5,571	3,921	0,968	1,421	M2	15
5,852	4,180	0,975	1,400	M2	16
5,502	3,960	0,987	1,389	M2	18
5,504	4,262	0,983	1,291	M2	22
5,523	4,070	0,974	1,357	M2	8
5,552	3,850	0,951	1,442	M2	9
6,246	5,500	0,946	1,136	M2	10
5,566	3,922	0,992	1,419	M2	14
5,676	4,388	0,981	1,294	M2	15
5,626	4,300	0,978	1,308	M2	20
5,788	4,617	0,925	1,254	M2	22
5,523	4,070	0,974	1,357	M2	8
5,552	3,850	0,951	1,442	M2	9
5,566	3,922	0,992	1,419	M2	15
5,676	4,388	0,981	1,294	M2	16
5,626	4,300	0,978	1,308	M2	21
5,619	4,070	0,986	1,381	M2	6
5,445	3,960	0,983	1,375	M2	7
6,164	4,231	0,965	1,457	M2	8
5,812	4,228	0,970	1,375	M2	10
5,581	3,960	0,969	1,409	M2	14
5,544	4,077	0,971	1,360	M2	16
5,772	4,290	0,979	1,345	M2	19
5,845	4,400	0,993	1,328	M2	3
5,918	4,165	0,947	1,421	M2	4

5,571	3,999	0,974	1,393	M2	6
5,918	4,190	0,934	1,412	M2	8
5,467	3,960	0,984	1,381	M2	10
5,572	4,097	0,980	1,360	M2	11
5,645	3,960	0,978	1,426	M2	12
5,519	4,077	0,976	1,354	M2	13
5,552	3,850	0,973	1,442	M2	15
5,717	3,960	0,950	1,444	M2	16
5,870	4,180	0,966	1,404	M4	4
5,870	4,180	0,948	1,404	M1	6
5,870	4,070	0,973	1,442	M3	8
5,870	4,400	0,961	1,334	M6	29
5,870	4,180	0,956	1,404	M5	5
5,871	4,290	0,972	1,369	M5	15
5,871	4,290	0,972	1,369	M3	8
5,871	4,180	0,960	1,405	M3	4
5,871	4,180	0,973	1,405	M5	13
5,871	4,070	0,971	1,442	M4	7
5,871	4,496	0,991	1,306	M5	7
5,871	4,423	0,982	1,327	M5	14
5,871	4,326	0,983	1,357	M4	3
5,871	4,211	0,981	1,394	M2	7
5,871	4,135	0,959	1,420	M6	16
5,872	4,290	0,977	1,369	M6	4
5,872	4,357	0,986	1,347	M2	4
5,872	4,292	0,978	1,368	M4	3
5,877	4,444	0,979	1,322	M3	10
5,877	4,233	0,968	1,388	M2	6
5,877	4,233	0,974	1,388	M3	4
5,877	4,155	0,962	1,415	M3	5
5,880	4,290	0,985	1,371	M3	10
5,880	4,070	0,968	1,445	M6	8
5,880	4,070	0,960	1,445	M3	14
5,880	4,510	0,996	1,304	M5	8
5,880	4,400	0,967	1,336	M4	2
5,880	4,400	0,993	1,336	M3	10
5,880	4,277	0,981	1,375	M5	7
5,882	4,466	0,979	1,317	M3	8
5,882	4,210	0,982	1,397	M4	10
5,882	4,077	0,964	1,443	M3	6
5,882	4,299	0,973	1,368	M6	6
5,882	4,217	0,974	1,395	M4	10
5,882	4,155	0,963	1,416	M3	4
5,889	4,639	0,997	1,269	M5	13
5,889	4,544	0,981	1,296	M2	14
5,889	4,425	0,980	1,331	M5	11
5,889	4,290	0,980	1,373	M5	13
5,889	4,244	0,978	1,388	M4	4
5,889	4,330	0,980	1,360	M1	5
5,889	4,290	0,965	1,373	M2	3
5,889	4,156	0,967	1,417	M2	4
5,890	4,510	0,984	1,306	M3	8

5,890	4,195	0,975	1,404	M2	12
5,890	4,179	0,975	1,409	M4	10
5,896	4,290	0,961	1,374	M1	11
5,898	4,622	0,973	1,276	M5	41
5,898	4,232	0,958	1,394	M5	8
5,899	4,457	0,981	1,324	M1	7
5,899	4,400	0,983	1,341	M2	3
5,899	4,290	0,972	1,375	M5	5
5,899	4,290	0,975	1,375	M3	18
5,899	4,290	0,981	1,375	M2	12
5,899	4,290	0,963	1,375	M1	9
5,899	4,290	0,970	1,375	M5	8
5,899	4,290	0,951	1,375	M6	12
5,899	4,180	0,972	1,411	M1	7
5,899	4,290	0,978	1,375	M5	24
5,899	4,290	0,978	1,375	M5	26
5,899	4,180	0,977	1,411	M4	18
5,899	3,960	0,955	1,490	M6	26
5,899	4,510	0,987	1,308	M3	7
5,899	4,290	0,975	1,375	M3	9
5,899	4,180	0,974	1,411	M4	13
5,899	4,180	0,981	1,411	M4	16
5,899	4,180	0,926	1,411	M2	5
5,904	4,699	0,963	1,256	M3	5
5,904	4,388	0,955	1,345	M5	16
5,904	4,311	0,955	1,370	M3	3
5,904	4,155	0,968	1,421	M4	4
5,904	3,999	0,946	1,476	M2	6
5,904	4,220	0,967	1,399	M4	7
5,904	4,155	0,973	1,421	M2	6
5,911	4,699	0,983	1,258	M5	11
5,911	4,188	0,954	1,411	M6	11
5,911	4,489	0,978	1,317	M1	3
5,913	4,466	0,960	1,324	M3	10
5,913	4,311	0,971	1,372	M3	9
5,913	4,388	0,951	1,347	M3	6
5,913	4,077	0,956	1,450	M4	2
5,913	4,459	0,962	1,326	M1	4
5,913	4,155	0,975	1,423	M4	7
5,915	3,960	0,958	1,494	M4	10
5,915	4,289	0,954	1,379	M2	29
5,915	4,289	0,954	1,379	M2	33
5,917	4,510	0,978	1,312	M3	14
5,917	4,354	0,987	1,359	M2	7
5,917	3,960	0,957	1,494	M2	4
5,918	4,379	0,965	1,351	M2	16
5,918	4,180	0,975	1,416	M5	6
5,918	4,290	0,970	1,379	M3	6
5,918	4,180	0,983	1,416	M4	16
5,918	4,466	0,989	1,325	M5	15
5,918	4,350	0,982	1,360	M5	8
5,918	4,397	0,983	1,346	M3	14

5,918	4,310	0,964	1,373	M6	19
5,918	4,169	0,983	1,419	M4	5
5,918	4,510	0,975	1,312	M3	14
5,918	4,350	0,958	1,361	M4	15
5,918	4,303	0,967	1,376	M4	26
5,922	4,449	0,978	1,331	M6	7
5,922	4,381	0,982	1,352	M5	11
5,922	4,576	0,949	1,294	M2	11
5,922	4,433	0,980	1,336	M4	7
5,922	4,308	0,970	1,375	M6	4
5,922	4,155	0,973	1,425	M3	13
5,927	4,450	0,979	1,332	M5	7
5,927	4,343	0,978	1,365	M4	6
5,927	4,290	0,965	1,382	M5	17
5,927	4,388	0,991	1,351	M1	8
5,927	4,330	0,972	1,369	M2	3
5,927	4,414	0,989	1,343	M1	6
5,927	4,400	0,989	1,347	M1	9
5,927	4,290	0,964	1,382	M3	9
5,927	4,150	0,969	1,428	M2	5
5,927	4,170	0,972	1,422	M6	17
5,927	4,290	0,985	1,382	M6	5
5,927	4,400	0,977	1,347	M6	10
5,927	4,290	0,973	1,382	M1	10
5,927	4,264	0,964	1,390	M2	13
5,927	4,290	0,974	1,382	M1	17
5,927	4,290	0,983	1,382	M4	8
5,927	4,510	0,976	1,314	M3	15
5,928	4,400	0,985	1,347	M5	6
5,928	4,290	0,970	1,382	M4	9
5,934	4,290	0,981	1,383	M2	10
5,934	4,400	0,961	1,349	M4	3
5,934	4,290	0,962	1,383	M3	15
5,935	4,400	0,971	1,349	M2	16
5,935	4,400	0,985	1,349	M5	8
5,935	4,400	0,982	1,349	M2	26
5,935	4,290	0,967	1,383	M3	6
5,607	3,803	0,958	1,475	M2	3
5,542	4,232	0,993	1,309	M2	6
5,827	4,077	0,969	1,429	M2	7
6,563	4,499	0,957	1,459	M2	15
5,814	4,233	0,971	1,374	M2	6
6,247	3,921	0,922	1,593	M2	6
6,071	3,946	0,939	1,539	M2	11
5,607	3,813	0,944	1,471	M2	4
5,881	4,155	0,977	1,415	M2	5
5,814	4,400	0,991	1,321	M2	6
6,057	4,155	0,966	1,458	M2	7
5,711	3,850	0,966	1,483	M2	8
5,827	3,922	0,948	1,486	M2	4
5,889	4,142	0,949	1,422	M2	5
5,719	4,044	0,974	1,414	M2	7

5,814	4,077	0,970	1,426	M2	4
5,777	3,960	0,960	1,459	M2	3
5,290	3,850	0,980	1,374	M2	6
5,645	3,960	0,970	1,426	M2	8
5,609	4,180	0,980	1,342	M2	3
5,523	3,740	0,967	1,477	M2	6
5,373	4,274	1,000	1,257	M2	7
5,544	3,999	0,986	1,386	M2	8
5,517	3,922	0,965	1,407	M2	9
5,731	4,077	0,985	1,406	M2	4
5,787	4,077	0,962	1,419	M2	7
5,391	3,766	0,965	1,431	M2	8
5,826	4,198	0,973	1,388	M2	10
5,736	3,960	0,967	1,449	M2	12
5,645	3,960	0,978	1,426	M2	13
5,715	4,290	0,981	1,332	M2	18
5,658	4,180	0,980	1,354	M2	5
5,673	4,070	0,983	1,394	M2	7
5,760	3,921	0,960	1,469	M2	9
5,661	4,077	0,973	1,389	M2	5
5,581	3,843	0,954	1,452	M2	5
5,732	4,075	0,973	1,407	M2	7
5,438	3,916	0,979	1,389	M2	9
5,607	4,064	0,968	1,380	M2	4
5,732	3,921	0,974	1,462	M2	6
5,420	3,921	0,968	1,382	M2	7
5,544	4,077	0,967	1,360	M2	5
5,715	3,960	0,966	1,443	M2	5
5,793	4,070	0,970	1,423	M2	3
5,474	3,960	0,980	1,382	M2	5
5,827	3,921	0,969	1,486	M2	8
5,737	4,070	0,962	1,409	M2	2
5,363	4,045	0,991	1,326	M2	5
5,339	4,180	0,979	1,277	M2	11
5,717	4,070	0,972	1,405	M2	3
5,867	4,178	0,970	1,404	M2	12
5,936	4,155	0,983	1,429	M2	5
5,572	4,097	0,988	1,360	M2	7
5,927	4,510	0,997	1,314	M2	8
5,786	4,249	0,983	1,362	M2	10
6,005	4,070	0,963	1,475	M2	6
5,748	4,180	0,981	1,375	M2	4
5,609	3,850	0,952	1,457	M2	6
5,737	4,070	0,970	1,409	M2	7
5,765	4,057	0,975	1,421	M2	8
5,812	4,147	0,974	1,401	M2	13
5,787	4,077	0,961	1,420	M2	4
5,731	4,077	0,964	1,406	M2	7
5,771	3,844	0,951	1,501	M2	5
5,532	3,740	0,960	1,479	M2	9
5,880	4,290	0,967	1,371	M2	12
5,526	4,232	0,989	1,306	M2	4

5,503	3,850	0,973	1,429	M2	8
5,825	4,111	0,969	1,417	M2	3
5,672	3,922	0,962	1,446	M2	7
5,765	3,921	0,974	1,470	M2	10
5,956	4,142	0,955	1,438	M2	16
5,580	4,131	0,982	1,351	M2	4
5,748	4,291	0,968	1,339	M2	11
6,428	4,270	0,912	1,505	M2	8
5,719	4,311	0,983	1,327	M2	13
5,658	3,960	0,971	1,429	M2	14
5,504	4,070	0,992	1,352	M2	6
6,096	4,383	0,957	1,391	M2	7
5,842	4,310	0,971	1,355	M2	11
5,736	3,960	0,973	1,449	M2	5
5,672	3,674	0,940	1,544	M2	7
5,634	3,999	0,963	1,409	M2	8
5,940	4,077	0,954	1,457	M2	7
5,580	4,180	0,985	1,335	M2	11
5,852	4,278	0,972	1,368	M2	7
5,793	4,070	0,979	1,423	M2	12
5,523	3,960	0,976	1,395	M2	3
5,621	4,077	0,982	1,379	M2	6
5,967	3,999	0,960	1,492	M2	7
5,683	3,960	0,964	1,435	M2	9
5,751	4,155	0,970	1,384	M2	4
5,439	4,232	0,986	1,285	M2	6
5,502	3,850	0,978	1,429	M2	4
5,719	4,016	0,969	1,424	M2	8
5,481	3,766	0,972	1,455	M2	4
5,544	4,155	0,980	1,334	M2	9
5,686	4,163	0,973	1,366	M2	6
5,445	3,850	0,967	1,414	M2	7
6,264	4,136	0,922	1,514	M2	3
6,072	4,104	0,943	1,479	M2	5
6,018	3,850	0,936	1,563	M2	7
5,927	4,143	0,955	1,431	M2	12
5,827	3,999	0,956	1,457	M2	7
5,760	4,155	0,961	1,386	M2	3
6,310	4,219	0,937	1,496	M2	7
5,453	3,922	0,970	1,391	M2	9
5,751	4,077	0,956	1,410	M2	6
5,526	4,000	0,976	1,382	M2	12
5,607	4,077	0,955	1,375	M2	7
5,431	4,070	0,982	1,334	M2	6
5,396	4,070	0,977	1,326	M2	6
5,772	4,155	0,976	1,389	M2	5
5,417	4,180	0,991	1,296	M2	7
6,151	4,070	0,949	1,511	M2	8
6,140	4,180	0,968	1,469	M2	9
6,091	4,070	0,954	1,496	M2	11
6,148	3,997	0,945	1,538	M2	5
5,956	4,400	0,978	1,354	M2	10

6,331	4,155	0,944	1,524	M2	14
6,031	3,960	0,948	1,523	M2	5
5,607	4,141	0,986	1,354	M2	4
6,003	4,077	0,963	1,473	M2	5
5,917	4,180	0,970	1,416	M2	6
6,151	4,070	0,957	1,511	M2	9
6,018	4,016	0,942	1,498	M2	10
6,531	4,388	0,940	1,488	M2	6
6,112	4,009	0,949	1,525	M2	3
6,182	4,264	0,952	1,450	M2	4
6,368	4,567	0,974	1,394	M2	8
5,918	3,960	0,929	1,494	M2	3
6,171	3,926	0,919	1,572	M2	5
5,967	3,843	0,946	1,553	M2	6
6,147	3,999	0,947	1,537	M2	9
5,850	3,999	0,958	1,463	M2	5
5,918	3,880	0,960	1,525	M2	8
6,071	4,085	0,958	1,486	M2	5
6,053	4,310	0,955	1,404	M2	13
5,765	3,850	0,944	1,497	M2	3
6,006	4,070	0,960	1,476	M2	5
6,463	4,416	0,967	1,464	M2	7
6,164	4,304	0,968	1,432	M2	11
6,190	4,400	0,967	1,407	M2	15
6,809	4,855	0,960	1,403	M2	4
6,410	4,179	0,940	1,534	M2	6
5,904	3,999	0,952	1,476	M2	12
6,410	4,179	0,940	1,534	M2	7
5,904	3,999	0,952	1,476	M2	13
6,083	4,180	0,952	1,455	M2	5
5,743	3,850	0,973	1,492	M2	8
5,889	4,180	0,963	1,409	M2	4
5,994	4,077	0,962	1,470	M2	7
6,030	3,999	0,953	1,508	M2	4
6,030	4,160	0,965	1,449	M2	6
6,222	4,232	0,955	1,470	M2	11
6,017	4,311	0,948	1,396	M2	10
6,274	3,850	0,910	1,630	M2	11
5,715	3,960	0,930	1,443	M2	6
6,167	4,077	0,950	1,513	M2	4
5,904	3,922	0,948	1,505	M2	6
5,772	4,155	0,954	1,389	M2	5
6,064	4,351	0,970	1,394	M2	7
6,021	4,070	0,960	1,479	M2	9
5,807	4,070	0,955	1,427	M2	13
6,059	4,290	0,970	1,412	M2	3
5,872	4,220	0,969	1,392	M2	7
5,749	4,180	0,982	1,375	M2	8
6,042	4,070	0,938	1,485	M2	14
6,058	4,134	0,955	1,466	M2	7
5,871	4,343	0,968	1,352	M2	3
5,922	4,123	0,960	1,436	M2	6

5,818	4,197	0,972	1,386	M2	8
5,777	4,122	0,956	1,401	M2	5
5,683	4,290	0,990	1,325	M2	7
5,825	4,163	0,970	1,399	M2	8
6,011	4,354	0,976	1,381	M2	6
5,821	3,850	0,955	1,512	M2	7
5,814	4,180	0,970	1,391	M2	4
5,922	4,147	0,965	1,428	M2	6
5,971	4,036	0,939	1,479	M2	7
6,403	4,254	0,932	1,505	M2	6
6,062	3,960	0,957	1,531	M2	13
6,018	4,310	0,967	1,396	M2	11
5,711	3,943	0,958	1,448	M2	5
6,553	4,388	0,949	1,493	M2	7
5,814	3,999	0,967	1,454	M2	9
6,274	4,232	0,948	1,482	M2	3
6,168	4,186	0,920	1,474	M2	5
5,572	3,911	0,973	1,425	M2	3
5,793	3,850	0,946	1,505	M2	8
6,064	4,152	0,964	1,461	M2	13
5,977	4,180	0,945	1,430	M2	3
5,870	3,740	0,919	1,570	M2	5
5,899	3,850	0,967	1,532	M2	8
6,018	4,070	0,946	1,479	M2	11
5,724	3,999	0,959	1,431	M2	14
5,429	3,740	0,970	1,452	M2	4
6,184	4,077	0,945	1,517	M2	5
6,003	4,077	0,946	1,472	M2	7
6,032	4,155	0,948	1,452	M2	7
5,850	3,999	0,943	1,463	M2	4
5,865	3,844	0,940	1,526	M2	7
5,841	4,077	0,958	1,433	M2	5
5,967	3,843	0,934	1,553	M2	8
5,777	3,850	0,970	1,500	M2	9
6,087	3,999	0,945	1,522	M2	9
5,686	3,960	0,962	1,436	M2	12
5,786	4,290	0,971	1,349	M2	6
5,937	3,766	0,933	1,576	M2	10
5,918	4,070	0,959	1,454	M2	5
6,058	4,077	0,960	1,486	M2	3
6,162	4,180	0,960	1,474	M2	6
5,967	4,155	0,976	1,436	M2	10
6,011	3,843	0,918	1,564	M2	11
5,871	4,255	0,968	1,380	M2	3
6,264	4,411	0,963	1,420	M2	13
5,571	3,999	0,968	1,393	M2	5
6,096	4,236	0,960	1,439	M2	8
5,814	4,077	0,967	1,426	M2	10
5,871	4,000	0,966	1,468	M2	11
6,017	4,232	0,958	1,422	M2	3
5,748	4,104	0,971	1,401	M2	6
6,064	4,068	0,946	1,491	M2	7

6,217	4,400	0,971	1,413	M2	8
5,818	4,075	0,960	1,428	M2	10
6,094	4,449	0,935	1,370	M2	13
5,889	4,058	0,955	1,451	M2	6
5,915	4,180	0,963	1,415	M2	8
6,134	4,318	0,964	1,420	M2	10
5,645	4,085	0,976	1,382	M2	4
6,021	4,255	0,973	1,415	M2	6
6,083	5,060	0,960	1,202	M2	8
5,731	4,160	0,976	1,378	M2	10
5,880	4,198	0,958	1,401	M2	11
5,922	4,423	0,981	1,339	M2	17
5,645	4,085	0,976	1,382	M2	4
6,021	4,255	0,973	1,415	M2	6
5,731	4,160	0,976	1,378	M2	11
5,880	4,198	0,958	1,401	M2	12
5,922	4,423	0,981	1,339	M2	18
6,021	4,180	0,965	1,440	M2	3
5,962	4,077	0,975	1,462	M2	8
6,264	4,381	0,933	1,430	M2	9
6,391	4,134	0,938	1,546	M2	11
6,021	4,180	0,965	1,440	M2	3
5,962	4,077	0,975	1,462	M2	8
6,391	4,134	0,938	1,546	M2	12
5,677	4,075	0,971	1,393	M2	7
5,936	4,388	0,977	1,353	M4	7
5,936	4,388	0,976	1,353	M6	6
5,936	4,544	0,981	1,306	M5	5
5,936	4,311	0,980	1,377	M3	2
5,936	4,233	0,967	1,402	M5	4
5,936	4,311	0,977	1,377	M3	5
5,936	4,155	0,964	1,429	M1	11
5,937	4,533	0,996	1,310	M4	8
5,937	4,466	0,969	1,329	M2	13
5,937	4,294	0,956	1,383	M4	13
5,937	4,291	0,962	1,383	M5	10
5,937	4,232	0,963	1,403	M3	21
5,937	4,423	0,988	1,342	M5	11
5,937	4,544	0,981	1,307	M6	9
5,937	4,388	0,976	1,353	M2	26
5,937	4,310	0,980	1,377	M2	27
5,937	4,232	0,968	1,403	M3	2
5,937	4,155	0,964	1,429	M5	3
5,940	4,614	0,968	1,287	M3	9
5,940	4,388	0,969	1,354	M2	9
5,940	4,233	0,974	1,403	M3	7
5,940	4,233	0,978	1,403	M4	3
5,940	4,310	0,973	1,378	M2	7
5,940	4,310	0,973	1,378	M2	8
5,940	4,310	0,963	1,378	M3	8
5,940	4,232	0,972	1,404	M2	17
5,940	4,388	0,978	1,354	M4	11

5,940	4,232	0,960	1,404	M3	10
5,953	4,400	0,970	1,353	M5	34
5,953	4,180	0,966	1,424	M1	5
5,955	4,386	0,965	1,358	M3	5
5,955	4,290	0,982	1,388	M5	6
5,955	4,277	0,971	1,392	M3	6
5,955	4,248	0,981	1,402	M4	9
5,955	4,245	0,971	1,403	M1	5
5,955	4,176	0,973	1,426	M4	7
5,955	4,151	0,962	1,435	M2	7
5,955	4,070	0,960	1,463	M4	11
5,955	4,313	0,978	1,381	M5	3
5,956	4,354	0,975	1,368	M4	3
5,956	4,400	0,973	1,354	M2	10
5,956	4,290	0,971	1,388	M6	9
5,956	4,543	0,980	1,311	M5	7
5,956	4,400	0,976	1,354	M6	16
5,956	4,400	0,962	1,354	M3	5
5,956	4,400	0,974	1,354	M3	9
5,956	4,070	0,957	1,463	M6	13
5,956	4,277	0,967	1,393	M4	8
5,956	4,180	0,950	1,425	M6	8
5,956	4,180	0,975	1,425	M1	9
5,956	4,290	0,960	1,388	M6	5
5,956	4,510	0,994	1,321	M3	9
5,956	4,290	0,982	1,388	M2	13
5,956	4,180	0,960	1,425	M1	2
5,962	4,466	0,991	1,335	M5	4
5,962	4,104	0,962	1,453	M3	7
5,963	4,440	0,979	1,343	M6	14
5,964	4,496	0,986	1,327	M5	14
5,964	4,351	0,974	1,371	M3	11
5,964	4,381	0,981	1,361	M2	3
5,964	4,379	0,972	1,362	M1	5
5,964	4,311	0,981	1,384	M5	7
5,964	4,330	0,981	1,378	M4	4
5,964	4,299	0,977	1,388	M2	11
5,964	4,350	0,961	1,371	M2	6
5,964	4,134	0,963	1,443	M6	3
5,965	4,529	0,999	1,317	M5	27
5,965	4,400	0,993	1,356	M5	6
5,965	4,310	0,979	1,384	M1	11
5,965	4,212	0,967	1,416	M2	7
5,965	4,155	0,966	1,436	M4	6
5,967	4,544	0,987	1,313	M5	7
5,967	4,550	0,983	1,312	M5	11
5,967	4,311	0,978	1,384	M4	15
5,967	4,388	0,978	1,360	M2	6
5,967	4,311	0,970	1,384	M1	7
5,967	4,311	0,957	1,384	M4	7
5,967	4,155	0,956	1,436	M6	8
5,967	4,155	0,964	1,436	M4	6

5,967	4,077	0,965	1,464	M2	13
5,967	4,077	0,968	1,464	M5	7
5,967	4,466	0,994	1,336	M2	14
5,967	4,232	0,974	1,410	M3	12
5,967	4,077	0,964	1,464	M4	8
5,967	4,134	0,962	1,444	M4	30
5,971	4,730	0,916	1,262	M5	16
5,971	4,598	0,994	1,299	M2	5
5,971	4,466	0,992	1,337	M3	5
5,971	4,395	0,982	1,359	M4	10
5,971	4,403	0,988	1,356	M5	12
5,971	4,290	0,991	1,392	M4	24
5,971	4,233	0,977	1,411	M4	6
5,971	4,388	0,981	1,361	M4	5
5,971	4,443	0,977	1,344	M4	13
5,971	4,277	0,976	1,396	M1	15
5,971	4,208	0,972	1,419	M4	5
5,971	4,093	0,940	1,459	M2	8
5,971	4,470	0,979	1,336	M5	6
5,971	4,510	0,992	1,324	M5	19
5,971	4,510	0,976	1,324	M3	10
5,971	4,350	0,978	1,373	M5	8
5,971	3,960	0,950	1,508	M6	7
5,971	4,180	0,965	1,429	M3	11
5,971	4,198	0,967	1,422	M3	7
5,972	4,367	0,976	1,367	M5	18
5,972	4,449	0,992	1,342	M1	5
5,977	4,400	0,986	1,358	M4	2
5,977	4,290	0,976	1,393	M2	8
5,977	4,290	0,980	1,393	M3	5
5,977	4,180	0,967	1,430	M4	7
5,977	4,180	0,960	1,430	M1	11
5,977	3,960	0,953	1,509	M1	10
5,977	4,155	0,964	1,438	M6	29
5,977	4,598	0,992	1,300	M3	3
5,977	4,310	0,973	1,387	M5	6
5,977	4,400	0,992	1,358	M5	16
5,977	4,290	0,976	1,393	M5	18
5,977	4,070	0,957	1,468	M6	12
5,977	4,180	0,965	1,430	M5	12
5,977	4,180	0,965	1,430	M5	13
5,977	4,311	0,986	1,387	M2	14
5,977	4,388	0,967	1,362	M1	6
5,977	4,245	0,964	1,408	M6	6
5,977	4,077	0,955	1,466	M2	8
5,977	4,510	0,968	1,325	M2	4
5,977	4,400	0,965	1,358	M3	5
5,977	4,180	0,966	1,430	M4	5
5,977	4,180	0,967	1,430	M1	8
5,977	4,180	0,967	1,430	M1	10
5,977	4,388	0,981	1,362	M6	16
5,977	4,310	0,976	1,387	M2	5

5,982	4,544	0,981	1,316	M5	5
5,982	4,233	0,967	1,413	M2	3
5,982	4,233	0,945	1,413	M4	11
5,982	4,233	0,960	1,413	M1	3
5,982	4,232	0,964	1,413	M5	6
5,982	4,388	0,973	1,363	M1	12
5,982	4,310	0,959	1,388	M6	5
5,984	4,510	0,986	1,327	M5	2
5,984	4,469	0,983	1,339	M5	18
5,984	4,290	0,977	1,395	M4	6
5,984	4,290	0,965	1,395	M3	4
5,984	4,180	0,970	1,432	M4	5
5,984	4,255	0,970	1,407	M1	2
5,984	4,290	0,959	1,395	M6	14
5,984	4,180	0,965	1,432	M1	9
5,984	4,070	0,950	1,470	M6	8
5,985	4,400	0,983	1,360	M6	2
5,985	4,400	0,982	1,360	M5	15
5,993	4,795	0,986	1,250	M2	21
5,993	4,510	0,988	1,329	M2	5
5,993	4,290	0,982	1,397	M3	5
5,993	4,290	0,979	1,397	M1	9
5,993	4,282	0,956	1,399	M3	6
5,993	4,058	0,962	1,477	M2	6
5,993	4,595	0,979	1,304	M6	2
5,993	4,498	0,973	1,332	M5	39
5,993	4,395	0,974	1,364	M1	6
5,993	4,388	0,976	1,366	M1	14
6,062	4,400	0,965	1,378	M3	2
5,772	4,378	0,982	1,318	M3	3
5,927	4,180	0,962	1,418	M3	6
5,927	4,365	0,982	1,358	M3	8
5,852	4,180	0,983	1,400	M3	9
5,787	4,310	0,986	1,343	M3	10
5,821	4,290	0,978	1,357	M3	15
5,915	4,510	0,934	1,312	M3	17
6,247	4,077	0,940	1,532	M3	18
5,814	4,232	0,972	1,374	M3	5
5,793	3,960	0,956	1,463	M3	6
5,542	4,155	0,986	1,334	M3	7
5,917	4,070	0,976	1,454	M3	5
6,030	4,204	0,965	1,435	M3	2
5,691	4,311	0,990	1,320	M3	4
5,877	4,388	0,978	1,339	M3	11
5,629	4,290	0,968	1,312	M3	6
5,982	4,310	0,974	1,388	M3	7
5,922	4,466	0,987	1,326	M3	10
6,182	4,075	0,915	1,517	M3	9
5,686	4,290	0,980	1,325	M3	7
6,140	4,290	0,968	1,431	M3	9
5,634	4,077	0,972	1,382	M3	6
5,825	4,273	0,980	1,363	M3	5

5,918	3,630	0,901	1,630	M3	8
5,937	4,621	0,976	1,285	M3	6
6,030	4,180	0,960	1,443	M3	7
5,841	4,310	0,951	1,355	M3	7
5,749	4,226	0,970	1,360	M3	9
6,222	4,544	0,953	1,370	M3	11
6,182	4,466	0,944	1,384	M3	13
6,580	4,388	0,939	1,500	M3	18
5,552	4,400	0,997	1,262	M3	6
5,814	4,232	0,969	1,374	M3	6
5,743	4,290	0,986	1,339	M3	6
5,692	4,449	0,976	1,279	M3	10
6,172	4,414	0,978	1,398	M3	2
6,277	4,466	0,971	1,406	M3	7
5,890	4,360	0,986	1,351	M3	4
5,732	4,232	0,974	1,354	M3	6
6,021	4,510	0,998	1,335	M3	7
5,977	4,444	0,961	1,345	M3	5
5,826	4,290	0,993	1,358	M3	5
5,486	4,310	0,991	1,273	M3	9
5,889	4,180	0,981	1,409	M3	5
5,993	4,392	0,962	1,364	M3	7
5,956	4,496	0,985	1,325	M3	7
5,508	4,077	0,971	1,351	M3	8
6,072	4,400	0,972	1,380	M3	10
5,743	4,180	0,981	1,374	M3	5
5,899	4,070	0,944	1,449	M3	2
5,827	4,232	0,970	1,377	M3	3
5,827	4,466	0,987	1,305	M3	6
6,199	4,290	0,958	1,445	M3	7
6,607	4,596	0,938	1,437	M3	8
6,066	4,311	0,969	1,407	M3	9
5,872	4,354	0,987	1,349	M3	12
5,911	4,155	0,960	1,423	M3	4
5,719	4,070	0,970	1,405	M3	4
6,032	4,310	0,966	1,399	M3	4
5,819	4,150	0,973	1,402	M3	5
5,284	4,180	0,989	1,264	M3	5
5,827	4,077	0,959	1,429	M3	7
6,211	4,466	0,964	1,391	M3	9
6,275	4,510	0,963	1,391	M3	7
5,676	4,064	0,970	1,397	M3	3
5,748	4,180	0,960	1,375	M3	6
5,904	4,077	0,965	1,448	M3	8
6,011	4,488	0,984	1,340	M3	14
5,772	4,466	0,991	1,292	M3	5
5,867	4,311	0,974	1,361	M3	6
5,977	4,400	0,971	1,358	M3	8
5,977	4,290	0,958	1,393	M3	5
5,598	4,620	0,998	1,212	M3	7
5,977	4,155	0,965	1,439	M3	3
5,877	4,388	0,992	1,339	M3	6

5,786	4,403	0,978	1,314	M3	7
6,140	4,510	0,974	1,361	M3	11
6,508	4,544	0,946	1,432	M3	13
5,882	4,544	0,986	1,295	M3	4
5,977	4,449	0,976	1,344	M3	6
6,021	4,400	0,974	1,368	M3	6
6,197	4,290	0,971	1,445	M3	9
5,581	4,180	0,975	1,335	M3	4
5,870	4,180	0,942	1,404	M3	5
6,004	4,388	0,962	1,368	M3	3
5,827	4,310	0,987	1,352	M3	6
5,870	4,290	0,967	1,368	M3	9
5,918	4,375	0,981	1,353	M3	10
5,984	4,290	0,971	1,395	M3	5
5,821	4,070	0,957	1,430	M3	7
5,915	4,290	0,965	1,379	M3	5
6,112	4,310	0,967	1,418	M3	6
5,937	4,155	0,959	1,429	M3	6
5,776	4,180	0,950	1,382	M3	2
5,985	4,180	0,949	1,432	M3	3
5,977	4,388	0,970	1,362	M3	5
5,880	4,400	0,978	1,336	M3	11
5,658	4,180	0,962	1,354	M3	15
5,971	4,510	0,978	1,324	M3	7
5,697	4,311	0,953	1,322	M3	4
5,852	4,180	0,981	1,400	M3	8
5,971	4,207	0,970	1,419	M3	2
5,473	4,252	0,990	1,287	M3	6
5,841	4,388	0,988	1,331	M3	12
5,765	4,310	0,985	1,338	M3	14
5,672	4,310	0,971	1,316	M3	15
5,927	4,180	0,968	1,418	M3	6
6,262	5,280	0,951	1,186	M3	7
5,917	4,510	1,000	1,312	M3	14
5,815	4,400	0,963	1,321	M3	16
6,134	4,620	0,983	1,328	M3	17
5,871	4,180	0,979	1,405	M3	21
5,927	4,180	0,968	1,418	M3	6
5,917	4,510	1,000	1,312	M3	17
5,815	4,400	0,963	1,321	M3	19
6,134	4,620	0,983	1,328	M3	20
5,871	4,180	0,979	1,405	M3	24
6,011	4,310	0,966	1,395	M3	2
5,940	4,233	0,964	1,403	M3	3
5,807	4,290	0,961	1,354	M3	5
5,719	4,311	0,983	1,327	M3	7
6,005	4,400	0,960	1,365	M3	15
6,083	4,510	0,966	1,349	M3	19
6,017	4,290	0,945	1,403	M3	22
5,630	4,070	0,980	1,383	M3	4
5,119	3,850	0,957	1,330	M3	5
5,772	4,180	0,978	1,381	M3	7

5,715	4,070	0,968	1,404	M3	10
5,956	4,400	0,975	1,354	M3	5
6,237	4,731	0,941	1,318	M3	9
6,264	4,724	0,941	1,326	M3	10
6,003	4,453	0,948	1,348	M3	11
5,826	4,070	0,965	1,431	M3	3
5,582	4,310	0,990	1,295	M3	5
5,793	4,274	0,972	1,355	M3	6
5,977	4,544	0,996	1,315	M3	9
5,607	4,388	0,989	1,278	M3	3
6,034	4,400	0,962	1,371	M3	7
6,211	4,466	0,967	1,391	M3	10
5,523	4,290	0,974	1,287	M3	9
5,927	4,350	0,979	1,363	M3	2
6,005	4,070	0,942	1,475	M3	5
5,609	4,400	0,993	1,275	M3	6
5,645	4,290	0,991	1,316	M3	7
5,918	4,290	0,976	1,379	M3	8
5,871	4,400	0,982	1,334	M3	9
5,956	4,443	0,993	1,341	M3	11
6,128	4,466	0,972	1,372	M3	12
5,674	4,290	0,984	1,323	M3	16
5,940	4,310	0,963	1,378	M3	17
6,137	4,388	0,961	1,398	M3	17
5,717	4,180	0,935	1,368	M3	18
5,850	4,310	0,960	1,357	M3	20
5,927	4,180	0,969	1,418	M3	5
5,552	4,070	0,980	1,364	M3	5
5,825	4,180	0,973	1,394	M3	7
5,882	4,466	0,991	1,317	M3	9
6,247	4,077	0,940	1,532	M3	10
6,018	4,463	0,985	1,348	M3	15
6,164	4,395	0,959	1,402	M3	6
6,200	4,290	0,959	1,445	M3	8
5,977	4,400	0,961	1,358	M3	11
6,463	4,579	0,934	1,411	M3	2
6,018	4,388	0,966	1,371	M3	3
5,977	4,388	0,988	1,362	M3	6
5,793	4,290	0,971	1,350	M3	7
5,994	4,388	0,961	1,366	M3	9
6,118	4,449	0,979	1,375	M3	10
5,788	4,400	0,964	1,315	M3	11
5,766	4,180	0,971	1,379	M3	12
6,550	4,836	0,952	1,354	M3	14
5,967	4,466	0,976	1,336	M3	16
5,661	4,311	0,981	1,313	M3	19
5,519	3,829	0,936	1,441	M3	20
6,428	4,777	0,972	1,345	M3	22
6,391	4,155	0,933	1,538	M3	4
6,151	4,400	0,947	1,398	M3	5
6,017	4,311	0,974	1,396	M3	7
5,629	4,510	0,974	1,248	M3	9

6,523	4,767	0,930	1,368	M3	11
5,474	4,620	0,979	1,185	M3	13
5,793	4,290	0,973	1,350	M3	14
5,882	4,466	0,973	1,317	M3	15
6,032	4,507	0,966	1,339	M3	16
6,279	4,510	0,964	1,392	M3	18
6,604	4,388	0,925	1,505	M3	21
5,786	4,180	0,978	1,384	M3	6
6,005	4,155	0,918	1,445	M3	9
6,057	4,388	0,950	1,380	M3	10
5,962	4,544	0,979	1,312	M3	13
5,965	4,621	0,972	1,291	M3	19
6,456	4,435	0,960	1,456	M3	7
5,867	4,621	0,970	1,270	M3	8
6,274	4,510	0,966	1,391	M3	11
5,731	4,233	0,946	1,354	M3	12
5,867	4,388	0,985	1,337	M3	13
5,676	4,466	0,992	1,271	M3	14
5,311	4,932	0,996	1,077	M3	5
5,691	4,311	0,977	1,320	M3	7
5,608	4,070	0,953	1,378	M3	10
6,018	4,239	0,964	1,420	M3	12
5,852	4,270	0,967	1,371	M3	5
5,580	3,960	0,968	1,409	M3	8
5,427	4,290	1,000	1,265	M3	9
5,715	4,070	0,977	1,404	M3	5
5,629	4,070	0,957	1,383	M3	6
6,164	4,466	0,944	1,380	M3	3
5,666	4,290	0,968	1,321	M3	5
5,786	4,180	0,951	1,384	M3	7
6,096	4,227	0,953	1,442	M3	2
5,962	4,388	0,978	1,359	M3	3
6,042	4,070	0,956	1,485	M3	5
5,850	4,388	0,979	1,333	M3	9
5,845	4,180	0,971	1,398	M3	17
5,927	4,470	0,980	1,326	M3	2
5,772	4,196	0,987	1,376	M3	4
5,691	4,388	0,981	1,297	M3	6
5,544	4,155	0,971	1,334	M3	8
6,613	3,960	0,912	1,670	M3	9
5,697	4,077	0,970	1,397	M3	10
5,661	4,311	0,984	1,313	M3	11
5,819	4,354	0,989	1,336	M3	13
6,162	4,290	0,968	1,436	M3	2
5,890	4,290	0,972	1,373	M3	5
5,867	4,307	0,975	1,362	M3	8
5,890	4,383	0,987	1,344	M3	9
6,096	4,400	0,984	1,385	M3	2
5,852	4,400	0,988	1,330	M3	6
5,877	4,466	0,978	1,316	M3	2
5,956	4,290	0,968	1,388	M3	6
6,017	4,336	0,968	1,388	M3	8

5,956	4,510	0,989	1,321	M3	9
5,748	3,960	0,969	1,452	M3	9
6,168	4,510	0,963	1,368	M3	14
5,934	4,070	0,952	1,458	M3	2
5,818	4,400	0,972	1,322	M3	12
5,913	4,544	0,979	1,301	M3	13
6,091	4,290	0,971	1,420	M3	14
6,057	4,150	0,952	1,460	M3	8
6,376	4,311	0,944	1,479	M3	11
6,210	4,550	0,976	1,365	M3	14
5,977	4,258	0,964	1,404	M3	15
5,977	4,510	0,964	1,325	M3	17
6,112	4,400	0,973	1,389	M3	19
6,336	4,622	0,975	1,371	M3	8
5,994	4,466	0,969	1,342	M3	5
5,786	4,388	0,983	1,319	M3	7
5,984	4,400	0,988	1,360	M3	9
5,776	4,290	0,998	1,346	M3	10
5,827	4,155	0,988	1,403	M3	12
5,904	4,544	0,977	1,299	M3	14
5,793	4,290	0,966	1,350	M3	3
6,162	4,290	0,951	1,436	M3	11
6,087	4,311	0,970	1,412	M3	3
6,275	4,290	0,966	1,463	M3	5
5,852	4,070	0,962	1,438	M3	4
5,744	4,180	0,968	1,374	M3	7
5,827	4,232	0,978	1,377	M3	8
5,927	4,290	0,969	1,382	M3	11
5,937	4,310	0,959	1,377	M3	5
5,786	4,155	0,962	1,393	M3	8
6,018	4,400	0,979	1,368	M3	10
5,882	4,232	0,973	1,390	M3	11
6,170	4,180	0,946	1,476	M3	13
6,042	3,960	0,921	1,526	M3	2
6,264	4,730	0,963	1,324	M3	3
6,032	4,310	0,971	1,400	M3	6
6,006	4,611	0,958	1,302	M3	7
5,772	4,388	0,972	1,315	M3	8
6,414	4,310	0,950	1,488	M3	11
6,754	4,388	0,945	1,539	M3	12
5,850	4,310	0,964	1,357	M3	14
5,867	4,730	0,979	1,240	M3	15
6,157	4,232	0,958	1,455	M3	16
5,661	4,155	0,960	1,362	M3	18
6,168	4,400	0,980	1,402	M3	21
6,032	4,339	0,956	1,390	M3	3
5,814	4,241	0,963	1,371	M3	5
5,917	4,290	0,940	1,379	M3	7
5,994	4,310	0,966	1,391	M3	9
6,017	4,232	0,971	1,422	M3	10
6,503	4,715	0,948	1,379	M3	12
6,183	4,388	0,966	1,409	M3	18

5,977	4,180	0,964	1,430	M3	3
5,850	4,233	0,965	1,382	M3	5
5,743	4,180	0,967	1,374	M3	6
6,183	4,388	0,959	1,409	M3	9
6,067	4,466	0,971	1,358	M3	10
6,368	4,461	0,958	1,428	M3	13
6,031	4,232	0,964	1,425	M3	15
5,917	4,400	0,987	1,345	M3	4
6,157	4,466	0,972	1,379	M3	7
6,487	4,290	0,931	1,512	M3	11
6,217	4,305	0,967	1,444	M3	15
6,164	4,544	0,970	1,356	M3	4
6,032	4,544	0,975	1,327	M3	7
5,661	3,688	0,948	1,535	M3	3
5,814	4,400	0,989	1,321	M3	5
6,211	4,310	0,964	1,441	M3	7
6,018	4,496	0,994	1,338	M3	9
5,717	3,960	0,969	1,444	M3	11
6,318	4,291	0,950	1,472	M3	12
6,059	4,290	0,969	1,412	M3	3
6,021	4,290	0,957	1,404	M3	5
5,964	4,466	0,977	1,335	M3	6
5,636	4,425	0,963	1,274	M3	7
6,111	4,157	0,959	1,470	M3	9
6,167	4,386	0,973	1,406	M3	10
5,904	4,155	0,962	1,421	M3	11
6,121	4,155	0,956	1,473	M3	13
6,073	4,388	0,974	1,384	M3	14
5,826	4,070	0,958	1,431	M3	15
5,872	4,077	0,932	1,440	M3	19
5,845	4,400	0,979	1,328	M3	22
6,310	4,777	0,969	1,321	M3	5
6,147	4,388	0,970	1,401	M3	7
6,310	4,699	0,982	1,343	M3	8
6,332	4,388	0,964	1,443	M3	10
5,918	4,180	0,950	1,416	M3	11
6,091	4,400	0,966	1,384	M3	14
6,200	4,274	0,971	1,451	M3	16
6,083	4,180	0,956	1,455	M3	19
5,899	4,290	0,962	1,375	M3	6
6,303	4,461	0,967	1,413	M3	7
6,495	4,400	0,952	1,476	M3	9
6,168	4,400	0,975	1,402	M3	10
5,821	4,620	0,976	1,260	M3	17
6,005	4,290	0,965	1,400	M3	6
6,005	3,960	0,935	1,516	M3	7
6,246	4,544	0,970	1,375	M3	9
6,059	4,510	0,973	1,343	M3	12
6,094	4,310	0,965	1,414	M3	4
6,264	5,088	0,921	1,231	M3	12
6,062	4,070	0,951	1,489	M3	14
5,934	4,400	0,964	1,349	M3	3

5,486	4,649	0,975	1,180	M3	6
6,067	4,310	0,962	1,408	M3	8
6,563	4,950	0,989	1,326	M3	10
5,985	4,730	0,996	1,265	M3	11
6,237	4,579	0,973	1,362	M3	18
6,274	4,400	0,959	1,426	M3	20
5,928	4,180	0,959	1,418	M3	23
6,003	4,232	0,969	1,418	M3	3
6,482	4,730	0,977	1,370	M3	4
5,928	4,510	0,993	1,314	M3	6
5,964	4,510	0,966	1,322	M3	12
6,331	4,510	0,954	1,404	M3	14
5,793	4,070	0,973	1,423	M3	17
5,852	4,290	0,995	1,364	M3	20
6,356	4,574	0,980	1,390	M3	22
6,301	4,684	0,972	1,345	M3	23
6,124	4,510	0,967	1,358	M3	24
5,882	4,310	0,968	1,365	M3	8
6,124	4,400	0,971	1,392	M3	12
5,621	4,362	0,985	1,289	M3	14
5,760	4,155	0,961	1,386	M3	7
6,189	4,400	0,955	1,407	M3	12
5,822	4,070	0,970	1,430	M3	3
6,117	4,334	0,974	1,412	M3	4
6,111	4,510	0,957	1,355	M3	5
5,764	4,290	0,956	1,344	M3	7
6,032	4,232	0,963	1,425	M3	11
5,542	3,960	0,957	1,399	M3	12
5,661	4,232	0,981	1,338	M3	15
6,140	4,400	0,970	1,395	M3	19
5,380	4,070	1,000	1,322	M3	20
6,363	4,310	0,949	1,476	M3	3
6,217	4,444	0,962	1,399	M3	4
6,073	4,388	0,973	1,384	M3	7
5,814	4,070	0,954	1,429	M3	8
5,787	4,233	0,956	1,367	M3	9
5,877	4,232	0,959	1,389	M3	22
6,310	4,356	0,960	1,448	M3	6
6,096	4,400	0,983	1,386	M3	9
5,882	4,155	0,958	1,416	M3	7
5,692	4,155	0,974	1,370	M3	4
5,977	4,233	0,954	1,412	M3	5
5,638	3,960	0,961	1,424	M3	6
6,032	3,999	0,949	1,508	M3	8
5,676	4,311	0,976	1,317	M3	9
5,826	4,254	0,969	1,369	M3	10
6,018	4,284	0,959	1,405	M3	12
5,673	4,070	0,955	1,394	M3	2
6,111	4,290	0,954	1,425	M3	7
6,199	4,070	0,936	1,523	M3	6
6,238	4,250	0,947	1,468	M3	8
5,673	4,117	0,982	1,378	M3	9

5,609	4,290	0,979	1,307	M3	12
6,094	4,077	0,933	1,495	M3	4
5,717	4,150	0,975	1,378	M3	5
5,993	4,180	0,970	1,434	M6	4
5,993	4,180	0,963	1,434	M6	14
5,993	4,257	0,963	1,408	M3	7
5,993	4,510	0,976	1,329	M5	24
5,993	4,503	0,992	1,331	M2	24
5,993	4,400	0,989	1,362	M4	12
5,993	4,290	0,979	1,397	M4	10
5,993	4,290	0,982	1,397	M3	3
5,993	4,180	0,963	1,434	M1	17
5,994	4,474	0,932	1,340	M2	5
5,994	4,539	0,976	1,321	M5	4
5,994	4,388	0,969	1,366	M5	5
5,994	4,381	0,965	1,368	M5	9
5,994	4,233	0,958	1,416	M3	9
5,994	4,311	0,967	1,391	M1	7
5,994	4,155	0,964	1,443	M1	4
5,994	4,388	0,978	1,366	M2	8
5,995	4,588	0,966	1,306	M2	7
6,003	4,388	0,971	1,368	M4	3
6,003	4,388	0,991	1,368	M4	7
6,003	4,388	0,973	1,368	M5	5
6,003	4,233	0,969	1,418	M1	8
6,003	4,233	0,950	1,418	M2	3
6,003	4,233	0,975	1,418	M2	9
6,003	4,466	0,979	1,344	M4	4
6,004	4,232	0,982	1,418	M2	15
6,005	4,620	0,988	1,300	M2	12
6,005	4,510	0,979	1,331	M2	4
6,005	4,290	0,968	1,400	M4	17
6,005	4,290	0,965	1,400	M6	10
6,005	4,180	0,972	1,437	M3	18
6,005	4,290	0,971	1,400	M3	8
6,005	4,290	0,966	1,400	M3	13
6,005	4,290	0,961	1,400	M1	4
6,005	4,290	0,970	1,400	M5	5
6,005	4,290	0,966	1,400	M5	19
6,005	4,290	0,958	1,400	M5	3
6,005	4,070	0,949	1,475	M6	6
6,006	4,910	0,982	1,223	M3	6
6,006	4,840	0,978	1,241	M2	33
6,006	4,400	0,964	1,365	M1	3
6,006	4,290	0,978	1,400	M5	12
6,006	4,290	0,969	1,400	M1	9
6,006	4,290	0,974	1,400	M4	17
6,006	4,290	0,972	1,400	M1	12
6,006	4,180	0,979	1,437	M4	6
6,011	4,435	0,990	1,355	M3	4
6,011	4,378	0,963	1,373	M3	12
6,011	4,311	0,964	1,394	M4	13

6,011	4,127	0,966	1,457	M4	8
6,011	4,544	0,976	1,323	M5	11
6,011	4,443	0,979	1,353	M3	12
6,011	4,152	0,962	1,448	M4	5
6,011	4,414	0,975	1,362	M2	19
6,011	4,414	0,975	1,362	M2	21
6,011	4,310	0,978	1,395	M3	4
6,017	4,388	0,972	1,371	M3	7
6,017	4,155	0,965	1,448	M3	10
6,017	3,994	0,938	1,507	M4	4
6,017	4,659	0,983	1,292	M5	69
6,017	4,381	0,963	1,373	M5	28
6,017	4,233	0,972	1,422	M1	6
6,017	4,556	0,952	1,321	M2	31
6,017	4,339	0,961	1,387	M4	7
6,017	4,297	0,965	1,400	M5	10
6,017	4,350	0,973	1,383	M2	9
6,017	4,053	0,957	1,485	M4	9
6,018	4,544	0,995	1,324	M2	22
6,018	4,510	0,990	1,334	M5	10
6,018	4,596	0,991	1,309	M5	8
6,018	4,400	0,986	1,368	M5	10
6,018	4,400	0,967	1,368	M3	17
6,018	4,311	0,967	1,396	M5	7
6,018	4,311	0,971	1,396	M3	8
6,018	4,466	0,979	1,347	M1	7
6,018	4,273	0,966	1,408	M4	7
6,018	4,180	0,975	1,440	M5	16
6,018	4,350	0,977	1,383	M1	7
6,018	4,264	0,960	1,411	M4	3
6,018	4,515	0,979	1,333	M5	52
6,018	4,400	0,977	1,368	M4	13
6,018	4,430	0,976	1,358	M5	15
6,018	4,584	0,986	1,313	M5	13
6,018	4,356	0,969	1,381	M3	6
6,018	4,294	0,975	1,402	M1	10
6,018	4,324	0,967	1,392	M4	5
6,018	4,223	0,968	1,425	M1	6
6,021	4,400	0,970	1,368	M4	10
6,021	4,400	0,986	1,368	M5	14
6,021	4,290	0,969	1,403	M1	8
6,021	4,461	0,974	1,350	M2	9
6,021	4,070	0,987	1,479	M4	13
6,021	4,290	0,982	1,403	M4	8
6,021	4,180	0,970	1,440	M6	4
6,021	4,180	0,968	1,440	M2	7
6,021	4,510	0,974	1,335	M5	13
6,021	4,400	0,995	1,368	M3	9
6,021	4,180	0,970	1,440	M3	3
6,021	4,400	0,991	1,368	M6	6
6,021	4,400	0,984	1,368	M1	3
6,023	4,510	0,965	1,336	M1	12

6,023	4,510	0,965	1,336	M1	13
6,023	4,180	0,963	1,441	M3	4
6,024	4,400	0,951	1,369	M2	11
6,024	4,180	0,942	1,441	M4	7
6,030	4,466	0,978	1,350	M5	33
6,030	4,388	0,985	1,374	M2	28
6,030	4,388	0,973	1,374	M1	3
6,030	4,311	0,966	1,399	M3	7
6,030	4,311	0,965	1,399	M1	9
6,030	4,077	0,959	1,479	M3	17
6,030	4,155	0,957	1,451	M2	23
6,030	4,155	0,939	1,451	M3	14
6,030	4,510	0,991	1,337	M4	4
6,030	4,070	0,952	1,482	M2	7
6,030	4,310	0,970	1,399	M5	13
6,030	4,232	0,976	1,425	M5	9
6,030	4,203	0,960	1,435	M5	9
6,031	4,310	0,968	1,399	M3	7
6,031	4,280	0,965	1,409	M2	13
6,031	4,564	0,983	1,321	M5	6
6,031	4,544	0,992	1,327	M2	4
6,031	4,398	0,976	1,371	M2	20
6,031	4,510	0,987	1,337	M1	6
5,821	4,180	0,960	1,393	M3	7
5,890	4,290	0,972	1,373	M3	9
5,904	4,310	0,958	1,370	M3	10
6,514	4,400	0,910	1,480	M3	11
5,636	4,855	0,973	1,161	M3	12
5,965	4,459	0,950	1,338	M3	18
5,725	3,960	0,956	1,446	M3	19
5,793	4,070	0,971	1,423	M3	2
6,257	4,507	0,974	1,388	M3	4
5,880	4,400	0,975	1,336	M3	6
5,956	4,510	0,973	1,321	M3	7
5,691	4,233	0,974	1,344	M3	10
6,071	4,400	0,971	1,380	M3	7
5,993	4,510	0,973	1,329	M3	8
5,911	4,717	0,941	1,253	M3	10
5,814	4,290	0,991	1,355	M3	11
5,765	4,400	0,994	1,310	M3	12
5,865	4,510	0,980	1,301	M3	3
5,904	4,232	0,975	1,395	M3	5
5,787	4,466	0,972	1,296	M3	10
5,786	4,180	0,982	1,384	M3	2
5,821	4,290	0,980	1,357	M3	5
5,827	4,398	0,992	1,325	M3	6
5,787	4,155	0,954	1,393	M3	8
6,120	4,378	0,957	1,398	M3	14
5,504	4,290	0,973	1,283	M3	5
6,210	4,303	0,950	1,443	M3	7
6,300	4,544	0,954	1,387	M3	9
5,928	4,290	0,971	1,382	M3	12

6,072	4,388	0,972	1,384	M3	3
6,147	4,233	0,952	1,452	M3	6
5,772	4,106	0,977	1,406	M3	4
5,777	4,070	0,946	1,419	M3	6
5,373	3,999	1,000	1,344	M3	8
5,636	4,388	0,977	1,284	M3	10
6,034	4,070	0,960	1,483	M3	2
6,087	4,456	0,967	1,366	M3	4
5,956	4,620	0,988	1,289	M3	6
6,275	4,180	0,948	1,501	M3	9
5,826	4,466	0,972	1,304	M3	10
5,899	4,290	0,964	1,375	M3	11
5,787	4,311	0,982	1,342	M3	12
6,032	4,326	0,956	1,394	M3	14
5,904	4,232	0,950	1,395	M3	3
6,265	4,400	0,940	1,424	M3	5
5,725	4,180	0,965	1,370	M3	9
5,274	3,850	0,996	1,370	M3	13
6,162	4,510	0,984	1,366	M3	18
5,927	4,284	0,963	1,384	M3	21
5,814	4,070	0,979	1,428	M3	4
5,882	4,129	0,942	1,424	M3	2
5,899	4,070	0,964	1,449	M3	6
5,821	4,180	0,975	1,393	M3	7
6,083	4,400	0,981	1,382	M3	14
5,850	3,999	0,950	1,463	M3	6
6,058	4,155	0,939	1,458	M3	7
5,918	4,077	0,936	1,452	M3	8
6,111	4,388	0,936	1,393	M3	10
6,481	4,620	0,930	1,403	M3	11
6,062	4,290	0,958	1,413	M3	13
6,170	4,510	0,955	1,368	M3	15
5,502	3,850	0,901	1,429	M3	20
6,275	4,620	0,965	1,358	M3	21
5,964	4,328	0,978	1,378	M3	5
5,793	4,400	0,978	1,317	M3	9
6,459	4,400	0,947	1,468	M3	12
6,134	4,434	0,967	1,383	M3	16
5,850	4,180	0,958	1,399	M3	3
5,841	4,388	0,977	1,331	M3	5
6,017	4,388	0,969	1,371	M3	10
5,327	3,922	0,972	1,358	M3	11
5,977	4,510	0,939	1,325	M3	7
6,217	4,620	0,950	1,346	M3	8
5,955	4,950	0,967	1,203	M3	9
6,189	4,510	0,934	1,372	M3	10
5,504	3,960	0,965	1,390	M3	6
6,277	4,232	0,927	1,483	M3	7
5,870	4,070	0,962	1,442	M3	10
6,998	5,119	0,932	1,367	M3	12
5,913	4,466	0,962	1,324	M3	13
5,748	4,250	0,963	1,352	M3	15

6,387	4,400	0,951	1,451	M3	16
5,940	4,388	0,965	1,354	M3	17
6,200	4,510	0,981	1,375	M3	18
6,264	4,381	0,951	1,430	M3	22
5,818	4,311	0,958	1,350	M3	2
6,059	4,180	0,980	1,449	M3	4
5,571	4,077	0,969	1,366	M3	9
5,630	4,070	0,959	1,383	M3	11
6,168	4,400	0,972	1,402	M3	2
5,967	4,388	0,968	1,360	M3	6
5,724	4,155	0,963	1,378	M3	7
5,687	4,400	0,975	1,292	M3	8
6,067	4,310	0,961	1,407	M3	10
6,454	4,628	0,965	1,394	M3	11
5,937	4,466	0,978	1,329	M3	16
6,017	4,544	0,972	1,324	M3	13
5,786	4,449	0,988	1,300	M3	16
6,030	4,622	0,951	1,305	M3	2
6,021	4,400	0,979	1,368	M3	4
5,815	4,400	0,986	1,321	M3	6
6,275	4,290	0,960	1,463	M3	8
5,899	4,290	0,978	1,375	M3	9
5,911	4,544	0,978	1,301	M3	12
5,826	4,180	0,966	1,394	M3	13
6,096	4,620	0,984	1,319	M3	2
5,917	4,400	0,972	1,345	M3	5
6,183	4,544	0,976	1,361	M3	6
5,993	4,180	0,972	1,434	M3	13
6,189	4,510	0,968	1,372	M3	14
5,962	4,388	0,967	1,359	M3	3
6,058	4,257	0,972	1,423	M3	6
5,953	4,290	0,962	1,388	M3	9
6,087	4,699	0,968	1,295	M3	2
5,772	4,400	1,000	1,312	M3	7
6,121	4,621	0,970	1,324	M3	12
4,884	4,070	0,966	1,200	M3	13
6,228	4,510	0,970	1,381	M3	15
6,414	4,466	0,958	1,436	M3	16
6,087	4,699	0,968	1,295	M3	2
5,772	4,400	1,000	1,312	M3	7
6,121	4,621	0,970	1,324	M3	12
6,228	4,510	0,970	1,381	M3	17
6,414	4,466	0,958	1,436	M3	18
6,148	4,466	0,963	1,377	M3	5
6,032	4,466	0,974	1,351	M3	8
6,211	4,336	0,959	1,432	M3	12
6,275	4,840	0,997	1,296	M3	5
5,825	4,510	0,998	1,292	M3	6
5,918	4,460	0,971	1,327	M3	10
6,456	4,720	0,982	1,368	M3	18
5,719	4,232	0,964	1,351	M3	2
5,431	3,893	0,963	1,395	M3	5

6,157	4,466	0,957	1,379	M3	8
5,841	4,388	0,991	1,331	M3	9
5,793	4,290	0,976	1,350	M3	6
5,871	4,466	0,979	1,315	M3	9
6,183	4,466	0,954	1,384	M3	17
5,579	4,070	0,971	1,371	M3	6
5,962	4,544	0,979	1,312	M3	8
6,124	4,509	0,973	1,358	M3	9
5,819	4,479	0,971	1,299	M3	3
5,928	4,510	0,968	1,314	M3	6
6,064	4,730	0,981	1,282	M3	8
6,151	4,290	0,944	1,434	M3	2
6,140	4,290	0,969	1,431	M3	3
5,826	4,310	0,975	1,352	M3	5
6,278	4,510	0,952	1,392	M3	7
6,352	4,290	0,937	1,481	M3	8
6,158	4,621	0,917	1,332	M3	13
5,786	4,466	0,975	1,296	M3	14
6,157	4,466	0,960	1,379	M3	16
5,871	4,233	0,972	1,387	M3	17
6,364	4,310	0,948	1,476	M3	23
6,210	4,544	0,976	1,367	M3	25
5,917	4,400	0,975	1,345	M3	6
6,711	4,620	0,944	1,453	M3	7
6,113	4,560	0,965	1,341	M3	10
6,183	4,855	0,974	1,274	M3	13
6,850	4,621	0,935	1,482	M3	7
5,967	4,388	0,965	1,360	M3	8
6,018	4,510	0,975	1,334	M3	13
5,899	4,070	0,947	1,449	M3	4
5,865	4,155	0,979	1,412	M3	7
5,918	4,310	0,977	1,373	M3	3
6,118	4,405	0,960	1,389	M3	2
5,889	4,330	0,976	1,360	M3	3
6,071	4,383	0,972	1,385	M3	6
5,852	4,180	0,957	1,400	M3	7
5,626	4,339	0,997	1,296	M3	10
5,850	4,180	0,971	1,400	M3	15
5,984	4,290	0,980	1,395	M3	4
6,032	4,155	0,960	1,452	M3	6
5,731	4,270	0,961	1,342	M3	5
5,582	3,999	0,952	1,396	M3	6
6,032	4,310	0,936	1,399	M3	8
5,637	4,155	0,989	1,357	M3	10
6,134	3,903	0,917	1,572	M3	11
5,927	4,180	0,965	1,418	M3	3
6,096	4,284	0,956	1,423	M3	8
6,091	4,290	0,974	1,420	M3	6
6,472	4,180	0,931	1,548	M3	10
5,822	4,070	0,969	1,430	M3	4
5,882	4,155	0,942	1,416	M3	8
5,481	4,232	0,964	1,295	M3	8

6,091	4,226	0,946	1,441	M3	10
6,275	4,180	0,937	1,501	M3	3
6,199	4,178	0,957	1,484	M3	5
6,032	4,155	0,951	1,452	M3	6
5,934	3,960	0,943	1,499	M3	11
6,162	3,960	0,929	1,556	M3	4
6,164	4,462	0,958	1,381	M3	6
5,336	3,999	0,967	1,334	M3	11
5,940	3,921	0,949	1,515	M3	2
6,222	4,138	0,933	1,504	M3	3
5,852	4,163	0,960	1,406	M3	5
6,217	4,510	0,946	1,379	M3	6
5,807	3,740	0,951	1,553	M3	10
6,093	4,311	0,957	1,414	M3	6
5,726	4,290	0,987	1,335	M3	4
5,185	4,193	0,995	1,237	M3	5
5,826	4,400	0,960	1,324	M3	7
5,658	4,070	0,957	1,390	M3	9
5,748	4,290	0,993	1,340	M3	10
6,067	4,310	0,954	1,408	M3	16
6,237	4,180	0,923	1,492	M3	5
6,030	4,544	0,967	1,327	M3	6
5,819	4,290	0,954	1,356	M3	8
5,776	4,290	0,964	1,346	M3	17
6,087	4,388	0,975	1,387	M3	6
5,786	4,290	0,979	1,349	M3	5
5,845	4,290	0,961	1,362	M3	11
6,481	4,730	0,940	1,370	M3	13
5,787	4,232	0,970	1,367	M3	7
5,786	4,290	0,980	1,349	M3	9
5,872	4,217	0,965	1,393	M3	13
6,094	4,232	0,963	1,440	M3	4
5,882	4,232	0,966	1,390	M3	5
6,031	4,289	0,966	1,406	M3	5
6,032	4,679	0,978	1,289	M3	7
5,504	4,145	0,985	1,328	M3	4
5,918	4,290	0,964	1,379	M3	5
5,918	4,510	0,956	1,312	M3	7
5,889	4,290	0,981	1,373	M3	8
5,765	4,388	0,976	1,314	M3	12
5,994	4,388	0,979	1,366	M3	13
5,927	4,400	0,966	1,347	M3	2
5,927	4,305	0,970	1,377	M3	4
5,793	4,290	0,968	1,350	M3	9
6,217	4,450	0,978	1,397	M3	16
5,821	4,290	0,969	1,357	M3	3
5,927	4,248	0,983	1,395	M3	5
5,825	4,446	0,986	1,310	M3	8
5,955	4,544	0,959	1,311	M3	10
6,117	4,417	0,977	1,385	M3	12
5,977	4,400	0,963	1,358	M3	5
5,786	3,999	0,965	1,447	M3	8

5,715	4,400	0,974	1,299	M3	6
6,386	4,400	0,941	1,451	M3	16
5,922	4,466	0,987	1,326	M3	2
5,743	4,388	0,970	1,309	M3	3
6,264	4,310	0,969	1,453	M3	7
6,017	4,150	0,953	1,450	M3	8
6,463	4,354	0,939	1,484	M3	12
5,982	4,388	0,955	1,363	M3	9
6,151	4,400	0,958	1,398	M3	11
6,034	4,290	0,961	1,406	M3	12
6,409	4,783	0,950	1,340	M3	13
6,184	4,388	0,962	1,409	M3	16
6,199	4,400	0,970	1,409	M3	4
6,140	4,674	0,951	1,314	M3	7
6,094	4,232	0,958	1,440	M3	10
6,304	4,347	0,947	1,450	M3	3
5,994	4,436	0,956	1,351	M3	10
6,266	4,510	0,960	1,389	M3	13
6,367	4,180	0,946	1,523	M3	21
6,208	4,313	0,941	1,439	M3	3
6,517	4,621	0,951	1,410	M3	6
5,994	4,388	0,958	1,366	M3	8
6,348	4,290	0,930	1,480	M3	15
6,523	4,544	0,959	1,435	M3	8
5,964	4,496	0,965	1,327	M3	6
5,880	4,180	0,970	1,407	M3	9
5,827	4,291	0,989	1,358	M3	9
6,363	4,077	0,942	1,561	M3	7
5,764	4,180	0,955	1,379	M3	8
5,882	4,461	0,951	1,319	M3	10
5,850	4,620	0,999	1,266	M3	12
5,673	4,070	0,948	1,394	M3	18
5,845	3,850	0,937	1,518	M3	3
6,058	4,155	0,946	1,458	M3	4
6,017	4,155	0,952	1,448	M3	9
5,890	4,510	0,979	1,306	M3	5
5,772	4,310	0,967	1,339	M3	4
6,510	4,466	0,912	1,458	M3	16
6,157	4,500	0,972	1,368	M3	8
5,922	4,155	0,963	1,425	M3	7
5,956	4,070	0,973	1,463	M3	7
5,766	4,077	0,963	1,414	M3	5
5,882	3,999	0,953	1,471	M3	6
5,918	4,070	0,958	1,454	M3	3
5,717	4,155	0,975	1,376	M3	7
5,772	4,232	0,958	1,364	M3	5
6,030	4,310	0,974	1,399	M3	8
6,031	4,398	0,981	1,371	M1	3
6,031	4,410	0,963	1,368	M1	4
6,031	4,290	0,967	1,406	M2	10
6,031	4,226	0,965	1,427	M4	4
6,031	4,220	0,961	1,429	M4	10

6,032	4,466	0,977	1,351	M4	6
6,032	4,471	0,985	1,349	M3	5
6,032	4,257	0,966	1,417	M5	6
6,032	4,589	0,982	1,314	M5	7
6,032	4,466	0,980	1,351	M2	8
6,032	4,311	0,970	1,399	M1	3
6,032	4,443	0,983	1,358	M1	5
6,032	4,466	0,977	1,351	M1	5
6,032	4,232	0,965	1,425	M4	9
6,032	4,433	0,966	1,361	M6	5
6,032	4,297	0,971	1,404	M5	11
6,032	4,449	0,976	1,356	M4	10
6,032	4,310	0,974	1,400	M2	12
6,032	3,999	0,951	1,508	M2	6
6,034	4,510	0,974	1,338	M3	12
6,034	4,400	0,970	1,371	M1	12
6,034	4,180	0,968	1,443	M4	6
6,034	4,180	0,959	1,443	M4	15
6,034	4,180	0,959	1,443	M4	15
6,034	4,070	0,970	1,482	M1	10
6,034	3,960	0,965	1,524	M3	6
6,034	4,555	0,965	1,325	M5	13
6,034	4,180	0,972	1,443	M5	13
6,034	4,290	0,971	1,406	M6	17
6,034	4,180	0,969	1,443	M5	11
6,034	4,180	0,960	1,443	M3	16
6,034	4,180	0,970	1,443	M2	10
6,034	4,070	0,962	1,482	M1	8
6,034	4,400	0,980	1,371	M3	12
6,042	4,180	0,960	1,446	M1	8
6,042	4,510	0,971	1,340	M3	12
6,042	4,400	0,962	1,373	M2	19
6,042	4,400	0,974	1,373	M2	23
6,042	4,290	0,956	1,408	M3	13
6,042	4,180	0,968	1,446	M1	6
6,042	4,180	0,945	1,446	M5	20
6,043	4,290	0,975	1,409	M3	6
6,043	4,400	0,967	1,373	M5	4
6,053	3,999	0,952	1,513	M4	7
6,057	4,466	0,963	1,356	M3	7
6,057	4,388	0,976	1,380	M2	6
6,057	4,233	0,967	1,431	M4	13
6,057	4,388	0,969	1,380	M4	2
6,057	4,388	0,965	1,380	M1	6
6,057	4,232	0,970	1,431	M3	11
6,057	4,232	0,965	1,431	M1	18
6,057	4,466	0,983	1,356	M1	7
6,057	4,388	0,971	1,380	M5	9
6,057	4,311	0,971	1,405	M6	6
6,058	4,489	0,978	1,349	M5	12
6,058	4,544	0,982	1,333	M3	9
6,058	4,449	0,958	1,362	M5	5

6,058	4,310	0,968	1,405	M2	29
6,058	4,292	0,971	1,411	M3	15
6,058	4,299	0,973	1,409	M4	11
6,058	4,700	0,977	1,289	M2	6
6,058	4,400	0,970	1,377	M6	10
6,058	4,284	0,963	1,414	M4	3
6,058	4,180	0,968	1,449	M1	6
6,058	4,070	0,948	1,489	M3	9
6,059	4,367	0,967	1,387	M5	15
6,059	4,362	0,979	1,389	M2	8
6,059	4,507	0,988	1,344	M1	3
6,059	4,180	0,970	1,449	M3	5
6,059	4,292	0,962	1,412	M4	16
6,061	4,510	0,965	1,344	M1	7
6,061	4,070	0,961	1,489	M1	12
6,061	4,070	0,924	1,489	M6	8
6,062	4,180	0,971	1,450	M2	3
6,062	4,180	0,961	1,450	M4	17
6,062	4,620	0,982	1,312	M5	28
6,062	4,290	0,983	1,413	M3	10
6,062	4,400	0,979	1,378	M4	4
6,062	4,290	0,979	1,413	M2	15
6,062	4,290	0,961	1,413	M2	5
6,062	3,960	0,946	1,531	M4	10
6,062	3,960	0,949	1,531	M1	4
6,062	4,290	0,972	1,413	M5	8
6,062	4,290	0,971	1,413	M2	20
6,062	4,290	0,978	1,413	M5	13
6,062	4,290	0,971	1,413	M5	14
6,063	4,400	0,970	1,378	M6	8
6,063	4,400	0,979	1,378	M4	3
6,063	4,290	0,961	1,413	M6	11
6,063	4,328	0,955	1,401	M2	7
6,064	4,510	0,991	1,345	M3	19
6,064	4,400	0,982	1,378	M5	6
6,064	4,530	0,988	1,339	M5	20
6,064	4,354	0,968	1,393	M5	9
6,064	4,388	0,989	1,382	M4	7
6,064	4,339	0,969	1,398	M1	8
6,064	4,376	0,970	1,386	M5	4
6,064	4,294	0,975	1,412	M1	12
6,066	4,311	0,959	1,407	M1	8
6,066	4,311	0,975	1,407	M2	22
6,066	4,311	0,975	1,407	M2	24
6,067	4,388	0,967	1,383	M2	17
6,067	4,388	0,963	1,383	M2	6
6,071	4,599	0,978	1,320	M5	10
6,071	4,546	0,981	1,336	M2	19
6,071	4,620	0,986	1,314	M2	11
6,071	4,434	0,978	1,369	M4	10
6,071	4,400	0,965	1,380	M1	3
6,071	4,377	0,974	1,387	M4	23

6,071	4,290	0,960	1,415	M5	15
6,071	4,284	0,973	1,417	M5	10
6,071	4,461	0,980	1,361	M6	8
6,071	4,510	0,984	1,346	M5	9
6,071	4,496	0,988	1,350	M4	11
6,071	4,403	0,980	1,379	M2	12
6,071	4,233	0,952	1,434	M3	3
6,071	4,164	0,962	1,458	M1	3
6,071	4,510	0,985	1,346	M4	14
6,071	4,400	0,957	1,380	M4	9
6,071	4,529	0,983	1,340	M5	3
6,071	4,423	0,980	1,373	M3	3
6,072	5,114	0,917	1,187	M2	27
6,072	4,388	0,975	1,384	M3	4
6,072	4,417	0,975	1,375	M3	3
6,072	4,183	0,952	1,451	M6	6
6,072	4,466	0,977	1,360	M3	16
6,072	4,466	0,975	1,360	M1	6
6,072	4,482	0,975	1,355	M5	11
6,072	4,365	0,973	1,391	M5	8
6,072	4,388	0,977	1,384	M1	18
6,073	4,426	0,967	1,372	M4	6
6,073	4,207	0,950	1,444	M3	5
6,083	4,400	0,974	1,382	M2	18
6,083	4,510	0,964	1,349	M5	7
6,083	4,400	0,970	1,382	M5	6
6,083	4,510	0,944	1,349	M6	31
6,083	4,180	0,981	1,455	M2	20
6,083	4,510	0,950	1,349	M1	11
6,083	4,510	0,950	1,349	M1	12
6,083	4,400	0,975	1,383	M5	15
6,083	4,180	0,969	1,455	M5	8
6,087	4,311	0,965	1,412	M3	12
6,087	4,463	0,987	1,364	M5	3
6,087	4,311	0,957	1,412	M4	4
6,087	4,311	0,961	1,412	M2	7
6,087	4,232	0,971	1,438	M4	6
6,087	4,232	0,961	1,438	M4	13
5,927	4,290	0,987	1,382	M4	5
5,764	4,070	0,962	1,416	M4	6
5,327	3,954	0,977	1,347	M4	7
5,764	4,070	0,960	1,416	M4	8
6,062	4,480	0,981	1,353	M4	12
6,017	4,155	0,960	1,448	M4	13
6,536	4,400	0,954	1,486	M4	19
5,658	3,960	0,958	1,429	M4	3
6,352	4,290	0,965	1,481	M4	8
5,570	3,740	0,969	1,489	M4	9
6,053	4,466	0,960	1,355	M4	11
5,743	4,245	0,982	1,353	M4	3
5,826	3,999	0,957	1,457	M4	4
6,386	4,400	0,957	1,451	M4	6

6,182	4,232	0,943	1,461	M4	10
5,764	4,070	0,955	1,416	M4	11
5,431	3,921	0,957	1,385	M4	12
5,955	4,180	0,959	1,425	M4	13
6,164	4,104	0,936	1,502	M4	15
5,993	4,180	0,958	1,434	M4	16
5,904	4,233	0,965	1,395	M4	21
6,170	4,400	0,952	1,402	M4	4
5,609	3,850	0,967	1,457	M4	5
5,760	4,233	0,965	1,361	M4	7
5,793	4,070	0,953	1,423	M4	3
5,850	4,290	0,957	1,364	M4	7
5,940	4,311	0,966	1,378	M4	10
6,124	4,180	0,959	1,465	M4	16
5,882	4,155	0,964	1,416	M4	5
5,580	4,180	0,988	1,335	M4	6
6,083	4,180	0,954	1,455	M4	11
6,042	4,290	0,945	1,408	M4	4
5,967	4,233	0,904	1,410	M4	10
5,880	4,290	0,971	1,371	M4	12
6,301	4,621	0,968	1,363	M4	13
6,170	4,180	0,938	1,476	M4	14
5,772	4,077	0,979	1,416	M4	20
5,582	4,232	0,987	1,319	M4	21
5,786	4,077	0,966	1,419	M4	5
6,301	4,155	0,932	1,517	M4	6
5,882	4,155	0,953	1,416	M4	8
5,995	4,077	0,947	1,470	M4	9
5,928	4,180	0,963	1,418	M4	10
6,317	4,620	0,958	1,367	M4	11
5,732	4,309	0,971	1,330	M4	14
6,197	4,290	0,960	1,444	M4	18
6,413	4,151	0,902	1,545	M4	19
6,087	4,311	0,957	1,412	M4	7
5,697	4,155	0,971	1,371	M4	13
6,356	4,329	0,948	1,468	M4	15
5,793	4,180	0,958	1,386	M4	16
6,170	4,400	0,956	1,402	M4	21
5,818	4,233	0,977	1,375	M4	4
6,237	4,311	0,963	1,447	M4	9
5,826	3,960	0,940	1,471	M4	10
6,171	4,357	0,972	1,416	M4	3
5,815	4,290	0,987	1,355	M4	6
5,927	4,233	0,965	1,400	M4	9
6,140	4,400	0,970	1,395	M4	7
5,918	4,222	0,969	1,402	M4	8
5,826	3,960	0,946	1,471	M4	5
5,977	4,233	0,971	1,412	M4	10
6,531	4,388	0,937	1,488	M4	14
5,672	4,155	0,966	1,365	M4	5
5,825	4,298	0,978	1,355	M4	9
5,865	4,454	0,991	1,317	M4	5

5,433	4,400	0,957	1,235	M4	11
5,841	4,311	0,979	1,355	M4	12
5,937	4,311	0,975	1,377	M4	14
5,786	4,070	0,961	1,422	M4	5
5,786	4,311	0,976	1,342	M4	6
5,940	4,155	0,953	1,430	M4	8
5,526	3,999	0,967	1,382	M4	8
6,238	4,232	0,944	1,474	M4	12
5,927	4,614	0,975	1,285	M4	8
6,064	4,077	0,952	1,487	M4	10
5,622	4,290	0,987	1,310	M4	11
5,826	4,400	0,971	1,324	M4	4
5,882	4,232	0,978	1,390	M4	10
5,918	4,070	0,946	1,454	M4	11
5,899	4,180	0,966	1,411	M4	5
5,777	4,174	0,971	1,384	M4	7
5,764	4,180	0,956	1,379	M4	5
5,985	4,180	0,961	1,432	M4	6
5,977	4,180	0,952	1,430	M4	6
5,719	4,232	0,983	1,351	M4	7
6,120	4,077	0,933	1,501	M4	3
5,956	4,400	0,986	1,354	M4	8
6,337	4,291	0,949	1,477	M4	10
5,772	4,180	0,924	1,381	M4	2
5,692	4,070	0,964	1,398	M4	6
6,117	4,418	0,947	1,385	M4	8
6,061	4,510	0,913	1,344	M4	4
6,034	3,960	0,956	1,524	M4	7
6,111	4,510	0,975	1,355	M4	9
6,358	4,190	0,925	1,517	M4	10
6,156	4,233	0,962	1,454	M4	12
6,003	4,311	0,967	1,393	M4	5
6,064	4,298	0,959	1,411	M4	3
6,017	4,388	0,974	1,371	M4	6
5,922	4,232	0,958	1,399	M4	9
6,317	4,556	0,964	1,387	M4	11
5,645	4,243	0,991	1,330	M4	12
6,867	4,564	0,950	1,505	M4	17
5,940	4,232	0,967	1,404	M4	6
6,117	4,400	0,977	1,390	M4	10
5,683	4,155	0,976	1,368	M4	5
5,913	4,155	0,947	1,423	M4	6
5,971	4,396	0,982	1,358	M4	9
5,977	4,290	0,973	1,393	M4	10
6,094	4,232	0,959	1,440	M4	8
6,067	4,077	0,955	1,488	M4	10
5,821	4,180	0,964	1,393	M4	12
6,094	4,232	0,961	1,440	M4	2
5,380	3,944	0,978	1,364	M4	5
6,127	4,598	0,981	1,333	M4	8
6,062	4,180	0,956	1,450	M4	10
5,865	4,303	0,974	1,363	M4	18

6,134	4,290	0,969	1,430	M4	5
6,519	4,389	0,954	1,485	M4	6
6,017	4,155	0,948	1,448	M4	8
5,814	4,388	0,972	1,325	M4	10
5,673	3,960	0,974	1,433	M4	11
5,899	4,070	0,961	1,449	M4	12
5,777	4,180	0,975	1,382	M4	3
5,984	4,070	0,965	1,470	M4	5
6,309	4,622	0,958	1,365	M4	10
6,222	4,155	0,958	1,498	M4	12
5,899	4,400	0,971	1,341	M4	5
5,629	4,180	0,957	1,347	M4	9
6,275	4,432	0,965	1,416	M4	11
6,030	4,493	0,979	1,342	M4	12
5,717	4,282	0,967	1,335	M4	2
5,825	4,400	0,978	1,324	M4	3
6,147	4,388	0,958	1,401	M4	6
5,466	4,070	0,980	1,343	M4	7
5,636	3,922	0,958	1,437	M4	9
5,845	4,070	0,965	1,436	M4	11
5,956	4,347	0,974	1,370	M4	12
6,164	4,466	0,977	1,380	M4	13
6,111	4,405	0,970	1,387	M4	15
5,994	4,311	0,947	1,391	M4	20
6,257	4,255	0,936	1,470	M4	4
5,881	4,311	0,971	1,364	M4	5
5,793	4,180	0,973	1,386	M4	6
5,571	3,802	0,961	1,465	M4	8
6,032	4,310	0,963	1,399	M4	16
6,112	4,466	0,970	1,369	M4	3
6,210	4,466	0,942	1,391	M4	8
6,310	4,544	0,959	1,389	M4	12
5,918	4,290	0,947	1,379	M4	15
6,309	4,388	0,957	1,438	M4	20
6,062	4,400	0,985	1,378	M4	21
5,927	4,277	0,962	1,386	M4	7
5,724	4,077	0,957	1,404	M4	8
6,333	4,544	0,977	1,394	M4	9
5,922	4,435	0,972	1,335	M4	18
6,032	4,348	0,956	1,387	M4	4
6,034	4,400	0,958	1,371	M4	7
5,743	4,620	0,953	1,243	M4	8
5,922	4,232	0,948	1,399	M4	9
6,134	4,290	0,962	1,430	M4	11
5,918	4,290	0,964	1,379	M4	12
6,032	4,362	0,944	1,383	M4	17
5,776	4,180	0,972	1,382	M4	5
6,263	4,077	0,930	1,536	M4	6
6,428	4,388	0,961	1,465	M4	7
6,134	4,290	0,953	1,430	M4	8
5,607	4,324	0,951	1,297	M4	9
5,776	4,290	0,960	1,346	M4	10

6,200	4,587	0,986	1,352	M4	11
5,480	3,997	0,976	1,371	M4	17
6,167	4,388	0,956	1,406	M4	4
5,777	4,180	0,967	1,382	M4	5
5,934	4,400	0,968	1,349	M4	9
6,011	4,228	0,961	1,422	M4	10
6,488	4,611	0,958	1,407	M4	14
5,993	4,180	0,942	1,434	M4	4
6,124	4,546	0,982	1,347	M4	5
6,140	4,070	0,931	1,509	M4	6
5,936	4,388	0,972	1,353	M4	9
6,128	4,394	0,969	1,395	M4	10
6,222	4,466	0,947	1,393	M4	10
5,956	4,180	0,964	1,425	M4	15
5,899	4,180	0,966	1,411	M4	3
6,032	4,388	0,965	1,374	M4	5
6,222	4,463	0,965	1,394	M4	11
6,032	4,232	0,960	1,425	M4	18
6,064	4,536	0,978	1,337	M4	2
6,005	4,290	0,975	1,400	M4	3
6,090	4,400	0,963	1,384	M4	6
5,826	4,233	0,950	1,377	M4	2
5,748	4,070	0,979	1,412	M4	5
5,542	4,180	0,979	1,326	M4	6
5,807	4,070	0,943	1,427	M4	7
6,042	4,070	0,950	1,485	M4	8
5,502	4,290	0,975	1,283	M4	9
6,058	4,290	0,972	1,412	M4	3
6,117	4,383	0,969	1,396	M4	4
6,065	4,388	0,968	1,382	M4	5
6,301	4,310	0,929	1,462	M4	8
6,199	3,960	0,942	1,565	M4	9
5,870	4,377	0,946	1,341	M4	11
5,936	4,466	0,971	1,329	M4	12
5,877	4,388	0,979	1,339	M4	14
6,363	4,290	0,964	1,483	M4	5
5,918	4,180	0,965	1,416	M4	7
5,912	4,461	0,968	1,325	M4	11
6,096	4,400	0,984	1,386	M4	15
5,726	4,290	0,983	1,335	M4	3
6,127	4,311	0,963	1,421	M4	5
6,111	4,290	0,969	1,425	M4	7
5,927	4,290	0,970	1,382	M4	9
6,169	4,290	0,967	1,438	M4	15
6,096	4,510	0,961	1,352	M4	16
6,043	4,290	0,952	1,409	M4	2
5,691	4,233	0,969	1,345	M4	5
6,112	4,180	0,953	1,462	M4	11
5,889	4,379	0,980	1,345	M4	2
6,018	4,232	0,967	1,422	M4	3
6,111	4,180	0,965	1,462	M4	6
5,227	4,113	1,000	1,271	M4	7

5,818	4,290	0,982	1,356	M4	9
6,087	4,291	0,963	1,418	M4	10
6,531	4,155	0,942	1,572	M4	11
6,063	4,374	0,960	1,386	M4	14
6,358	4,264	0,952	1,491	M4	15
5,544	3,999	0,966	1,386	M4	3
5,927	4,310	0,981	1,375	M4	6
6,096	4,348	0,984	1,402	M4	8
5,904	4,267	0,963	1,384	M4	11
6,168	4,610	0,977	1,338	M4	14
6,438	4,510	0,977	1,428	M4	17
5,661	3,844	0,971	1,473	M4	3
5,786	4,310	0,986	1,342	M4	6
6,091	4,350	0,977	1,400	M4	8
5,882	4,155	0,976	1,416	M4	13
6,118	4,544	0,984	1,347	M4	15
6,332	4,510	0,985	1,404	M4	17
5,993	4,474	0,988	1,340	M4	4
5,582	4,388	0,972	1,272	M4	6
6,169	4,290	0,974	1,438	M4	8
6,059	4,277	0,961	1,417	M4	9
5,725	4,180	0,974	1,370	M4	10
5,552	4,070	0,971	1,364	M4	12
5,956	4,070	0,939	1,463	M4	13
6,018	4,108	0,949	1,465	M4	3
6,058	4,310	0,970	1,406	M4	6
6,018	4,390	0,974	1,371	M4	7
6,278	4,449	0,973	1,411	M4	8
6,124	4,290	0,963	1,428	M4	10
6,376	4,388	0,963	1,453	M4	12
5,626	4,290	0,976	1,311	M4	18
5,985	4,180	0,953	1,432	M4	19
6,112	4,449	0,978	1,374	M4	21
6,073	4,388	0,965	1,384	M4	4
6,151	4,180	0,950	1,471	M4	5
5,731	4,233	0,974	1,354	M4	6
6,087	4,408	0,964	1,381	M4	7
5,480	4,077	0,989	1,344	M4	8
6,005	4,180	0,957	1,437	M4	8
5,717	3,850	0,938	1,485	M4	4
5,927	3,960	0,972	1,497	M4	5
6,172	4,369	0,952	1,413	M4	7
5,715	3,960	0,959	1,443	M4	5
5,608	4,163	0,962	1,347	M4	12
6,487	4,383	0,920	1,480	M4	5
6,120	4,311	0,973	1,420	M4	4
5,904	4,233	0,962	1,395	M4	6
6,032	4,310	0,964	1,399	M4	7
6,227	4,180	0,935	1,490	M4	8
6,167	4,341	0,944	1,421	M4	10
6,337	4,310	0,942	1,470	M4	11
5,934	4,070	0,947	1,458	M4	5

6,058	4,070	0,949	1,489	M4	7
5,572	4,070	0,980	1,369	M4	2
6,211	4,077	0,960	1,523	M4	3
6,017	4,207	0,964	1,430	M4	4
6,164	4,290	0,939	1,437	M4	6
5,658	4,070	0,962	1,390	M4	5
5,953	4,290	0,952	1,388	M4	8
6,083	4,400	0,967	1,382	M4	11
5,904	3,999	0,956	1,476	M4	3
6,400	4,148	0,931	1,543	M4	9
6,364	3,999	0,944	1,591	M4	3
5,486	3,756	0,962	1,461	M4	4
5,765	4,048	0,960	1,424	M4	7
5,827	4,077	0,970	1,429	M4	5
5,715	4,180	0,979	1,367	M4	7
6,112	3,999	0,927	1,528	M4	6
6,137	4,233	0,953	1,450	M4	6
5,850	4,180	0,982	1,400	M4	8
5,822	4,070	0,962	1,430	M4	9
6,410	4,227	0,932	1,516	M4	5
5,927	4,180	0,969	1,418	M4	8
6,259	4,070	0,942	1,538	M4	2
6,456	4,190	0,923	1,541	M4	8
6,168	4,104	0,945	1,503	M4	11
6,062	3,850	0,901	1,574	M4	3
6,071	4,241	0,955	1,432	M4	4
6,162	4,290	0,984	1,436	M4	4
6,510	4,093	0,921	1,590	M4	7
6,273	4,466	0,949	1,405	M4	8
5,927	3,953	0,949	1,500	M4	4
5,984	4,357	0,945	1,373	M4	8
6,409	4,290	0,926	1,494	M4	11
5,922	4,368	0,976	1,356	M4	4
5,872	4,310	0,991	1,362	M4	5
5,532	3,960	0,957	1,397	M4	5
6,072	4,303	0,956	1,411	M4	4
6,111	4,070	0,943	1,502	M4	8
5,870	3,850	0,946	1,525	M4	9
5,321	3,946	0,984	1,348	M4	11
6,021	4,400	0,966	1,368	M4	15
5,814	4,180	0,984	1,391	M4	4
5,825	4,070	0,936	1,431	M4	5
5,841	4,232	0,967	1,380	M4	10
5,995	4,310	0,958	1,391	M4	5
6,140	4,180	0,946	1,469	M4	7
5,852	4,180	0,965	1,400	M4	4
6,246	4,290	0,962	1,456	M4	6
5,953	3,960	0,937	1,503	M4	7
5,724	4,077	0,959	1,404	M4	4
6,011	3,999	0,954	1,503	M4	6
6,112	4,400	0,921	1,389	M4	5
5,658	3,960	0,942	1,429	M4	3

6,453	4,732	0,965	1,364	M4	10
5,918	4,277	0,964	1,384	M4	4
5,872	4,232	0,980	1,387	M4	5
6,157	4,077	0,946	1,510	M4	5
6,247	4,310	0,931	1,449	M4	4
5,967	4,388	0,953	1,360	M4	5
5,880	4,290	0,968	1,371	M4	5
5,542	3,960	0,978	1,400	M4	5
6,072	4,466	0,960	1,360	M4	7
5,786	4,290	0,975	1,349	M4	4
6,042	4,180	0,935	1,446	M4	6
6,168	4,400	0,972	1,402	M4	8
5,764	3,960	0,964	1,456	M4	5
5,645	4,070	0,968	1,387	M4	7
6,184	4,310	0,965	1,435	M4	8
5,686	3,960	0,978	1,436	M4	10
5,524	4,180	0,960	1,321	M4	11
5,818	4,311	0,974	1,350	M4	4
5,748	3,960	0,969	1,452	M4	6
5,927	4,303	0,961	1,377	M4	7
5,687	4,180	0,970	1,360	M4	2
5,877	4,233	0,980	1,388	M4	8
5,940	4,077	0,955	1,457	M4	9
6,246	4,400	0,960	1,420	M4	3
5,609	4,070	0,980	1,378	M4	5
5,737	4,180	0,967	1,372	M4	6
5,882	4,232	0,972	1,390	M4	7
5,826	4,070	0,971	1,431	M4	16
6,157	4,310	0,944	1,428	M4	6
5,927	3,960	0,959	1,497	M4	7
5,852	4,290	0,947	1,364	M4	9
5,841	4,155	0,960	1,406	M4	11
5,827	4,233	0,976	1,377	M4	4
6,067	4,310	0,956	1,408	M4	5
6,011	4,156	0,909	1,446	M4	7
5,677	4,077	0,964	1,392	M4	9
6,369	4,481	0,914	1,421	M4	10
6,297	4,400	0,952	1,431	M4	13
5,865	4,029	0,966	1,456	M4	6
5,751	4,390	0,970	1,310	M4	8
5,927	4,290	0,971	1,382	M4	7
5,474	4,070	0,971	1,345	M4	8
5,918	4,308	0,958	1,374	M4	13
5,676	4,284	0,960	1,325	M4	5
6,278	4,070	0,939	1,543	M4	10
6,087	4,310	0,958	1,412	M2	5
6,087	4,232	0,946	1,438	M2	5
6,090	4,510	0,975	1,350	M3	9
6,090	4,400	0,971	1,384	M3	5
6,090	4,379	0,977	1,391	M4	16
6,090	4,290	0,948	1,420	M6	22
6,090	4,400	0,960	1,384	M3	11

6,090	4,400	0,951	1,384	M5	14
6,090	4,290	0,977	1,420	M6	9
6,090	4,070	0,964	1,496	M3	8
6,090	4,290	0,953	1,420	M5	4
6,091	4,400	0,970	1,384	M5	14
6,091	4,510	0,981	1,350	M6	11
6,091	4,180	0,968	1,457	M5	31
6,091	4,180	0,950	1,457	M1	9
6,091	4,070	0,955	1,497	M2	15
6,093	4,544	0,966	1,341	M4	16
6,093	4,622	0,963	1,318	M6	13
6,093	4,466	0,974	1,364	M5	11
6,093	4,388	0,970	1,388	M5	8
6,093	4,388	0,966	1,389	M2	13
6,093	4,233	0,959	1,440	M4	8
6,093	4,311	0,961	1,414	M5	13
6,094	4,388	0,967	1,389	M2	25
6,094	4,232	0,968	1,440	M2	8
6,094	4,466	0,961	1,365	M1	3
6,094	3,999	0,945	1,524	M1	4
6,096	4,510	0,988	1,352	M3	9
6,096	4,400	0,984	1,385	M4	7
6,096	4,400	0,971	1,385	M3	11
6,096	4,400	0,978	1,385	M2	12
6,096	4,461	0,983	1,366	M6	8
6,096	4,273	0,961	1,426	M1	9
6,096	4,326	0,964	1,409	M3	11
6,096	4,355	0,963	1,400	M3	7
6,096	3,960	0,910	1,539	M6	5
6,096	4,510	0,985	1,352	M6	12
6,096	4,390	0,979	1,389	M5	10
6,096	4,400	0,978	1,385	M3	13
6,096	4,290	0,953	1,421	M1	10
6,096	4,290	0,949	1,421	M2	5
6,096	4,180	0,951	1,458	M1	7
6,096	4,567	0,981	1,335	M2	15
6,096	4,493	0,983	1,357	M4	6
6,096	4,400	0,971	1,386	M5	11
6,096	4,290	0,967	1,421	M4	6
6,111	4,589	0,986	1,332	M3	16
6,111	4,299	0,962	1,421	M6	18
6,111	4,433	0,979	1,379	M2	20
6,111	4,462	0,982	1,370	M5	17
6,111	4,400	0,974	1,389	M5	13
6,111	4,400	0,990	1,389	M5	15
6,111	4,400	0,978	1,389	M5	12
6,111	4,180	0,964	1,462	M5	7
6,111	4,180	0,959	1,462	M3	7
6,111	4,290	0,971	1,425	M5	12
6,111	4,070	0,954	1,502	M6	17
6,111	4,180	0,954	1,462	M1	8
6,111	4,070	0,949	1,502	M6	5

6,111	4,510	0,974	1,355	M5	37
6,111	4,400	0,975	1,389	M4	3
6,111	4,620	0,946	1,323	M6	21
6,111	4,180	0,959	1,462	M1	10
6,111	4,180	0,959	1,462	M1	11
6,111	4,290	0,962	1,425	M1	6
6,111	4,070	0,947	1,502	M5	18
6,111	4,070	0,947	1,502	M5	20
6,111	3,960	0,941	1,543	M4	5
6,112	4,620	0,947	1,323	M1	18
6,112	4,290	0,971	1,425	M4	12
6,112	4,290	0,961	1,425	M4	9
6,112	4,498	0,943	1,359	M1	5
6,112	4,180	0,956	1,462	M3	8
6,112	4,277	0,957	1,429	M4	8
6,112	4,388	0,960	1,393	M1	5
6,112	4,620	0,982	1,323	M1	15
6,112	4,436	0,973	1,378	M1	6
6,112	4,310	0,979	1,418	M2	4
6,112	4,350	0,959	1,405	M3	9
6,112	4,229	0,956	1,445	M4	7
6,113	4,180	0,970	1,462	M5	10
6,113	4,466	0,977	1,369	M3	12
6,113	4,478	0,979	1,365	M5	15
6,113	4,396	0,955	1,391	M4	5
6,117	4,330	0,963	1,413	M5	7
6,117	4,395	0,974	1,392	M4	7
6,117	4,398	0,960	1,391	M5	10
6,117	4,418	0,975	1,385	M1	8
6,117	4,393	0,969	1,392	M4	9
6,118	4,400	0,969	1,390	M1	13
6,118	4,510	0,969	1,357	M3	12
6,118	4,390	0,958	1,393	M4	23
6,118	4,396	0,971	1,392	M4	14
6,118	4,377	0,978	1,398	M1	11
6,118	4,378	0,973	1,397	M1	21
6,120	4,533	0,973	1,350	M5	8
6,120	4,466	0,964	1,370	M5	17
6,120	4,466	0,970	1,370	M2	16
6,120	4,466	0,971	1,370	M3	5
6,120	4,233	0,947	1,446	M2	3
6,121	4,310	0,965	1,420	M2	18
6,121	4,232	0,955	1,446	M6	10
6,121	4,210	0,962	1,454	M4	11
6,121	4,621	0,967	1,324	M3	3
6,121	4,621	0,975	1,324	M2	11
6,121	4,466	0,961	1,371	M5	16
6,124	4,400	0,977	1,392	M1	5
6,124	4,400	0,978	1,392	M4	8
6,124	4,400	0,977	1,392	M2	3
6,124	4,400	0,977	1,392	M2	3
6,124	4,180	0,957	1,465	M1	7

6,124	4,163	0,951	1,471	M3	4
6,124	4,290	0,972	1,428	M6	12
6,124	4,400	0,971	1,392	M6	21
6,125	4,290	0,972	1,428	M1	6
6,125	4,290	0,972	1,428	M1	6
6,125	4,620	0,968	1,326	M5	11
6,125	4,290	0,965	1,428	M3	9
6,125	4,290	0,956	1,428	M2	13
6,127	4,508	0,967	1,359	M3	3
6,127	4,388	0,985	1,396	M3	11
6,127	4,466	0,972	1,372	M1	10
6,127	4,368	0,968	1,403	M2	14
6,127	4,233	0,959	1,447	M1	9
6,127	4,232	0,963	1,448	M5	26
6,127	4,310	0,956	1,422	M5	5
6,127	4,228	0,962	1,449	M4	6
6,127	4,219	0,950	1,452	M1	7
6,128	4,318	0,964	1,419	M4	10
6,132	4,180	0,960	1,467	M2	5
6,132	4,180	0,942	1,467	M1	3
6,134	4,620	0,993	1,328	M2	17
6,134	4,534	0,985	1,353	M5	23
6,134	4,599	0,981	1,334	M5	2
6,134	4,400	0,971	1,394	M5	6
6,134	4,358	0,975	1,408	M5	2
6,134	4,343	0,965	1,412	M1	8
6,134	4,319	0,968	1,420	M1	14
6,134	4,250	0,964	1,443	M3	15
6,134	4,400	0,988	1,394	M6	9
6,134	4,461	0,966	1,375	M1	8
6,134	4,335	0,957	1,415	M6	10
6,134	4,180	0,951	1,467	M1	8
6,134	4,400	0,982	1,394	M5	18
6,134	4,400	0,979	1,394	M2	4
6,137	4,311	0,959	1,424	M3	14
6,137	4,077	0,943	1,505	M4	5
6,137	4,310	0,963	1,424	M2	14
6,140	4,730	0,978	1,298	M3	4
6,140	4,510	0,977	1,361	M1	4
6,140	4,400	0,958	1,395	M1	14
6,140	4,290	0,972	1,431	M1	8
6,140	4,400	0,972	1,395	M1	4
6,140	4,290	0,968	1,431	M6	21
6,140	4,400	0,951	1,395	M1	10
6,140	4,290	0,966	1,431	M1	10
6,140	4,354	0,960	1,410	M5	11
6,140	4,180	0,947	1,469	M6	12
6,157	4,435	0,962	1,388	M4	7
5,683	4,051	0,974	1,403	M4	8
5,807	4,180	0,961	1,389	M4	12
6,034	4,255	0,958	1,418	M4	7
6,200	4,400	0,971	1,409	M4	8

5,609	3,960	0,972	1,416	M4	5
5,841	4,232	0,970	1,380	M4	6
5,446	3,903	0,960	1,395	M4	7
5,882	4,093	0,954	1,437	M4	8
5,872	4,151	0,966	1,415	M4	9
5,872	4,180	0,981	1,405	M4	11
5,967	4,310	0,972	1,385	M4	12
5,867	4,235	0,969	1,385	M4	19
5,927	4,290	0,979	1,382	M4	3
5,786	4,290	0,979	1,349	M4	4
6,073	4,250	0,955	1,429	M4	8
5,927	4,070	0,968	1,456	M4	13
5,697	4,077	0,960	1,397	M4	5
5,934	4,070	0,935	1,458	M4	8
5,867	4,310	0,944	1,361	M4	3
6,728	4,365	0,917	1,542	M4	7
5,619	4,070	0,963	1,381	M4	9
6,030	4,341	0,978	1,389	M4	11
5,852	4,290	0,956	1,364	M4	15
5,749	4,290	0,990	1,340	M4	4
5,852	4,510	0,989	1,298	M4	6
6,303	4,489	0,957	1,404	M4	7
5,743	4,070	0,970	1,411	M4	13
5,880	4,180	0,977	1,407	M4	7
6,071	4,424	0,977	1,372	M4	8
6,121	4,379	0,942	1,398	M4	9
5,971	4,400	0,984	1,357	M4	14
6,167	4,233	0,952	1,457	M4	16
5,977	4,372	0,966	1,367	M4	3
5,870	3,960	0,960	1,482	M4	6
5,927	4,180	0,974	1,418	M4	7
6,157	4,350	0,936	1,415	M4	9
6,167	4,311	0,967	1,431	M4	15
6,340	4,180	0,917	1,517	M4	3
5,927	4,180	0,965	1,418	M4	7
6,692	4,290	0,911	1,560	M4	8
5,021	4,271	0,994	1,176	M4	7
6,071	4,180	0,950	1,452	M4	10
6,197	4,180	0,928	1,483	M4	11
5,772	4,077	0,960	1,416	M4	5
5,977	4,449	0,975	1,343	M4	10
6,112	4,311	0,941	1,418	M4	13
6,058	3,921	0,925	1,545	M4	4
6,337	4,388	0,940	1,444	M4	6
5,964	3,999	0,971	1,491	M4	8
6,167	4,533	0,983	1,360	M4	12
6,273	4,388	0,965	1,430	M4	14
5,937	4,388	0,964	1,353	M4	9
6,031	4,180	0,956	1,443	M4	5
5,841	4,077	0,974	1,433	M4	7
5,786	4,203	0,965	1,377	M4	8
5,571	4,155	0,972	1,341	M4	3

6,058	4,290	0,962	1,412	M4	5
5,619	3,960	0,974	1,419	M4	7
5,772	4,288	0,974	1,346	M4	7
6,264	4,443	0,972	1,410	M4	8
6,071	4,400	0,970	1,380	M4	11
5,850	4,510	0,976	1,297	M4	12
6,087	4,466	0,964	1,363	M4	16
5,927	4,180	0,962	1,418	M4	2
6,071	4,204	0,945	1,444	M4	6
5,917	4,180	0,962	1,416	M4	11
5,967	4,388	0,972	1,360	M4	12
6,237	4,311	0,954	1,447	M4	4
5,552	4,070	0,971	1,364	M4	7
6,210	4,544	0,976	1,367	M4	13
5,683	3,960	0,975	1,435	M4	3
5,676	3,994	0,960	1,421	M4	5
5,787	4,155	0,963	1,393	M4	7
5,917	4,290	0,993	1,379	M4	8
5,673	4,290	0,985	1,322	M4	10
5,273	4,077	0,983	1,293	M4	6
5,661	4,155	0,969	1,362	M4	7
5,580	3,960	0,964	1,409	M4	8
5,956	4,180	0,969	1,425	M4	10
5,972	4,433	0,983	1,347	M4	11
5,580	3,960	0,979	1,409	M4	12
5,749	4,070	0,983	1,412	M4	14
5,904	3,999	0,971	1,476	M4	16
5,552	3,960	0,958	1,402	M4	4
5,691	3,999	0,962	1,423	M4	5
5,819	3,889	0,921	1,496	M4	8
6,311	4,617	0,967	1,367	M4	12
6,111	4,400	0,959	1,389	M4	14
6,011	4,310	0,945	1,395	M4	4
6,073	4,310	0,960	1,409	M4	13
5,581	4,290	0,984	1,301	M4	6
5,717	4,070	0,960	1,405	M4	7
6,363	4,311	0,939	1,476	M4	8
6,006	4,180	0,954	1,437	M4	12
5,934	4,400	0,979	1,349	M4	16
5,880	4,290	0,980	1,371	M4	17
5,993	4,400	0,978	1,362	M4	4
6,064	4,510	0,976	1,345	M4	9
6,363	4,311	0,958	1,476	M4	12
5,977	4,400	0,952	1,358	M4	8
5,922	4,310	0,987	1,374	M4	9
6,005	4,290	0,970	1,400	M4	11
6,237	4,510	0,977	1,383	M4	12
5,507	4,155	0,980	1,325	M4	5
5,825	3,979	0,950	1,464	M4	6
5,744	4,383	0,991	1,310	M4	9
5,881	4,388	0,965	1,340	M4	3
5,090	3,921	0,957	1,298	M4	4

5,787	4,233	0,958	1,367	M4	8
5,764	4,290	0,975	1,344	M4	14
5,937	4,155	0,971	1,429	M4	16
5,672	4,157	0,967	1,364	M4	3
5,890	4,174	0,948	1,411	M4	4
6,030	4,155	0,949	1,451	M4	9
6,147	4,155	0,934	1,479	M4	11
5,963	4,466	0,969	1,335	M4	15
6,071	4,405	0,935	1,378	M4	4
5,871	4,210	0,959	1,395	M4	5
6,447	4,601	0,951	1,401	M4	7
6,162	4,510	0,986	1,366	M4	8
6,487	4,180	0,938	1,552	M4	13
5,877	3,999	0,951	1,469	M4	5
6,118	4,336	0,926	1,411	M4	7
5,812	4,390	0,969	1,324	M4	9
6,157	4,718	0,971	1,305	M4	14
6,087	4,155	0,963	1,465	M4	15
5,748	4,169	0,980	1,379	M4	7
5,634	4,155	0,954	1,356	M4	11
5,598	4,299	0,991	1,302	M4	19
5,697	4,388	0,973	1,298	M4	21
5,786	4,290	0,975	1,349	M4	4
5,852	4,256	0,970	1,375	M4	8
5,716	4,309	0,981	1,326	M4	16
5,913	4,466	0,975	1,324	M4	18
5,994	3,921	0,948	1,529	M4	3
5,609	4,180	0,970	1,342	M4	6
6,168	4,388	0,970	1,406	M4	13
5,726	4,232	0,973	1,353	M4	14
6,003	4,155	0,960	1,445	M4	20
5,867	4,038	0,950	1,453	M4	2
5,772	4,290	0,971	1,345	M4	3
5,626	3,883	0,968	1,449	M4	4
5,719	4,165	0,968	1,373	M4	7
5,825	4,070	0,956	1,431	M4	10
5,977	4,309	0,954	1,387	M4	14
5,872	4,085	0,964	1,437	M4	18
6,021	4,290	0,963	1,403	M4	3
5,446	3,850	0,972	1,415	M4	8
6,157	4,232	0,966	1,455	M4	10
5,502	4,180	0,962	1,316	M4	14
5,889	4,093	0,967	1,439	M4	2
6,034	4,180	0,963	1,444	M4	3
5,882	4,077	0,966	1,443	M4	6
5,676	4,329	0,987	1,311	M4	4
5,922	4,057	0,954	1,460	M4	6
6,117	4,427	0,962	1,382	M4	7
6,170	4,400	0,972	1,402	M4	3
5,683	4,290	1,000	1,325	M4	8
6,011	4,297	0,966	1,399	M4	12
5,940	3,921	0,947	1,515	M4	2

5,825	4,147	0,967	1,405	M4	7
5,772	4,256	0,969	1,356	M4	9
6,017	4,232	0,968	1,422	M4	16
5,871	4,203	0,972	1,397	M4	3
6,003	4,077	0,963	1,473	M4	7
5,871	4,212	0,969	1,394	M4	8
5,619	4,388	0,982	1,280	M4	13
6,003	3,999	0,939	1,501	M4	5
5,825	4,222	0,978	1,380	M4	6
5,486	4,155	0,979	1,320	M4	11
6,222	4,229	0,946	1,471	M4	2
5,940	4,446	0,964	1,336	M4	3
5,911	4,201	0,973	1,407	M4	4
6,336	4,233	0,948	1,497	M4	7
5,298	4,179	1,000	1,268	M4	9
5,812	4,070	0,943	1,428	M4	10
5,637	4,070	0,978	1,385	M4	12
6,137	4,233	0,957	1,450	M4	13
5,619	4,204	0,961	1,337	M4	18
5,711	4,290	0,989	1,331	M4	20
5,629	3,960	0,958	1,422	M4	3
6,030	4,123	0,928	1,463	M4	8
5,751	4,233	0,969	1,359	M4	9
6,222	4,388	0,962	1,418	M4	10
5,889	4,180	0,934	1,409	M4	2
5,955	4,290	0,977	1,388	M4	5
5,580	4,070	0,996	1,371	M4	7
5,716	4,311	0,970	1,326	M4	9
6,062	4,510	0,971	1,344	M4	13
5,725	4,226	0,967	1,355	M4	14
6,303	4,410	0,947	1,429	M4	4
6,137	4,544	0,950	1,351	M4	6
6,030	4,290	0,947	1,406	M4	10
6,083	4,290	0,944	1,418	M4	14
5,731	4,077	0,972	1,406	M4	4
6,961	4,730	0,929	1,472	M4	5
6,257	4,388	0,955	1,426	M4	8
6,112	4,246	0,963	1,439	M4	5
5,579	4,057	0,966	1,375	M4	6
5,764	4,400	0,974	1,310	M4	9
5,993	4,160	0,960	1,441	M4	11
5,940	4,180	0,925	1,421	M4	1
5,870	4,070	0,959	1,442	M4	9
6,211	4,496	0,977	1,381	M4	10
6,021	4,400	0,985	1,368	M4	12
6,032	4,233	0,958	1,425	M4	19
6,021	4,290	0,979	1,404	M4	5
6,134	4,399	0,960	1,394	M4	6
6,011	4,264	0,966	1,410	M4	12
6,032	4,388	0,966	1,375	M4	4
5,849	4,180	0,990	1,399	M4	5
6,222	4,464	0,966	1,394	M4	8

6,064	4,258	0,974	1,424	M4	9
5,877	4,233	0,978	1,388	M4	11
6,057	4,405	0,935	1,375	M4	6
5,572	4,291	0,970	1,299	M4	7
5,814	4,310	0,976	1,349	M4	8
5,743	4,180	0,982	1,374	M4	11
6,031	4,395	0,977	1,372	M4	16
5,748	3,960	0,973	1,452	M4	3
6,359	4,504	0,973	1,412	M4	7
5,871	4,363	0,985	1,346	M4	8
5,807	4,290	0,976	1,354	M4	13
5,417	4,180	0,985	1,296	M4	3
6,427	4,544	0,939	1,414	M4	4
5,827	3,999	0,962	1,457	M4	5
5,777	4,180	0,983	1,382	M4	6
6,018	4,250	0,966	1,416	M4	15
5,719	4,155	0,966	1,377	M4	20
5,904	4,544	0,970	1,299	M4	4
6,318	4,699	0,987	1,344	M4	5
5,825	4,131	0,968	1,410	M4	6
6,403	4,481	0,959	1,429	M4	12
6,304	4,552	0,956	1,385	M4	13
6,172	4,290	0,960	1,439	M4	15
6,111	4,388	0,969	1,393	M4	2
5,772	4,112	0,971	1,404	M4	3
5,922	4,329	0,938	1,368	M4	7
6,168	4,639	0,982	1,329	M4	9
5,772	4,311	0,972	1,339	M4	10
6,403	4,777	0,960	1,340	M4	16
6,317	4,630	0,963	1,364	M4	17
5,691	3,921	0,960	1,451	M4	3
5,751	4,311	0,964	1,334	M4	7
5,927	4,290	0,915	1,382	M4	8
6,400	4,155	0,941	1,540	M4	9
6,157	4,516	0,945	1,363	M4	11
5,827	4,388	0,982	1,328	M4	14
6,032	4,388	0,962	1,375	M4	2
5,928	4,290	0,981	1,382	M4	7
5,889	4,290	0,986	1,373	M4	10
6,223	4,409	0,970	1,412	M4	16
5,726	4,180	0,991	1,370	M4	6
5,749	4,290	0,978	1,340	M4	12
5,580	4,070	0,981	1,371	M4	2
5,519	4,310	1,000	1,281	M4	5
5,297	3,960	0,985	1,338	M4	9
5,870	3,850	0,947	1,525	M4	2
6,087	4,150	0,959	1,467	M4	6
6,032	4,232	0,945	1,425	M4	7
5,899	4,180	0,957	1,411	M4	9
6,067	4,077	0,952	1,488	M4	13
6,157	4,466	0,968	1,379	M4	16
5,523	3,960	0,972	1,395	M4	3

5,645	3,960	0,991	1,426	M4	7
6,096	4,290	0,976	1,421	M4	8
6,003	4,310	0,957	1,393	M4	13
6,199	4,367	0,957	1,420	M4	2
5,967	3,999	0,957	1,492	M4	8
5,786	4,155	0,955	1,393	M4	2
5,890	4,461	0,994	1,320	M4	11
6,247	4,232	0,960	1,476	M4	4
6,128	4,267	0,949	1,436	M4	5
5,922	4,388	0,972	1,350	M4	2
5,760	4,155	0,965	1,386	M4	7
6,018	4,290	0,976	1,403	M4	8
5,953	4,290	0,969	1,388	M4	13
6,183	4,388	0,965	1,409	M4	4
6,017	4,311	0,981	1,396	M4	7
6,454	4,388	0,941	1,471	M4	6
6,058	4,310	0,975	1,405	M4	10
5,825	4,290	0,990	1,358	M4	6
6,140	4,180	0,971	1,469	M2	5
6,140	4,510	0,979	1,361	M1	9
6,140	4,510	0,965	1,361	M1	7
6,140	4,180	0,962	1,469	M5	18
6,140	4,070	0,960	1,509	M6	12
6,140	4,620	0,982	1,329	M1	6
6,140	4,290	0,966	1,431	M3	11
6,140	4,290	0,970	1,431	M4	18
6,140	4,379	0,963	1,402	M1	9
6,147	4,466	0,977	1,376	M5	10
6,147	4,388	0,973	1,401	M1	6
6,147	4,388	0,977	1,401	M3	9
6,147	4,463	0,962	1,378	M5	10
6,148	4,934	0,944	1,246	M2	20
6,148	4,544	0,970	1,353	M5	16
6,148	4,388	0,951	1,401	M5	15
6,148	4,310	0,974	1,426	M2	14
6,148	4,232	0,955	1,453	M4	4
6,148	4,077	0,955	1,508	M4	8
6,151	4,400	0,957	1,398	M1	6
6,151	4,290	0,963	1,434	M1	6
6,151	4,180	0,961	1,471	M1	11
6,151	4,180	0,965	1,471	M1	15
6,151	4,180	0,965	1,471	M1	17
6,151	4,070	0,942	1,511	M5	20
6,151	4,400	0,962	1,398	M3	5
6,151	4,290	0,961	1,434	M3	6
6,156	4,544	0,973	1,355	M5	9
6,156	4,466	0,980	1,378	M2	29
6,156	4,466	0,938	1,378	M3	7
6,156	4,233	0,952	1,454	M1	9
6,156	4,233	0,938	1,454	M1	5
6,156	3,999	0,947	1,539	M4	9
6,157	4,388	0,957	1,403	M6	23

6,157	4,155	0,956	1,482	M2	18
6,157	4,310	0,967	1,428	M3	9
6,157	4,548	0,975	1,354	M3	16
6,157	4,489	0,985	1,371	M5	9
6,157	4,388	0,974	1,403	M2	9
6,157	4,244	0,962	1,451	M4	9
6,157	4,409	0,944	1,397	M2	6
6,162	4,589	0,980	1,343	M1	6
6,162	4,527	0,975	1,361	M5	13
6,162	4,510	0,974	1,366	M3	3
6,162	4,070	0,940	1,514	M1	3
6,162	4,510	0,982	1,366	M3	24
6,162	4,461	0,976	1,381	M3	2
6,162	4,362	0,970	1,413	M1	2
6,162	4,282	0,952	1,439	M5	18
6,164	4,622	0,985	1,334	M4	14
6,164	4,730	0,988	1,303	M2	18
6,164	4,730	0,988	1,303	M2	20
6,164	4,347	0,962	1,418	M4	2
6,164	4,151	0,961	1,485	M4	11
6,164	4,282	0,948	1,439	M5	6
6,164	4,403	0,975	1,400	M2	16
6,164	4,310	0,978	1,430	M2	10
6,164	4,282	0,960	1,440	M4	12
6,167	4,378	0,961	1,409	M3	6
6,167	4,358	0,969	1,415	M3	17
6,167	4,498	0,960	1,371	M1	7
6,167	4,383	0,960	1,407	M5	13
6,167	4,343	0,964	1,420	M1	13
6,167	4,363	0,970	1,414	M5	12
6,167	4,233	0,972	1,457	M4	12
6,167	4,232	0,957	1,457	M1	14
6,167	4,010	0,927	1,538	M6	7
6,168	4,466	0,970	1,381	M3	11
6,168	4,189	0,952	1,473	M2	6
6,168	4,400	0,966	1,402	M1	7
6,168	4,290	0,972	1,438	M1	9
6,168	4,290	0,976	1,438	M4	6
6,168	4,180	0,959	1,476	M4	18
6,168	4,400	0,973	1,402	M5	9
6,168	4,290	0,959	1,438	M4	9
6,168	4,180	0,960	1,476	M4	11
6,170	4,290	0,962	1,438	M3	3
6,170	4,290	0,964	1,438	M6	25
6,170	4,070	0,959	1,516	M5	9
6,170	4,510	0,962	1,368	M1	15
6,170	4,510	0,962	1,368	M1	16
6,170	4,400	0,969	1,402	M1	3
6,170	4,290	0,977	1,438	M5	30
6,170	4,290	0,965	1,438	M3	6
6,171	4,582	0,974	1,347	M1	3
6,171	4,443	0,971	1,389	M5	36

6,171	4,400	0,969	1,403	M4	6
6,171	4,496	0,976	1,373	M3	14
6,171	4,414	0,970	1,398	M5	22
6,171	4,410	0,976	1,399	M4	3
6,171	4,367	0,962	1,413	M5	6
6,171	4,556	0,980	1,355	M5	10
6,171	4,510	0,980	1,368	M4	31
6,171	4,400	0,971	1,403	M3	20
6,171	4,502	0,974	1,371	M2	12
6,171	4,417	0,970	1,397	M1	3
6,171	4,320	0,968	1,429	M4	5
6,171	4,343	0,967	1,421	M4	5
6,172	4,532	0,968	1,362	M2	21
6,172	4,347	0,963	1,420	M4	3
6,172	4,496	0,981	1,373	M5	13
6,172	4,449	0,972	1,387	M1	10
6,172	4,489	0,972	1,375	M1	8
6,172	4,395	0,981	1,404	M4	9
6,182	4,665	0,990	1,325	M3	14
6,182	4,311	0,968	1,434	M1	7
6,182	4,622	0,985	1,338	M5	4
6,182	4,544	0,966	1,361	M2	28
6,182	4,466	0,973	1,384	M3	10
6,182	4,463	0,964	1,385	M5	13
6,182	4,233	0,962	1,461	M1	6
6,182	4,311	0,973	1,434	M1	12
6,182	4,232	0,948	1,461	M1	9
6,182	4,232	0,971	1,461	M3	20
6,183	4,678	0,971	1,322	M4	7
6,183	4,388	0,974	1,409	M3	5
6,183	4,388	0,973	1,409	M5	14
6,183	4,388	0,968	1,409	M4	21
6,183	4,388	0,960	1,409	M1	6
6,183	4,233	0,962	1,461	M4	5
6,183	4,233	0,969	1,461	M2	24
6,184	4,388	0,964	1,409	M2	4
6,184	4,621	0,970	1,338	M3	10
6,184	4,310	0,980	1,435	M4	7
6,184	4,232	0,964	1,461	M4	21
6,184	4,155	0,948	1,488	M3	4
6,189	4,620	0,965	1,340	M1	18
6,189	4,290	0,959	1,443	M4	9
6,189	4,290	0,964	1,443	M3	10
6,189	4,290	0,977	1,443	M3	12
6,190	4,620	0,982	1,340	M1	12
6,190	4,620	0,970	1,340	M2	19
6,190	4,400	0,972	1,407	M4	8
6,190	4,290	0,953	1,443	M4	12
6,190	4,070	0,960	1,521	M4	2
6,197	4,400	0,973	1,408	M5	20
6,197	4,400	0,970	1,408	M5	5
6,197	4,400	0,960	1,408	M3	14

6,197	4,290	0,968	1,444	M1	10
6,197	4,271	0,954	1,451	M1	7
6,197	4,630	0,970	1,339	M5	19
6,197	4,290	0,966	1,445	M4	11
6,197	4,481	0,975	1,383	M5	17
6,197	4,290	0,952	1,445	M1	11
6,199	4,620	0,987	1,342	M5	7
6,199	4,649	0,980	1,333	M3	12
6,199	4,502	0,969	1,377	M5	16
6,199	4,510	0,979	1,375	M3	12
6,199	4,400	0,962	1,409	M2	8
6,199	4,463	0,965	1,389	M1	14
6,199	4,274	0,964	1,451	M5	23
6,199	4,348	0,972	1,426	M1	3
6,199	4,440	0,976	1,396	M2	7
6,199	4,290	0,957	1,445	M4	9
6,199	4,322	0,964	1,434	M5	5
6,199	4,290	0,961	1,445	M1	10
6,199	4,226	0,952	1,467	M4	8
6,199	4,180	0,963	1,483	M5	12
6,200	4,201	0,923	1,476	M2	7
6,200	4,620	0,993	1,342	M3	5
6,200	4,400	0,972	1,409	M4	4
6,200	4,180	0,955	1,483	M1	5
6,207	4,544	0,992	1,366	M5	13
6,207	4,466	0,979	1,390	M3	18
6,207	4,466	0,969	1,390	M5	11
6,208	4,461	0,971	1,392	M5	22
6,208	4,510	0,968	1,376	M3	12
6,208	4,372	0,974	1,420	M2	9
6,208	4,529	0,977	1,371	M5	11
6,208	4,388	0,960	1,415	M1	7
6,210	4,311	0,974	1,441	M4	8
6,210	4,155	0,949	1,495	M3	6
6,210	4,556	0,979	1,363	M5	13
6,210	4,533	0,976	1,370	M1	2
6,210	4,357	0,967	1,425	M4	5
6,210	4,496	0,965	1,381	M1	6
6,210	4,367	0,968	1,422	M4	3
6,210	4,179	0,955	1,486	M4	2
6,211	4,768	0,976	1,303	M5	12
6,211	4,423	0,980	1,404	M5	26
6,211	4,310	0,962	1,441	M1	5
6,211	4,449	0,967	1,396	M3	3
5,311	3,960	0,956	1,341	M5	5
5,731	4,290	0,983	1,336	M5	7
5,290	3,850	0,976	1,374	M5	8
6,057	4,388	0,967	1,380	M5	13
5,579	4,400	1,000	1,268	M5	4
5,977	4,388	0,984	1,362	M5	5
5,956	4,620	0,983	1,289	M5	6
5,581	4,070	0,978	1,371	M5	7

5,967	4,388	0,982	1,360	M5	8
5,953	4,180	0,972	1,424	M5	10
6,066	4,388	0,967	1,382	M5	12
5,772	4,310	0,988	1,339	M5	5
5,658	4,180	0,973	1,354	M5	6
5,870	4,290	0,978	1,368	M5	8
6,032	4,388	0,966	1,375	M5	10
6,021	4,350	0,973	1,384	M5	5
6,585	4,749	0,958	1,387	M5	15
6,211	4,291	0,968	1,447	M5	16
6,237	4,678	0,984	1,333	M5	6
6,071	4,695	0,978	1,293	M5	7
5,849	4,510	0,985	1,297	M5	8
6,147	4,463	0,978	1,377	M5	4
5,827	4,489	0,967	1,298	M5	6
5,827	4,466	0,976	1,305	M5	8
5,526	4,155	0,987	1,330	M5	6
5,711	4,400	0,979	1,298	M5	7
5,827	4,310	0,981	1,352	M5	8
5,977	4,310	0,986	1,387	M5	6
5,777	4,400	0,986	1,313	M5	8
5,882	4,456	0,976	1,320	M5	9
5,736	4,070	0,975	1,409	M5	9
6,303	4,620	0,981	1,364	M5	6
6,977	4,855	0,927	1,437	M5	6
6,575	4,596	0,945	1,431	M5	7
5,953	4,290	0,962	1,388	M5	8
6,184	4,699	0,950	1,316	M5	5
5,927	4,290	0,974	1,382	M5	7
6,189	4,510	0,972	1,372	M5	9
5,927	4,290	0,974	1,382	M5	8
6,189	4,510	0,972	1,372	M5	10
5,918	4,290	0,964	1,379	M5	5
5,977	4,544	0,979	1,315	M5	11
6,170	4,180	0,951	1,476	M5	12
5,672	4,311	0,973	1,316	M5	6
5,967	4,155	0,958	1,436	M5	8
5,845	4,290	0,966	1,362	M5	9
5,904	4,232	0,975	1,395	M5	2
5,311	3,850	0,995	1,379	M5	9
6,265	4,489	0,937	1,396	M5	4
6,032	4,324	0,978	1,395	M5	6
6,199	4,303	0,962	1,441	M5	11
5,904	4,233	0,977	1,395	M5	7
5,956	4,180	0,973	1,425	M5	9
5,776	3,960	0,960	1,459	M5	4
6,118	4,290	0,949	1,426	M5	5
6,134	4,425	0,969	1,386	M5	7
6,162	4,290	0,960	1,436	M5	11
5,821	4,180	0,964	1,393	M5	12
6,208	4,423	0,965	1,404	M5	13
6,162	4,730	0,972	1,303	M5	19

5,904	4,077	0,948	1,448	M5	4
6,171	4,350	0,966	1,419	M5	6
5,890	4,400	0,972	1,339	M5	10
5,731	4,311	0,968	1,330	M5	12
6,127	4,310	0,956	1,422	M5	5
6,363	4,582	0,969	1,389	M5	6
5,764	4,290	0,984	1,344	M5	7
5,772	4,257	0,977	1,356	M5	5
5,984	4,400	0,969	1,360	M5	7
5,872	4,502	0,980	1,304	M5	10
6,018	4,388	0,972	1,371	M5	3
5,580	4,383	0,985	1,273	M5	5
5,882	4,119	0,946	1,428	M5	6
5,870	4,290	0,981	1,368	M5	14
5,964	4,510	0,977	1,322	M5	5
6,030	4,180	0,967	1,443	M5	6
6,358	4,398	0,946	1,446	M5	7
6,112	4,311	0,937	1,418	M5	8
5,977	4,180	0,946	1,430	M5	9
5,956	4,290	0,978	1,388	M5	10
6,064	4,642	0,969	1,306	M5	11
6,222	4,699	0,959	1,324	M5	13
5,977	4,180	0,946	1,430	M5	14
5,877	4,311	0,964	1,363	M5	3
6,297	4,400	0,941	1,431	M5	5
6,112	4,496	0,965	1,359	M5	10
5,927	4,510	0,985	1,314	M5	13
5,918	4,290	0,971	1,379	M5	16
5,417	3,960	0,962	1,368	M5	3
5,772	4,232	0,967	1,364	M5	4
5,927	4,397	0,982	1,348	M5	4
6,034	3,960	0,946	1,524	M5	9
5,913	4,466	0,967	1,324	M5	2
5,566	4,077	0,985	1,365	M5	4
5,672	4,410	0,987	1,286	M5	7
5,849	4,290	0,983	1,364	M5	9
6,172	4,542	0,988	1,359	M5	15
5,731	4,155	0,989	1,379	M5	7
5,956	4,290	0,969	1,388	M5	8
6,111	4,560	0,989	1,340	M5	4
6,318	4,466	0,960	1,415	M5	5
6,430	4,620	0,966	1,392	M5	6
5,184	3,740	0,987	1,386	M5	8
5,913	4,155	0,962	1,423	M5	2
5,519	4,180	0,976	1,320	M5	3
5,967	4,466	0,985	1,336	M5	4
5,691	4,311	0,979	1,320	M5	6
5,918	4,400	0,984	1,345	M5	3
5,725	4,290	0,977	1,335	M5	5
6,264	4,390	0,961	1,427	M5	6
6,112	4,508	0,975	1,356	M5	7
5,686	4,070	0,982	1,397	M5	2

5,772	4,232	0,983	1,364	M5	7
5,936	4,311	0,975	1,377	M5	3
6,058	4,449	0,970	1,362	M5	5
5,967	4,388	0,968	1,360	M5	6
5,724	3,999	0,967	1,431	M5	8
5,937	5,610	1,000	1,058	M5	5
5,913	4,310	0,970	1,372	M5	7
5,871	4,400	0,987	1,334	M5	11
6,112	4,622	0,979	1,323	M5	5
6,199	4,730	0,983	1,311	M5	12
6,171	4,620	0,988	1,336	M5	6
5,527	4,310	0,978	1,282	M5	9
6,067	4,388	0,952	1,383	M5	12
6,057	4,310	0,965	1,405	M5	10
5,818	3,960	0,957	1,469	M5	12
6,127	4,311	0,973	1,421	M5	13
5,867	4,310	0,963	1,361	M5	4
5,882	4,310	0,975	1,365	M5	5
5,955	4,544	0,967	1,311	M5	8
6,340	4,496	0,977	1,410	M5	10
5,626	4,400	0,985	1,279	M5	12
5,744	4,400	0,968	1,305	M5	4
5,904	4,232	0,953	1,395	M5	6
5,972	4,284	0,932	1,394	M5	4
5,993	4,290	0,984	1,397	M5	5
5,532	3,850	0,965	1,437	M5	14
5,889	4,290	0,974	1,373	M5	17
5,912	4,621	0,982	1,279	M5	19
5,638	4,290	0,972	1,314	M5	4
6,259	4,290	0,956	1,459	M5	7
5,971	4,303	0,968	1,388	M5	8
5,867	4,544	1,000	1,291	M5	14
6,093	4,155	0,948	1,466	M5	5
5,815	4,070	0,960	1,429	M5	7
6,373	4,388	0,961	1,452	M5	9
5,787	4,155	0,985	1,393	M5	10
5,867	4,018	0,959	1,460	M5	12
6,156	4,155	0,962	1,482	M5	16
5,937	4,258	0,942	1,394	M5	17
6,237	4,388	0,958	1,421	M5	19
5,621	4,311	0,983	1,304	M5	5
5,881	4,244	0,960	1,386	M5	7
5,732	4,310	0,983	1,330	M5	9
6,059	4,070	0,953	1,489	M5	12
5,776	4,180	0,988	1,382	M5	9
6,057	4,503	0,976	1,345	M5	15
6,585	4,711	0,956	1,398	M5	16
6,140	4,542	0,952	1,352	M5	4
6,523	4,466	0,959	1,460	M5	5
5,971	4,479	0,992	1,333	M5	6
5,821	4,425	0,969	1,315	M5	10
5,871	4,400	0,969	1,334	M5	12

6,011	4,699	0,982	1,279	M5	14
5,918	4,436	0,986	1,334	M5	16
6,018	4,445	0,978	1,354	M5	22
5,953	4,180	0,968	1,424	M5	24
5,993	4,290	0,973	1,397	M5	4
5,977	4,290	0,983	1,393	M5	6
5,821	4,290	0,989	1,357	M5	7
6,183	4,388	0,964	1,409	M5	16
6,018	4,527	0,984	1,330	M5	3
5,911	4,417	0,982	1,338	M5	5
5,715	4,180	0,966	1,367	M5	8
6,093	4,311	0,971	1,414	M5	9
5,772	4,180	0,985	1,381	M5	13
5,737	4,070	0,976	1,409	M5	18
6,410	4,510	0,932	1,421	M5	20
6,208	4,699	0,982	1,321	M5	2
6,096	4,499	0,979	1,355	M5	5
5,737	4,290	0,970	1,337	M5	6
5,953	4,290	0,965	1,388	M5	7
5,937	4,381	0,978	1,355	M5	12
6,096	4,508	0,967	1,352	M5	13
5,937	4,232	0,954	1,403	M5	16
6,310	4,434	0,962	1,423	M5	21
5,940	4,155	0,957	1,430	M5	22
6,125	4,180	0,980	1,465	M5	3
6,514	4,620	0,935	1,410	M5	4
6,509	4,629	0,967	1,406	M5	7
5,608	4,290	0,996	1,307	M5	12
5,787	4,232	0,977	1,367	M5	14
6,087	4,311	0,969	1,412	M5	15
5,760	4,311	0,971	1,336	M5	18
6,406	4,556	0,950	1,406	M5	22
6,275	4,730	0,977	1,327	M5	26
6,062	4,290	0,962	1,413	M5	27
5,918	4,430	0,971	1,336	M5	1
5,880	4,354	0,980	1,351	M5	8
5,953	4,290	0,966	1,388	M5	9
6,259	4,620	0,968	1,355	M5	11
5,955	4,330	0,967	1,375	M5	12
5,772	4,145	0,972	1,392	M5	4
6,222	4,611	0,961	1,349	M5	5
5,917	4,180	0,985	1,416	M5	6
5,880	4,290	0,977	1,371	M5	8
5,119	3,850	0,976	1,330	M5	10
6,167	4,388	0,961	1,405	M5	12
5,889	4,290	0,987	1,373	M5	13
6,221	4,699	0,970	1,324	M5	2
5,845	4,180	0,967	1,398	M5	5
5,807	4,180	0,960	1,389	M5	6
5,818	4,562	0,992	1,275	M5	16
5,807	4,400	0,971	1,320	M5	3
6,184	4,621	0,971	1,338	M5	6

6,087	4,410	0,955	1,380	M5	8
5,849	4,070	0,977	1,437	M5	9
6,240	4,400	0,962	1,418	M5	10
6,237	4,466	0,961	1,397	M5	12
6,063	4,510	0,971	1,344	M5	14
6,094	4,466	0,967	1,365	M5	18
5,786	4,077	0,961	1,419	M5	3
5,977	4,311	0,969	1,387	M5	4
5,993	4,510	0,970	1,329	M5	9
5,826	4,290	0,963	1,358	M5	12
5,877	4,466	0,977	1,316	M5	13
5,683	4,400	0,989	1,292	M5	5
6,147	4,233	0,965	1,452	M5	7
6,468	4,466	0,959	1,448	M5	5
6,030	4,400	0,966	1,371	M5	6
6,275	4,694	0,979	1,337	M5	8
6,042	4,400	0,972	1,373	M5	10
6,112	4,544	0,979	1,345	M5	11
5,760	4,232	0,964	1,361	M5	4
6,363	4,855	0,999	1,311	M5	6
5,542	4,070	0,983	1,362	M5	7
6,183	4,466	0,956	1,384	M5	10
5,787	4,077	0,961	1,420	M5	11
5,877	4,233	0,958	1,388	M5	13
5,821	3,960	0,951	1,470	M5	10
5,658	4,180	0,965	1,354	M5	8
6,238	4,232	0,974	1,474	M5	10
5,842	4,232	0,955	1,380	M5	13
5,967	4,155	0,944	1,436	M5	15
5,922	4,155	0,967	1,426	M5	16
5,904	4,777	0,988	1,236	M5	7
6,618	4,777	0,962	1,385	M5	9
6,482	4,700	0,970	1,379	M5	16
5,786	4,388	0,977	1,319	M5	5
5,748	4,400	0,993	1,306	M5	7
6,164	4,699	0,997	1,312	M5	4
6,030	4,290	0,975	1,406	M5	6
6,303	4,678	0,992	1,347	M5	6
6,468	4,588	0,964	1,410	M5	9
6,237	4,290	0,967	1,454	M5	11
6,274	4,565	0,959	1,374	M5	8
6,118	4,510	0,988	1,357	M5	9
5,880	4,510	0,985	1,304	M5	11
6,545	4,730	0,973	1,384	M5	12
6,004	4,388	0,978	1,368	M5	5
5,580	4,180	0,992	1,335	M5	6
6,317	4,898	0,982	1,290	M5	9
5,934	4,290	0,972	1,383	M5	4
6,331	4,620	0,972	1,370	M5	6
6,148	4,466	0,969	1,377	M5	7
5,580	3,960	0,978	1,409	M5	9
6,083	4,400	0,986	1,383	M5	10

6,157	4,544	0,961	1,355	M5	5
6,277	4,544	0,961	1,382	M5	6
5,977	4,232	0,966	1,412	M5	8
5,715	4,180	0,982	1,367	M5	10
5,956	4,470	0,983	1,332	M5	13
6,006	4,400	0,959	1,365	M5	16
5,426	4,077	1,000	1,331	M5	20
6,265	4,730	0,995	1,325	M5	4
6,428	4,855	0,964	1,324	M5	6
5,787	4,310	0,982	1,343	M5	8
5,826	4,510	1,000	1,292	M5	9
6,454	4,855	0,971	1,329	M5	12
6,072	4,699	0,971	1,292	M5	13
6,510	4,683	0,979	1,390	M5	15
6,237	4,679	0,991	1,333	M5	3
6,860	4,855	0,963	1,413	M5	5
6,618	4,933	0,976	1,342	M5	7
5,918	4,444	0,986	1,332	M5	9
6,381	4,510	0,963	1,415	M5	10
6,363	4,946	0,984	1,287	M5	11
5,934	4,400	0,970	1,349	M5	13
6,310	4,544	0,951	1,389	M5	14
6,222	4,910	0,984	1,267	M5	16
6,113	4,489	0,985	1,362	M5	5
6,199	4,589	0,983	1,351	M5	7
6,318	4,595	0,965	1,375	M5	13
5,636	4,310	0,978	1,308	M5	14
6,030	4,510	0,994	1,337	M5	15
6,218	4,611	0,979	1,348	M5	4
6,071	4,197	0,963	1,447	M5	7
6,017	4,534	0,981	1,327	M5	8
6,113	4,543	0,974	1,345	M5	9
5,917	4,400	0,986	1,345	M5	10
6,061	4,510	0,973	1,344	M5	11
6,120	4,544	0,963	1,347	M5	13
6,018	4,662	1,000	1,291	M5	18
6,337	4,614	0,964	1,374	M5	19
5,993	4,180	0,986	1,434	M5	3
5,899	4,510	0,999	1,308	M5	4
6,378	4,730	0,979	1,348	M5	7
6,902	4,922	0,951	1,402	M5	9
5,922	4,535	0,981	1,306	M5	10
5,676	4,310	0,983	1,317	M5	11
6,510	4,544	0,949	1,433	M5	14
6,264	4,567	0,978	1,371	M5	15
6,032	4,388	0,979	1,375	M5	16
5,993	4,510	0,988	1,329	M5	17
6,072	4,599	0,998	1,320	M5	18
6,172	4,620	0,977	1,336	M5	20
6,172	4,621	0,982	1,335	M5	21
6,005	4,510	0,990	1,331	M5	22
6,444	4,722	0,976	1,365	M5	26

6,228	4,510	0,964	1,381	M5	27
6,093	4,378	0,960	1,392	M5	28
6,118	4,509	0,969	1,357	M5	29
6,331	4,609	0,975	1,374	M5	5
5,772	4,388	0,983	1,315	M5	8
5,698	4,510	0,970	1,263	M5	2
6,210	4,510	0,975	1,377	M5	6
6,128	4,466	0,965	1,372	M5	8
6,237	4,510	0,966	1,383	M5	9
5,922	4,466	0,979	1,326	M5	10
5,965	4,620	0,993	1,291	M5	15
6,217	4,433	0,972	1,403	M5	16
6,217	4,582	0,987	1,357	M5	4
5,581	3,960	0,978	1,409	M5	5
6,030	4,449	0,975	1,355	M5	7
5,814	4,180	0,978	1,391	M5	2
5,683	4,203	0,981	1,352	M5	3
6,317	4,483	0,963	1,409	M5	6
5,814	4,250	0,971	1,368	M5	7
6,030	4,510	0,991	1,337	M5	8
6,030	4,310	0,971	1,399	M5	9
6,094	4,536	0,940	1,343	M5	13
5,474	4,070	0,995	1,345	M5	4
5,965	4,621	0,983	1,291	M5	5
5,935	4,290	0,965	1,383	M5	3
5,852	4,290	0,967	1,364	M5	5
5,927	4,290	0,978	1,382	M5	11
6,217	4,624	0,980	1,345	M5	4
5,446	4,290	0,992	1,269	M5	5
5,744	4,180	0,985	1,374	M5	6
5,765	4,155	0,970	1,388	M5	8
6,064	4,450	0,982	1,363	M5	5
5,814	4,290	0,983	1,355	M5	4
6,072	4,408	0,965	1,377	M5	5
6,018	4,152	0,958	1,449	M5	6
5,580	4,290	1,000	1,301	M5	9
6,211	4,466	0,984	1,391	M2	23
6,211	4,310	0,954	1,441	M1	9
6,211	4,606	0,991	1,348	M2	9
6,211	4,456	0,972	1,394	M1	10
6,211	4,423	0,977	1,404	M1	11
6,211	4,148	0,949	1,497	M3	6
6,217	4,391	0,970	1,416	M5	9
6,217	4,400	0,974	1,413	M5	27
6,217	4,311	0,956	1,442	M4	8
6,217	4,457	0,965	1,395	M1	20
6,217	4,373	0,959	1,422	M5	8
6,217	4,297	0,971	1,447	M4	6
6,217	4,388	0,976	1,417	M4	6
6,217	4,400	0,968	1,413	M2	5
6,217	4,211	0,956	1,476	M4	16
6,217	4,510	0,965	1,379	M1	9

6,217	4,400	0,974	1,413	M4	6
6,217	4,510	0,977	1,379	M1	6
6,218	4,620	0,970	1,346	M3	11
6,218	4,510	0,953	1,379	M3	10
6,218	4,400	0,965	1,413	M4	11
6,218	4,290	0,975	1,449	M6	29
6,218	4,400	0,962	1,413	M1	4
6,218	4,180	0,956	1,487	M4	7
6,218	4,070	0,955	1,528	M3	12
6,218	4,070	0,939	1,528	M1	7
6,218	4,379	0,963	1,420	M4	9
6,218	4,204	0,961	1,479	M4	9
6,218	4,231	0,950	1,469	M3	5
6,218	4,290	0,950	1,449	M4	5
6,222	4,388	0,961	1,418	M2	23
6,222	4,388	0,955	1,418	M1	11
6,222	4,155	0,946	1,498	M4	18
6,222	4,471	0,970	1,392	M4	9
6,222	4,622	0,987	1,346	M3	5
6,222	4,388	0,979	1,418	M5	30
6,222	4,233	0,963	1,470	M4	5
6,222	4,189	0,941	1,486	M4	13
6,222	4,462	0,968	1,395	M4	12
6,222	4,426	0,971	1,406	M4	9
6,222	4,489	0,977	1,386	M5	7
6,222	4,445	0,962	1,400	M5	14
6,222	4,443	0,971	1,400	M4	9
6,222	4,233	0,952	1,470	M1	8
6,222	4,237	0,957	1,469	M4	13
6,222	4,310	0,958	1,444	M1	6
6,223	4,466	0,986	1,394	M2	5
6,223	4,745	0,903	1,311	M1	8
6,223	4,378	0,954	1,421	M4	9
6,227	4,620	0,968	1,348	M3	11
6,227	4,400	0,960	1,415	M1	8
6,237	4,341	0,957	1,437	M3	6
6,237	4,728	0,985	1,319	M5	10
6,237	4,728	0,985	1,319	M5	11
6,237	4,388	0,968	1,421	M4	34
6,237	4,388	0,962	1,421	M4	4
6,237	4,490	0,960	1,389	M4	17
6,237	4,466	0,966	1,397	M1	8
6,237	4,388	0,970	1,421	M4	6
6,237	4,602	0,957	1,355	M5	6
6,237	4,479	0,974	1,392	M4	6
6,237	4,400	0,962	1,418	M3	13
6,237	4,277	0,954	1,459	M4	6
6,238	4,388	0,955	1,422	M1	7
6,238	4,243	0,957	1,470	M1	6
6,238	5,280	0,964	1,181	M5	3
6,238	4,310	0,971	1,447	M5	6
6,238	4,388	0,950	1,422	M4	6

6,238	3,999	0,939	1,560	M3	3
6,240	4,180	0,961	1,493	M1	4
6,240	4,180	0,946	1,493	M4	5
6,240	4,180	0,946	1,493	M4	5
6,246	4,290	0,971	1,456	M4	10
6,246	4,290	0,961	1,456	M1	14
6,246	4,400	0,961	1,420	M5	20
6,246	4,400	0,952	1,420	M4	15
6,246	4,290	0,958	1,456	M4	9
6,246	4,290	0,976	1,456	M5	13
6,246	4,290	0,976	1,456	M5	14
6,246	4,180	0,950	1,494	M6	26
6,246	4,274	0,944	1,462	M1	5
6,246	4,388	0,965	1,423	M4	4
6,246	4,466	0,973	1,399	M4	11
6,246	4,388	0,964	1,423	M2	13
6,246	4,149	0,944	1,506	M1	11
6,247	4,290	0,958	1,456	M1	13
6,247	4,070	0,940	1,535	M1	15
6,247	4,388	0,961	1,424	M2	27
6,247	3,999	0,956	1,562	M4	7
6,247	4,232	0,954	1,476	M4	10
6,247	4,310	0,956	1,449	M1	5
6,247	4,310	0,956	1,449	M1	5
6,247	4,077	0,939	1,532	M3	10
6,247	4,694	0,968	1,331	M5	16
6,247	4,388	0,964	1,424	M1	4
6,247	4,310	0,946	1,449	M3	6
6,257	4,529	0,973	1,381	M5	12
6,257	4,443	0,972	1,408	M5	18
6,257	4,410	0,974	1,419	M5	8
6,257	4,409	0,962	1,419	M5	7
6,257	4,383	0,966	1,428	M4	10
6,257	4,544	0,946	1,377	M3	8
6,259	4,070	0,936	1,538	M6	4
6,260	4,290	0,972	1,459	M1	4
6,262	4,621	0,972	1,355	M3	7
6,262	4,443	0,962	1,410	M3	12
6,262	4,400	0,946	1,423	M1	3
6,262	4,264	0,961	1,469	M4	3
6,263	4,544	0,975	1,378	M4	15
6,263	4,544	0,960	1,378	M2	25
6,263	4,508	0,966	1,389	M3	18
6,263	4,417	0,964	1,418	M4	6
6,263	4,381	0,955	1,429	M5	20
6,263	4,474	0,980	1,400	M4	10
6,263	4,310	0,950	1,453	M4	7
6,263	4,456	0,952	1,406	M1	8
6,264	4,474	0,970	1,400	M4	11
6,264	4,383	0,964	1,429	M4	2
6,264	4,546	0,967	1,378	M2	6
6,264	4,434	0,970	1,413	M3	15

6,264	4,510	0,981	1,389	M1	9
6,264	4,310	0,976	1,453	M2	25
6,264	4,310	0,976	1,453	M2	27
6,264	4,310	0,952	1,453	M3	3
6,265	4,510	0,950	1,389	M3	8
6,265	4,471	0,969	1,401	M4	11
6,265	4,180	0,962	1,499	M1	9
6,265	4,451	0,972	1,407	M4	12
6,265	4,479	0,974	1,399	M1	2
6,266	4,180	0,943	1,499	M1	3
6,273	4,388	0,970	1,430	M5	14
6,273	4,388	0,970	1,430	M5	15
6,273	4,311	0,973	1,455	M4	9
6,273	4,311	0,956	1,455	M1	5
6,274	4,462	0,964	1,406	M3	18
6,274	4,310	0,954	1,456	M2	12
6,274	4,368	0,957	1,436	M2	8
6,274	4,232	0,918	1,482	M1	7
6,274	4,510	0,975	1,391	M5	7
6,274	4,290	0,947	1,463	M3	9
6,275	4,510	0,972	1,391	M4	4
6,275	4,471	0,976	1,404	M4	12
6,275	4,450	0,974	1,410	M4	15
6,275	4,503	0,966	1,393	M5	8
6,275	4,480	0,982	1,401	M1	17
6,275	4,412	0,960	1,422	M5	3
6,275	4,179	0,945	1,501	M6	7
6,275	4,290	0,930	1,463	M1	14
6,275	4,400	0,977	1,426	M1	5
6,275	4,510	0,969	1,391	M5	22
6,275	4,730	0,983	1,327	M5	16
6,275	4,620	0,972	1,358	M5	43
6,275	4,364	0,966	1,438	M3	11
6,277	4,544	0,965	1,381	M3	3
6,277	4,466	0,981	1,405	M5	17
6,277	4,354	0,961	1,442	M4	11
6,277	4,353	0,962	1,442	M4	14
6,277	4,339	0,953	1,447	M4	12
6,278	4,699	0,972	1,336	M2	21
6,278	4,699	0,972	1,336	M2	23
6,278	4,508	0,968	1,393	M1	7
6,278	4,232	0,953	1,483	M4	6
6,278	4,620	0,964	1,359	M6	30
6,278	4,290	0,955	1,463	M5	16
6,278	4,180	0,960	1,502	M3	15
6,278	4,400	0,967	1,427	M3	11
6,278	4,400	0,960	1,427	M4	5
6,278	4,180	0,968	1,502	M5	30
6,278	4,290	0,957	1,463	M5	7
6,279	4,510	0,965	1,392	M2	22
6,279	4,400	0,957	1,427	M3	5
6,292	4,466	0,964	1,409	M5	5

6,292	4,466	0,961	1,409	M5	11
6,297	4,290	0,971	1,468	M4	10
6,297	4,400	0,961	1,431	M3	15
6,297	4,180	0,950	1,507	M1	7
6,297	4,070	0,944	1,547	M5	6
6,298	4,290	0,950	1,468	M2	9
6,300	4,517	0,967	1,395	M4	11
6,300	4,466	0,978	1,411	M4	9
6,300	4,305	0,955	1,464	M4	15
6,300	4,311	0,950	1,462	M1	5
6,300	4,309	0,947	1,462	M3	7
6,301	4,310	0,956	1,462	M1	5
6,301	4,388	0,959	1,436	M4	15
6,301	4,388	0,973	1,436	M5	22
6,303	4,503	0,968	1,400	M1	13
6,303	4,596	0,959	1,371	M2	25
6,303	4,290	0,952	1,469	M5	37
6,303	4,400	0,965	1,432	M2	19
6,303	4,510	0,971	1,398	M5	36
6,303	4,409	0,958	1,430	M4	19
6,303	4,291	0,961	1,469	M3	7
6,304	4,592	0,961	1,373	M2	21
6,304	4,388	0,969	1,437	M3	9
5,233	3,850	0,958	1,359	M5	12
5,580	3,740	0,948	1,492	M5	19
5,870	4,070	0,959	1,442	M5	22
5,380	3,999	0,970	1,345	M5	7
5,445	4,058	0,918	1,342	M5	8
5,971	4,290	0,943	1,392	M5	5
5,865	4,180	0,950	1,403	M5	7
5,363	4,070	0,976	1,318	M5	8
5,743	4,290	0,986	1,339	M5	9
6,263	4,155	0,938	1,507	M5	3
5,786	4,070	0,934	1,422	M5	11
6,217	4,403	0,966	1,412	M5	15
5,609	4,070	0,964	1,378	M5	6
5,764	4,180	0,972	1,379	M5	9
5,737	4,180	0,958	1,372	M5	11
5,962	4,466	0,981	1,335	M5	7
5,479	4,180	0,972	1,311	M5	10
6,083	4,180	0,943	1,455	M5	11
6,427	4,850	0,955	1,325	M5	12
6,087	4,424	0,962	1,376	M5	14
5,760	4,077	0,925	1,413	M5	7
5,993	4,357	0,962	1,375	M5	8
6,057	4,422	0,951	1,370	M5	10
6,059	4,459	0,957	1,359	M5	7
6,406	4,510	0,943	1,420	M5	7
5,911	4,427	0,928	1,335	M5	11
6,622	4,510	0,947	1,468	M5	12
6,073	4,453	0,944	1,364	M5	17
6,111	4,070	0,953	1,502	M5	10

6,162	3,850	0,933	1,600	M5	6
5,776	3,960	0,921	1,459	M5	10
6,182	4,418	0,959	1,399	M5	11
6,032	4,621	0,960	1,305	M5	20
5,956	4,523	0,969	1,317	M5	21
5,967	4,466	0,953	1,336	M5	29
6,032	4,348	0,973	1,387	M5	49
5,748	4,620	0,982	1,244	M5	52
5,955	4,290	0,986	1,388	M5	8
5,899	3,960	0,916	1,490	M5	11
5,845	4,070	0,958	1,436	M5	10
6,094	4,375	0,934	1,393	M5	11
5,417	3,850	0,936	1,407	M5	21
5,937	4,388	0,985	1,353	M5	43
6,378	4,400	0,930	1,450	M5	25
5,786	4,180	0,975	1,384	M5	27
6,140	4,070	0,930	1,509	M5	2
6,134	4,045	0,903	1,516	M5	4
5,637	4,070	0,934	1,385	M5	12
5,295	3,922	0,948	1,350	M5	5
6,030	4,388	0,953	1,374	M5	3
5,826	4,308	0,953	1,353	M5	8
5,626	3,960	0,946	1,421	M5	9
5,725	4,290	0,954	1,335	M5	11
5,827	4,300	0,951	1,355	M5	5
5,852	4,180	0,949	1,400	M5	15
5,995	4,621	0,964	1,297	M5	22
6,118	4,290	0,954	1,426	M5	5
5,673	4,564	0,954	1,243	M5	6
5,446	4,070	0,940	1,338	M5	8
6,062	4,400	0,956	1,378	M5	9
5,431	4,150	0,979	1,309	M5	11
5,899	4,070	0,959	1,449	M5	3
6,257	4,539	0,941	1,378	M5	6
5,743	4,180	0,979	1,374	M5	15
5,571	4,077	0,942	1,366	M5	4
5,572	4,093	0,960	1,362	M5	7
5,567	4,077	0,907	1,366	M5	8
5,502	3,850	0,958	1,429	M5	8
5,661	4,466	0,983	1,268	M5	6
6,059	4,290	0,972	1,412	M5	8
6,237	4,453	0,969	1,401	M5	9
5,719	4,510	0,995	1,268	M5	10
6,117	4,550	0,983	1,345	M5	12
6,030	4,510	0,997	1,337	M5	2
6,059	4,070	0,967	1,489	M5	4
5,899	4,358	0,976	1,354	M5	6
5,533	4,070	0,976	1,359	M5	7
6,112	4,445	0,969	1,375	M5	10
5,501	4,180	0,999	1,316	M5	4
5,772	4,257	0,981	1,356	M5	5
6,183	4,483	0,973	1,379	M5	8

6,218	4,305	0,953	1,444	M5	9
5,899	4,180	0,969	1,411	M5	13
5,814	4,077	0,949	1,426	M5	15
5,936	4,291	0,973	1,383	M5	17
6,111	4,180	0,959	1,462	M5	7
5,956	4,070	0,965	1,463	M5	8
5,787	4,232	0,986	1,367	M5	11
5,977	4,290	0,975	1,393	M5	15
6,021	4,400	0,972	1,368	M5	7
6,018	4,410	0,973	1,365	M5	8
5,977	4,264	0,971	1,402	M5	9
5,815	3,960	0,964	1,468	M5	13
5,927	4,449	0,985	1,332	M5	3
6,087	4,310	0,977	1,412	M5	5
6,134	4,374	0,965	1,402	M5	8
6,237	4,400	0,979	1,418	M5	10
5,609	4,410	0,992	1,272	M5	11
5,819	4,180	0,974	1,392	M5	18
6,111	4,290	0,962	1,425	M5	3
5,956	4,290	0,972	1,388	M5	6
6,151	4,290	0,955	1,434	M5	7
5,481	3,999	0,978	1,371	M5	9
5,787	4,183	0,967	1,383	M5	6
5,715	4,180	0,984	1,367	M5	7
6,118	4,634	0,982	1,320	M5	8
5,772	3,850	0,946	1,499	M5	9
6,073	4,310	0,970	1,409	M5	11
5,842	4,388	0,977	1,331	M5	13
5,827	4,364	0,979	1,335	M5	4
5,725	4,077	0,972	1,404	M5	5
6,222	4,466	0,974	1,393	M5	6
6,222	4,310	0,947	1,443	M5	3
5,927	4,507	0,992	1,315	M5	6
5,793	4,180	0,987	1,386	M5	7
5,984	4,290	0,961	1,395	M5	10
6,222	4,388	0,960	1,418	M5	11
5,772	4,303	0,979	1,341	M5	12
6,372	4,544	0,961	1,402	M5	16
6,004	4,595	0,963	1,306	M5	20
5,865	4,445	0,986	1,320	M5	21
6,364	4,345	0,924	1,465	M5	2
5,821	4,180	0,963	1,393	M5	6
6,357	4,584	0,966	1,387	M5	7
6,373	4,466	0,948	1,427	M5	9
5,743	4,377	0,962	1,312	M5	6
5,787	4,155	0,978	1,393	M5	7
5,786	4,180	0,970	1,384	M5	8
5,845	4,070	0,965	1,436	M5	9
5,715	4,290	0,992	1,332	M5	11
5,927	4,290	0,983	1,382	M5	15
6,428	4,621	0,960	1,391	M5	18
5,993	4,336	0,971	1,382	M5	6

5,993	4,290	0,973	1,397	M5	8
6,034	4,400	0,972	1,371	M5	10
6,277	4,544	0,974	1,381	M5	11
5,772	4,134	0,974	1,396	M5	13
6,292	4,388	0,959	1,434	M5	14
5,786	4,510	1,000	1,283	M5	5
6,190	4,290	0,968	1,443	M5	12
5,793	4,070	0,961	1,423	M5	4
6,714	4,388	0,910	1,530	M5	8
6,017	4,466	0,980	1,347	M5	9
6,067	4,232	0,963	1,433	M5	10
6,164	4,622	0,959	1,334	M5	14
5,609	3,850	0,959	1,457	M5	2
6,003	4,077	0,965	1,473	M5	3
5,749	4,248	0,987	1,353	M5	5
5,825	4,329	0,987	1,346	M5	12
6,087	4,354	0,972	1,398	M5	7
5,524	4,180	0,974	1,321	M5	8
5,751	4,155	0,970	1,384	M5	9
5,607	4,288	0,959	1,308	M5	10
5,711	4,217	0,966	1,354	M5	16
6,170	4,400	0,959	1,402	M5	17
5,466	4,070	0,978	1,343	M5	5
5,793	4,180	0,970	1,386	M5	8
5,928	4,180	0,952	1,418	M5	13
6,058	4,388	0,971	1,381	M5	2
5,937	4,544	0,992	1,307	M5	4
5,867	3,922	0,948	1,496	M5	6
5,552	4,180	0,976	1,328	M5	8
5,953	4,180	0,967	1,424	M5	9
6,003	4,388	0,967	1,368	M5	12
6,208	4,388	0,961	1,415	M5	16
5,956	4,070	0,968	1,463	M5	4
5,363	3,999	0,982	1,341	M5	6
5,940	4,310	0,977	1,378	M5	10
6,274	4,510	0,975	1,391	M5	14
5,717	4,070	0,962	1,405	M5	15
5,962	4,423	0,960	1,348	M5	17
5,993	4,400	0,989	1,362	M5	7
5,661	4,155	0,965	1,362	M5	9
5,772	4,077	0,972	1,416	M5	2
5,815	4,400	0,989	1,321	M5	5
5,526	4,129	0,981	1,338	M5	7
5,598	4,155	0,981	1,347	M5	2
5,715	4,271	0,982	1,338	M5	7
6,565	4,400	0,962	1,492	M5	9
5,743	4,180	0,977	1,374	M5	13
6,072	4,311	0,986	1,409	M5	5
5,995	4,466	0,982	1,342	M5	6
6,034	4,510	0,976	1,338	M5	7
5,737	4,290	0,994	1,337	M5	8
6,237	4,276	0,954	1,459	M5	4

5,955	4,457	0,981	1,336	M5	8
6,304	4,388	0,974	1,437	M5	22
6,309	4,544	0,960	1,389	M5	8
6,309	4,388	0,955	1,438	M1	11
6,309	4,311	0,940	1,464	M2	18
6,310	4,544	0,963	1,389	M1	13
6,310	4,544	0,963	1,389	M1	14
6,310	4,759	0,987	1,326	M5	14
6,310	4,612	0,974	1,368	M4	9
6,310	4,471	0,958	1,411	M2	6
6,310	4,472	0,967	1,411	M4	12
6,310	4,354	0,965	1,449	M1	12
6,310	4,500	0,971	1,402	M5	41
6,311	4,699	0,985	1,343	M4	8
6,311	4,544	0,970	1,389	M1	10
6,317	4,710	0,983	1,341	M6	15
6,317	4,589	0,979	1,377	M1	6
6,317	4,452	0,947	1,419	M5	5
6,317	4,489	0,968	1,407	M4	5
6,317	4,538	0,978	1,392	M4	5
6,318	4,643	0,966	1,361	M4	11
6,318	4,544	0,975	1,390	M1	4
6,318	4,372	0,946	1,445	M1	7
6,318	4,228	0,935	1,494	M1	9
6,318	4,519	0,955	1,398	M1	9
6,324	4,290	0,949	1,474	M4	13
6,324	4,400	0,970	1,437	M4	14
6,329	3,850	0,935	1,644	M4	19
6,331	4,709	0,979	1,344	M3	10
6,331	4,630	0,957	1,367	M5	5
6,331	4,400	0,973	1,439	M3	13
6,331	4,501	0,957	1,407	M5	9
6,331	4,180	0,964	1,515	M3	8
6,332	4,400	0,961	1,439	M1	7
6,332	4,840	0,984	1,308	M1	9
6,332	4,543	0,961	1,394	M5	14
6,332	4,463	0,958	1,419	M3	12
6,333	4,453	0,964	1,422	M3	3
6,333	4,388	0,968	1,443	M1	7
6,336	4,311	0,966	1,470	M4	7
6,336	4,233	0,945	1,497	M1	3
6,337	4,232	0,956	1,497	M4	10
6,337	4,232	0,952	1,497	M1	3
6,337	4,232	0,952	1,497	M1	3
6,340	4,510	0,967	1,406	M6	9
6,340	4,180	0,949	1,517	M3	6
6,340	4,249	0,947	1,492	M5	31
6,340	4,620	0,958	1,372	M1	8
6,340	4,436	0,951	1,429	M5	15
6,348	4,070	0,935	1,560	M6	5
6,352	4,510	0,977	1,408	M5	3
6,352	4,620	0,972	1,375	M1	14

6,352	4,400	0,967	1,444	M4	1
6,352	4,410	0,915	1,440	M2	8
6,352	4,180	0,938	1,520	M6	8
6,353	4,510	0,982	1,409	M1	6
6,356	4,630	0,966	1,373	M3	7
6,357	4,436	0,974	1,433	M4	19
6,357	4,443	0,969	1,431	M5	16
6,358	4,449	0,970	1,429	M4	11
6,358	4,434	0,972	1,434	M5	3
6,358	4,423	0,967	1,437	M3	4
6,358	4,311	0,959	1,475	M1	7
6,358	4,636	0,960	1,372	M3	6
6,358	4,430	0,957	1,435	M4	11
6,358	4,388	0,950	1,449	M4	13
6,363	4,544	0,955	1,400	M4	4
6,363	4,388	0,958	1,450	M4	5
6,363	4,466	0,967	1,425	M5	17
6,363	4,466	0,962	1,425	M2	30
6,363	4,466	0,962	1,425	M2	34
6,363	4,400	0,968	1,446	M5	5
6,363	4,595	0,973	1,385	M3	5
6,363	4,489	0,962	1,417	M3	14
6,363	4,077	0,945	1,561	M4	5
6,364	4,606	0,978	1,382	M4	2
6,364	4,544	0,977	1,400	M4	8
6,364	4,310	0,963	1,477	M4	11
6,367	4,400	0,963	1,447	M4	5
6,367	4,290	0,961	1,484	M4	10
6,367	3,960	0,913	1,608	M3	10
6,368	4,510	0,970	1,412	M4	9
6,368	4,290	0,941	1,484	M3	7
6,369	4,510	0,971	1,412	M2	5
6,369	4,840	0,995	1,316	M1	21
6,369	4,395	0,966	1,449	M4	16
6,372	4,466	0,960	1,427	M3	3
6,372	4,311	0,949	1,478	M4	3
6,373	4,603	0,970	1,385	M3	7
6,373	4,264	0,947	1,495	M4	28
6,373	4,544	0,948	1,403	M1	8
6,376	4,388	0,956	1,453	M3	7
6,377	4,621	0,975	1,380	M3	13
6,377	4,466	0,964	1,428	M3	13
6,378	4,675	0,976	1,364	M1	6
6,378	4,620	0,964	1,381	M2	29
6,381	4,400	0,916	1,450	M2	22
6,381	4,589	0,954	1,391	M1	8
6,381	4,461	0,948	1,430	M5	9
6,381	4,180	0,958	1,526	M1	12
6,381	4,620	0,958	1,381	M2	13
6,390	4,700	0,981	1,360	M5	15
6,390	4,311	0,956	1,483	M4	8
6,390	4,388	0,962	1,456	M1	6

6,391	4,466	0,954	1,431	M3	7
6,391	4,310	0,929	1,483	M5	10
6,391	4,388	0,962	1,456	M2	15
6,400	4,311	0,949	1,485	M1	15
6,400	4,388	0,948	1,459	M4	10
6,400	4,466	0,968	1,433	M4	7
6,403	4,583	0,967	1,397	M3	11
6,404	4,378	0,966	1,463	M4	14
6,406	4,290	0,962	1,493	M3	5
6,406	4,290	0,962	1,493	M5	10
6,406	4,730	0,978	1,354	M1	4
6,409	4,596	0,959	1,395	M3	8
6,409	4,290	0,966	1,494	M3	3
6,409	4,510	0,972	1,421	M4	6
6,410	4,474	0,962	1,433	M4	7
6,410	4,510	0,969	1,421	M1	11
6,410	4,400	0,966	1,457	M4	3
6,410	4,364	0,961	1,469	M2	9
6,410	4,429	0,954	1,447	M5	8
6,410	4,895	0,978	1,310	M6	22
6,410	4,595	0,943	1,395	M4	7
6,413	4,700	0,970	1,365	M1	8
6,413	4,643	0,976	1,381	M5	7
6,413	4,388	0,950	1,461	M1	20
6,413	4,335	0,959	1,479	M3	6
6,414	4,802	0,955	1,336	M2	23
6,414	4,802	0,955	1,336	M2	25
6,416	4,516	0,970	1,421	M3	10
6,416	4,376	0,954	1,466	M1	3
5,940	4,232	0,974	1,404	M6	3
6,058	4,155	0,969	1,458	M6	4
6,125	4,695	0,985	1,304	M6	5
5,965	4,290	0,978	1,390	M6	6
6,274	4,388	0,965	1,430	M6	8
5,814	4,490	0,980	1,295	M6	11
5,880	3,960	0,953	1,485	M6	13
6,071	4,546	0,979	1,336	M6	14
6,073	4,232	0,968	1,435	M6	17
6,094	4,310	0,963	1,414	M6	18
5,717	4,297	0,931	1,331	M6	20
5,918	4,248	0,970	1,393	M6	24
5,582	4,310	0,985	1,295	M6	25
5,579	3,960	0,957	1,409	M6	2
6,157	4,388	0,959	1,403	M6	3
6,059	4,180	0,969	1,449	M6	8
6,260	4,180	0,952	1,498	M6	13
5,935	4,070	0,952	1,458	M6	2
6,183	4,284	0,966	1,443	M6	7
5,956	4,374	0,977	1,362	M6	8
6,111	4,400	0,968	1,389	M6	11
6,223	4,466	0,974	1,394	M6	15
5,965	4,840	0,957	1,232	M6	18

5,977	4,290	0,970	1,393	M6	21
6,061	4,180	0,963	1,450	M6	22
6,430	4,730	0,965	1,359	M6	24
6,509	4,510	0,977	1,443	M6	26
6,151	4,290	0,955	1,434	M6	9
6,200	4,510	0,978	1,375	M6	11
6,004	4,388	0,971	1,368	M6	15
5,911	4,250	0,971	1,391	M6	17
6,210	4,324	0,961	1,436	M6	20
5,590	3,850	0,952	1,452	M6	23
6,265	4,400	0,964	1,424	M6	27
5,748	4,400	0,999	1,306	M6	4
5,619	4,376	0,993	1,284	M6	11
6,134	4,363	0,966	1,406	M6	12
5,850	4,466	0,985	1,310	M6	13
6,030	4,290	0,979	1,406	M6	14
5,793	4,180	0,975	1,386	M6	13
6,018	4,434	0,980	1,357	M6	15
5,726	4,290	0,982	1,335	M6	16
5,697	4,232	0,967	1,346	M6	18
6,034	4,290	0,979	1,407	M6	19
6,643	4,466	0,941	1,488	M6	2
6,217	4,233	0,955	1,469	M6	3
6,120	4,466	0,967	1,370	M6	5
5,845	4,400	0,975	1,328	M6	7
6,168	4,180	0,966	1,476	M6	10
5,826	4,180	0,944	1,394	M6	13
6,604	4,510	0,951	1,464	M6	3
5,665	4,466	1,000	1,269	M6	4
6,147	4,409	0,961	1,394	M6	7
6,156	4,311	0,966	1,428	M6	10
5,977	4,493	0,984	1,330	M6	13
6,406	5,170	0,970	1,239	M6	15
6,337	4,504	0,957	1,407	M6	16
7,003	4,388	0,932	1,596	M6	24
6,246	4,544	0,977	1,375	M6	25
5,502	4,070	0,985	1,352	M6	4
6,034	4,290	0,965	1,406	M6	5
6,309	4,233	0,959	1,491	M6	6
6,031	4,466	0,972	1,350	M6	8
6,278	4,400	0,966	1,427	M6	10
6,170	4,290	0,965	1,438	M6	11
6,237	4,620	0,976	1,350	M6	14
6,189	4,290	0,959	1,443	M6	15
6,585	4,510	0,945	1,460	M6	2
5,731	4,544	1,000	1,261	M6	3
6,222	4,466	0,970	1,393	M6	6
6,246	4,311	0,972	1,449	M6	8
6,059	4,510	0,990	1,343	M6	11
6,265	5,060	0,991	1,238	M6	12
6,247	4,310	0,956	1,449	M6	14
6,982	4,311	0,928	1,620	M6	21

6,376	4,777	0,968	1,335	M6	22
6,164	4,303	0,957	1,432	M6	4
6,157	4,699	0,963	1,310	M6	9
6,275	4,290	0,957	1,463	M6	2
5,899	4,290	0,983	1,375	M6	3
6,275	4,400	0,963	1,426	M6	5
5,772	4,311	0,987	1,339	M6	6
6,263	4,496	0,957	1,393	M6	9
6,238	4,621	0,957	1,350	M6	13
6,140	4,510	0,963	1,361	M6	14
6,303	4,290	0,956	1,469	M6	15
5,940	4,388	0,974	1,354	M6	16
6,072	4,347	0,975	1,397	M6	21
6,096	4,290	0,965	1,421	M6	2
6,171	4,373	0,961	1,411	M6	3
6,463	4,507	0,960	1,434	M6	4
6,058	4,510	0,985	1,343	M6	6
5,870	4,180	0,986	1,404	M6	7
6,168	4,290	0,972	1,438	M6	8
5,619	4,233	0,975	1,327	M6	9
6,168	4,463	0,975	1,382	M6	12
6,238	4,730	0,962	1,319	M6	16
6,111	4,510	0,953	1,355	M6	17
6,369	4,290	0,951	1,485	M6	18
5,904	4,310	0,975	1,370	M6	19
6,134	4,290	0,968	1,430	M6	2
6,237	4,290	0,959	1,454	M6	3
6,463	4,474	0,962	1,445	M6	4
6,162	4,620	0,981	1,334	M6	7
5,899	4,180	0,982	1,411	M6	8
6,324	4,290	0,960	1,474	M6	10
5,626	4,155	0,986	1,354	M6	11
6,128	4,503	0,976	1,361	M6	14
6,238	4,855	0,963	1,285	M6	18
6,062	4,620	0,951	1,312	M6	19
6,510	4,290	0,951	1,517	M6	20
5,940	4,388	0,962	1,354	M6	21
5,724	4,077	0,964	1,404	M6	2
5,335	3,960	0,951	1,347	M6	5
5,726	3,999	0,948	1,432	M6	6
5,609	3,740	0,913	1,500	M6	8
5,609	4,290	0,986	1,307	M6	9
5,911	3,999	0,919	1,478	M6	13
5,777	3,960	0,904	1,459	M6	6
5,526	3,960	0,907	1,396	M6	8
5,523	3,630	0,922	1,522	M6	8
5,429	3,740	0,921	1,452	M6	10
5,645	4,318	0,965	1,307	M6	12
5,552	4,070	0,953	1,364	M6	13
5,581	3,999	0,947	1,396	M6	8
5,676	4,077	0,955	1,392	M6	9
5,697	4,077	0,929	1,397	M6	7

5,636	4,232	0,972	1,332	M6	13
5,786	3,914	0,939	1,479	M6	10
5,544	4,223	0,974	1,313	M6	16
5,573	4,109	0,954	1,356	M6	4
6,211	4,621	0,979	1,344	M6	3
6,063	4,400	0,964	1,378	M6	6
5,661	4,310	0,976	1,313	M6	8
5,977	4,353	0,968	1,373	M6	6
5,977	4,290	0,967	1,393	M6	8
5,937	4,466	0,966	1,329	M6	15
5,821	4,070	0,964	1,430	M6	20
5,889	4,180	0,962	1,409	M6	7
5,977	4,155	0,958	1,439	M6	11
5,911	4,311	0,975	1,371	M6	8
5,922	4,232	0,971	1,399	M6	11
5,523	4,180	0,969	1,321	M6	9
5,927	4,290	0,984	1,382	M6	15
5,882	4,155	0,965	1,416	M6	6
6,032	4,223	0,950	1,428	M6	10
5,955	4,577	0,979	1,301	M6	11
6,211	4,449	0,961	1,396	M6	5
6,403	4,264	0,924	1,502	M6	7
6,096	4,350	0,960	1,401	M6	9
5,786	4,233	0,954	1,367	M6	6
5,327	3,960	0,963	1,345	M6	14
5,934	4,180	0,908	1,420	M6	23
5,552	3,960	0,939	1,402	M6	7
6,059	4,180	0,914	1,449	M6	10
5,731	4,267	0,960	1,343	M6	5
5,519	4,223	0,971	1,307	M6	7
5,607	3,850	0,955	1,456	M6	6
6,623	4,070	0,910	1,627	M6	11
5,807	3,960	0,933	1,466	M6	10
5,581	4,311	0,966	1,295	M6	11
5,918	4,070	0,951	1,454	M6	5
5,940	4,155	0,940	1,430	M6	9
5,927	4,070	0,943	1,456	M6	10
5,793	4,290	0,953	1,350	M6	12
5,749	4,180	0,978	1,375	M6	9
5,812	4,231	0,967	1,374	M6	10
6,278	4,070	0,917	1,543	M6	8
5,725	4,180	0,983	1,370	M6	9
5,658	4,070	0,955	1,390	M6	3
5,751	4,232	0,977	1,359	M6	7
6,094	4,388	0,957	1,389	M6	9
5,793	4,180	0,948	1,386	M6	8
5,937	4,311	0,957	1,377	M6	13
6,218	4,435	0,957	1,402	M6	5
6,237	4,430	0,957	1,408	M6	7
6,278	4,180	0,942	1,502	M6	18
5,977	4,155	0,949	1,439	M6	3
6,162	4,400	0,973	1,400	M6	6

6,167	4,376	0,952	1,410	M6	8
5,993	4,400	0,974	1,362	M6	10
5,849	4,180	0,970	1,399	M6	11
5,619	4,004	0,934	1,403	M6	3
6,278	4,070	0,925	1,543	M6	9
6,132	4,070	0,940	1,507	M6	14
5,581	4,362	0,967	1,279	M6	4
5,677	4,077	0,968	1,392	M6	7
5,871	4,214	0,955	1,393	M6	10
5,826	4,310	0,979	1,352	M6	11
5,889	4,070	0,970	1,447	M6	12
6,096	4,432	0,957	1,375	M6	14
5,572	4,180	0,971	1,333	M6	15
6,091	4,290	0,961	1,420	M6	16
5,826	4,180	0,971	1,394	M6	17
5,814	4,228	0,958	1,375	M6	18
5,880	4,290	0,968	1,371	M6	19
5,508	4,155	0,976	1,326	M6	22
6,265	4,400	0,966	1,424	M6	28
6,030	4,077	0,933	1,479	M6	4
5,977	4,250	0,963	1,406	M6	6
6,087	4,233	0,964	1,438	M6	10
5,977	4,400	0,945	1,358	M6	13
6,167	4,395	0,957	1,403	M6	17
6,417	4,252	0,937	1,509	M2	6
6,417	4,470	0,953	1,435	M4	4
6,427	4,466	0,954	1,439	M3	7
6,427	4,729	0,949	1,359	M3	15
6,427	4,529	0,953	1,419	M1	7
6,428	4,699	0,971	1,368	M6	13
6,428	4,311	0,966	1,491	M2	17
6,428	4,714	0,971	1,364	M5	7
6,428	4,544	0,970	1,415	M4	3
6,428	4,543	0,969	1,415	M4	5
6,428	4,232	0,936	1,519	M3	8
6,430	4,510	0,959	1,426	M3	8
6,430	3,960	0,923	1,624	M3	11
6,431	4,510	0,952	1,426	M1	13
6,438	4,290	0,959	1,501	M1	17
6,444	4,400	0,955	1,464	M6	29
6,444	4,503	0,965	1,431	M4	15
6,444	4,510	0,938	1,429	M1	8
6,444	4,510	0,938	1,429	M1	8
6,444	4,992	0,959	1,291	M2	23
6,453	4,544	0,987	1,420	M4	11
6,454	4,311	0,959	1,497	M4	3
6,454	4,311	0,947	1,497	M1	10
6,454	4,480	0,976	1,441	M4	4
6,454	4,438	0,959	1,454	M4	3
6,454	4,544	0,968	1,420	M4	4
6,454	4,310	0,952	1,497	M2	8
6,454	4,210	0,923	1,533	M1	12

6,456	4,840	0,981	1,334	M5	32
6,458	4,180	0,931	1,545	M2	20
6,458	4,070	0,946	1,587	M4	11
6,463	4,729	0,971	1,367	M1	7
6,463	4,350	0,952	1,486	M3	6
6,463	4,310	0,938	1,499	M1	11
6,463	4,310	0,968	1,500	M4	17
6,463	4,403	0,961	1,468	M3	6
6,468	4,533	0,963	1,427	M5	18
6,469	4,699	0,967	1,377	M1	17
6,469	4,443	0,936	1,456	M4	6
6,469	4,466	0,951	1,448	M1	3
6,481	4,442	0,955	1,459	M1	9
6,481	4,310	0,948	1,504	M1	14
6,481	4,645	0,961	1,395	M5	14
6,481	4,742	0,938	1,367	M2	28
6,482	4,840	0,961	1,339	M2	39
6,483	4,621	0,966	1,403	M3	15
6,483	4,390	0,959	1,477	M4	6
6,483	4,388	0,949	1,478	M3	7
6,487	4,400	0,937	1,474	M6	13
6,490	4,544	0,962	1,428	M1	7
6,490	4,544	0,956	1,428	M5	9
6,490	4,310	0,949	1,506	M4	4
6,495	4,924	0,950	1,319	M1	13
6,503	4,508	0,961	1,443	M4	8
6,503	4,699	0,921	1,384	M3	23
6,509	4,950	0,946	1,315	M3	24
6,509	4,376	0,956	1,487	M3	4
6,509	4,201	0,935	1,549	M3	17
6,509	4,636	0,966	1,404	M5	3
6,509	4,406	0,944	1,478	M2	34
5,852	4,235	0,943	1,382	M6	5
5,880	3,850	0,908	1,527	M6	6
5,865	4,180	0,932	1,403	M6	7
5,396	3,630	0,967	1,487	M6	14
6,208	3,909	0,920	1,588	M6	17
5,634	3,921	0,930	1,437	M6	6
6,162	4,609	0,951	1,337	M6	9
6,208	4,244	0,958	1,463	M6	11
5,826	3,850	0,936	1,513	M6	9
5,922	4,233	0,967	1,399	M6	14
5,826	4,210	0,968	1,384	M6	6
5,645	4,180	0,953	1,350	M6	11
5,637	3,921	0,969	1,437	M6	21
6,275	4,290	0,946	1,463	M6	5
6,127	3,921	0,932	1,563	M6	6
5,927	3,960	0,940	1,497	M6	12
6,096	4,188	0,939	1,455	M6	13
5,912	4,264	0,937	1,387	M6	16
5,852	4,400	0,979	1,330	M6	17
6,121	4,077	0,944	1,501	M6	23

5,889	4,364	0,968	1,349	M6	25
5,928	4,489	0,922	1,320	M6	3
6,167	4,077	0,924	1,513	M6	4
6,218	4,180	0,943	1,487	M6	5
5,819	4,077	0,913	1,427	M6	6
5,786	4,070	0,965	1,422	M6	13
5,904	4,310	0,965	1,370	M6	14
6,536	4,400	0,952	1,486	M6	21
6,275	4,699	0,956	1,335	M6	2
6,030	4,229	0,948	1,426	M6	9
6,200	4,180	0,936	1,483	M6	10
6,111	4,381	0,968	1,395	M6	14
6,071	4,125	0,947	1,472	M6	8
6,413	4,720	0,973	1,359	M6	12
6,096	4,180	0,945	1,458	M6	13
5,899	4,290	0,959	1,375	M6	15
6,318	4,388	0,954	1,440	M6	16
6,164	4,204	0,961	1,466	M6	17
6,211	4,220	0,942	1,472	M6	7
6,157	4,488	0,963	1,372	M6	8
6,034	4,180	0,942	1,443	M6	10
5,841	4,311	0,975	1,355	M6	5
5,658	3,850	0,940	1,470	M6	3
5,749	4,699	0,955	1,223	M6	5
5,899	3,630	0,905	1,625	M6	6
5,819	4,290	0,982	1,356	M6	8
5,880	4,180	0,967	1,407	M6	9
5,993	4,503	0,982	1,331	M6	12
5,852	4,290	0,978	1,364	M6	14
5,743	3,960	0,968	1,450	M6	15
5,918	4,290	0,928	1,380	M6	16
6,164	4,727	0,982	1,304	M6	17
6,182	4,479	0,974	1,380	M6	23
6,112	4,311	0,978	1,418	M6	26
7,166	4,834	0,921	1,482	M6	5
5,845	4,510	0,960	1,296	M6	6
6,517	4,466	0,921	1,459	M6	7
6,476	4,180	0,944	1,549	M6	10
6,706	4,408	0,911	1,521	M6	11
6,430	4,180	0,944	1,538	M6	12
6,564	4,555	0,937	1,441	M6	13
6,148	4,232	0,933	1,452	M6	15
6,428	4,466	0,935	1,439	M6	17
6,162	3,960	0,940	1,556	M6	5
6,091	4,070	0,949	1,496	M6	7
5,845	3,960	0,949	1,476	M6	9
6,457	4,510	0,949	1,432	M6	10
6,157	4,388	0,959	1,403	M6	3
6,297	4,355	0,949	1,446	M6	6
6,391	4,155	0,915	1,538	M6	5
6,112	4,155	0,953	1,471	M6	11
5,977	4,155	0,943	1,438	M6	7

6,111	3,999	0,962	1,528	M6	8
5,940	4,310	0,960	1,378	M6	31
5,426	3,997	0,972	1,357	M6	5
5,814	4,303	0,959	1,351	M6	10
6,062	4,070	0,946	1,489	M6	11
6,297	4,400	0,950	1,431	M6	14
5,870	4,070	0,962	1,442	M6	15
6,564	4,438	0,923	1,479	M6	13
6,006	4,257	0,945	1,411	M6	5
6,381	4,070	0,936	1,568	M6	6
5,955	4,180	0,955	1,425	M6	8
6,223	4,381	0,947	1,420	M6	13
5,917	4,274	0,968	1,384	M6	14
5,852	4,180	0,972	1,400	M6	2
6,157	4,643	0,989	1,326	M6	8
5,479	4,290	1,000	1,277	M6	9
6,167	4,622	0,973	1,334	M6	11
6,162	4,400	0,961	1,401	M6	12
5,751	4,440	0,984	1,295	M6	13
5,793	4,290	0,968	1,350	M6	14
6,164	4,290	0,932	1,437	M6	18
6,318	4,635	0,986	1,363	M6	19
5,697	4,232	0,970	1,346	M6	20
5,850	4,310	0,965	1,357	M6	21
5,787	4,388	0,972	1,319	M6	25
5,526	4,311	1,000	1,282	M6	3
5,927	4,622	0,985	1,282	M6	6
5,877	4,310	0,977	1,364	M6	8
6,509	4,564	0,950	1,426	M6	9
5,841	4,388	0,979	1,331	M6	12
6,247	4,077	0,929	1,532	M6	18
5,731	4,310	0,974	1,330	M6	19
5,984	4,290	0,985	1,395	M6	21
5,825	4,231	0,972	1,377	M6	4
6,378	4,383	0,926	1,455	M6	6
5,956	4,180	0,958	1,425	M6	7
5,956	4,290	0,978	1,388	M6	9
6,301	4,310	0,944	1,462	M6	10
6,340	4,582	0,955	1,384	M6	12
5,956	4,290	0,977	1,388	M6	13
5,580	4,292	0,985	1,300	M6	18
5,918	4,290	0,946	1,380	M6	3
6,134	4,510	0,980	1,360	M6	7
5,899	4,290	0,965	1,375	M6	9
5,744	4,180	0,972	1,374	M6	10
6,011	4,290	0,927	1,401	M6	11
5,956	4,510	0,983	1,321	M6	13
6,059	4,620	0,977	1,311	M6	18
5,890	4,070	0,957	1,447	M6	9
5,977	4,544	0,976	1,316	M6	10
5,645	3,960	0,970	1,425	M6	12
6,072	4,264	0,924	1,424	M6	7

5,977	4,070	0,965	1,468	M6	9
5,454	4,155	0,982	1,313	M6	11
5,985	4,180	0,947	1,432	M6	12
5,825	4,367	0,983	1,334	M6	4
5,963	4,310	0,971	1,383	M6	6
5,609	4,070	0,959	1,378	M6	7
6,034	4,510	0,973	1,338	M6	9
5,972	4,503	0,965	1,326	M6	3
5,977	4,449	0,968	1,343	M6	4
6,111	4,510	0,970	1,355	M6	8
5,982	4,388	0,957	1,363	M6	6
6,171	4,284	0,971	1,441	M6	9
6,113	4,388	0,972	1,393	M6	16
5,743	4,290	0,988	1,339	M6	5
6,349	4,620	0,963	1,374	M6	6
5,940	4,466	0,975	1,330	M6	8
6,030	4,290	0,972	1,406	M6	10
6,094	4,388	0,964	1,389	M6	18
5,814	4,466	0,978	1,302	M6	19
5,881	4,564	0,985	1,289	M6	5
6,337	4,310	0,951	1,470	M6	7
5,711	4,400	0,988	1,298	M6	8
5,972	4,607	0,990	1,296	M6	10
6,132	4,400	0,947	1,394	M6	13
5,826	4,388	0,988	1,328	M6	17
6,124	4,510	0,966	1,358	M6	5
5,880	4,290	0,976	1,371	M6	7
5,993	4,604	0,981	1,302	M6	8
6,168	4,232	0,968	1,457	M6	9
5,793	4,290	0,984	1,350	M6	11
5,911	4,466	0,978	1,324	M6	13
5,880	4,070	0,960	1,445	M6	16
6,062	4,290	0,953	1,413	M6	5
6,066	4,388	0,980	1,382	M6	6
5,825	4,290	0,973	1,358	M6	9
6,171	4,290	0,981	1,439	M6	15
5,955	4,395	0,968	1,355	M6	4
5,882	4,354	0,965	1,351	M6	6
5,634	4,174	0,981	1,350	M6	8
5,889	4,290	0,961	1,373	M6	4
5,814	4,365	0,979	1,332	M6	9
5,867	4,802	0,979	1,222	M6	10
5,726	4,303	0,972	1,331	M6	11
5,871	4,443	0,981	1,322	M6	13
5,882	4,388	0,970	1,341	M6	14
6,246	4,290	0,952	1,456	M6	15
5,766	4,077	0,967	1,414	M6	16
5,899	4,180	0,967	1,411	M6	17
5,871	4,311	0,987	1,362	M6	18
5,912	4,466	0,984	1,324	M6	19
6,067	4,310	0,959	1,408	M6	20
6,018	4,381	0,963	1,374	M6	21

6,182	4,311	0,947	1,434	M6	23
5,865	4,311	0,968	1,361	M6	24
6,111	4,311	0,978	1,418	M6	25
5,899	4,400	0,979	1,341	M6	27
5,827	4,233	0,975	1,377	M6	6
6,021	4,423	0,974	1,361	M6	8
5,827	4,300	0,982	1,355	M6	10
6,112	4,622	0,974	1,323	M6	11
6,030	4,396	0,974	1,372	M6	12
5,607	4,290	0,965	1,307	M6	13
5,977	4,388	0,974	1,362	M6	14
6,264	4,388	0,966	1,428	M6	20
5,850	4,365	0,958	1,340	M6	5
5,927	4,390	0,979	1,350	M6	6
5,850	4,232	0,959	1,382	M6	7
5,819	4,290	0,986	1,356	M6	8
5,971	4,446	0,980	1,343	M6	7
6,183	4,388	0,975	1,409	M6	9
5,658	4,510	0,978	1,255	M6	15
6,407	4,510	0,950	1,421	M6	19
6,246	4,570	0,948	1,367	M6	5
5,845	4,180	0,956	1,398	M6	6
5,609	4,070	0,978	1,378	M6	11
6,189	4,180	0,934	1,481	M6	8
5,922	4,544	0,981	1,303	M6	12
6,091	4,290	0,965	1,420	M6	7
6,058	4,544	0,972	1,333	M6	15
5,977	4,290	0,931	1,393	M6	3
5,955	4,503	0,984	1,323	M6	11
6,005	4,510	0,974	1,332	M6	13
5,814	4,248	0,979	1,369	M6	3
5,995	4,601	0,969	1,303	M6	5
6,030	4,456	0,965	1,353	M6	6
5,764	4,510	0,995	1,278	M6	9
5,827	4,250	0,968	1,371	M6	14
5,865	4,544	0,979	1,291	M6	17
5,918	4,438	0,979	1,333	M6	21
5,786	4,514	0,999	1,282	M6	22
5,967	4,232	0,962	1,410	M6	5
5,821	4,180	0,969	1,393	M6	6
5,827	4,510	0,991	1,292	M6	8
6,169	4,620	0,981	1,335	M6	11
5,918	4,150	0,971	1,426	M6	12
5,927	4,290	0,978	1,382	M6	14
6,509	4,406	0,944	1,478	M2	38
6,516	4,400	0,949	1,481	M5	16
6,516	4,180	0,927	1,559	M6	9
6,517	4,311	0,954	1,512	M1	3
6,517	4,466	0,950	1,459	M3	14
6,517	4,544	0,945	1,434	M5	3
6,517	4,544	0,945	1,434	M5	3
6,517	4,310	0,958	1,512	M5	26

6,519	4,794	0,971	1,360	M1	9
6,519	4,755	0,970	1,371	M5	25
6,519	4,771	0,968	1,366	M5	22
6,523	4,700	0,968	1,388	M5	24
6,523	4,466	0,963	1,460	M1	14
6,523	4,410	0,955	1,479	M1	14
6,523	4,354	0,938	1,498	M4	4
6,523	4,466	0,951	1,461	M4	9
6,523	4,544	0,954	1,436	M3	8
6,523	4,310	0,906	1,513	M2	17
6,532	4,388	0,947	1,489	M5	23
6,536	5,720	0,954	1,143	M2	5
6,544	4,932	0,982	1,327	M1	6
6,544	4,769	0,950	1,372	M2	17
6,544	4,769	0,950	1,372	M2	19
6,545	4,290	0,941	1,526	M4	29
6,547	4,649	0,982	1,408	M1	5
6,547	4,442	0,957	1,474	M3	7
6,556	4,734	0,962	1,385	M3	16
6,563	4,822	0,976	1,361	M5	17
6,563	4,536	0,955	1,447	M5	11
6,563	4,620	0,962	1,421	M3	5
6,563	4,508	0,952	1,456	M4	8
6,565	4,400	0,956	1,492	M1	9
6,575	4,510	0,933	1,458	M1	6
6,575	4,217	0,940	1,559	M4	14
6,580	4,481	0,951	1,469	M5	13
6,580	4,544	0,948	1,448	M1	11
6,585	4,705	0,954	1,399	M1	14
6,602	4,544	0,964	1,453	M1	11
6,602	4,610	0,975	1,432	M4	22
6,603	4,180	0,936	1,580	M4	7
6,604	4,516	0,956	1,462	M1	4
6,604	4,519	0,941	1,462	M4	13
6,607	4,233	0,941	1,561	M2	9
6,609	4,236	0,935	1,560	M4	33
6,613	4,611	0,961	1,434	M5	31
6,613	4,290	0,956	1,541	M4	10
6,613	4,621	0,913	1,431	M2	6
6,618	4,426	0,950	1,495	M4	17
6,618	4,426	0,950	1,495	M4	17
6,622	4,400	0,946	1,505	M4	16
6,622	4,290	0,948	1,544	M4	24
6,622	4,290	0,915	1,544	M4	20
6,634	4,798	0,917	1,383	M1	14
6,634	4,617	0,958	1,437	M1	6
6,643	4,290	0,904	1,548	M3	9
6,643	4,466	0,928	1,488	M3	4
6,643	4,310	0,922	1,541	M2	3
6,649	4,795	0,975	1,387	M3	10
6,650	4,510	0,957	1,475	M3	4
6,659	4,599	0,954	1,448	M5	12

6,659	4,436	0,946	1,501	M3	7
6,659	4,433	0,953	1,502	M1	6
6,663	4,290	0,908	1,553	M1	16
6,673	5,885	0,976	1,134	M1	7
6,674	4,621	0,958	1,444	M5	7
6,688	4,688	0,934	1,427	M2	13
6,697	4,764	0,952	1,406	M1	8
6,700	5,127	0,940	1,307	M2	35
6,706	4,311	0,934	1,556	M4	14
6,709	4,642	0,951	1,445	M2	10
6,714	4,621	0,958	1,453	M4	9
6,714	4,155	0,914	1,616	M4	11
6,714	4,621	0,966	1,453	M5	7
6,733	4,830	0,931	1,394	M1	13
6,733	4,466	0,948	1,508	M1	10
6,754	4,400	0,934	1,535	M1	13
6,754	4,730	0,961	1,428	M3	7
6,754	4,620	0,962	1,462	M4	8
6,754	4,609	0,962	1,466	M3	17
6,769	4,466	0,950	1,516	M5	19
6,788	4,388	0,903	1,547	M1	4
6,796	4,444	0,932	1,529	M5	5
6,802	5,060	0,974	1,344	M5	26
6,809	4,470	0,946	1,523	M4	3
6,809	4,489	0,932	1,517	M4	14
6,849	4,388	0,943	1,561	M3	8
6,913	4,290	0,902	1,611	M1	4
6,919	4,444	0,941	1,557	M4	8
6,941	4,722	0,956	1,470	M3	12
7,166	5,010	0,950	1,430	M1	13
7,600	4,895	0,912	1,553	M5	9
